# Sex- and Tissue-Specific Expression Profiles of Odorant Binding Protein and Chemosensory Protein Genes in *Bradysia odoriphaga* (Diptera: Sciaridae)

**DOI:** 10.3389/fphys.2018.00107

**Published:** 2018-04-03

**Authors:** Yunhe Zhao, Jinfeng Ding, Zhengqun Zhang, Feng Liu, Chenggang Zhou, Wei Mu

**Affiliations:** ^1^College of Plant Protection, Shandong Provincial Key Laboratory for Biology of Vegetable Diseases and Insect Pests, Shandong Agricultural University, Tai'an, China; ^2^College of Horticultural Science and Engineering, Shandong Agricultural University, Tai'an, China; ^3^College of Plant Protection, Shandong Agricultural University, Tai'an, China

**Keywords:** *Bradysia odoriphaga*, odorant binding protein, chemosensory protein, expression profiles analysis, transcriptomes

## Abstract

*Bradysia odoriphaga* is an agricultural pest insect affecting the production of Chinese chive and other liliaceous vegetables in China, and it is significantly attracted by sex pheromones and the volatiles derived from host plants. Despite verification of this chemosensory behavior, however, it is still unknown how *B. odoriphaga* recognizes these volatile compounds on the molecular level. Many of odorant binding proteins (OBPs) and chemosensory proteins (CSPs) play crucial roles in olfactory perception. Here, we identified 49 OBP and 5 CSP genes from the antennae and body transcriptomes of female and male adults of *B. odoriphaga*, respectively. Sequence alignment and phylogenetic analysis among Dipteran OBPs and CSPs were analyzed. The sex- and tissue-specific expression profiles of 54 putative chemosensory genes among different tissues were investigated by quantitative real-time PCR (qRT-PCR). qRT-PCR analysis results suggested that 22 OBP and 3 CSP genes were enriched in the antennae, indicating they might be essential for detection of general odorants and pheromones. Among these antennae-enriched genes, nine OBPs (*BodoOBP2/4/6/8/12/13/20/28/33*) were enriched in the male antennae and may play crucial roles in the detection of sex pheromones. Moreover, some OBP and CSP genes were enriched in non-antennae tissues, such as in the legs (*BodoOBP3/9/19/21/34/35/38/39/45* and *BodoCSP1*), wings (*BodoOBP17/30/32/37/44*), abdomens and thoraxes (*BodoOBP29/36*), and heads (*BodoOBP14/23/31* and *BodoCSP2*), suggesting that these genes might be involved in olfactory, gustatory, or other physiological processes. Our findings provide a starting point to facilitate functional research of these chemosensory genes in *B. odoriphaga* at the molecular level.

## Introduction

The Chinese chive maggot, *Bradysia odoriphaga* (Diptera: Sciaridae), is the major destructive pest of Chinese chive and other liliaceous vegetables in China (Zhang et al., 2015; Chen et al., 2017). The larvae of this pest feed on the underground roots, bulbs, and immature stems of Chinese chive and cause yield losses of more than 50% in the absence of insecticide protection (Ma et al., 2013). Thus far, the application of chemical insecticides remains the primary measure for controlling *B. odoriphaga*, and it has led to many adverse impacts, such as widespread insecticide resistance and toxic residues in chives, threatening consumer health (Zhang P. et al., 2016; Chen et al., 2017). Hence, a new ecofriendly pest management strategy is needed to control this pest. Previous studies have shown that *B. odoriphaga* was significantly attracted by sex pheromones, the volatiles derived from host plants and microbial secondary metabolites (Li et al., 2007; Chen et al., 2014; Uddin, 2016; Zhang Z. J. et al., 2016), and that it was repelled by green leaf volatile compounds (Chen C. Y. et al., 2015). Moreover, *B. odoriphaga* exhibited a strong electroantennogram (EAG) response to *trans*-2-hexenal and benzothiazole (Chen C. Y. et al., 2015). The evidence from these behavioral responses contribute to control of this pest using push-pull strategies (Cook et al., 2007). Despite these reports on chemosensory behavior, however, the mechanism by which *B. odoriphaga* recognizes these volatile compounds on the molecular level is still unknown.

Olfaction is the primary sensory modality in insects and plays a crucial role in various physiological behaviors, such as locating sexual partners, food sources, oviposition sites, and avoiding predators (Visser, 1986; Leal, 2013). The antennae are the principal olfactory organs for insect olfaction, and the olfactory perception process generally includes two main steps. First, odorant molecular penetrate into the sensillar lymph through pores, and they are bound by small, amphipathic proteins [odorant binding proteins (OBPs) or chemosensory proteins (CSPs); (Pelosi et al., 2006; Zhou, 2010; He et al., 2017)]. Second, the OBPs or CSPs will transfer the odorant molecule through the sensillar lymph to the olfactory receptors (ORs), activate the olfactory receptor neurons (ORNs) and convert chemical signals into electrical signals that are sent to the insect brain (Vogt et al., 1999; Leal, 2013; Pelosi et al., 2018). Hence, OBPs and CSPs are very important because they mediate the first step of odor perception (Li et al., 2015; Brito et al., 2016).

The first step toward understanding the molecular mechanism of olfactory perception process is to investigate olfaction-related genes, which encode the proteins that function in odorant molecular detection. Since OBPs and CSPs were identified and characterized in the model insect, *Drosophila melanogaster* (Robertson et al., 2003), a large number of OBP and CSP genes have been identified from diverse families of Diptera insects, including sanitary pests (Pelletier and Leal, 2011; Manoharan et al., 2013; Rinker et al., 2013; Scott et al., 2014; Chen X. G. et al., 2015; Leitch et al., 2015; He X. et al., 2016), agricultural pests (Andersson et al., 2014; Gong et al., 2014; Ohta et al., 2014, 2015; Elfekih et al., 2016; Liu et al., 2016), and predators (Wang et al., 2017). Furthermore, the functions of some OBP and CSP genes in the olfactory perception process of insects have been predicted and verified (Swarup et al., 2011; Siciliano et al., 2014; Wu et al., 2016; Zhu et al., 2016). However, thus far, only two OBP genes and one CSP gene have been identified in *B. odoriphaga* from Sciaridae, and the number, classification, expression characteristics and functions of OBP and CSP genes in *B. odoriphaga* are still unknown.

In the present study, we performed transcriptome analysis of the antennae and body of female and male adult of *B. odoriphaga*, respectively, and identified 54 putative chemosensory genes comprising 49 OBPs and 5 CSPs. Then, sequence alignment and phylogenetic analysis were undertaken among Dipteran OBPs and CSPs. The transcript expression levels of 54 putative chemosensory genes among different tissues (female antennae, male antennae, legs, wings, abdomens and thoraxes, and heads) were investigated by quantitative real-time PCR (qRT-PCR) (Graphical Abstract). This work provides a starting point to facilitate functional studies of these OBP and CSP genes in *B. odoriphaga* at the molecular level.

**Graphical Abstract:**
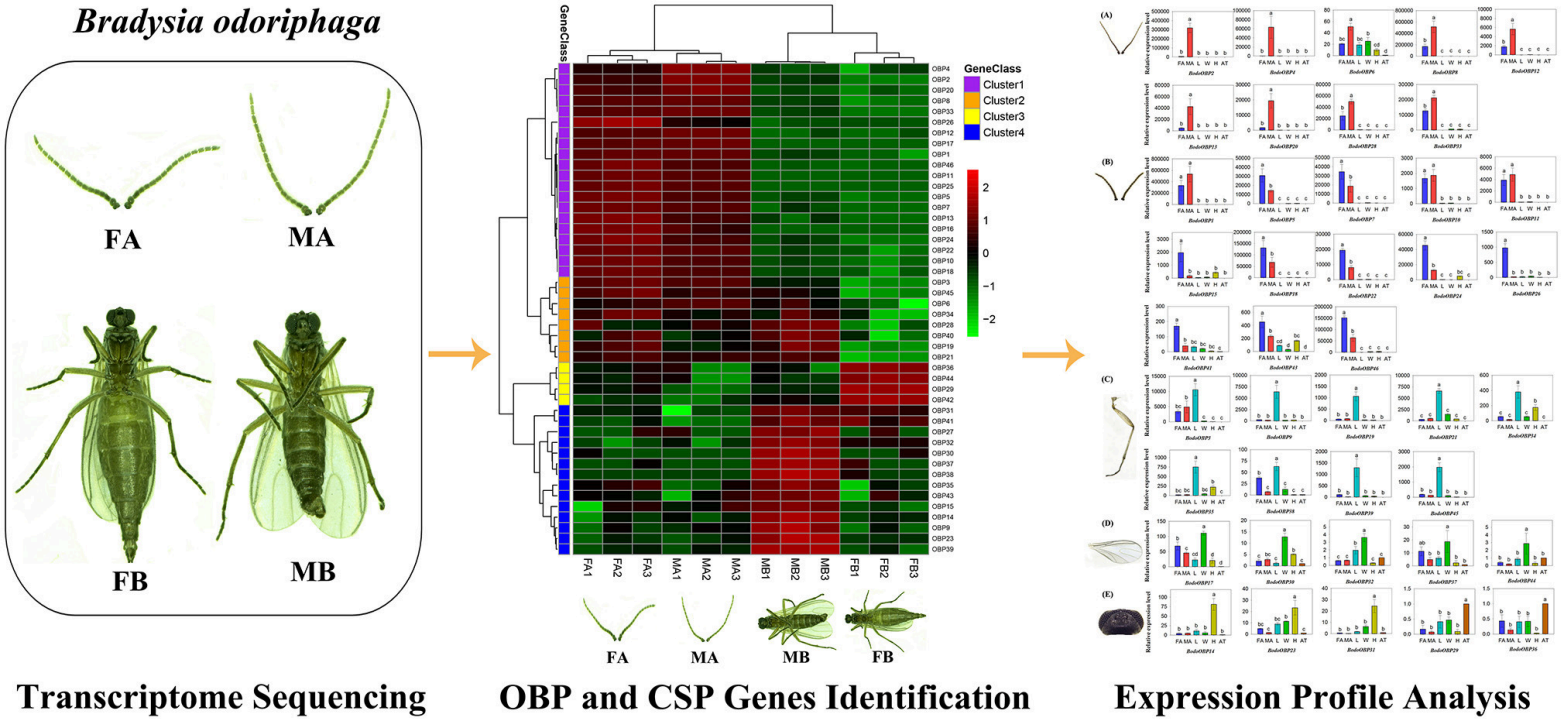
Identification and expression profiles analysis of odorant binding protein and chemosensory protein genes in Bradysia odoriphaga.

## Materials and methods

### Insect culture and tissue collection

A laboratory colony of *B. odoriphaga* was collected from a Chinese chive field in Liaocheng, Shandong Province, China (36°02′N, 115°30′E) in April 2013. The insects were reared on fresh chive rhizomes and placed in Petri dishes, which were maintained at 25 ± 1°C, 70 ± 5% RH with a photoperiod of 14:10 h (L:D) in a climate-controlled chamber. The antennae and the remaining body parts (mixture of heads, thoraxes, abdomens, legs and wings) of female and male adults were separated quickly and then stored in liquid nitrogen until RNA extraction (female antennae: FA; male antennae: MA; female body: FB; male body: MB). Approximately 1,000 antennae and 30 bodies of females and males were collected for RNA extraction, and three biological replicates were performed.

### RNA isolation, cDNA library construction, and illumina sequencing

Total RNA was isolated from antennae and bodies using Trizol reagent (Invitrogen, Carlsbad, CA, USA), according to the manufacturer's instructions. Then, all the RNA samples were treated with DNase I (Invitrogen, Carlsbad, CA, USA) to eliminate the genomic DNA. The concentration of isolated RNA was measured with a NanoDrop ND-2000 spectrophotometer (NanoDrop products, Wilmington, DE, USA), and the integrity of RNA extractions were determined by agarose gel electrophoresis. cDNA library construction was performed using a TruseqTM RNA sample prep Kit (Illumina, San Diego, CA, USA) and was sequenced on an Illumina HiSeq 4000 (Illumina, San Diego, CA, USA). After removing the low quality and adapter sequences, clean short reads were mapped to contigs, and contigs were assembled to unigenes by the short-read assembly program Trinity (Grabherr et al., 2011). Then, unigenes were annotated using different databases, including the non-redundant protein (Nr), nucleotide sequence (Nt), Swiss-Prot, Clusters of Orthologous Groups (COG), Kyoto Encyclopedia of Genes and Genomes (KEGG), and Gene Ontology (GO) databases (E-value < 10^−5^).

### Identification and comparison of transcript abundance of OBP and CSP genes

The tBLASTn program was used to identify candidate unigenes that encode putative OBPs and CSPs from the antennae, body transcriptomes and fourth instar larval transcriptome of *B. odoriphaga* (unpublished data). All putative OBP and CSP genes were confirmed by the BLASTx program at the National Center for Biotechnology Information (NCBI, http://blast.ncbi.nlm.nih.gov/Blast.cgi). The open reading frames (ORFs) of OBP and CSP genes were predicted by the ORF Finder (https://www.ncbi.nlm.nih.gov/orffinder/). The conserved domains of these candidate OBPs and CSPs were predicted utilizing SMART (http://smart.embl.de; Letunic and Bork, 2017).

To compare the expression levels of the candidate OBP and CSP genes in the antennae and body transcriptomes (FA, MA, FB, and MB) of *B. odoriphaga*, the FPKM (fragments per kilobase of exon per million fragments mapped) values were used for calculating transcript abundance (Andersson et al., 2014). Heatmaps of gene expression for different OBPs among FA, MA, FB and MB were generated by R version 3.4.1 (R Development Core Team, The R Foundation for Statistical Computing, Vienna, Austria).

### Verification of the OBP and CSP sequences by cloning and sequencing

All the putative OBP and CSP nucleotide sequences obtained from the *B. odoriphaga* transcriptomes were confirmed by gene cloning and sequencing. Gene-specific primers were designed to amplify the complete or partial ORF sequences of each OBP and CSP gene (Table S1). The cDNA template was synthesized by the *TransScript*® All-in-One First-Strand cDNA Synthesis SuperMix for PCR Kit (TransGen Biotech, Beijing, China). PCR amplification was performed in a 25 μl volume containing 2.0 μl of cDNA (300 ng), 0.5 μl of *TransScript*® KD Plus DNA polymerase (TransGen Biotech, Beijing, China), 5 μl of 5 × *TransScript*® KD Plus Buffer, 2 μl of dNTPs (2.5 mM), 0.5 μl each of the forward and reverse primers (10 μM), and 14.5 μl of nuclease free H_2_O. The cycling conditions were an initial denaturation at 94°C for 3 min, followed by 35 cycles of 94°C for 30 s, 56°C for 30 s, 68°C for 45 s, and a final extension at 68°C for 10 min. Then, the PCR products were purified by agarose gel electrophoresis and an *EasyPure*® Quick Gel Extraction Kit (TransGen Biotech, Beijing, China), and subcloned into the *pEASY*®-Blunt cloning vector (TransGen Biotech, Beijing, China) and sequenced.

### Sequence and phylogenetic analysis

The putative N-terminal signal peptides of BodoOBPs and BodoCSPs were predicted by the SignalP V 4.1 program (http://www.cbs.dtu.dk/services/SignalP/; Nielsen, 2017). Multiple alignments and identity calculation were conducted by Clustal X 2.0 software (Larkin et al., 2007). A total of 280 OBP protein sequences from four Diptera species were used to construct the phylogenetic tree, including 49 OBPs from *B. odoriphaga* identified in this study, 51 OBPs of *D. melanogaster*, 69 OBPs of *Anopheles gambiae*, and 111 OBPs of *Aedes aegypti* (Sequences are listed in Table S2). In addition, 97 CSP protein sequences from seven Diptera species were used for the phylogenetic analysis, including 5 CSPs of *B. odoriphaga* identified in the present study, 4 CSPs of *D. melanogaster*, 8 CSPs of *A. gambiae*, 8 CSPs of *Anopheles sinensis*, 43 CSPs of *A. aegypti*, 27 CSPs of *Culex quinquefasciatus*, and 2 CSPs of *D. antiqua* (sequences are listed in Table S3). All the phylogenetic trees were constructed by MEGA 6.0 software with the neighbor-joining method using default settings and 1,000 bootstrap replications (Tamura et al., 2013). The final phylogenetic tree was visualized by an online tool, EvolView (He Z. L. et al., 2016).

### Motif analysis

A total of 318 OBPs (from 6 Diptera species) and 138 CSPs (from 18 Diptera species) were used for comparing the motif pattern between Diptera OBPs and CSPs. All OBP and CSP sequences (Table S4) with intact ORFs were used for motif discovery and pattern analysis. The protein motifs analysis was performed using the MEME (version 4.12.0) online server (http://meme-suite.org; Bailey et al., 2015). The parameters used for motif discovery were: minimum width = 6, maximum width = 10, and the maximum number of motifs to find = 8.

### Tissue expression profile analysis

The expression profiles for different tissues of these 49 OBPs and 5 CSPs were evaluated by qRT-PCR. The female antennae (FA), male antennae (MA), legs (L), wings (W), abdomens and thoraxes (AT), and heads (H) were collected from adult *B. odoriphaga* after eclosion without mating. Total RNA was isolated from different tissues using Trizol reagent (Invitrogen, Carlsbad, CA, USA), according to the manufacturer's instructions. The cDNA template was synthesized by the *TransScript*® All-in-One First-Strand cDNA Synthesis SuperMix for qPCR (One-Step gDNA Removal) Kit (TransGen Biotech, Beijing, China). Specific primers used for qRT-PCR were designed by the software Beacon Designer 7.90 (PREMIER Biosoft International) and are listed in Table S5. Two reference genes, RPS15 (ribosomal protein S15) and RPL18 (ribosomal protein L18) were used for normalizing target gene expression and to correct for sample-to-sample variation (Shi et al., 2016). The experiment was conducted using the LightCycler® 96 System (Roche Molecular Biochemicals, Lewes, United Kingdom) and each reaction was conducted in a 20 μl reaction mixture containing 1.0 μl of sample cDNA (150 ng), 10 μl of Mix (2 × *TransScript*® Tip Green qPCR SuperMix) (TransGen Biotech, Beijing, China), 1.0 μl of forward primer (10 μM), 1.0 μl of reverse primer (10 μM), and 7 μl of nuclease free H_2_O. The reaction programs were as follows: 95°C for 10 min, followed by 45 cycles of amplification (95°C for 10 s and 60°C for 30 s). Then, a melting curve was analyzed for PCR products to detect a single gene-specific peak and to check for the absence of primer dimer peaks. Negative controls were non-template reactions (replacing cDNA with H_2_O). Three technical replicates and three biological replicates were conducted for all experiments.

The results were analyzed using the LightCycler® 96 software. Relative quantification of different tissues was calculated by the comparative 2^−ΔΔCt^ method (Livak and Schmittgen, 2001). Comparative analyses of each target gene among different tissues were determined using one-way ANOVA tests followed by Tukey's HSD method using SPSS statistical software (version 18.0, SPSS Inc., Chicago, IL, USA) (*P* < 0.05). When applicable, the values are shown as the mean ± SE.

## Results

### Overview of the transcriptome of *B. odoriphaga*

A total of 42.6 GB of clean data was obtained from the antennae and body transcriptomes of *B. odoriphaga*. After assembling all samples together, we identified 55,867 unigenes with an N50 length of 2,806 bp (Table S6). For the annotation, 32,492, 17,867, 26,930, 26,289, 15,633, 26,541, and 11,578 unigenes were annotated to Nr, Nt, SwissProt, InterPro, KEGG, COG, and GO databases, respectively, which covered 35,013 (62.67%) of the total unigenes (Table S7).

Gene Ontology (GO) annotation analysis was used to categorize these unigenes into different categories. In the molecular function category, the genes associated with binding, catalytic, and transporter activities were the most abundant groups. In the biological process category, most genes were involved in cellular process, metabolic process, and single-organism process. Cell, cell part, and membrane were the most prevalent in the cellular component category (Figure S1).

### Identification and analysis of OBP genes

A total of 46 putative OBP genes (*BodoOBP1-46*) were identified in the antennae and body transcriptome of adult *B. odoriphaga* (Table 1). Moreover, we also discovered three other putative OBP genes (*BodoOBP47-49*) from the fourth instar larval transcriptome of *B. odoriphaga* (unpublished data). Forty-eight of the 49 OBP genes (except for *BodoOBP32*) have intact open reading frames (ORFs) with lengths ranging from 378 to 759 bp (Table 1). Nearly all full-length OBPs had a predicted signal peptide (a signature of secretory proteins) at the N-terminal region except for BodoOBP22/25. All 49 OBPs had the predicted domains of pheromone/general odorant binding protein (PhBP or PBP_GOBP) (InterPro: IPR006170) (Table S8). Based on the number and location of the conserved cysteines, all BodoOBPs could be divided into the following three groups: Minus-C OBPs group (BodoOBP14/23/26/31/33/41/42/43/44), Plus-C OBPs group (BodoOBP19/34), and the remaining OBPs belong to Classic OBPs group (Figure S2).

**Table 1:** List of OBP genes in *Bradysia odoriphaga*.

**Gene name**	**Accession number**	**ORF (bp)**	**Amino acid length (AA)**	**Signal peptide (AA)**	**Pfam**	**BLASTx annotation**	**Score**	***E*-value**	**Identity (%)**
*BodoOBP1*	MG544121	453	150	1–17	PF01395	gb|ANA52575.1|odorant binding protein 1 (*Bradysia odoriphaga*)	313	2e-108	100
*BodoOBP2*	MG544122	453	150	1–17	PF01395	gb|ANA52576.1|odorant binding protein 2 (*Bradysia odoriphaga*)	313	2e-108	100
*BodoOBP3*	MG544123	423	140	1–19	PF01395	gb|AHW83258.1|odorant binding protein OBP13, partial (*Sitodiplosis mosellana*)	79.7	1e-16	42
*BodoOBP4*	MG544124	507	168	1–23	PF01395	ref|XP_020713726.1|pheromone-binding protein-related protein 6 (*Ceratitis capitata*)	66.6	9e-11	27
*BodoOBP5*	MG544125	438	145	1–25	PF01395	ref|XP_001647921.1|general odorant-binding protein 72 (*Aedes aegypti*)	116	1e-30	36
*BodoOBP6*	MG544126	399	132	1–20	PF01395	ref|XP_001863133.1|odorant-binding protein (*Culex quinquefasciatus*)	102	2e-25	43
*BodoOBP7*	MG544127	444	147	1–22	PF01395	ref|NP_001035316.1|odorant binding protein 11 precursor (*Apis mellifera*)	47.8	5e-04	30
*BodoOBP8*	MG544128	435	144	1–17	PF01395	ref|XP_001999215.1|odorant-binding protein 83abL1 (*Drosophila mojavensis*)	148	4e-43	57
*BodoOBP9*	MG544129	447	148	1–18	PF01395	ref|XP_001867253.1|odorant-binding protein 56e (*Culex quinquefasciatus*)	66.6	4e-11	32
*BodoOBP10*	MG544130	435	144	1–19	PF01395	gb|ACR43440.1|odorant-binding protein 12 (*Culex quinquefasciatus*)	126	1e-34	42
*BodoOBP11*	MG544131	426	141	1–20	PF01395	gb|ACR43440.1|odorant-binding protein 12 (*Culex quinquefasciatus*)	147	1e-42	49
*BodoOBP12*	MG544132	432	143	1–19	PF01395	gb|AKI28998.1|odorant binding protein 19a (*Bactrocera dorsalis*)	120	6e-32	43
*BodoOBP13*	MG544133	447	148	1–24	PF01395	gb|AAL84183.1|odorant binding protein (*Anopheles gambiae*)	128	6e-35	47
*BodoOBP14*	MG544134	432	143	1–16	PF01395	ref|XP_002005074.2|odorant-binding protein 44a (*Drosophila mojavensis*)	149	6e-43	49
*BodoOBP15*	MG544135	450	149	1–24	PF01395	ref|XP_017468801.1|general odorant-binding protein 19d-like (*Rhagoletis zephyria*)	67	3e-11	34
*BodoOBP16*	MG544136	435	144	1–18	PF01395	ref|XP_008200270.1|general odorant-binding protein 19d (*Tribolium castaneum*)	85.5	2e-18	36
*BodoOBP17*	MG544137	384	127	1–18	PF01395	gb|AOW41523.1|odorant-binding protein OBP56d-2, partial (*Anastrepha oblique*)	62.8	6e-10	38
*BodoOBP18*	MG544138	417	138	1–20	PF01395	gb|AHW83249.1|odorant binding protein OBP21d (*Sitodiplosis mosellana*)	123	2e-33	45
*BodoOBP19*	MG544139	759	252	1–17	PF01395	gb|AKI29006.1|odorant binding protein 50c (*Bactrocera dorsalis*)	135	5e-35	36
*BodoOBP20*	MG544140	549	182	1–19	PF01395	gb|ETN61506.1|odorant binding protein, antennal (*Anopheles darling*)	52.4	3e-05	29
*BodoOBP21*	MG544141	435	144	1–22	PF01395	ref|XP_002064402.2|odorant-binding protein 19d (*Drosophila willistoni*)	68.6	1e-11	31
*BodoOBP22*	MG544142	444	147	ND	PF01395	gb|AAL84183.1|odorant binding protein (*Anopheles gambiae*)	167	1e-50	57
*BodoOBP23*	MG544143	426	141	1–16	PF01395	gb|ASM41500.1|ordorant binding protein 8 (*Bactrocera minax*)	107	3e-27	42
*BodoOBP24*	MG544144	435	144	1–18	PF01395	gb|AHW83258.1|odorant binding protein OBP13, partial (*Sitodiplosis mosellana)*	77	2e-15	40
*BodoOBP25*	MG544145	447	148	ND	PF01395	gb|AAL84183.1|odorant binding protein (*Anopheles gambiae*)	167	2e-50	59
*BodoOBP26*	MG544146	462	153	1–18	PF01395	—	—	—	—
*BodoOBP27*	MG544147	465	154	1–19	PF01395	gb|KNC21649.1|general odorant-binding protein 56a (*Lucilia cuprina*)	43.5	6e-2	25
*BodoOBP28*	MG544148	438	145	1–18	PF01395	gb|ETN61420.1|odorant binding protein (*Anopheles darling*)	194	2e-61	65
*BodoOBP29*	MG544149	720	239	1–18	PF01395	gb|AGI37367.1|pheromone binding protein 3 (*Cnaphalocrocis medinalis*)	39.3	3.2	35
*BodoOBP30*	MG544150	516	171	1–21	PF01395	gb|ETN60853.1|odorant binding protein (*Anopheles darling*)	45.4	6e-3	36
*BodoOBP31*	MG544151	426	141	1–15	PF01395	ref|XP_001999222.1|odorant-binding protein 83g (*Drosophila mojavensis*)	46.2	2e-3	28
*BodoOBP32*	MG544152	5'missing	>223	ND	PF01395	ref|XP_004525139.2|general odorant-binding protein 19d (*Ceratitis capitata*)	39.7	1.6	24
*BodoOBP33*	MG544153	459	152	1–17	PF01395	—	—	—	—
*BodoOBP34*	MG544154	570	189	1–17	PF01395	ref|XP_001843379.1|odorant-binding protein 50d (*Culex quinquefasciatus*)	102	1e-23	33
*BodoOBP35*	MG544155	450	149	1–24	PF01395	gb|AHW83245.1|odorant binding protein OBP14 (*Sitodiplosis mosellana)*	91.7	1e-20	35
*BodoOBP36*	MG544156	720	239	1–20	PF01395	ref|XP_004525035.1|general odorant-binding protein 19d (*Ceratitis capitata*)	37.7	7.2	30
*BodoOBP37*	MG544157	399	132	1–19	PF01395	ref|XP_005188786.1|general odorant-binding protein 28a (*Musca domestica)*	48.9	2e-04	30
*BodoOBP38*	MG544158	543	180	1–22	PF01395	—	—	—	—
*BodoOBP39*	MG544159	414	137	1–19	PF01395	gb|ETN60853.1|odorant binding protein (*Anopheles darling*)	67.4	2e-11	29
*BodoOBP40*	MG544160	429	142	1–24	PF01395	gb|AHW83245.1|odorant binding protein OBP14 (*Sitodiplosis mosellana*)	72.8	2e-13	35
*BodoOBP41*	MG544161	426	141	1–18	PF01395	ref|XP_004536902.1|general odorant-binding protein 99a-like (*Ceratitis capitata*)	59.7	1e-08	32
*BodoOBP42*	MG544162	435	144	1–18	PF01395	ref|XP_017087731.1|general odorant-binding protein 99a (*Drosophila bipectinata*)	75.5	1e-14	35
*BodoOBP43*	MG544163	417	138	1–16	PF01395	ref|XP_022223959.1|general odorant-binding protein 99a (*Drosophila obscura*)	77	3e-15	33
*BodoOBP44*	MG544164	396	131	1–17	PF01395	ref|XP_001651445.1|general odorant-binding protein 99a (*Aedes aegypti*)	49.7	8e-05	34
*BodoOBP45*	MG544165	423	140	1–19	PF01395	gb|AHW83258.1|odorant binding protein OBP13, partial (*Sitodiplosis mosellana*)	76.3	3e-15	40
*BodoOBP46*	MG544166	417	138	1–20	PF01395	gb|AHW83249.1|odorant binding protein OBP21d (*Sitodiplosis mosellana)*	123	2e-33	44
*BodoOBP47*	MG544167	378	125	1–18	PF01395	ref|XP_002049119.1|odorant-binding protein 56a (*Drosophila virilis*)	59.3	1e-08	33
*BodoOBP48*	MG544168	450	149	1–21	PF01395	gb|AHW83245.1|odorant binding protein OBP14 (*Sitodiplosis mosellana*)	75.1	3e-14	35
*BodoOBP49*	MG544169	435	144	1–21	PF01395	gb|AMD02857.1|odorant binding protein 17, partial (*Adelphocoris lineolatus)*	63.5	3e-10	33

*ND: Not detected; “—”: No significant similarity annotation found, these genes encode proteins that have a conserved domain (PhBP or PBP_GOBP) predicted by SMART (Table S8)*.

**Figure 1:**
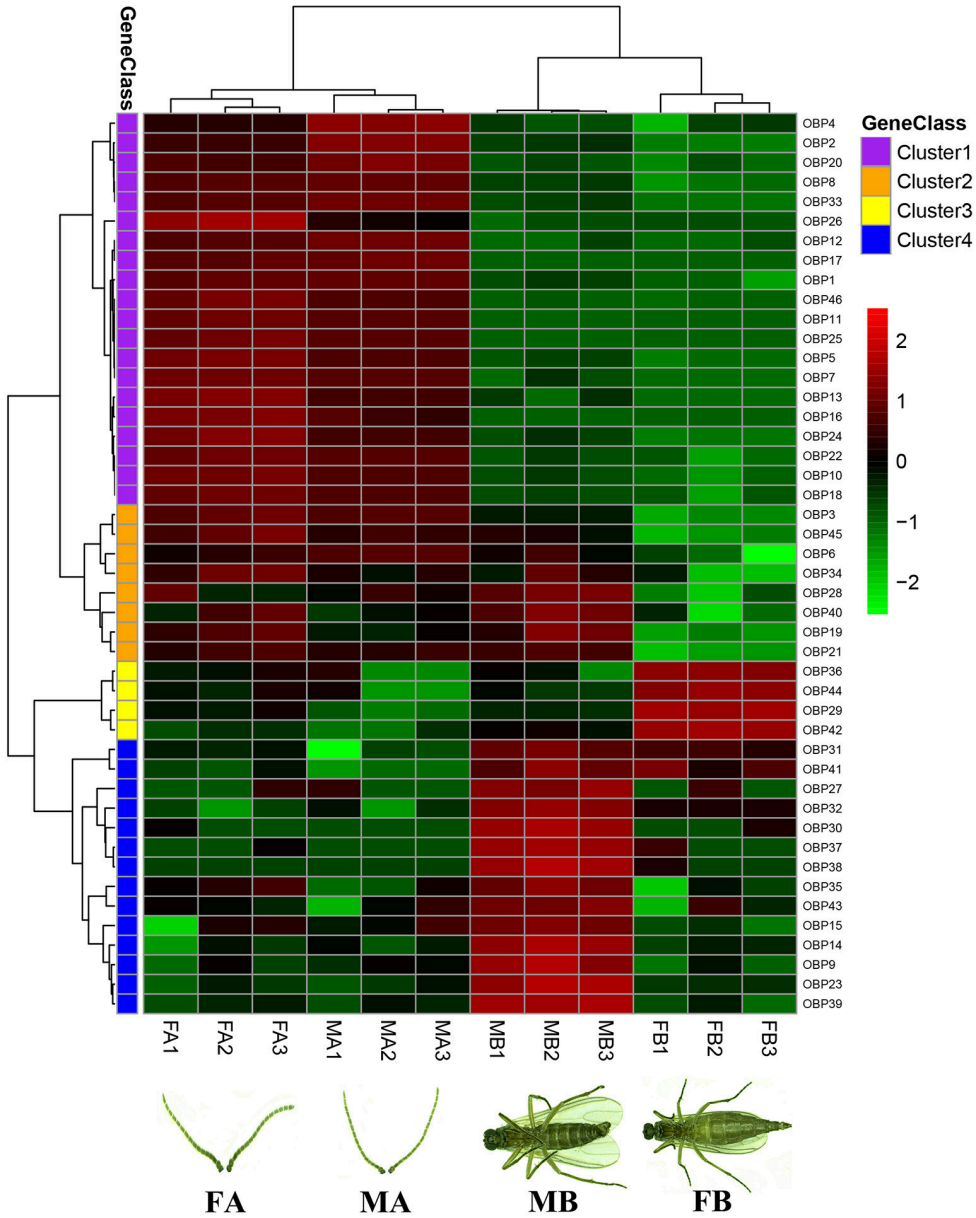
Tissue- and sex-specific expression profiles of OBP genes in antennae and body transcriptomes in *B. odoriphaga*. FA, female antennae; MA, male antennae; MB, male body; FB, female body. The FPKM-values were used for calculating transcript abundance. These 46 OBP genes identified from antennae and body transcriptomes were clustered into four classes (Cluster 1–4). Cluster 1 represents the OBPs mainly expressed in the FA and MA. OBPs in Cluster 2 were relatively highly expressed in not only the FA and MA but also the MB. Genes in Clusters 3 and 4 were more highly expressed in the FB and MB, respectively. Three biological replicates were conducted for each treatment (such as FA1, FA2, and FA3).

Gene expression levels of all 46 OBPs identified from antennae and body transcriptomes were assessed using FPKM-values, represented in a heatmap (Figure 1). The three repetitions of each tissue (FA, MA, FB, and MB) were clustered together, indicating that the results are stable and repeatable. Based on the expression levels in different tissues, all 46 OBP genes were clustered into 4 groups. Cluster analysis revealed that 20 OBP genes (Cluster 1) have similar expression patterns and were relatively high in the female and male antennae (FA and MA). Four and fourteen OBPs were more highly expressed in the FB (Cluster 3) and MB (Cluster 4), respectively. Moreover, the remaining eight OBPs were relatively highly expressed in not only the FA and MA but also the MB (Cluster 2) (Figure 1).

### Identification and analysis of CSP genes

We have identified five putative CSP genes (*BodoCSP1-5*) from the antennae, body and larval transcriptome of *B. odoriphaga*. All the CSP genes have intact ORFs with lengths ranging from 327 to 708 bp, and with predicted signal peptide sequences at the N-terminus (Table 2). All BodoCSPs had typical structural features of insect CSPs with four conserved cysteines (Figure S3) and a conserved OS-D domain (olfactory system of *D. melanogaster*) (InterPro: IPR005055) (Table S9).

**Table 2:** List of CSP genes in *Bradysia odoriphaga*.

**Gene name**	**Accession number**	**ORF (bp)**	**Amino acid length (AA)**	**Signal peptide (AA)**	**Pfam**	**FPKM-value**				**BLASTx annotation**	**Score**	***E*-value**	**Identity (%)**
						**FA**	**MA**	**FB**	**MB**				
*BodoCSP1*	MG544170	390	129	1–18	PF03392	693	991	106	1508	gb|ANA52574.1|chemosensory protein (*Bradysia odoriphaga*)	236	3e-78	100
*BodoCSP2*	MG544171	327	108	1–25	PF03392	3	2	0	7	gb|AID61323.1|chemosensory protein, partial (*Calliphora stygia*)	160	3e-49	76
*BodoCSP3*	MG544172	426	141	1–25	PF03392	1077	1029	0	0	gb|BAV56812.1|chemosensory protein 8 (*Ostrinia furnacalis*)	122	7e-33	54
*BodoCSP4*	MG544173	357	118	1–18	PF03392	7	14	4	7	gb|CAG26923.1|putative chemosensory protein CSP1 (*Anopheles gambiae*)	136	3e-39	50
*BodoCSP5*	MG544174	708	235	1–20	PF03392	36	23	0	0	gb|AJP61958.1|chemosensory protein (*Phenacoccus solenopsis*)	107	7e-26	54

*FA, female antennae; MA, male antennae; FB, female body; MB, male body*.

Gene expression levels of all five CSPs in different tissues were assessed by FPKM-values. *BodoCSP3* and *BodoCSP5* were significantly higher expressed in the female and male antennae (FA and MA), *BodoCSP1* and *BodoCSP2* were relatively highly expressed in the MB, and *BodoCSP4* exhibited similar expression levels in different tissues (Table 2).

### Phylogenetic analysis of *B. odoriphaga* OBP and CSP genes

**Figure 2:**
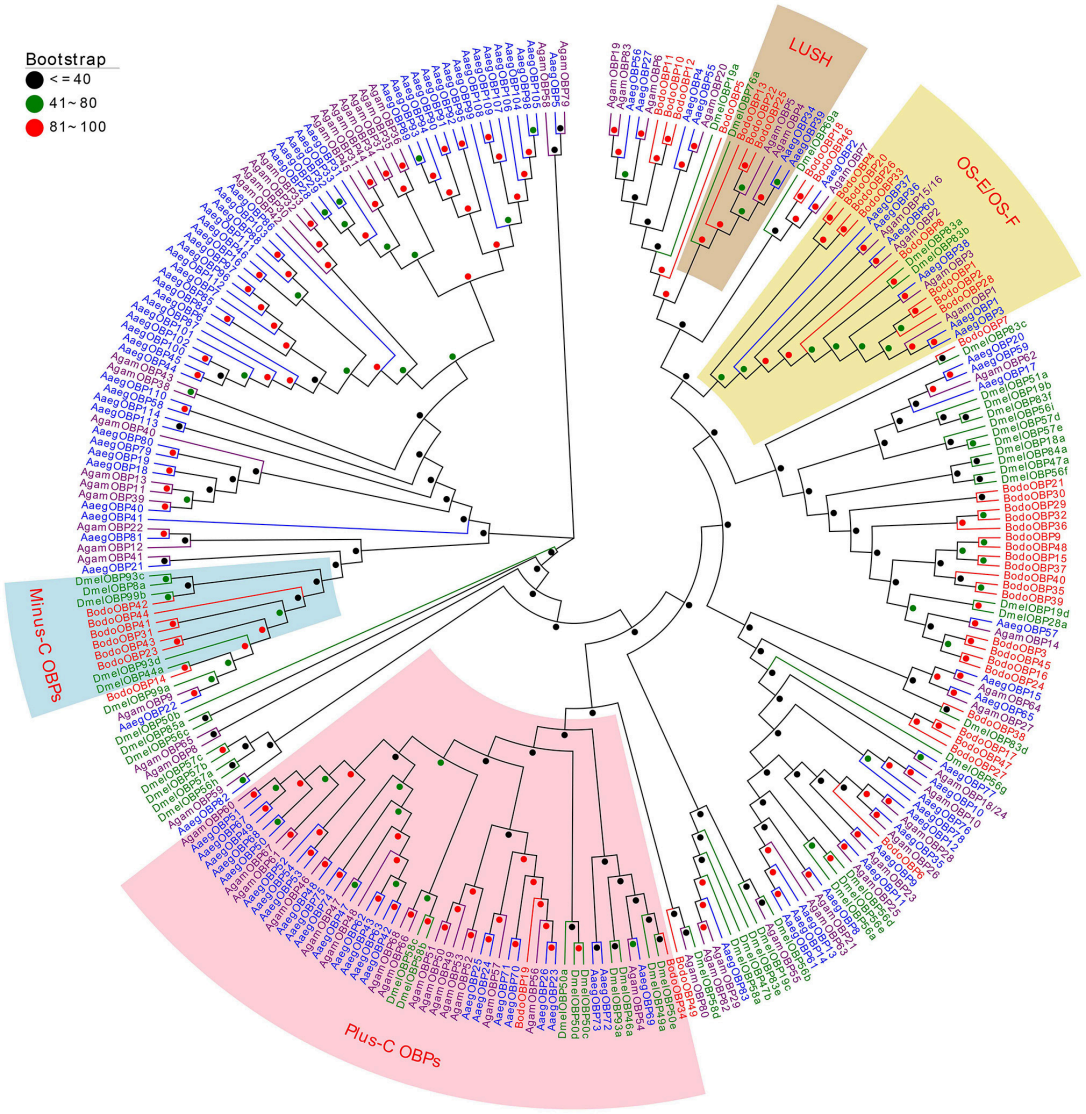
Neighbor-joining tree of 280 OBP proteins from Diptera species. The protein names and sequences of the 280 OBPs that were used in this analysis are listed in Table S2. Bootstrap values are shown. The Plus-C OBPs clade, Minus-C OBPs clade, LUSH clade, and OS-E/OS-F clade are shown. The Diptera species used to construct this tree include *B. odoriphaga* (Bodo, red), *D. melanogaster* (Dmel, green), *A. gambiae* (Agam, purple), and *A. aegypti* (Aaeg, blue).

A phylogenetic tree of 280 OBPs from 4 Diptera species (*B. odoriphaga, D. melanogaster, A. gambiae*, and *A. aegypti*) was constructed using the protein sequences to reveal the diverging relationships among them (Figure 2). Some pairs of BodoOBPs are paralogous genes, such as BodoOBP26/33, BodoOBP4/20, BodoOBP1/2, BodoOBP18/46, BodoOBP22/25, BodoOBP10/12, BodoOBP3/45, BodoOBP16/24, BodoOBP17/47, BodoOBP23/43, and BodoOBP31/41. All of these paralogous genes showed very high bootstrap values, which may indicate that these genes are the result of a recent gene duplication event within the *B. odoriphaga* genome. Moreover, 2 putative Plus-C OBPs (BodoOBP19 and 34) were clustered into the Plus-C OBP group with the 50 Plus-C OBPs from the other Diptera insect, and 7 putative Minus-C OBPs (BodoOBP14/23/31/41/42/43/44) were clustered into the Minus-C OBP group with 5 Minus-C OBPs from *D. melanogaster*, suggesting their different evolutionary relationships compared to the classic OBPs (Figure 2). In addition, BodoOBP13/22/25 were clustered with the DmelOBP76a (LUSH, an OBP with binding affinity to the pheromone), and BodoOBP1/2/4/8/20/26/28/33 were clustered with DmelOBP83a/83b (OS-E/OS-F, an OBP group co-expressed with LUSH and associated with pheromone detection) (Figure 2), indicating that they might have a similar function in the detection of candidate pheromones in *B. odoriphaga*.

**Figure 3:**
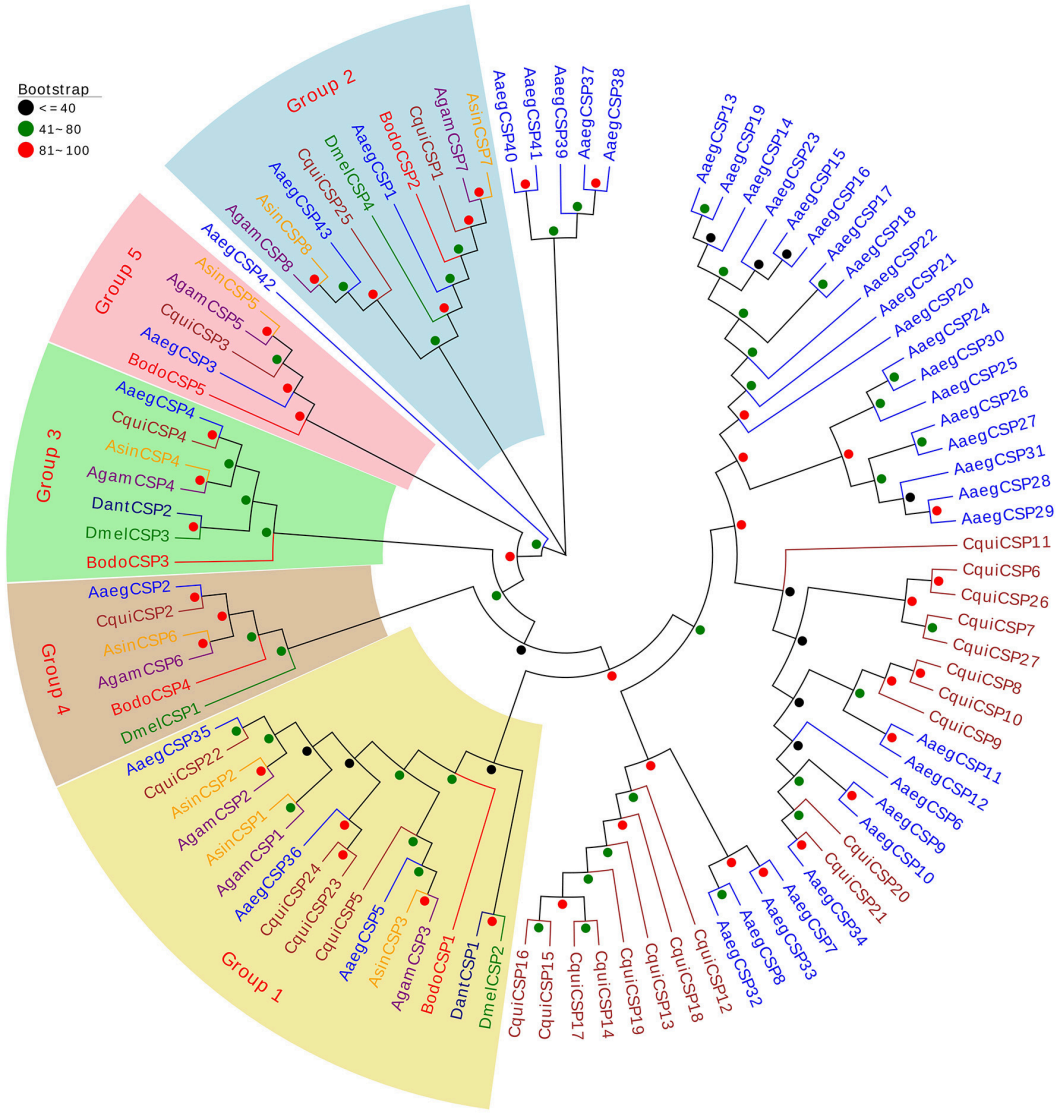
Neighbor-joining tree of 97 CSP proteins from Diptera species. The protein names and sequences of the 97 CSPs that were used in this analysis are listed in Table S3. Bootstrap values are shown. Five BodoCSPs were scattered into five subgroups (Groups 1–5), where each group includes one BodoCSP. The Diptera species used to construct this tree include *B. odoriphaga* (Bodo, red), *D. melanogaster* (Dmel, green), *A. gambiae* (Agam, purple), *A. aegypti* (Aaeg, blue), *A. sinensis* (Asin, orange), *C. quinquefasciatus* (Cqui, brown), and *D. antiqua* (Dant, navy).

The neighbor-joining tree of CSPs was conducted using 5 putative BodoCSPs and 92 CSPs from 6 other Diptera species (*D. melanogaster, A. gambiae, A. sinensis, A. aegypti, C. quinquefasciatus*, and *D. antiqua*) (Figure 3). Five putative BodoCSPs were scattered into five subgroups (Groups 1–5), where each group included one BodoCSP. Moreover, four DmelCSPs were scattered into four subgroups (Groups 1–4), with one DmelCSP in each group (Figure 3). Almost every group included one or more CSPs from each Dipteran species, suggesting that the CSP gene has been highly conserved among different Dipteran insects.

### Motif pattern analysis of OBPs and CSPs

**Figure 4:**
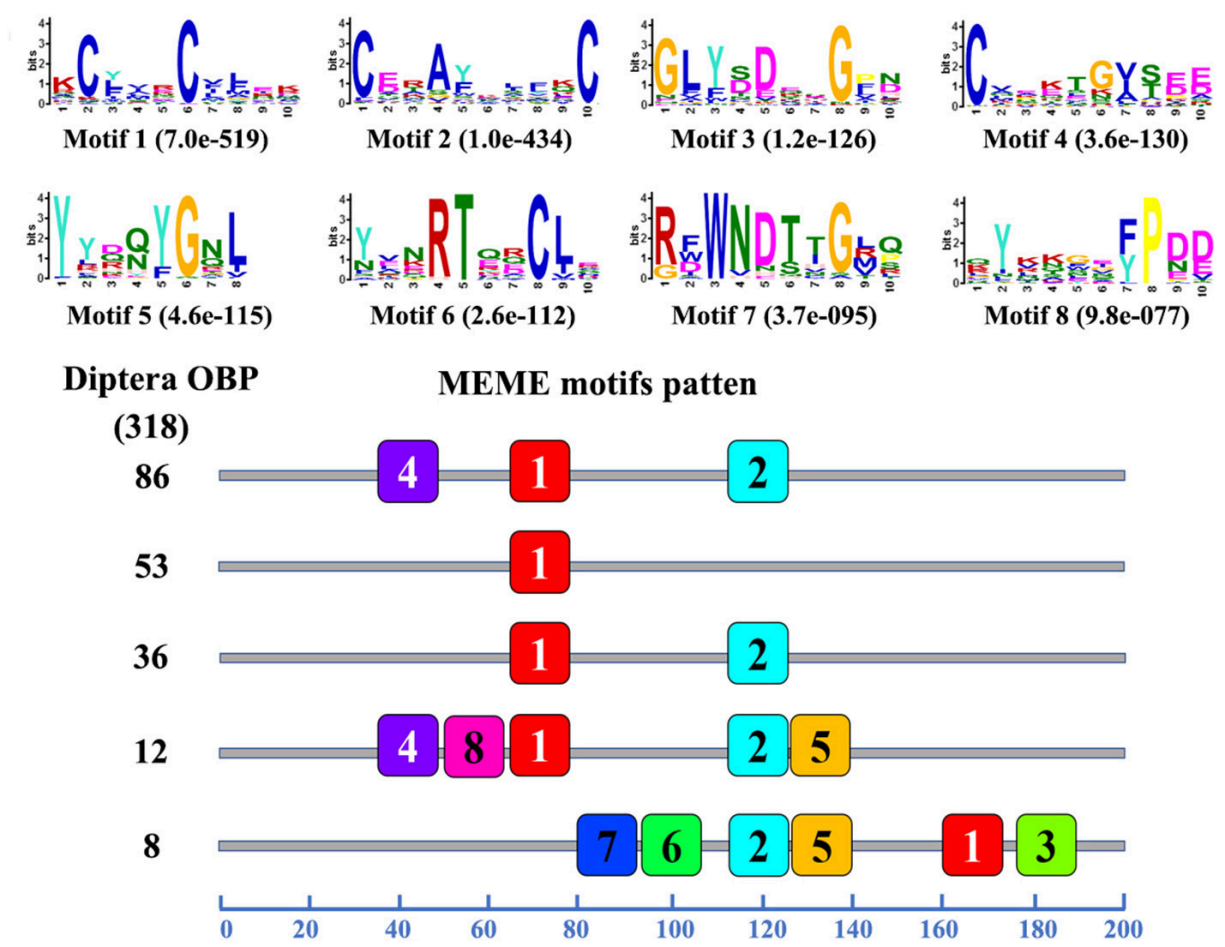
Motif analysis of Diptera OBPs. Parameters used for motif discovery were as follows: minimum width = 6, maximum width = 10, maximum number of motif to find = 8. The upper parts list the eight motifs discovered in the Diptera OBPs. The numbers in the boxes correspond to the numbered motifs in the upper part of the figure, where a small number indicates high conservation. The numbers on the bottom show the approximate locations of each motif on the protein sequence, starting from the N-terminus. The protein names and sequences of the 318 OBPs from different Diptera species are listed in Table S4.

The motif pattern analysis results showed that 68 different motif patterns were observed in the 318 OBPs, and 195 OBPs (61.32%) had the most common five motif-patterns. Eighty-six of them had the most common motif-pattern 4-1-2, fifty-three OBPs only had motif 1, and thirty-six OBPs had the motif-pattern 1-2 (Figure 4). The motif pattern analysis results of 138 CSPs of Diptera insects showed that 8 different motif patterns were found, suggesting that CSPs were more conserved than the OBPs. In the 8 different motif patterns, 123 CSPs (89.13%) had the most common three motif patterns: 93 CSPs had motif pattern 8-5-6-1-3-2-4-7, 16 CSPs had motif pattern 6-1-3-2-4, and 14 CSPs had motif pattern 5-6-1-3-2-4-7 (Figure S4). The remaining 15 CSPs shared the 5 other different motif patterns.

### Transcript expression levels of *B. odoriphaga* OBPs

**Figure 5:**
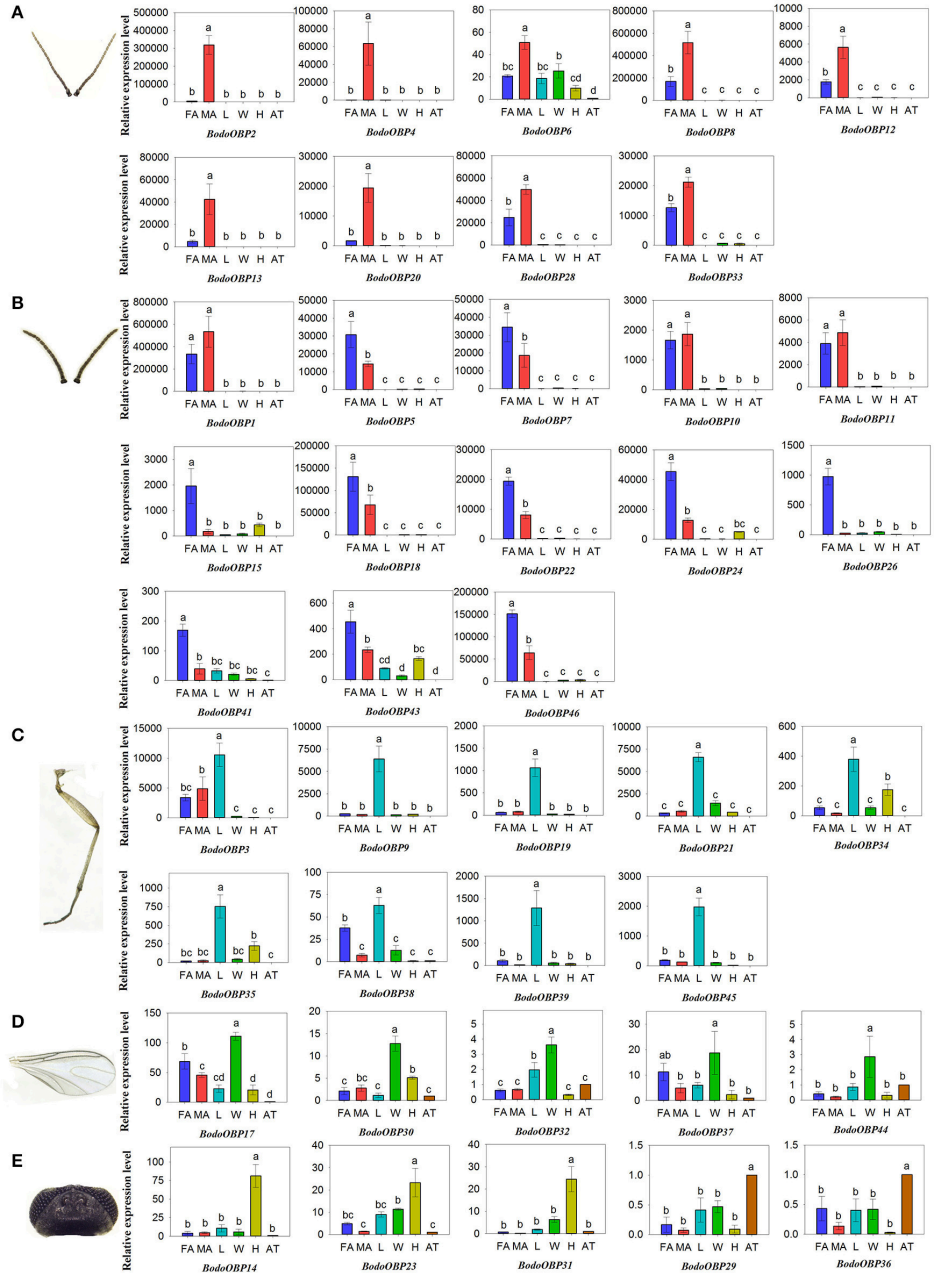
Transcript levels of tissue-specific OBP genes in different tissues of *B. odoriphaga*. FA, female antennae; MA, male antennae; L, leg; W, wing; H, head (without antennae); AT, abdomen and thorax. **(A)** MA-specific, **(B)** antennae-specific, **(C)** L-specific, **(D)** W-specific, **(E)** H- and AT-specific. Two reference genes, RPS15 (ribosomal protein S15) and RPL18 (ribosomal protein L18) were used for normalizing OBP gene expression and to correct for sample-to-sample variation. Transcript levels were normalized to those of AT. The standard error is represented by the error bar, and the different lower cases above each bar indicate significant differences (*P* < 0.05).

**Figure 6:**
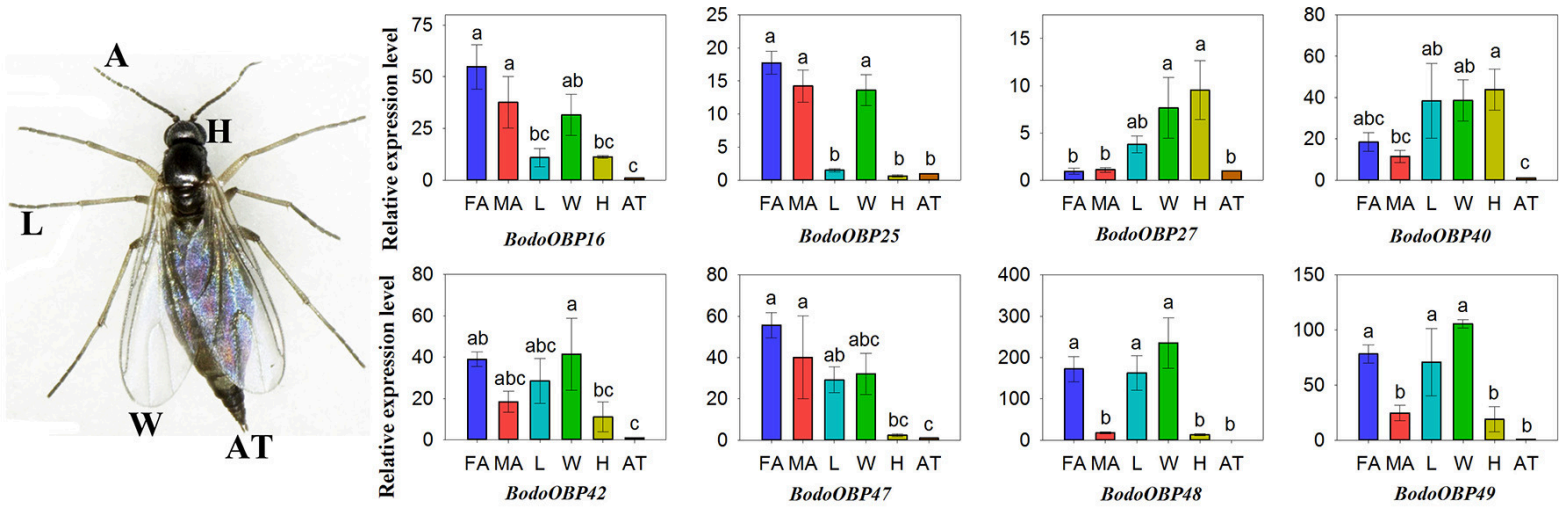
Transcript levels of non-tissue-specific OBP genes in different tissues of *B. odoriphaga*. FA, female antennae; MA, male antennae; L, leg; W, wing; H, head (without antennae); AT, abdomen and thorax. Two reference genes, RPS15 (ribosomal protein S15) and RPL18 (ribosomal protein L18), were used for normalizing OBP genes expression and to correct for sample-to-sample variation. Transcript levels were normalized to those of AT. The standard error is represented by the error bar, and the different lower cases above each bar indicate significant differences (*P* < 0.05).

The transcript expression levels of 49 BodoOBP genes in female antennae (FA), male antennae (MA), legs (L), wings (W), heads (without antennae, H), and abdomens and thoraxes (AT) were analyzed by qRT-PCR. The results suggested that 22 OBP genes (*BodoOBP1*/*2*/*4*/*5*/*6*/*7*/*8*/*10*/*11*/*12*/*13*/*15*/*18*/*20*/*22*/*24*/*26*/*28*/*33*/*41*/*43*/*46*) were significantly higher expressed in the antennae (FA or MA) (Figures 5A,B), and 9 of the 22 antennae-biased OBP genes (*BodoOBP2/4*/*6*/*8*/*12*/*13*/*20*/*28*/*33*) were predominantly expressed in the male antennae (MA) (Figure 5A). Moreover, nine BodoOBP genes (*BodoOBP3*/*9*/*19*/*21*/*34*/*35*/*38*/*39*/*45*) were intensively expressed in the legs (L) than in other tissues (Figure 5C), whereas five BodoOBP genes (*BodoOBP17*/*30*/*19*/*21*/*34*) were mainly detected in the wings (W) (Figure 5D). Three BodoOBP genes (*BodoOBP14*/*23*/*31*) were significantly higher expressed in the heads (H), and two BodoOBP genes (*BodoOBP29*/*36*) showed higher expression levels in the abdomens and thoraxes (AT) (Figure 5E). In addition, the remaining eight BodoOBP genes (*BodoOBP16*/*25*/*27*/*40*/*42*/*47*/*48*/*49*) were expressed in more than three tissues, or they showed no significant differences among different tissues (Figure 6).

### Transcript expression levels of *B. odoriphaga* CSPs

**Figure 7:**
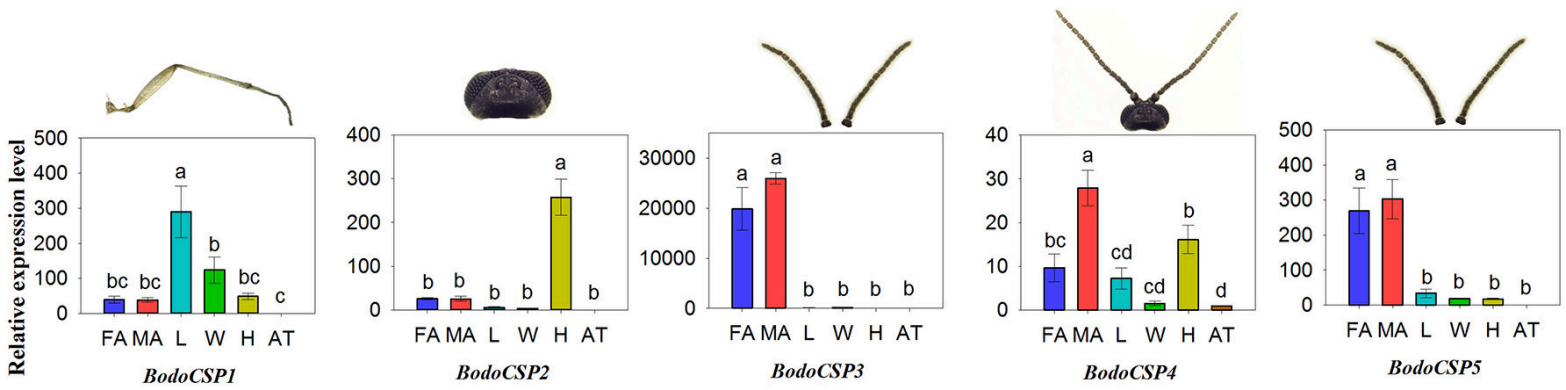
Transcript levels of CSP genes in different tissues of *B. odoriphaga*. FA, female antennae; MA, male antennae; L, leg; W, wing; H, head (without antennae); AT, abdomen and thorax. Two reference genes, RPS15 (ribosomal protein S15) and RPL18 (ribosomal protein L18) were used for normalizing CSP genes expression and to correct for sample-to-sample variation. Transcript levels were normalized to those of AT. The standard error is represented by the error bar, and the different lower cases above each bar indicate significant differences (*P* < 0.05).

The quantitative expression levels of five BodoCSP genes in different tissues were characterized using qRT-PCR. The results showed that *BodoCSP1* had higher expression levels in the legs (L) than in other tissues (Figure 7), *BodoCSP2* was significantly higher expressed in the heads (H), and *BodoCSP3* and *BodoCSP5* were mainly expressed in antennae (FA and MA). Moreover, *BodoCSP4* showed predominantly expression in the male antennae (MA) and higher expression in the female antennae (FA) and heads (H) (Figure 7).

## Discussion

**Figure 8:**
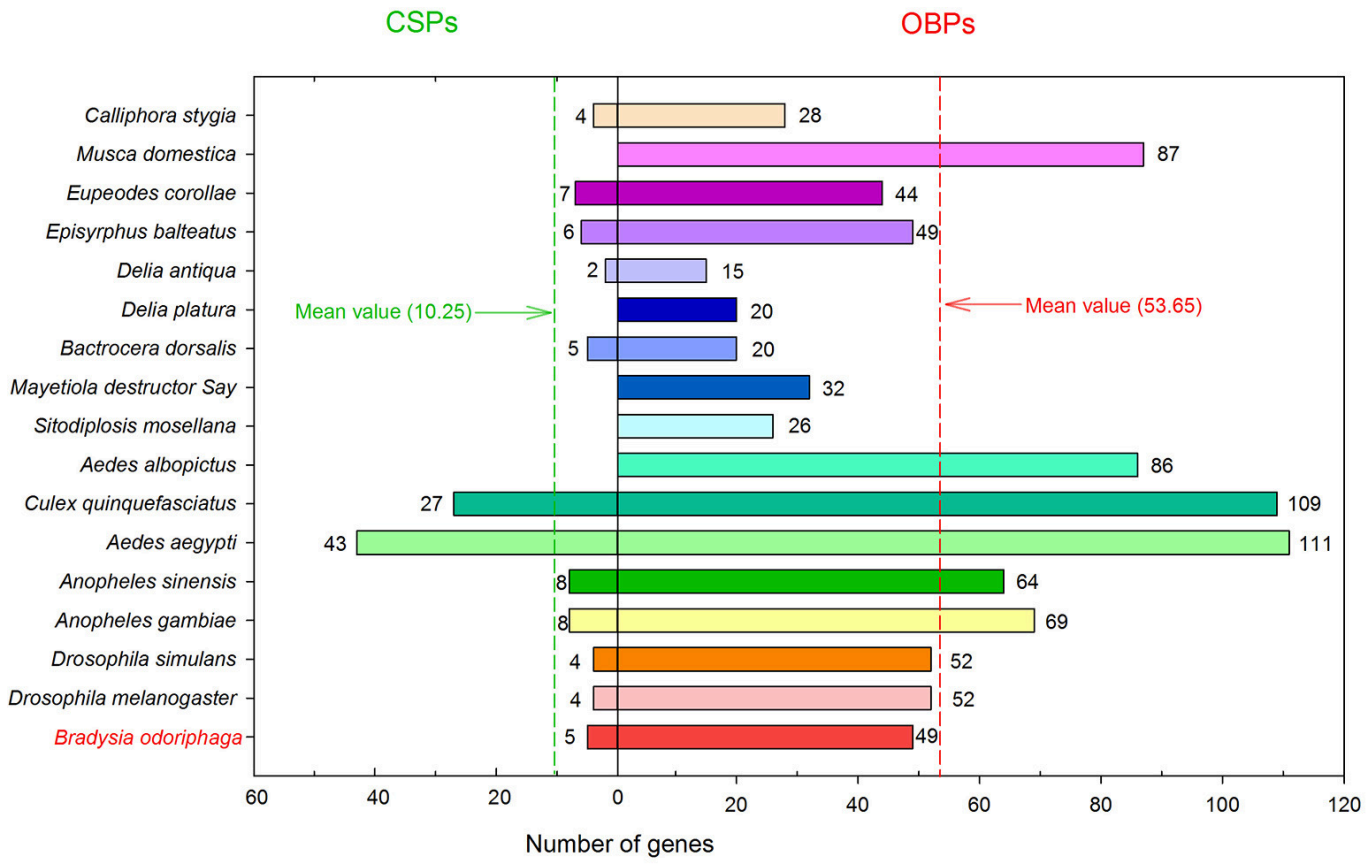
The number of OBP and CSP genes in 17 different Diptera insects. The digits near the histogram bars represent the number of OBP/CSP genes in different insects. The red and green dashed lines represent the mean number of OBP and CSP genes in 17 Diptera insects, respectively.

In the present study, we sequenced and analyzed the transcriptomes of antennae and bodies of adult *B. odoriphaga* (female and male), and searched for OBP and CSP genes from the transcriptomes of adults and larvae (our unpublished data). In total, we identified 49 OBP and 5 CSP genes in *B. odoriphaga*. The number of OBPs in *B. odoriphaga* was similar to the number in *D. melanogaster* (52), *D. simulans* (52), *Episyrphus balteatus* (49), and *Eupeodes corollae* (44) (Vieira and Rozas, 2011; Wang et al., 2017). Meanwhile, the number of OBPs in *B. odoriphaga* was greater than in some other Dipteran agricultural pests. For example, 15 OBPs were found in *Delia antiqua*, 20 in *Delia platura*, 20 in *Bactrocera dorsalis*, 32 in *Mayetiola destructor* Say, and 26 in *Sitodiplosis mosellana* (Andersson et al., 2014; Gong et al., 2014; Ohta et al., 2014, 2015; Liu et al., 2016) (Figure 8). There are likely multiple reasons responsible for identifying so many OBP genes in our study. First, this pest has a wide range of host plants (such as chive, shallot, garlic, cabbage, and mushrooms) (Ma et al., 2013), which might result in an increase in the number of OBP genes for detecting various odor molecules in a complex environment. Second, OBP genes were identified not only from the adult antennae transcriptome but also from the adult body and larval transcriptomes. If we solely identified OBP genes from the antennae transcriptome, the “Cluster 3” and “Cluster 4” genes (18 OBP genes) (Figure 2) and 3 larval transcriptome OBP genes may not have been identified. Additionally, previous studies have shown that the sequencing depth of different sequencing platforms will influence the number of identified OBP genes (Gu et al., 2015; Cui et al., 2017). The FPKM-values of 13 OBP genes were lower than 25 in the antennae and body transcriptomes of *B. odoriphaga*, which suggests that the sequencing depth of the Hiseq 4000 sequencing platform was superior, and this may be another reason for the identification of so many OBP genes in the present study. In addition, we identified five CSP genes in *B. odoriphaga*, and this number is very close to the number of CSP genes in *D. melanogaster* (4), *D. simulans* (4), *B. dorsalis* (5), and *E. balteatus* (6) (Vieira and Rozas, 2011; Liu et al., 2016; Wang et al., 2017). Compared with the OBP genes (mean value: 53.65), only a small number of CSP genes (mean value: 10.25) were detected in 17 species of Diptera insects (Figure 8), which is due to the evolutionary pattern in the CSP gene family and is less dynamic than in the OBP gene family (Vieira and Rozas, 2011). In addition, previous studies demonstrated that the C-patterns of OBPs and CSPs are similar among different insect Orders, whereas the motif-patterns are different (Zhou, 2010; Gu et al., 2015; He et al., 2017). For example, the motif-patterns between Dipteran and Lepidopteran GOBPs are different (Xu et al., 2009). Our present study also found that the motif-patterns among Dipteran OBPs were different, this is because the C-patterns of OBPs determines their crucial conserved structure, and motif-patterns fine-tune their specific functions (Xu et al., 2009).

The tissue expression profiles of chemosensory genes may be indicative of their biological functions, and they contribute to our understanding of the molecular mechanism of insect olfaction (He et al., 2011; Gu et al., 2015; Yuan et al., 2015). Various investigations have suggested that a high percentage of OBP genes are expressed in the antennae of insects, and antennae-enriched OBPs play crucial roles in detecting sex pheromones and host volatile compounds (Gong et al., 2014; Brito et al., 2016). In the current study, 22 of 49 *BodoOBPs* were uniquely or primarily expressed in the antennae compared to other tissues (Figures 5A,B). Among the 22 antennae-enriched OBPs, 9 were specifically expressed in male antennae (*BodoOBP2/4/6/8/12/13/20/28/33*) and might have potential functions in sex pheromone detection. Moreover, a phylogenetic analysis of OBPs suggested that BodoOBP13 clustered with the 11-*cis*-vaccenyl acetate binding PBP DmelOBP76a (LUSH) (Ha and Smith, 2006), and BodoOBP2/4/8/20/28/33 clustered together with DmelOBP83a/83b, an OBP group associated with the detection of volatile pheromones in *D. melanogaster* (Shanbhag et al., 2001a; Siciliano et al., 2014) (Figure 2). Hence, our results suggest that these proteins (BodoOBP2/4/8/13/20/28/33) may be involved in the detection of sex pheromones in *B. odoriphaga*. In addition, 13 other OBPs that were highly expressed in the antennae (BodoOBP1/5/7/10/11/15/18/22/24/26/41/43/46) might be associated with functions in general host odorant perception.

Although the majority of OBPs are specifically expressed in antennae, it has become clear that many OBPs are enriched in non-antennal tissues and play key roles in olfactory or gustatory perception (Yasukawa et al., 2010; Sparks et al., 2014; Sun et al., 2017). For instance, two OBP genes (OBP57d and OBP57e) in Drosophila species were co-expressed in the taste sensilla of the leg, and these contribute to the perception of octanoic acid and the location of host plants (Yasukawa et al., 2010). Previously it was demonstrated that *AlinOBP11* is predominately expressed in adult legs of *Adelphocoris lineolatus* and has a crucial role for detection of non-volatile secondary metabolites of host plants (Sun et al., 2016, 2017). In the present study, qRT-PCR results show that nine *BodoOBPs* (*BodoOBP3/9/19/21/34/35/38/39/45*) were significantly higher expressed in the legs (Figure 5C), and the transcript abundance (FPKM-value) of these genes in transcriptomes suggested that four of nine leg-specific OBPs (*BodoOBP9*/35/38/39) were male body (MB) enriched (Figure 1), implying that these four OBPs might also function in the recognition of sex pheromone compounds. The remaining five leg-specific OBPs may probably have a function to bind host plant volatile or non-volatile compounds. Previous studies have suggested that OBPs were also more highly expressed in gustatory organs, such as the heads and wings (Galindo and Smith, 2001; Shanbhag et al., 2001b; Jeong et al., 2013). In the present study, five OBP genes (*BodoOBP17/30/32/37/44*) were abundantly expressed in the wings, and three OBP genes (*BodoOBP14/23/31*) were enriched in the heads, suggesting that these genes might also participate in taste functions (Amrein and Thorne, 2005). In addition, two OBP genes (*BodoOBP29*/*36*) were significantly more highly expressed in the abdomens and thoraxes (AT), and heatmap results show that *BodoOBP29*/*36* were specifically expressed in the female body (FB), indicating that these two genes might be involved in the synthesis and release of sex pheromones, or in the detection of egg-laying substrates (Zheng et al., 2013; Yuan et al., 2015).

CSPs belong to another type of small soluble proteins identified in multiple insect species (Brito et al., 2016; Pelosi et al., 2018). Compared with OBPs, CSPs are more conserved, often exhibiting 40–50% identical amino acid residues between orthologs from different species (Pelosi et al., 2006, 2018). In the present study, the results of MEME motif analysis showed that 123 CSPs (89.13%) had the three most common motif-patterns, whereas this number was only 55.03% in the OBPs. Moreover, the CSP-gene phylogeny suggested that most CSPs were scattered into five subgroups. Nearly every group included one or more CSPs from each Diptera species, which also suggests that CSPs are highly conserved among different Diptera insects. In olfactory perception, CSPs have similar functions to OBP. The hydrophobic pocket of CSPs can also recognize and transport chemical signals to chemoreceptors (Sun et al., 2014; Wang et al., 2016). Our results show that *BodoCSP3/5* were antennae-enriched and might be involved in the chemosensory process. Moreover, previous studies have demonstrated that CSPs are not only associated with chemoreception but also participate in multiple physiological processes, such as limb regeneration of cockroaches, embryo maturation of honeybees, and larvae ecdysis of fire ants (Kitabayashi et al., 1998; Maleszka et al., 2007; Cheng et al., 2015; Pelosi et al., 2018). *BodoCSP1* and *BodoCSP2* were significantly more highly expressed in the legs and heads, respectively, and *BodoCSP4* was more highly expressed in both the antennae and heads. We speculate that these CSPs might have other crucial physiological functions and require further functional verification.

In conclusion, we identified 49 putative OBP and 5 putative CSP genes in the adult (antennae and body) and larval transcriptomes of *B. odoriphaga*, and further tissue expression profiles and phylogenetic tree analyses indicated that some of these genes were antennae- or non-antennae-enriched and may play crucial roles in identifying hosts, locating mates and oviposition sites, avoiding natural enemies, and other important physiological processes. Based on the results of this work, future research will focus on the binding function of antennae-enriched OBPs with identified sex pheromones and host volatile components. The results of the present study provide a starting point to facilitate functional studies of these chemosensory genes in *B. odoriphaga* at the molecular level.

## Author contributions

YZ, CZ, and WM designed the experiments. YZ, JD, and ZZ carried out the experiments. YZ, ZZ, FL, and WM analyzed the data. YZ, ZZ, FL, and WM drafted the manuscript. All authors approved the final version of the manuscript.

### Conflict of interest statement

The authors declare that the research was conducted in the absence of any commercial or financial relationships that could be construed as a potential conflict of interest.
