# Supplementary material for: Sex- and Tissue-Specific Expression Profiles of Odorant Binding Protein and Chemosensory Protein Genes in Bradysia odoriphaga (Diptera: Sciaridae)
Source: Front Physiol. 2018 Apr 3;9:107. doi: 10.3389/fphys.2018.00107 (PMC5891581; doi:10.3389/fphys.2018.00107)
Supplement: Supplementary file 1 [file Table1.docx]

**SUPPLEMENTARY MATERIAL**

**Sex- and tissue-specific expression profiles of odorant binding protein and chemosensory protein genes in *Bradysia odoriphaga* (Diptera: Sciaridae)**

**Authors:** Yunhe Zhao ^a^, Jinfeng Ding ^a^, Zhengqun Zhang ^b^, Feng Liu ^a^, Chenggang Zhou ^c^, Wei Mu ^a^ *.

^a^ *College of Plant Protection,* *Shandong Provincial Key Laboratory for Biology of Vegetable Diseases and Insect Pests, Shandong Agricultural University, Tai’an, Shandong 271018, P.R. China*

^b^ *College of Horticultural Science and Engineering, Shandong Agricultural University, Tai'an, Shandong 271018, PR China*

^c^ *College of Plant Protection, Shandong Agricultural University, Tai’an, Shandong 271018, P.R. China*

**Running title:** Identification and analysis *B. odoriphaga* OBPs and CSPs

**Corresponding authors:**

*Prof. Wei Mu, College of Plant Protection, Shandong Agricultural University, 61 Daizong Street, Tai’an, Shandong 271018, P.R. China. Tel: +86-538-8242611, Email: [muwei@sdau.edu.cn](mailto:muwei@sdau.edu.cn) (W. Mu).

**
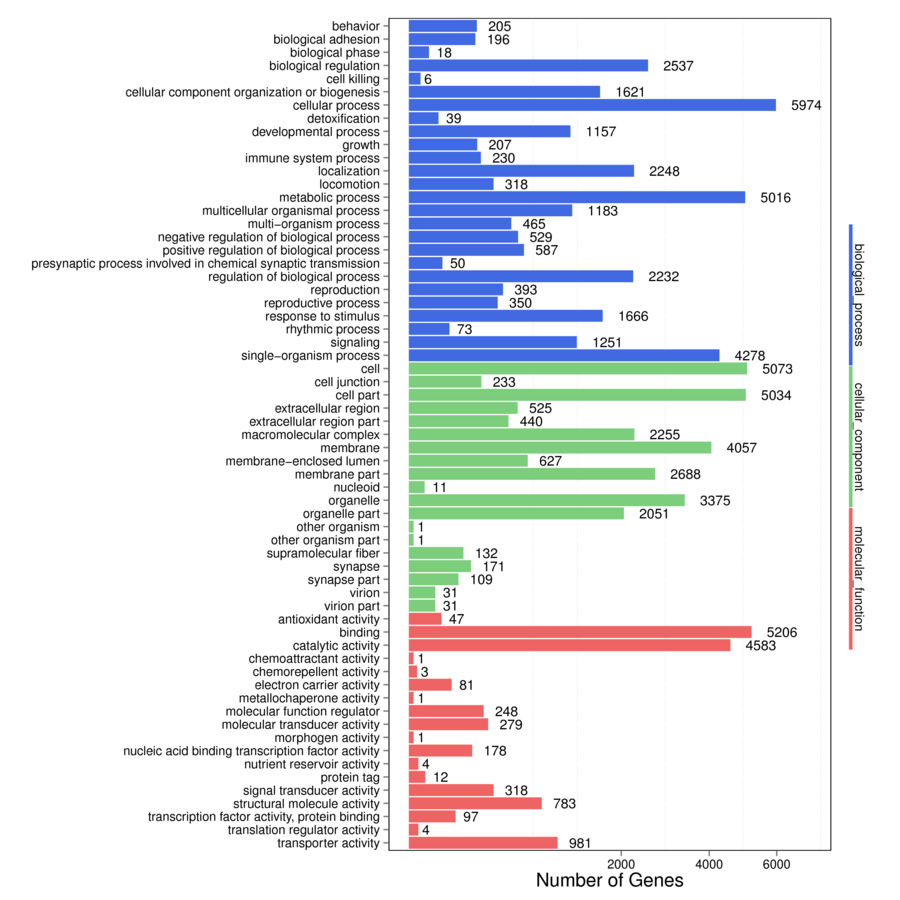
**

**Figure S1. Transcriptome GO annotation analysis of *B. odoriphaga*.** X axis represents the number of Unigenes. Y axis represents the Gene Ontology functional category.

**
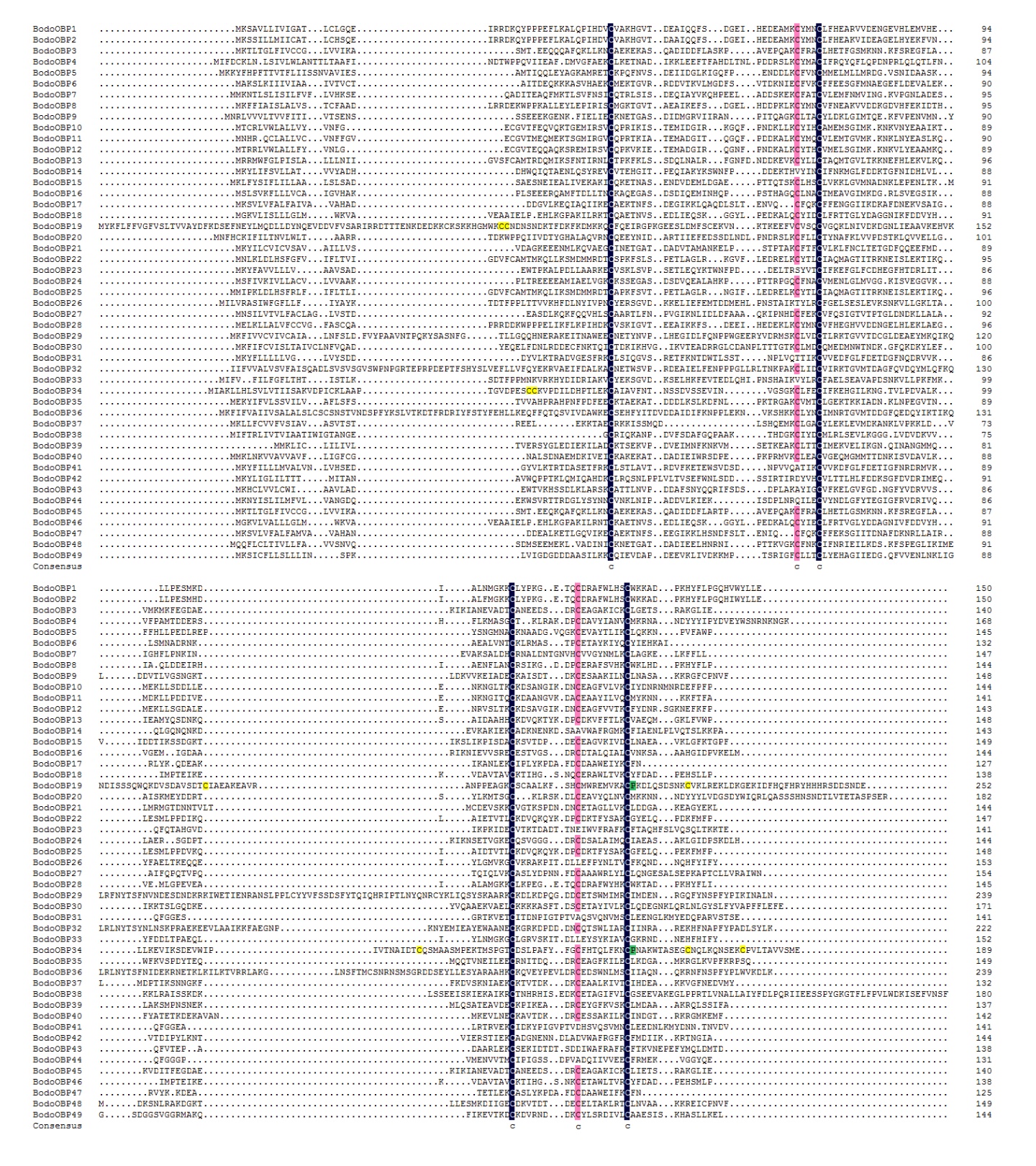
**

**Figure S2. Alignment of the identified *B. odoriphaga* OBPs.** Amino acid sequences of *B. odoriphaga* OBPs are aligned by Clustal X 2.0. Blue and pink boxes show conserved cysteine, and green boxes show conserved proline residues.

**
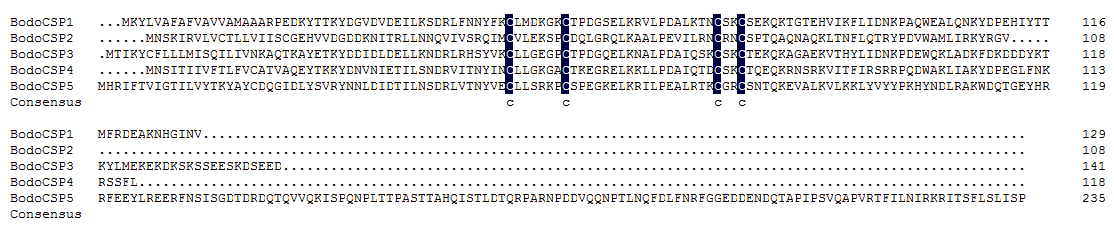
**

**Figure S3. Alignment of the identified *B. odoriphaga* CSPs.** Amino acid sequences of *B. odoriphaga* CSPs are aligned by Clustal X 2.0. Blue boxes show conserved cysteine.

**
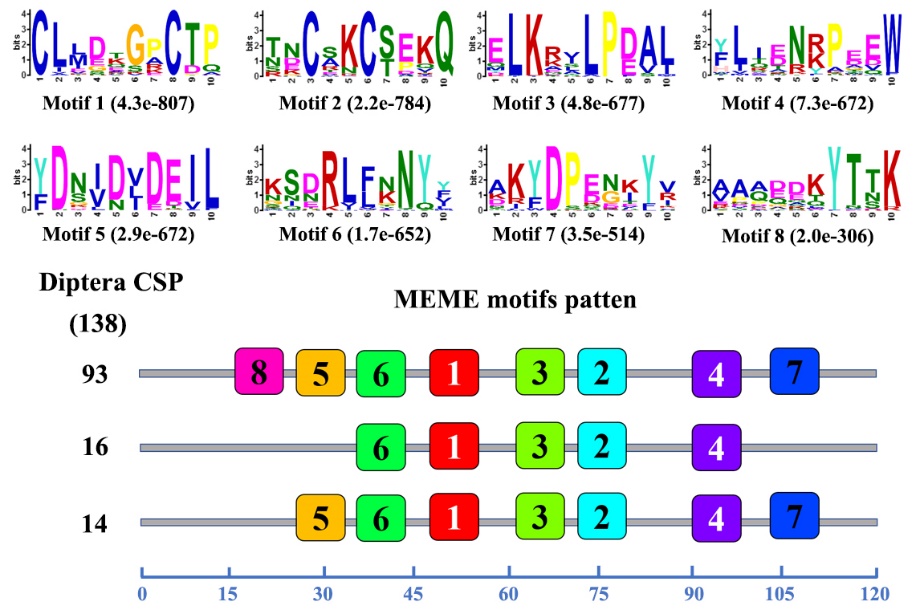
**

**Figure S4. Motif analysis of** **Diptera CSPs.** Parameters used for motif discovery were as follows: minimum width = 6, maximum width = 10, maximum number of motif to find = 8. The upper parts list the eight motifs discovered in the Diptera CSPs. The numbers in the boxes correspond to the numbered motifs in the upper part of the figure, where a small number indicates high conservation. The numbers on the bottom show the approximate locations of each motif on the protein sequence, starting from the N-terminus. The protein names and sequences of the 138 CSPs from different Diptera species are listed in **Table S4**.

**Table S1 Primers for cloning of OBP and CSP genes of *B. odoriphaga*.**

| Genes | Forward primer (5’ → 3’) | Reverse primer (5’ → 3’) |
| --- | --- | --- |
| **Odorant binding proteins (OBPs)** | | |
| *BodoOBP1* | ATGAAGTCCGCAGTTCTGTTGAT | TTATTCAAGAAGATACCAAACATGT |
| *BodoOBP2* | ATGAAGTCCTCGATTCTGTTGATGA | TTATTCAAGAAGATACCAAATATGTTG |
| *BodoOBP3* | ATGAAGACATTGACTGGTCTATTTATTG | CTACTCGATCAGACCTTTAGCACG |
| *BodoOBP4* | ATGATTTTCGACTGTAAACTTAATCTATC | TCACTTTCCGTTCTTATTCCTATTG |
| *BodoOBP5* | ATGAAGAAATATTTTCACCCATTC | TTAGGGCCACGCAAACAC |
| *BodoOBP6* | ATGGCCAAGTCATTAAAAATCA | TTAGATAGCTTTATGTTCAATATAGCAT |
| *BodoOBP7* | ATGATGAAAAACACACTTTCATTGA | TTAAAGTAAGAAAAATTTTAGCTCCTTT |
| *BodoOBP8* | ATGAAGTTTTTTATTGCCATAAGTC | TCAGGGCAAGAAATAATGTTTC |
| *BodoOBP9* | ATGAATCGTCTAGTTGTTGTGCTGA | TTAAAACACATTTGGACAGAAACCA |
| *BodoOBP10* | ATGACGTGTCGACTTGTGTGG | TTATGGAAATGGGAATTCGTC |
| *BodoOBP11* | ATGAATCATAGACAGTGCTTGGC | TTAAGCAAACGTGAACTTTTTATTG |
| *BodoOBP12* | ATGACTCGTCGACTTGTGTGG | TCATGGAAATTTGAATTCGTTT |
| *BodoOBP13* | ATGAGACGAATGTGGTTTGGATT | TCAGGGCCACACAAAAAGCT |
| *BodoOBP14* | ATGAAATACTTGATTTTCTCGGTAC | TTATGCCGGCTTCTTCAAAC |
| *BodoOBP15* | ATGAAGTTATTTTATTCGATATTTTTAA | TTAGAACGGTCCTGTTTTAAAGC |
| *BodoOBP16* | ATGAGTTTGTCGGTTAAGTTTTTG | CTACATCAATTCCTTGACGGG |
| *BodoOBP17* | ATGAAATCAGTTTTAGTTTTTGCTC | TTAATTGAAACATTTGTAAATTTCCC |
| *BodoOBP18* | ATGGGAAAAGTTTTAATTTCTTTGT | CTAGGGAAGTAGTGAATGCTCGG |
| *BodoOBP19* | ATGTATAAGTTTCTGTTTTTTGTTGGTT | TTACTCGTCATTGGAATCATCAGA |
| *BodoOBP20* | ATGAATTTTCACTGTAAAATCTTCAT | TCATCTTTCACTCGGTGAAGCT |
| *BodoOBP21* | ATGAAGTATATTTTATGTGTTATCTGTGT | CTACAATTTCTCGTAGCCAGCT |
| *BodoOBP22* | ATGAACCTAAAACTGGACCTCCA | TTAGGGAAACATAAATTTGTCCG |
| *BodoOBP23* | ATGAAGTACTTCGCTGTCGTTTT | TTATTCAGTTTTCTTCGTCAATTGA |
| *BodoOBP24* | ATGAGTTTTATAGTTAAAATTGTGCTTC | TTAATGCAAATCTTTAGATGGATCA |
| *BodoOBP25* | ATGATGATCCCAAAATTGGACC | TCAGGGAAACATAAACTTTTCCG |
| *BodoOBP26* | ATGATTTTGGTTAGAGCTTCAATCT | TCAGTAAAAAATATAAAAGTGCTGATTATC |
| *BodoOBP27* | ATGAATTCGATTTTGGTAACAGTTT | CTAATTCCAAATAGCTCGAACCA |
| *BodoOBP28* | ATGGAGTTGAAATTGTTGGCTT | CTAAATGAGGAAATAATGCTTTGGA |
| *BodoOBP29* | ATGAAATTTATTGTCGTTTGTGTCATTGT | TCAATTCAATGCATTGATCTTAATCGGA |
| *BodoOBP30* | ATGAAGTTTATTTTCTGTGTGATCAGTC | CTATTCAAATTCCAAGAAAAAAGGTGCAA |
| *BodoOBP31* | ATGAAATATTTCCTTTTACTGTTATTAGTC | CTATTCACTCGTGCTGACTCTTGC |
| *BodoOBP32* | ATTATTTTCGTTGTGGCTCTTGTCA | TTATTTAAGGTAAGACAAGTCAGCCGG |
| *BodoOBP33* | ATGATTTTCGTTTTCATTTTATTCGGTTT | TCAATAGAATATGTGAAAATGCTCATTGT |
| *BodoOBP34* | ATGATTGCGAAATTGCTTCATCTAAGT | TCACTCCATGCTAACCACTGC |
| *BodoOBP35* | ATGGAAAAATACATTTTCGTTTTGTCAA | TCATTGTGACGGACGTTTGAAAGG |
| *BodoOBP36* | ATGAAGTTCATTTTTGTTGCTATTATTG | TTACTTCAAATCTTTCACCCAAAGC |
| *BodoOBP37* | ATGAAAGTCTTAGTTGCCATCTG | CTAGGCCTTGGGGGCCAG |
| *BodoOBP38* | ATGATATTTACACGTTTAATCGTAACTG | TTAAAAACTATTCACAAATTCGCTTATC |
| *BodoOBP39* | ATGATGAAATTAATCTGTCTAATTTTGATT | TTACGCGAAAATGCTGCTCAATT |
| *BodoOBP40* | ATGATGAAATTAAATAAAGTCGTTGCT | TTAAAACATTTCCTTCATACCACGC |
| *BodoOBP41* | ATGAAATATTTCATTTTACTACTGATGGT | CTACACATCCACATTCGTATTATTG |
| *BodoOBP42* | ATGAAATATTTAATTGGACTTATACTGACG | TCATGCAATCCCATTTGTTCTC |
| *BodoOBP43* | ATGAAACATTGCCTTGTTGTATTG | TTAGTCCGTCATATCCAATTGCA |
| *BodoOBP44* | ATGAAGAATTACATTAGCCTGATATTAATG | TTATTCTTGATATCCTCCAACTTTCTC |
| *BodoOBP45* | ATGACTGAAGAGCAAAAGCAA | CTACTCGATCAGACCTTTAGCA |
| *BodoOBP46* | ATGGGAAAAGTTTTAGTTGCTCT | TTACGGTAGCATTGAGTGCT |
| *BodoOBP47* | ATGAAATCAGTTCTAGTTTTCGCT | TTAATTGAAACATTTGAAAATTTCCCAC |
| *BodoOBP48* | ATGCAACAGTTTCTCTGTTTGACT | TTAGAACACATTTGGGCAGATTTCT |
| *BodoOBP49* | ATGAAATCTATTTGCTTTCTTTTGTCTCT | TTATAATTCCTTCAGTAGACTTGCGTG |
| **Chemosensory proteins (CSPs)** | | |
| *BodoCSP1* | ATGAAATACTTAGTCGCTTTCGCAT | TTAGACATTGATACCATGGTTCTTAG |
| *BodoCSP2* | ATGAATTCAAAAATTCGAGTTTTAG | CTAAACTCCACGGTATTTTCTGATC |
| *BodoCSP3* | ATGACAATCAAATATTGTTTCCTAC | TCAGTCTTCTTCACTATCCTTACTT |
| *BodoCSP4* | ATGAATTCAATTACCATAATTGTATTT | TTATAGGAAGGATGACCTTTTGTTG |
| *BodoCSP5* | ATGCATCGTATTTTTACGGTAATTG | TCATGGACTAATGAGTGAGAGAAAT |

**Table S2 Amino acid sequences of 280 OBPs of *B. odoriphaga* and other Diptera insect used in phylogenetic analyses.**

>BodoOBP1

MKSAVLLIVIGATLCLGQEIRRDKQYPPPEFLKALQPIHDVCVAKHGVTDEAIQQFSDGEIHEDEAMKCYMNCLFHEARVVDENGEVHLEMVHELLPESMKDIALNMGKKCLYPKGETQCDRAFWLHSCWKKADPKHYFLPGQHVWYLLE

>BodoOBP2

MKSSILLMIICATLCHSQEIRRDKQYPPPEFLKALQPIHDVCVAKHGVTDAAIQQFSDGEIHEDEAMKCYMNCLFHEAKVIDEAGELHYEKFVNLLPESMHDIALFMGKKCLYPKGETQCDRAFWLHSCWKKADPKHYFLPGQHIWYLLE

>BodoOBP3

MKTLTGLFIVCCGLVVIKASMTEEQQQAFQKLLKNCAEKEKASQADIDDFLASKPAVEPQAKCFRACLHETFGSMKNNKFSREGFLAVMKMKFEGDAEKIKIANEVADTCANEEDSDRCEAGAKICKCLGETSRAKGLIE

>BodoOBP4

MIFDCKLNLSIVLWLANTTLTAAFINDTWPPQVIIEAFDMVGFAEKCLKETNADIKKLEEFTFAHDLTNLPDDRSLKCYMACIFRQYQFLQPDNPRLQLQTLFNVFPAMTDDERSHFLKMASGCTKLRAKDPCDAVYIANVCMKRNANDYYYIPYDVEYWSNRNKNGK

>BodoOBP5

MKKYFHPFTTVTFLIISSNVAVIESAMTIQQLEYAGKAMRETCKPQFNVSDEIIDGLKIGQFPENDDLKCFVNCMMELMLLMRDGVSNIDAASKFFHLLPEDLREPYSNGMNACKNAADGVQGKCEVAYTLIKCLQKKNPVFAWP

>BodoOBP6

MAKSLKIIIVIAAIVTVCTAITDEQKKKASVHAEKCMEKTGVRRDDVTKVLMGDFSVTDKNIECFVKCFFEESGFMNAEGEFLDEVALEKLSMNADRNKAEALVNTCKLRMASTPCETAYKIYQCYIEHKAI

>BodoOBP7

MMKNTLSLISILFVFLVHKSEQADITEAQFMKTLSVFNSICQTRLSISDEQIAYVKQHPEELADDSKEKCFATCVLEMFNMVINGKVPGNLADESIGHFLPNKINEVAKSALDHCRNALDNTGNVHCVVGYNMLKCLAGKELKFFLL

>BodoOBP8

MKFFIAISLALVSTCFAADLRRDEKWPPKALLEYLEPIRISCMGKTGVTAEAIKEFSDGELHDDPKLKCYMNCVFNEAKVVDDKGDVHFEKIDTHIAQLDDEIRHIAENFLANCRSIKGDDPCERAFSVHKCWKLHDPKHYFLP

>BodoOBP9

MNRLVVVLTVVFITIVTSENSSSEEEKGENKFIELIECKNETGASDIDMGRVIIRANPITQAGKCLTACYLDKLGIMTQEKFVPENVMNYLDDVTLVGSNGKTLDKVVKEIADECKAISDTDKCESAAKILNCLNASAKKRGFCPNVF

>BodoOBP10

MTCRLVWLALLVYVNFGECGVTFEQVQKTGEMIRSVCQPRIKISTEMIDGIRKGQFPNDKLLKCYIHCAMEMSGIMKKNKVNYEAAIKTMEKLLSDDLLEENKNGLTKCKDSANGIKDNCEAGFVLVKCIYDNRNMNRDEFPFP

>BodoOBP11

MNHRQCLALLVCVNFFGVECGVTMEQMEKTSGMIRGVCQPRTKIATEMADGITQGQFPDDKALKCYMQCVLEMTGVMKKNKLNYEASLKQMDKLLPDDIVEENKNGITQCKDAANGVKDACEAAYILVQCMYKNNKKFTFA

>BodoOBP12

MTRRLVWLALLFYVNLGECGVTEQQAQKSREMIRSVCQPKVKIETEMADGIRQGNFPNDKALKCYTHCVMELSGIMKKNKVLYEAAMKQMEKLLSGDALEENRVSLTKCKDSAVGIKDNCEAGFVVTKCFYDNRSGKNEFKFP

>BodoOBP13

MRRMWFGLPISLALLLNIIGVSFCAMTRDQMIKSFNTIRNLCTPKFKLSSDQLNALRFGNFDNDDKEVKCYLLCTAQMTGVLTKKNEFHLEKVLKQIEAMYQSDNKQSAIDAAHHCKDVQKTYKDPCDKVFFTLKCVAEQMGKLFVWP

>BodoOBP14

MKYLIFSVLLATVVYADHDHWQIQTAENLQSYREVCVTEHGITPEQIAKYKSWNFPDDEKTHVYINCIFNKMGLFDDKTGFNIDHLVLQLGQNQNKDEVKAKIEKCADKNENKDSAAVWAFRGMKCFIAENLPLVQTSLKKPA

>BodoOBP15

MKLFYSIFLILLAALSLSADSAESNEIEALIVEKAKICQKETNASENDVDEMLDGAEPTTQTSKCLHSCLVKKLGVMNADNKLEPENLTKMVIDDTIKSSDGKTIKSLIKPISDACKSVTDPDECEAGVKIVDCLNAEAVKLGFKTGPF

>BodoOBP16

MSLSVKFLLLVCAIGVHAKPLSEEERQAMFTDLLTNCKAQEGASDSDIQEMINHQPPSTHAGQCLNACTMEAVGIMKDGRLSVEGSIKVGEMIGDAARIKNIEVVSRECESTVGSDRCDTALQIALCVNKSAAAHGIDPVKELM

>BodoOBP17

MKSVLVFALFAIVAVAHADDDGVLKEQIAQIIKECAEKTNFSDEGIKKLQAQDLSLTENVQCFQKCFFENGGIIKDKAFDNEKVSAIGRLYKQDEAKIKANLEKCIPLYKPDAFDCDAAWEIYKCFN

>BodoOBP18

MGKVLISLLLGLMWKVAVEAAIELPEHLKGPAKILRKTCQAETNVSEDLIEQSKGGYLPEDKALQCYIDCLFRTTGLYDAGGNIKFDDVYHIMPTEIKEKVDAVTAVCKTIHGSNQCERAWLTVKCYFDADPEHSLLP

>BodoOBP19

MYKFLFFVGFVSLTVVAYDFKDSEFNEYLMQDLLDYNQEVDDVFVSARIRRDTTTENKDEDKKCKSKKHGMWKCCNDNSNDKTFDKFKDMKKQCFQEIRGPKGEESLDMFSCEKVNKTKEEFVCVSQCVGQKLNIVDKDGNLIEAAVKEHVKNDISSSQWQKDVSDAVSDTCIAEAKEAVRANPPEAGKCSCAALKFSHCMWREMVKACPKDLQSDSNKCVKLREKLDKGEKIDFHQFHRYHHHRSDDSNDE

>BodoOBP20

MNFHCKIFILTNVLWLTAARRTDKWPPQIIVDTYGHALAQVRNCQEEYNIDARTIIEFEDSSDLNDLPNDRSLKCFLLCTYNAFKLVVPDSTKLQVVELLGAISKMEYDDRTSYLKMTSGCKLRSKDLCEAVYQLNVCMKKNNNDYYYLVDGSDYWIQRLQASSSHNSNDTLVTETASPSER

>BodoOBP21

MKYILCVICVSAVAILLVSVDAGKEEENMLKQVAEGCINETGATDADVTAMANKELPSTPTAKCFTFCVLKLFNCLTETGDFQQEEFMDLMRMGTDNNTVLTMCDEVSKKCVGTKSPDNDNCETAGLLVKCLDDGAKEAGYEKL

>BodoOBP22

MNLKLDLHSFGFVIFLTVIGDVFCAMTMKQLLKSMDMMRDTCSPKFSLSPETLAGLRKGVFLEDRELKCYTLCIAQMAGTITRKNEISLEKTIKQLESMLPPDIKQLAIETVTLCKDVQKQYKDPCDKTFYSAKCGYELQPDKFMFP

>BodoOBP23

MKYFAVVLLLVAAVSADEWTPKALPDLLAARKECVSKLSVPSETLEQYKTWNFPDDELTRSYVTCIFKEFGLFCDHEGFHTDRLITQFQTAHGVDIKPKIDECVTKTDADTTNEIWVFRAFKCFTAQHFSLVQSQLTKKTE

>BodoOBP24

MSFIVKIVLLACVLVVAAKPLTREEEEAMIAELVGKCKSSEGASDSDVQEALAHKPPTTRPGQCFNACVMENLGLMVGGKISVEGGVKLAERSGDPTKIKNSETVGKECQSVGGGDRCDSALAIMQCIAEASAKLGIDPSKDLH

>BodoOBP25

MMIPKLDLHSFRLFIFLTLIGDVFCAMTMKQLIKSMDMMRDTCAPKFSVTPETLAGLRNGIFLEDRELKCYTLCIAQMAGTITRKNEISLEKTIKQLESMLPPDVKQIAIDTVTLCKDVQKQYKDPCDKTFYSAKCGFELQPEKFMFP

>BodoOBP26

MILVRASIWFGFLLFIYAYKTDTFPPLTTVVKHFDLNYIVPNCYERSGVDKKELIEFEMTDDMEHLPNSTAIKTYLRCFGELSESLEVKSNKVLLGKLTAYFAELTKEQQEIYLGMVKGCVKRAKPITDLLEFPYNLTVCFKQNDNQHFYIFY

>BodoOBP27

MNSILVTVLFACLAGLVSTDEASDLKQKFQQVHLSCAARTLFNPVGIKNLIDLDFAAAQKIPNHDCFEKCVFQSIGTVTPTGLDNDKLLALAAIFQPQTVPQTQIQLVKCASLYDPNNFDCAAAWRLYLCLQNGESALSEPKAPTCLLVRAIWN

>BodoOBP28

MELKLLALVFCCVGFASCQAPRRDDKWPPPELIKFLKPIHDKCVSKIGVTEEAIKKFSDEEIHEDEKLKCYMNCVFHEGHVVDDNGELHLEKLAEGVEMLGPEVEAIALAMGKKCLKPEGETQCDRAFWYHKCWKTADPKHYFLI

>BodoOBP29

MKFIVVCVIVCAIALNFSLDFVYPAAVNTPQKYSASNFGTLLGQQHNERAKEITNAWEECNETYNVPLHEGIDLFQNPPWGEERYVDRMSKCLVDCTLRKTGVVTDCGLDEAEYMKQIKQLRFNYTSFNVNDESDNDKRKIWETIENRANSLPPLCYYVFSSDSFYTQIQHRIPTLNYQNRCYKLIQSYSKAARKCKDLKDPQGDDCETSWMIMRCIMDENRGQFYNSPFYPIKINALN

>BodoOBP30

MKFIFCVISLTAIVCLNFVQADYEQELFDNLRDDECFNKTQICTDKIKHVGIKVTEADRRGLCDANPLTTTGTKCLMDCQMEDMNWTNDKGFQKDKYLEFIKKTSLGQDKEYVQAAEKVAELCKKKKASFTDSCETAYIVLKCLQDEGNKLQRLNLGYSLFYVAPFFLEFE

>BodoOBP31

MKYFLLLLLVGLVYSDDDYVLKTRADVGESFRKCLSIQGVSRETFKNTDWTLSSTNPLVQTTIKCVVEDFGLFDETDGFNQDRVVKQFGGESGRTKVETCITDNPIGTPTVAQSVQNVMSCLEENGLKMYEDQPARVSTSE

>BodoOBP32

IIFVVALVSVFAISQADLSVSVSGVSWPNPGRTEPRPDEPTFSHYSLVEFLLVFQYEKRVAEIFDALKACNETWSVPRDEAIELFENPPPGLLRLTNKPAKCLIDCVIRKTGVMTDAGFQVDQYMLQFKQLRLNYTSYNLNSKPRAEKEEVLAAIKKFAEGNPKNYEMIEAYEWAANECKGRKDPDDDNCQTSWLIARCIINRAREKHFNAPFYPADLSYLK

>BodoOBP33

MIFVFILFGFLTHTISTLKSDTFPPMNKVRKHYDIDRIAKVCYEKSGVDKSELHKFEVTEDLQHIPNSHAIKVYLRCFAELSEAVAPDSNKVLLPKFMKYFDDLTPAEQLIYLNMGKGCLGRVSKITDLLEYSYKIAVCGKRNDNEHFHIFY

>BodoOBP34

MIAKLLHLSVLVTIISAKVDPICKLAAPTGVDPESCCKVPDILDHPTLEKCAIAVFNTNSSDVSSEVINVGSGKCLFECIFKEHGILKNGTVLPDVALKLLKEVIKSDEVWIPIVTNAIDTCQSMAASMPEKTMSPGTCDSLPAFYFGCFHTQLFKNCPNAKWTASEGCNQLKQNSEKCPVLTAVVSME

>BodoOBP35

MEKYIFVLSSVILVAFLSFSTVVAHPRAHPNFPDFEECKTAEKATDDDLKSLKDFNLPKTRGAKCVMTCLGEKTKKIADNKLNPEGVTNWFKVSPDYTEQMQQTVNEILEECRNITDQDRCEAGFKILECLKDGAMKRGLKVPFKRPSQ

>BodoOBP36

MKFIFVAIIVSALALSLCSCSNSTVNDSPFYKSLVTKDTFRDRIYFSTYFEHLLKEQFFQTQSVIVDAWKECSEHFYITDVDDAIDIFKNPPLEKNVKSHKKCLYNCIMNRTGVMTDDGFQEDQYIKTIKQLRLNYTSFNIDEKRNETKLKILKTVRRLAKGLNSFTMCSNRNSMSGRDDSEYLLESYARAAHKCKQVEYPEVLDRCEDSWNLMSCIIAQNQKRNFNSPFYPLWVKDLK

>BodoOBP37

MKLLFCVVFVSIAVASVTSTREELEKKTAECRKKISSMQDLSHQEMKCLGACYLEKLEVMDKANKLVPKKLDVLMDPTIKSNNGKFFKDVSKNIAEKCKTVTDKDKCEAALKIVTCIHDEAKKVGFNEDVMY

>BodoOBP38

MIFTRLIVTVIAATIWIGTANGEGCRIQKANPDVFSDAFGQPAAKTHDGKCIYDCMLRLSEVLKGGGLVDVDKVVKKLRAISSKDKLSSEEISKIEKAIKRCTNHRHISEDKCETAGIFVLCGSEEVAKEGLPPRTLVNALLAIYFDLPQRIIEESSPYGKGTFLFPVLWDKISEFVNSF

>BodoOBP39

MMKLICLILIVLTVERSYGLEDIEKILADCKTSEKVPDVEIMNFKNKVMSETKEAKCLTTCIMEKVELIKGNQINANGMMQLAKSMPNSNEKMLQSATEAVDECKPIKEADRCEYGFKVSKCLMDAAAKRQLSSIFA

>BodoOBP40

MMKLNKVVAVVAVFLIGFCGNALSDNAEMDKIVEICKAKEKATDADIEIWRSDPEPKPRMVKCLEACVGEQMGMMTTDNKISVDAVLKFYATETKDEKAVANMKEVLNECKAVTDKDRCESSAKILKCINDGTRKRGMKEMF

>BodoOBP41

MKYFILLLMVALVNLVHSEDGYVLKTRTDASETFRKCLSTLAVTRDVFKETEWSVDSDNPVVQATIKCVVKDFGLFDETIGFNRDRMVKQFGGEALRTRVEKCIDKYPIGVPTVDHSVQSVMNCLEEDNLKMYDNNTNVDV

>BodoOBP42

MKYLIGLILTTTMITANAVWQPPTKLQMIQAHDKCLRQSNLPPLVLTVSEFWNLSDDSSIRTIRDYVHCVLTTLHLFDDKSGFDVDRIMEQVTDIPYLKNTVIERSTIEKCADGNENNDLADVWAFRGFRCFMDIIKKRTNGIA

>BodoOBP43

MKHCLVVLCWIAAVLADEWTVKHSSDLKLARSKCATTLNVPDDAFSNYQQRIFSDSDPLAKAYIGCVFKELGVFGDNGFYVDRVVSQFVTEPADAARLEKCSEKIDTDTSDDIWAFRAFRCFTKVNEPEFYMQLDMTD

>BodoOBP44

MKNYISLILMFVLVANGDQEKWSVRTTRDGLYSYNNCVNKLNIPADDVLKIEKISDPLNRQILECVYNDLGFYTEGIGFRVDRIVQQFGGGPVMENVVTMCIPIGSSDPVADQIIVVEECFRMEKVGGYQE

>BodoOBP45

MKTLTGLFIVCCGLVVIKASMTEEQKQAFQKLLKNCAEKEKASQADIDDFLARTPAVEPQAKCFRACLHETLGSMKNNKFSREGFLAKVDITFEGDAEKIKIANEVADTCANEEDSDRCEAGAKICKCLIETSRAKGLIE

>BodoOBP46

MGKVLVALLLGLMWKVAVEAAIELPEHLKGPAKILRNTCKAETNVSEDLIEQSKGGYLPEDKALQCYIECLFRTVGLYDDAGNIVFDDVYHIMPTEIKEKVDAVTAVCKTIHGSNKCETAWLTVRCYFDADPEHSMLP

>BodoOBP47

MKSVLVFALFAMVAVAHANDDEALKETLGQVIKECAEKTNFSEEGIKKLHSNDFSLTENIQCFQKCFFEKSGIITDNAFDKNRLLAIRRVYKKDEATETLEKCASLYKPDAFDCDAAWEIFKCFN

>BodoOBP48

MQQFLCLTIVLLFAVVSNVQSDMSEEMEKLVADINICKNETGATDADIEELHNRNIPTTKVGKCFNKCIFNRIEILKDSKFSPEGLIKIMEMDKSNLRAKDGKTLLESMKDIIGECDKVTDTDECELTAKLRTCLNVAAKKREICPNVF

>BodoOBP49

MKSICFLLSLLLINSPKLVIGDGDDDAASILKKCQIEVDAPDEEVKLIVDKKMPTSRIGFCLLTCLYEHAGIIEDGQFVVENLNKLIGGSDGGSVGGRMAKQFIKEVTKDCKDVRNDDKCYLSRDIVLCAAESISKHASLLKEL

>DmelOBP8a

MAHCMYILLLLLLVVELTPPAIPVPMRSSPQSLALLRARDQCGRELTAAQRLQLDRMQFEDAAHVRHYLHCFWSRLQLWLDETGFQAQRIVQSFGGERRLNVEQALPAINGCNAKTSSRGSGAQTVVDWCFRAFVCVLATPVGEWYKRHMSDVINGNA

>DmelOBP18a

MKVVCSIAVLWICLITMWQSAGRVNAEGCLKHHNLTSAQVQAVAPSTPVADVPVAVKCYSRCLIQDYFGDDGKIDLQKVGKRGSQEDHVILSQCKQQFDGVTNLDTCDYPYLILQCYFKGKQSGTIAS

>DmelOBP19a

MKFHLLLVCVAISLGPIPQSEAGVTEEQMWSAGKLMRDVCLPKYPKVSVEVADNIRNGDIPNSKDTNCYINCILEMMQAIKKGKFQLESTLKQMDIMLPDSYKDEYRKGINLCKDSTVGLKNAPNCDPAHALLSCLKNNIKVFVFP

>DmelOBP19b

MTNLLLAVACAAVLMGSATADEEEGSMTVDEVVELIEPFGDACTPKPSRENIVEMVLNKEDAKHETKCFRHCMLEQFELMPEDQLQYNEDKTVDMINMMFPDREDDGRRIVKTCNEELKAEQDKCEAAHGIAMCMLREMRSSGFKIPEIKE

>DmelOBP19c

MKPSTPVAAIPLMTIVVAVLLQTHCVRGQTQAFDLAKLLPKTGTEPIWAVIDRNLPQVQELVTAARMECIQKLQLPRDQRPLGKVTNPSEKEKCLVECVLKKIKLMDADNKLNVGQVEKLTSLVTQDNKMAIAVSSSMAQACSRGISSKNPCEVAHLFNQCISRQLERNNVKLVW

>DmelOBP19d

MSHLVHLTVLLLVGILCLGATSAKPHEEINRDHLLELANECKAETGATDEDVEQLMSHDLPERHEAKCLRACVMKKLQIMDESGKLNKEHAIELVKVMSKHDAEKEDAPAEVVAKCEAIETPEDHCDAAFAYEECIYEQMREHGLELEEH

>DmelOBP28a

MQSTPIILVAIVLLGAALVRAFDEKEALAKLMESAESCMPEVGATDADLQEMVKKQPASTYAGKCLRACVMKNIGILDANGKLDTEAGHEKAKQYTGNDPAKLKIALDIGETCAAITVPDDHCEAAEAYGTCFRGEAKKHGLL

>DmelOBP44a

MKNAVAILLCALLGLASASDYKLRTAEDLQSARKECAASSKVTEALIAKYKTFDYPDDDITRNYIQCIFVKFDLFDEAKGFKVENLVAQLGQGKEDKAALKADIEKCADKNEQKSPANEWAFRGFKCFLGKNLPLVQAAVQKN

>DmelOBP46a

MCSQLFAFLLLLLTAFVTGRSTPPALDEDCELNSVDTMHDFCCDLHDESPQFSDCQMEWHEKIPYETDEEEQTYMFCTAECSFNSTNFLGRDRRSLNLNEVKEHLESDLVNDADIKLLYDTYVKCDKHALSLMPHKGVKQLSKRLSRLGCHPYPGLVLECVANEMILHCPTKRFRQTAQCEETRNHLKQCMQYLKYKS

>DmelOBP47a

MNRVLVLLLVLKMFALSESRFAKININLGLTVADESPKTITEEMIRLCGDQTDISLRELNKLQREDFSDPSESVQCFTHCLYEQMGLMHDGVFVERDLFGLLSDVSNTDYWPERQCHAIRGNNKCETAYRIHQCQQQLKQQQQNLLATKEVEVTTTPAGSDETKP

>DmelOBP47b

MSPSQLLVIFASLALNTRLVFGQATIDCQRPPQLVDPALCCKDGGRDQVAEQCAQRILGTANGQKAGGPPSLDTAACLAECILTSSKYIDEPQKLNLANIRSDLSAKFSNDTLYVETMTMAFSKCEPQSQRRLAMIMQQQQQVQQQKTQQQQPRCSPFSAIVLGCTYMEYFKNCPDHRWTPNAQCTLAKAYVTQCGLGA

>DmelOBP49a

MLSKSQLLLLVVGFCLNAAVSADVDCSKRPSFVNPKTCCPMPDFVTAELKQKCIKFDMTPPPPPDGEASGSFESKRRHHHPHPPPCFFSCIFNETGIYQNRKLDEAKLNAYLQEVFEDSSDLQTTATQAFTTCATKVADFEANLPPRPAPSPPPGFPMCPHDAGHLMGCVFRNMMKNCPDSIRNDSQQCTDMKEFFTKCKPPRGPPPSAEDM

>DmelOBP50a

MRTGRILVALIFLGLIIPFRAAKCRAAPKSVQNVHVCCSAPLPNWGVFNRECHKSAIQASVSINRISKSKVNLANFLIKCRLDCDFNASSVLQGNRLIQAKVRPMLERAFSNEPTIDAYESNFAKCSTVVRSKYQELSPLSRQSDACDRHALFYSLCAYARLIFTCPDKMWQRNNRMCQEAKAYAKKCPWPALKMFMRNT

>DmelOBP50b

MSSVLHLLGFLWLPLLVYSVSNDMGGLQKCTELLNTHKLVYCCGKSFLDKFPFVGSNCTPFWDDYGPCRYECLYRHWDLLDQDNKIKKPELYLMITSLYSPLNGYDKYGAAFKAAHETCEALGSRHADFLLLYSNQVADKMGMASSTCLPYAMLHAQCTMVYLTANCPRENWIDDPKCNSLQKLLSSCTKKLDEKTNALKGKDEELTDNGCGHIDSEGSNLLMACFLTLMIAKFISDH

>DmelOBP50c

MARHIALLICSLLAMAGCDPIDVDCTRRQDFNIVKDCCVYPTFRFDQFKSQCGKYMPVGAPRISPCLYECIFNKTNTVVDGAIHPDNARLMLEKLFGNQDFEEAYFNGLMGCSDSVQEMISNRRSRPQRKTEQCSPFSLFYGICAQRYVFNHCPSSSWSGTESCEMARLQNMNCSKPSRGSSHRL

>DmelOBP50d

MLHKLTWVLIFIPAFRAADPICSQRPDVTALRNCCKLPNLDFSSFNSKCSQYLVNGVHISPCSFECIFRAANALNGTHLVMENIEKMMKTILGSDEFVHVYLDGFRSCGNQEKVLIKAMKRRRVPITGKCGSMAIMYGLCAHRYVYRNCPESVWSKSATCNEAREYSIRCDDM

>DmelOBP50e

MHKYIICFGFLLIILECSLASFNCSAPPNFNNFDINTCCRTPELDMGDVPQKCHKYVSGLKSANSKYPSYAHLCYPDCIYRETGAMVNGKIKVNRVKQYLEEHVHRRDQEIVSHIVQSFESCLSNVKGHMKSLNIESYKVLPHGCSPFAGIIYSCVNAETFLNCPQQMWKNEKPCNLAKQFAEQCNPLPHVPLPSS

>DmelOBP51a

MKVFIGLVLLLAVTTLSSALFESEANECAKKLGITPDYFENFPHSSRVKCFYHCQMEKLEIIANGVVTPFDLKVLNISPESYDKYGVKVKPCLKLSHRDKCELGYLVFQCLKREFNL

>DmelOBP56a

MNSYFVIALSALFVTLAVGSSLNLSDEQKDLAKQHREQCAEEVKLTEEEKAKVNAKDFNNPTENIKCFANCFFEKVGTLKDGELQESVVLEKLGALIGEEKTKAALEKCRTIKGENKCDTASKLYDCFESFKPAPEAKA

>DmelOBP56b

MKLIYLLVVFLIFALSELVAGQSAAELAAYKQIQQACIKELNIAASDANLLTTDKEVANPSESVKCYHSCVYKKLGLLGDDGKPNTDKIVKLAQIRFSSLPVDKLKSLLTSCGTTKSAATCDFVYNYEKCVVKGISA

>DmelOBP56c

MYFRASLMALLCLTLSEFVSKAWVMFFIFYISFTRSLSVSLNMSMTRTLVPDPPNGTENKLSQEMLRACMRRTEISMSQLKLFHMSLMNSDYNNDNDIAPTPVQSIGDVNNLGDLDFNGNSQMPYLDLKHNEPLQCFVSCLYETLDLDRYNVLLEEAFKNQVQTIIQHEKAEIKECSDLQGKTRCEAAYKLHLCYNHLKTLEAEQRIREILERTEAENEGFGPEGSDFIDGIQHSGEAMTTAKSE

>DmelOBP56d

MKFLIVLSVILAISAAELQLSDEQKAVAHANGALCAQQEGITKDQAIALRNGNFDDSDPKVKCFANCFLEKIGFLINGEVQPDVVLAKLGPLAGEDAVKAVQAKCDATKGADKCDTAYQLFECYYKNRAHI

>DmelOBP56e

MKVFFVFAALAALSLASAVGLTDSQKAEAKQRAKACVKQEGITKEQAIALRSGNFADSDPKVKCFANCFLEQTGLVANGQIKPDVVLAKLGPIAGEANVKEVQAKCDSTKGADKCDTSYLLYKCYYENHAQF

>DmelOBP56f

MKVFLLFIFISAIWLQAFCMKSSEKIKACLKRQLGYTITENTKFDAKEDSLQSKCFYHCLLEVKGVIANDAISSEQPRKVLEKKYGITDTDELEKAEEKCHSIKASGKCELGYEILKCYQSITKH

>DmelOBP56g

MRATFALTLLLGCLSGILAQQANIDSSVSKELVTDCLKENGVTPQDLADLQSGKVKAEDAKDNVKCSSQCILVKSGFMDSTGKLLTDKIKSYYANSNFKDVIEKDLDRCSAVKGANACDTAFKILSCFQAAN

>DmelOBP56h

MKFTLFCIALAAFLSMGQCNPDFRQIMQQCMETNQVTEADLKEFMASGMQSSAKENLKCYTKCLMEKQGHLTNGQFNAQAMLDTLKNVPQIKDKMDEISSGVNACKDIKGTNDCDTAFKVTMCLKEHKAIPGHH

>DmelOBP56i

MHFFTCCALLLVVVTLPTCFVQAGPIKDQCMAAAGITAQDVANRHETDDPGHSVKCFFRCFLENIGIIADNQIIPGAFDRVLGHIVTAEAVERMEATCNMIKSETSHDESCEFAWQISECYEGVRLSDVKKGQRTRNHRG

>DmelOBP57a

MLKLWLICILTVSVVSIQSLSLLEETNYVSDCLASNNISQAEFQELIDRNSSEEDDLENTDRRYKCFIHCLAEKGNLLDTNGYLDVDKIDQIEPVSDELREILYDCKKIYDEEEDHCEYAFKMVTCLTESFEQSDEVTEAGKNTNKLNE

>DmelOBP57b

MFIYRLVFIAPLILLLFSLAKARHPFDIFHWNWQDFQECLQVNNITIGEYEKYARHETLDYLLNEKVDLRYKCNIKCQLERDSTKWLNAQGRMDLDLMNTTDKASKSITKCMEKAPEELCAYSFRLVMCAFKAGHPVIDSE

>DmelOBP57c

MFNTRLAIFLLLIVVSLSQAKESQPFDFFEGTYDDFIDCLRINNITIEEYEKFDDTDNLDNVLKENVELKHKCNIKCQLEREPTKWLNARGEVDLKSMKATSETAVSISKCMEKAPQETCAYVYKLVICAFKSGHSVIKFDSYEQIQEETAGLIAEQQADLFDYDTIDL

>DmelOBP57d

MLDQLTLCLLLNFLCANVLANTSVFNPCVSQNELSEYEAHQVMENWPVPPIDRAYKCFLTCVLLDLGLIDERGNVQIDKYMKSGVVDWQWVAIELVTCRIEFSDERDLCELSYGIFNCFKDVKLAAEKYVSISNAK

>DmelOBP57e

MSLRLVPHLACIIFILEIQFRIADSNDPCPHNQGIDEDIAESILGDWPANVDLTSVKRSHKCYVTCILQYYNIVTASGEIFLDKYYDTGVIDELAVAPKINRCRYEFRMETDYCSRIFAIFNCLRQEILTKS

>DmelOBP58a

MKQLIFLLICLSCGTCSIYALKCRSQEGLSEAELKRTVRNCMHRQDEDEDRGRGGQGRQGNGYEYGYGMDHDQEEQDRNPGNRGGYGNRRQRGLRQSDGRNHTSNDGGQCVAQCFFEEMNMVDGNGMPDRRKVSYLLTKDLRDRELRNFFTDTVQQCFRYLESNGRGRHHKCSAARELVKCMSEYAKAQCEDWEEHGNMLFN

>DmelOBP58b

MLRIGFVICVIISLRLNGLVAVRVHCRHMERIHEENIHHCCKHQDGHDDVTESCAKQTNFRLPSPNEEAIVDVTVDQAMVGTCWAKCVFDHYNLMENNTLDMDKVRSYYKRYHQTDPEYATEMLNAYEKCHTQSEEATEKFLSLPIVRAFSTAKFCKPTSSIIMSCVIYNFFHNCPASRWSNTTECVETLAFARKCKDVLTT

>DmelOBP58c

MKCTILLSFFSLIWFAGGIKIDCENTEAINEDHIHYCCKHPDGHNDLIEGCARETNFTLPNQNEEALVDITADRAIRGTCFGKCVFSKLNLMKDNNLDMDAVRSLFTERFPDDPEYAKEMINAFDHCHGKSEENTSMFLSKPLFKQMSKQFCDPKSSVVLACVIRQFFHNCPADRWSKTKECEDTLAFSKKCQDSLATL

>DmelOBP58d

MVNIVCYWTFLILVAVSKAQDNEETTAVAISSGDLTEDKCNTSRAGCCSELYIGEEEDLVKCFVIHSPKLPVDGDADIGKTLRFLSCFVECLYKQKKYIGKSDTINMKMVKLDAEKTFVDRPKEKDYHIAMFEFCRKDAVGVYNLLKASPGAKVLLKGACRPYLLMVFMCISDYHQKHECPYFRWEGTAKAGTKDMCENAKAECYQIDGITLPTKSPA

>DmelOBP69a

MVARHFSFFLALLILYDLIPSNQGVEINPTIIKQVRKLRMRCLNQTGASVDVIDKSVKNRILPTDPEIKCFLYCMFDMFGLIDSQNIMHLEALLEVLPEEIYKTINGLVSSCGTQKGKDGCDTAYETVKCYIAVNGKFIWEEIIVLLG

>DmelOBP76a

MKHWKRRSSAVFAIVLQVLVLLLPDPAVAMTMEQFLTSLDMIRSGCAPKFKLKTEDLDRLRVGDFNFPPSQDLMCYTKCVSLMAGTVNKKGEFNAPKALAQLPHLVPPEMMEMSRKSVEACRDTHKQFKESCERVYQTAKCFSENADGQFMWP

>DmelOBP83a

MALNGFGRRVSASVLLIALSLLSGALILPPAAAQRDENYPPPGILKMAKPFHDACVEKTGVTEAAIKEFSDGEIHEDEKLKCYMNCFFHEIEVVDDNGDVHLEKLFATVPLSMRDKLMEMSKGCVHPEGDTLCHKAWWFHQCWKKADPKHYFLP

>DmelOBP83b

MVKYPLILLLIGCAAAQEPRRDGEWPPPAILKLGKHFHDICAPKTGVTDEAIKEFSDGQIHEDEALKCYMNCLFHEFEVVDDNGDVHMEKVLNAIPGEKLRNIMMEASKGCIHPEGDTLCHKAWWFHQCWKKADPVHYFLV

>DmelOBP83c

MQMKSGILIALCLCLSLNEGLALLEHEGETINRCIQNYGGLTAENAERLERFKEWSDSYEEIPCFTRCYLSEMFDFYNNLTGFNKDGIVGVFGRPVYEACRKKLELPFESGESSCKHAYEGFHCITNKEF

>DmelOBP83d

MESHPFTVIDNMPNISPSAKDAMKDCLQDVHQDEWKSFDAFAYYPVNEPIPCFTRCFVDKLHIFEEKTRLWKLEAMKQNLGIPAKGARIRTCHRHRGRDRCATYYKQFTCYAMAV

>DmelOBP83e

MSSPRAVLVSLFLICSQALADLSGDAQTLEKCLRQLSSPESIAGDLRKLERYSSWTREEVPCLMRCLAREKGWFDVEENKWRLKQLTEDLGADVYNYCRFELRRMGSDGCSFAYRGLRCLKQAEMHAGTSLSTLLQCSRQLNATNVELLQYSKLKSKEPIPCLFQCFADAMGFYDPDGNWRLENWKQAFGPSGNEDQSSGADYSGCRLSGTQREVALSKCSWMYHEYKCWERVNGNKLVEDNEEQ

>DmelOBP83f

MQSQSLLLIVAAVATFLVAQVRAQWLPLLMETTAKFLLKDHADAEKAFEECREDYYVPDDIYEKYLNYEFPAHRRTSCFVKCFLEKLELFSEKKGFDERAMIAQFTSKSSKDLSTVQHGLEKCIDHNEAESDVCTWANRVFSCWLPINRHVVRKVFA

>DmelOBP84a

MYSALVRACAVIAFLILSPNCARALQDHAKDNGDIFIINYDSFDGDVDDISTTTSAPREADYVDFDEVNRNCNASFITSMTNVLQFNNTGDLPDDKDKVTSMCYFHCFFEKSGLMTDYKLNTDLVRKYVWPATGDSVEACEAEGKDETNACMRGYAIVKCVFTRALTDARNKPTV

>DmelOBP85a

MSPGSVVFSMFLTRPSLDKGNSECRKSLNLPAHRKFNFAELYTINMCIEECNFIGCGYIEIDPPFRLDLANIRTNLQTIAPQPQNESIPFLVDAYRKCELFRSSHGRRFTLHLPDIEFIEEPCNPFALQITICVRIHAMQKCPSEFYVDSDECRLAREYFTQCVGDIETNLA

>DmelOBP93a

MKTSNKIVFLLLQLNIWQLSSCCDVQKNDKAINSCRKSLLGNNSTNSNGEVRNLKSDKVALHACIAECSFRTNGFLLSNGTVNTQALQKSYQQRYKNDPNMSQLMLKSLNSCTDYARKRVQEFQWMPKKGDCDFYPATLLACVMEKVYINCPTSKWKNTSDCTAMWKYLVACDDVASNKKK

>DmelOBP99a

MKVFVAICVLIGLASADYVVKNRHDMLAYRDECVKELAVPVDLVEKYQKWEYPNDAKTQCYIKCVFTKWGLFDVQSGFNVENIHQQLVGNHADHNEAFHASLAACVDKNEQGSNACEWAYRGATCLLKENLAQIQKSLAPKA

>DmelOBP99b

MLKYLIVALALCAVAHADDWTPKTGEEIRKIRVDCLKENPLSNDQISQLKNLIFPNEPDVRQYLTCSAIKLGIFCDQQGYHADRLAKQFKMDLSEEEALQIAQSCVDDNAQKNPTDVWAFRGHQCMMASKIGDKVRAFVKAKAEEAKKKAA

>DmelOBP93c

MNHLRLEIICWSCLLIAMAVSTEAASVWKLPTAQMVYEDLEKCRQESQEEDAATLRCLVKKLGLWTDESGYNARRIAKIFAGHNQMEELMLVVEHCNRMEQDTSHLDDWAFLAYRCATSGQFGHWVKDFMSQKEVER

>DmelOBP93d

MKVLIVLLLGLAFVLADHHHHHHDYVVKTHEDLTNYRTQCVEKVHASEELVEKYKKWQYPDDAVTHCYLECIFQKFGFYDTEHGFDVHKIHIQLAGPGVEVHESDEVHQKIAHCAETHSKEGDSCSKAYHAGMCFMNSNLQLVQHSVKV

>AaegOBP1

MNGSVVFVLSALVSLSVGDVTPRRDAEYPPPEFLEAMKPLREICIKKTGVTEEAIIEFSDGKVHEDENLKCYMNCLFHEAKVVDDTGHVHLEKLHDALPDSMHDIALHMGKRCLYPEGENLCEKAFWLHKCWKESDPKHYFLI

>AaegOBP2

MMEQLMLAVLLAVFLGLVADVTMAAQIKDNLELPEYYKRPAKILHNICLAESGAMESKLKQCMDGVLHDDREVKCYIHCLFDKVDVIDEATGQILLDRLAPLAPDNDVKDVFNHLTKECGHIKLQDSCDTAYEVAKCYFAAHDQVVKFCHLLMADVTS

>AaegOBP3

MIRFIVFVSSCLVAVSIADVTPRRDAEYPPPELLQALKPLRDICQKKTGVSDEAILEFSDGKVHEDEKLKCYMNCLFHEAKVVDDTGHVHLEKLHDALPDSMRDIAMHMGKRCLYPEGENLCEKAFWLHKCWKESDPKHYFLI

>AaegOBP4

MGLHKVKLLFHVLLAVMLSLHTSESKSTMEQLAKASEMMRGVCVGKTKAPLDLIDGLGRGEFVENKDLKCYANCVLEMMQAMRKGKVNADSAIKQVDLLIPPEIGEPTKKAFDMCRNSADGIKNNCEAAWALVKCLHQKNPKYFFA

>AaegOBP5

MFQKFWILLAFVGPALTHSDDYYDSNRRSRGYNERCYQHEQFPEPSECCTRPLWINQYLVRPCRFSNVQRDGYRQEHEACSVSCGVYRINMEMLNNQVNSVRIFRPARLRAYGDEDWKRTVAAALKLCKKRITSMVGSRAREGREAELCEEANDVFADCLDGQLFLQCPARVFIRTEGCELAKSHLLDGCPYRSLTDTTERHRNDWYDRNQWNGASNGGYDQRNGDQYDDEYEDVNSGHQTRNGGGYNNQNQNGYDRHNNDRNRNNGQW

>AaegOBP6

MKRLASLLLLFLTVRAEVSTQHCAVAKLPCTLHSECLQYLNSNDDGPENCAYRCIALTARFWDDQKADVIRTISRFYLTDANDDDFRNRTEQCLQETQETFPVTESCQRASCAFSCYNDQFGEVIAVRPSFIPFTALEHRRIVRECVDILQIGPQSRQAILDEGLMEVPEGRCLLRCVLLREGLYNDWRGPRLGSLWVQTEGYEDRFFDTAQKCYPLLKMQTLEPCELAARFAAECLPSRVPFVETVFAALASNQ

>AaegOBP7

MILHLRILFAVIALVSFCSCNQHKIVQKSLGKANDECVSYGSSEQCLARCVTLVTRDWNETVGLSSVYDRFYQPDPEDLCNTNRTQRCLEALQSTVAPEDKCLRAAGSVQCYLDQYGQVDMATSRFVKSAPVQQQQIIWECGAMLGYSGDQILRSIDDKDYSMQETRCLYRCYLIRSGMYTDEGGLNMERFYVACGGYEDEFYRNVTECAARVRSSTRCDDRCTLAQRLASECIGTRYDQTLTPGPATIDARDGSSVTYAVFQNYAGRDMTNTFVLNQR

>AaegOBP8

MKCLVLISLLAVGSQAFFTPEQHEVAKRLTMACATEIGEGLPDNVGNRFREGDLTLTDDKSKCFMKCVFGKVGFIDDAGTVNKEVLVEKLSKGNTQAKAEMFAEKCNMFEGANGCEKAHGLFECYWKNKEIFA

>AaegOBP9

MLKLVVALLSVTIALNQIKAFTLQQRQQGDIYAIECIAETGVNPASVALLRVGDFSSNDKRSKCFIRCFFEKEGFMDSKGNLHTEKIADALAGDFNREKVETVLANCLTKEKTACETAFRMYECFYNHREGL

>AaegOBP10

MTSFRLANLTVFLVLLFCFMRGVHSADDLSKIPEIKGYELHCIEASGITESSAKKLRNGDDIASPDQSIKCYVQCFFSKLRLMNEKGVVQKDKVLSLLGKLMEEDKAKKLAEKCDLRRTNPCDTAYAMYDCYRQNKAKLL

>AaegOBP11

MRLLISIVSFALVGAALSVPQQANLEDIGKIRNGETYALECLLASGLDVSSLKSLQTGDFSNGDRVKCLVKCFFEKTGFMDAEGNLNEEAIVTQLSQFMPKDQVETLVKNCKIEGTDACDTAYQATECYFKNKAGLF

>AaegOBP12

MKSFVCVVLVAALIGVNALTEEQLKKADEFASECLEKSNGLSKETVGKLRSGDFANVDQDSKCFVKCFLERAGFMSTDGNLVADYAIERLSLDREKSKVEALVMKCSMQMDDPCETAFRAFECYYNGKASLL

>AaegOBP13

MKTFAAIVSFALIAGCMAVTEDQKEAARQLAGKCMQQTGTSEESVQRLRNGDTSGADDNTKCFVQCFFQGAGVVDGEGNMQEAFVTEKLASEYGQAKAEEVVQRCRNNSGANACERSFSLLQCYIANRASLM

>AaegOBP14

MKTIAAIASFALIVGCMAVTEEQKEAARQLAGKCMQQTGTSEESVQRLRNGDTSGADDNTKCFVQCFFQGAGVVDGEGNMQEAFVTEKLASEYGQAKAEEVVQRCRNNSGANACERSFSLLQCYIANRASLM*

>AaegOBP15

MKSLVASGVALLMLASSIVPGCAQDFKGAIDECTKEFEMDMDVVVSLKYGDFSERDPLIECFTECLMKRSGFMFDDFSYNKTLIIGFAGRYLEPEGAQYVYDNCVDKFGTTVCVTGFEMYQCIHETAVAEWVESNF

>AaegOBP16

MIVIVVLVALSSEVLGAGQHDAVFKSIESTGKECARYLNNDGTGDCNTHCVGVIDHVWNDTVAMFTRNYERFFVPAPEDLCYQNRTQRCLSQVDQVVPVSDKCARARQLGQCYADQYGQLNASQLQYRPMTNLQYNRVFQQCSSMLGLSNDVLKDIATKGVDSVPAFACLVRCTMIRMGLYSDNEGFDLTLATGQCGKYNPALDPVPCQAKVKAEECDRCKRTVRIANECLGLRLNVKQLEENQPPQLVFNIEVYDFNCIILCDFEINF

>AaegOBP17

MKSIASAVLLLIFSVLVHSQSIKDLVEECKQTVPISEELEKSFLKLEFPPEEKTTHCLLDCIGKSLKVMDEKSGINLAVVTKLLQEVEPEGVIGEEQVRCATEAATSKEDQCTMAFKLYQCFEKEFLALMKMKLDQGE

>AaegOBP18

MTTHRLFIAATLLVLLVSLAYASEVLTKRQQYDQHKLMCGKIVRSTKEDRELYSQSQYPETHDTACFLRCVSILSGSYDDETGVNLDVLYDVYGKGTTAEEYAEESKACLALRDEVECYCMKAYKPLMCLREQFKKRNTA

>AaegOBP19

MLKAMTSNRCWIAVAIVCLMGVAAQAGPDFRTKREQYDHSKQMCGKILRTPAADLEHYLRSDYPESHDTACFIRCVSILNGGYDDETGVNMGVLFETYGGSLTKEEYADEAKECLALRDEVECYCMKAYKPILCLKEQFKKRNVL

>AaegOBP20

MKPGVKLSLLLLIGLMALLDSTSGCSMTNNDGVEQREALLADPSTAPARSMKDYSVEDIYADCNKTFAISMDFLNELNDTGSFPDETDKTPMCFIRCFLQKGEIVTSDDKINKEQAVALGWVKNGETIDDCLQELTGNPCERAYFLMRCVSTRHLVEGRSKDSKKR

>AaegOBP21

MQIECIIVLTSLIAAASAGWRLQTVDDLLRNRNKCVKILNMQDDLQEEFGLFDFPDQDSAKCVFKCIMNRMGLFSDKRGPHVGRLVKQMKFASMSSTKAIRDEILNCAYQDMEMDPEDVCDRAYALYQCIQNSNLLQLKSPETVKT

>AaegOBP22

MKVFIAVFALIAVAAAEFTVSTTEDLQRYRTECVSSLNIPADYVEKFKKWEFPEDDTTMCYIKCVFNKMQLFDDTEGPLVDNLVHQLAHGRDAEEVRTEVLKCVDKNTDNNACHWAFRGFKCFQKNNLSLIKASIKKD

>AaegOBP23

MLKLVLCLSALGLVACYDFKDSFYNELVLEEILDSEDAPSLMDRFKRSNPEMMDDKCKRNHRHKCCNDANGENMDKFRETKKQCFNEVRSKDRSARGMMNPVDMFDCEKMNKTKQEYICAVECVGRKFDIIDKDGNLLTTDKLVKFTKDNFAADPWQETVVDGLVESCLKEVAEKNEKMKSSGEHTTCNPSSSNFGYCMWRQMTLACPKDKQDTSKKCERMREKFANNESFSMYHKHDFDDK

>AaegOBP24

MNKLILVAIVATVAIGTSQAFVPVFHRFRRSPSVRCCNDGFEDSINHEKVVAVRRTCAEELGLNEMSEEELLKNRENLVCLVECIAKKHELADETGDLLHEDLAKAVKEHFSVAEWKAPLLDDFIKQCFDHAEEEHEKHPTEGGKCNPEGFDFSYCLWRHFTLACPEELQDDSERCEAIRRKLKSDEDVGFWNNDFDETK

>AaegOBP25

MNKLTLVAIVATVAIGTSQAFVPVFHRFRRSPSVRCCNDGFEDSINHEKVIAIRRTCAEELGLNEMSEEELLKNRENLVCLVECIAKKHELADETGDLLHEDLAKAVKEHFSVAEWKAPLLDDFIKQCFDHAEEEHEKHPTEEGKCNPEGFDFSYCLWRHFTLACPEELQDDSERCEAIRGKLKSDEDVGFWNNDLDETK

>AaegOBP26

MADDMAYQHFKMCDKATKKSEPPVSSENQSHLSLPIRVDAKRLQRRDDDDADQLFANQLTGSAASQQVQVKLGTYKNDKQNSLIIHSGGNCRTSITPTTREIGESEKLCRMLKLVLCLSALGLVACYDFKDSFYNELVLEEILDSEDAPSLMDRFKRSNPEMMDDKCKRNHRHKCCNDANGENMDKFRETKKQCFNEVRSKDRSARGMMNPVDMFDCEKMNKTKQEYICAVECVGRKFDIIDKDGNLLTTDKLVKFTKDNFAADPWQETVVDGLVESCLKEVAEKNEKMKSSGEHTTCNPSSSNFGYCMWRQMTLACPKDKQDTSKKCERMREKFANNESFSMYHKHDFDDK

>AaegOBP27

MKTLSVIILGAWLVHLGGVMSSMTFEDMQETAKMMRGICQPKYGIPDDVAENASSGVFPDSREFKCYASCLMDLTHTAKRGKLNYEAAVKQITMLPDDFREPFRVGLDSCRNAADGIDDYCEVAYTLLKCFFKASPKFFFP

>AaegOBP28

MKVFAGLLIAAIAASASAVYYPPLAPSDVEESHFAYQLKSFRQELDECAEYLQVSPGSVENLVAYNYVTDDPSLKCLIRCAGINAGWWSVGGNNSGLQPPVIESYFAPGCDDTCYVKRTQDCVSANVVPCQDDCSQAYQAFLCYYHQYGNLKSSEEYIPLPQLDAVQAAIDCMLILRTPKELLEQYVQGVFPDVPETQCLYRCQYLAEGLYDGVTFNLTRNYIREYAVPSPQIKDPATQACVDSALSSSSCNECARFWAGRGCLKNYGVPNHSGSYFQVAAGLVLNQRTCLDEDLNPHIFTCCSDVHGSCKAACESNLSKKL

>AaegOBP29

QQDAVQAAIDCILTLRIPKELLEQYAKGVVPHLKSFRQELDECAEYLQVSPGSVENLVAYNYVTDDPSLKCLIRCAGINAGWWSVGGNNSGLQPPVIESYFAPGCDDTCYVKRTQDCVSANVVPCQDDCSQAYQAFLCYYHQYGNLKSSEEYIPLPQLDAVQAAIDCMLILRTPKELLEQYVQGVFPDVPETQCLYRCQYLAEGLYDGVTFNLTRNYIREYAVPSPQIKDPATQACVDSALSSSSCNECARFWAGRGCLKNYGVPNHSGSYFQVAAGLVLNQRTCL

>AaegOBP31

MGVLIGLFVTAIAATASAVYYPPMAPLDVEESHFAYQLKSFRQQLDECAEYLQISPGSVENLVAYNYVTDDPSLKCLIRCAGINAGWWSVGGNSSGLQPPVIESYFAPACDDTCYVKRTQDCVSANVAPCQDDCSQAYQAFLCYYHQYGNLKSSEEYIPLPQLDAVQAAIDCMLILRIPKELLEQYAQGIVIKGPETLCLYRCQYLAEGLYDGAAFNLTRVYIREYPVPAPQIKDPATQACVDAALAAPHCNECVRFEAGHACFDAYGVPNHTTPIFQVAAGLVLAQRTCLDEDLNPRYNAGGSAPQPTPAPTPAPTAAPTPAGCVYNCGA

>AaegOBP32

MSVLICLFITAIAATASAVYYPPLTPSNVEESNFAYQLKSFRQQLDECAEYLQISAGSVENLVAYNYVTDDPSLKCLIRCAGINGGWWSVGGNNSGLQAPVIESYFAPGCDDTCYVKRTQDCISANVVPCQDDCSKAYQTFLCYYHQYGNLKSSEEYIPLPPLDAVQAAVDCMLILRIPKELLEQYAQGVFPEVPETQCLYRCQYLAEGIYDGVTFNLTRDYIREYTVPSPQIKDPATQACVDNALASSSCNECARFWAGLACFRDYGVPNRSVGSFQVAAGLVLGQRTCLDEDLNPRYNAEGPAPPAPTSAPTSASTPAPTPAPTPAGCMYNCGS

>AaegOBP33

MSVLTCLFITAIAASASAVYYPPLAPSDVEESNFAYQLKSFRQQLDECAEYLQVSPGSVENLVAYNYVTDDPNLKCLIRCAGINGGWWSVGGNNSGLQPPVIESYFAPACDDTCYAKRTQDCLSANVAPCQDDCTQAYQSFLCYYHQYGNLKSSEEYIPLPQLDAVQAAIDCMLILRTPKELLEQYVQGVFPDVPETQCLFRCQYLAEGLYDGVTFNLTRDYIREYAVPSPQIKDPATQACVDSALSSSSCNECARFWAGVDCLKNYGVPNLSTSYFHVAAGLVLNQRTCLDEDLNPPPAPAPTPGCMYNCGS

>AaegOBP34

MIKIRIVTLLVAVLLLETLRPSDAAMTMKQIKESMETMRKACAPKFDVPETTLNDLKAGNFRPDASKDEKCYAKCIAQMAGTLTKKGEISFSKTTAQIEALLPTELKAPAKEALKACKEVHTDYKDSCDKVYYSVKCAADFNRDVFIFP

>AaegOBP35

MKFFVAIAVVALAAGAWALTIDQQKKAEAYAAECVKSTGVAPDTPVKLKKGEFAGADDKTKCFSKCVLEKAGFMNEKGEIQEKTVIDKLSVDHDKAKVEATLKKCNQKGANACDTAFKMTECFYNTKAGLV

>AaegOBP36

MVRPCLYYCCILIAIFCWVQSWLVGAAPQKAGEFSRSMGIEMTASQHGECVTETGVSEESIARFNGPEIFEDDDKLKCYMDCMFRKFGATKPDGEVDMIEVYHKIPKDFNSVALIVNNKCRDAIQGANQCERAFSHHKCWKQMAPEVNHVHS

>AaegOBP37

MYRKTLLAFFFFLFLSCGDAVQNLTALRGSDYPPMYLINLVKSALERCHQLIDIEDSVIVRFRDDGDYEGTEQLGCYLHCVFREKGYWIPEKSEVDIMKILDIVPKDFEQPALKMGLRCLKVKGDDDCSRSLWYHSCWKKNDPAKVES

>AaegOBP38

MVTTLLLLFLVGVINCQEPRRDANYPPPELLEKMKPMHDACVAETGASEDAIKRFSDQEIHEDDNLKCYMNCLFHKAGVVNDNGEFHYVKIQDFLPESMHLITLNWFKRCLYPQGDNLCEKAFWLNKCWKERDPVHYFLP

>AaegOBP39

MYVVNLVLVLLSLEILSTSDAAMTMKQLKNSLEMMRKACAPKFNVVEASLDELKAGRFANEADKELKCYTMCIAQMAGTLTKKGELSLSKTTAQIEAMLPQEIKAAAKEALNACKDIQSGFKDPCEKVYFSAKCAAEYNPDVFFFP

>AaegOBP40

MTRLVSSIICVWASLLLSISAQYLQNEALLQAQATCVEYLGIPEARLEQYNISVYPPDRDTMCMIRCAGIVLGFWEDEQGLLIDGAKQLFPDSGDVDLVAQKVLHCAERKLLSCDPADACARAYYSFRCAMRKFEPSNSALSTDQKLTPEKFLKAQIVCANILRIPHDHLKLYNQGVYPDDAETRCLLRCIGVRLELYSDAMGPNLDRLHSEFAIDQPLEEFKTRATLCCEANRPLIQDYCTAAYRNLYLCFREHFNAFTSQNRQTLLSHTSSPQTCIDLESDILLYGDDV

>AaegOBP41

MKHLVSFVLLALAIYPVHSARRYRIAAEECVQYLKICPTRLEQYLKFIFPEDRETMCFMRCVATKLNLWCDRKGLNWAVLEDRICPSVREKVEACVCRKLDLIDPYDHCPRAYYAFRCLRNYLQEIFLFKNLDRDFDDGYIVKVPSSKELIVPSCASCSNSFNPLTITEMTQKLLQCAKKCQLCSLNLCDRTTDPVVETPEFQCTVYCASICTGVYSEQKGILMDNLYAQLARCETRESFDYRLGLCFGRNALPEGSSPQAVVFQQYFKCLRGDYERFYSSNLEELLQIPGISKYCF

>AaegOBP42

MECCNTPMLLDKDIMMDCYQKYGDQTKKQMKLEGVPRGCCIAECGLNATGLYSNGMIKRDDMTKMFMDSVKDMPEWQMLVRDTLDECFKMAESKMDEIQAGAMLEPSFEGEKICHPISGTILRCMGMNLFVKCPAGVYNESDECNQLKEYSKMCPIM

>AaegOBP43

MKIVIATCFLVGLLLRFLVAGDDDVCKNGSPTNKSLWDCCNMPNLVNQDIRADCHQKYGEQTMKQMKLEGTPRGCCIAECQLNATGLYADGMIKRDEMTTMFMDSVKDTPEWQPMVRDLLDECFRQAEANKDIIAAGAMLEPSFEGEKICHPISGAIMRCMNKNLFLMCPKESFTEGPECTQLMDYFKMCNGI

>AaegOBP44

MESKTFHFLLPLLCTLASYTEALDHAAILKSPNELQLECSKYLPSIDVSRNVDCTDRCIGLVGRFWNDSIGRPAQTIARYYQPDTGSQDYITRTDQCLCEKVLTVPRNAYCQRASSGLQCYRDNYGQLLTGTPQFVPVTEIRAAQIFWDCAQMLQISRDRLTQIFKDGYNKTSEGRCLIRCFLVRAGLYSDCQGPNIGRFAVQCEGYSAEYEQAAVMTG

>AaegOBP45

MELKTCCIILPLLCTLTSYAEALDHAAILKSPNELQLECSKYLPSIDVSQNVDCYDRCIGLVGRFWNDSIGRPAQTIARYYQPDTGSQDHITRTDQCLCEKVLTVPRNAYCQRASSGLQCYLDNYGQLLTGTPQFVPVTEIRAAQIFWDCAQMLQISRDRLTQIFKDGYNKTSEGRCLIRCFLVRAGLYSDRQGPNIGRFAVQCEGYTAEYEQAVVSCYDRLKKESLDSCSLATRTMDECIQGNQFSSSDIDGLEKLEVQ

>AaegOBP46

MSGSISLIVLAVVALAGQVLGRHDATFKSFGSTSGECSRYLNNDGNGECNIHCVGVIGHAWNETLAKFTQNYAGYFVPDPQDDCYQNRTERCLLQVDNAIPVYDKCTRASKLGQCYADQYGQLNAIQPQYVPMTDLQYTRVFLQCAAILGLSNNDLNAMVQQGAYNTPAGACLLRCTLIRMGLYTDDAGIDVALATRQCGLYNATSDIAQCQAKVQAEECDKCKRTTRIAKECLNMHYNVRNVGDSYGLELYGVDTCYSSCSFFYCYYYACPYLSYYNTNYAGSSSYSGSSNSLTFAG

>AaegOBP47

MHLSALFFTVVSFLGSFCFAINPCIEGPPVNKSPSECCTTPALIDPPLMMKCFQKWGEQTKRQSKMDGIPRGCCVADCAMEGTKLISKGKFNREKARKVFMAVVKDQPQWQPIVNETLDACFKQADENMAEIEAGAKLKPSYKGEKICHPISGSILRCMNMKLFSKCPNDLFNSGPECDQLKLYHEKCPLN

>AaegOBP48

MKATVTSVLVLLAISHATLADPAAPDNVPASCLNKNFNVDPFECCKTPKLLDEGTVKECVHSFPPPQNAQDEIKPDCMSECVMNSTRIFDRRQNVNDAKAMETFLEKLNGKSVWAEIVQKAVKQCLDDADNRKEEFSRDMKALQQKFPKERICSPAAGFIMECVHVSVYKNCPASIFKDNLAGCPAIKKHLNVDNCPFYTIFPEKKAPKPVKRH

>AaegOBP49

MIYSQPPPDDKACFQGYKVDANGCCELPRFVAREINAKCDEEFKPLSPRLPPEVQAYEGSCVIECLFNVTGMFKDGKLQQDKIAQQLKKTIGADRNFAPLLGGVVTDCYRLVMDNPANSFKPIPVKPGRPGCSFIPQAYMNCVKSELFENCPKANWTAADGCDLLKQKLNVGCSYYSIMIGKKGLKS

>AaegOBP50

LLIRFNTNLKTQVVAQLPQEDISCYMGNVKIARECCLMPRFIDKTVDDACTAEHKNPGPRVPPWTAKTEGSCVVECVLTRIKSFSNNIIDKEATKLSFGKSIGIKTFFGAVTNRSVDLCHKRILNNTALQLANPVSHDSNRTACSFVPTVFLDCFKENVFMNCPKQKWINVPECNALRSKISSGCTFNAIKGYSNSTHF

>AaegOBP51

MLVHLLPTLIVTLLGIGTVVAQPRPDDPSCMEGNQRKAHDCCRMPMLVEQSVMNRCMTENPMTPPVPGVQRTEGCCIAHCVLTTLNAFRDNLIDAAAAKRALTQSMGANSSFVSLVSGVVDECVNLVHGNAAYKVAPVASTPGRPGCSFMPEGFVNCIKGRFFQQCPTAEWTRDAACDQLKQKLTAGCSFGSLMG

>AaegOBP52

MFKLGFLLILVSCLAISVQCVGFDPSCFQSSSSKKADDCCLLPKFYDSQMVSDCLTSISKSTNDVEKYQCLVECIAKKLNLFKGNTLDREATMQLYKARIGSVPHFAPIMDNIFQQCYDGMAVYAAQDRSDPTKCSALPMMLLNCIQTRLFQNCPAKLWQGGPECQELKEKLLEGCPYAAIASF

>AaegOBP53

MLNLWLMVLSFAVTTHNSTWDKSCFELKTSKRADDCCDIPGSFDEALLKRCYDEQKASKNEQEAIKCIAECVARELGAYKNHTLIRENSRLVFESTIGSDPNFRPVLGDVFEKCFNRITAIEAQETYKNATCHFAPAFMLNCVESGLFENCPVSIWNEGVGCDELKEKLEQGCPFFAISETL

>AaegOBP54

MFIDAYSILKLSCNDGPVDKSCFELRTTKRADDCCKIPDILVESDESMVRRCFAQQNKTLDEHETAKCAAECIARELGTFKNGALDKELAKKVLLGRLDKDKNFKPIVGGVLDKCLGRINAVIEKESKRNGTCNATANFLFDCAEQGLFENCPSSVWDSNDGCVELKNKLAQGCPYSAIAE

>AaegOBP55

MTKRMELVLFGLFAVVTLFQTGLGGVGVEGKATVEQMTKTGEMIRNVCIGKLKVAEDLVNMLGDKQFPDNKELKCYVNCIFEMMQVVKKGKLNYDAAMKQIDTIMPDELAEPMRIALNACRTASDGIKNNCDASYAIAQCVAKNNPKFVFP

>AaegOBP56

MEKTGKLFRQVCQPKHKLSDDILEAGKNGVFPDTKNFKCYISCLLDMMQVTKRGKISYEKSLKQIDQLLPDDMKPDFRKGLEACKDVASGIKDQCDSAFVLLNCFYENNPQFILP

>AaegOBP57

KFSSALSFCSLQLAWRFVTELQCANSDEEKKAQAKEMMRGMAEECKKKEGATDEDVEALLEDKTPETEVQKCFLSCFQHQFQISDGKRFNKDGFMQLSAMMFGEDQEKMATAEEIAEECSSVENADRCQLSVDIKECVEKAMDKRGIKMEK

>AaegOBP58

SSPFYHALQVCARDLSVPPDRFEQYRLLIFPDEPDTHCFVRCLLLGIRAWHDQTGVRHSALQQYFSPDDNPVDAYARVQTCLDYVSNSCAATESCTKAYWSLNCYKQQFGSYFFSREQFVPATDIQLAQAMFDCADKLDISRSIVAAYRNGNRSELISSRNPCYVRCVAISIDLYDDDAGLQWDHLYVQLGLNEHRENYLEQVHKVVSELQLSTMDRCAAAAQVIEPFLLTALQQSRTAYRTGIVVETTASITTTEVSVTKPMTTKTPSTTIQMTKASTSAPPTT

>AaegOBP59

MKPGVKLSLLLLIGLMALLDSTSGCSMTNNDGVEQREALLADPSTAPARSMKDYSVEDIYAECNKTFAISMDFLNELNDTGSFPDETDKTPMCFIRCFLQKGEIVTSDDKINKEQAVALGWVKSGETIDDCLQELTGNPCERAYFLMRCVSTRHLVEGRSKDSKKR

>AaegOBP60

MILLNMAVVLLEVMLTLAADKPIPRRDAEYPPPFVLEISKKPHKMCVASTGVSEAAIKRFSDEDIFEDDEKLKCYMQCLFEKLRYTDDKGELHLGKVMDSVPEEYEDIALKMGSKCLKPKGKTQCERAFWYHKCWKTSDPVVSICDYVFL

>AaegOBP61

MKTIAAIVSFALIVGCMAVTEEQKEAARQLAGKCMQQTGTSEESVQRLRNGDTSGADDNTKCFVQCFFQGAGVVDGEGNMQEAFVTEKLASEYGQAKAEEVVQRCRNNSGANACERSFSLLQCYIANRASLM

>AaegOBP62

MKIVIATCFLVGLLLRFLVAGEDDVCKNGSPTNKSLWDCCNMPNLVNQDIRADCHQKYGEQTMKQMKLEGTPRGCCIAECQLNATGLYADGMIKRDEMTTMFMDSVKDTPEWQPMVRDLLDECFREAEANKDIIAAGAMLEPSFEGEKICHPISGAIMRCMNKNLFLMCPKESFTEGPECTQLMDYFKMCNGI

>AaegOBP63

MECCNTPMLLDKDIMMDCYQKYGDQTKKQMKLEGVPRGCCIAECGLNATGLYSNGMIKRDDMTKMFMDSVKDMPEWQMLVRDTLDECFKMAESKMDEIQAGAMLEPSFEGEKICHPISGTILRCMGMNLFVKCPAGVYNESDECNQLKEYSKMCPIM

>AaegOBP65

MDTFNAIRNGDFSIRTPFIECFGDCLVKKAGFMNDDLSFNKDVIVKFASRFIKPEDAETVYSQCTADVAPVLCATAYDVYQCIYENALAKWGTRRNGK

>AaegOBP67

MNESLCRNNTVLARNCCRLPGIINQSIVDDCDDKFPHHAPVKRVEGSCVVDCMYKTIGAFQNGTLDLDITLQHISQTVGRYPNFEPLVNETVSWCYRNVTENPALQKSVGCSFIPQEMNDCVKKMLFMSCPPSNWTTKVECDDLKGKIAEGCSYSSLY

>AaegOBP68

MDIFIIGLMLASSVLGQPPAENKTCYQGNQKTAAECCPLPRMMEKSIADMCNSKYKALSPRVPPGVQKTEGSCVTQCIFTTIGGYNEKNNTLNIEAIRKAILTTTANAKAFLPLFNSSIDHCYPIISKDPQFLANPVSPIPEREGCSFLPPALMNCIKIDLFQGDVVSLDLSKAFDRAWRFPILKSFED

>AaegOBP69

MKMSYSHELLFVAMLSAVLHLSSAMDCKEVWERKHETADCCSAPAILNLDNLKSNIEGQEGNKHEKFFCGVHNLMKEQNLVDDEGNLDVDAMKQNTEGFDDEWKQIAQQAIDHCVQKTESMMADMEQRGGPKGQCQPTAGMFLMCLGKASIKNCPADKWNSSELCEKVKSGECDKRGHKH

>AaegOBP70

MIRVLLFLTFFVGATLSYDFKDPYFNDFLLEDLMVLQGRPALKKASDSEESQLQYTCCDYLNEENFSKLQQTQIVCYVENSLLSATKTKSGRAVSPVDMFSCDRLDKLKQQYICASDCVARKENITDDSGNLLGSEVLVPFVSQYYAPEVFQDEQIKEFVDTCLGESKTDETVANKCNPSSARFGYCMWRKTILSCPNERQDTSPACDNLRDKLLYQEAKYLSDETR

>AaegOBP71

MVRVLLFLTFVVGATLSYEFKDPYFNDLLLEDLMVLQGRPALTKASDSEESQLQYTCCDYLNEENFSKLQQTQIACYVENALLSATQMKSGRAVSPVDMFSCDRLDKLKQQYICASDCVARKENITDDSGNLLGSEVLIPFVSQYYAPEVFQDEQIKEFVQTCLDESKTDESVANKCNPSSARFGYCMWRKTILSCPTERQDTSPACDNLRDKLLYQEAKYLSDETR

>AaegOBP72

MNIIVFLAFLVLAVDTDKSPVDAECIDVEKNADEIRQCCDIPSPLEMENIQTCKEKYQEELGSDVPNLVACIFDCHARELGVLKDDLEIDEAKMMEYVSQTPDEDVKKLMVESAKECLKAKGEIMEKAKEHAMKCHPLAFMMTECIMHAVYSECDKLPNHWKDSEICSKVKNGAEPCE

>AaegOBP73

MQVLNFLCLVLLCLVLEKVAVAEECIKFEDHKDEILNCCKYQPPYPKDDVKECVQEAQGKSGGDKHEFFACLLECYLPKIGIINGDSIDEDKISEHLQSLDENARDILLAAYKECDESTTGTTRAQCSSYALDLETCVLQKLDQQCPDEFYNPSEICDKLKSGVEICH

>AaegOBP74

MKLNLALAALIGMVAMVHGQQQINQECFNRPNDKNPMECCRAPNIMPPREELITCMQKFPKPSGPPTPGSPPPGHNCMAECMLEQQGIMSGGALSKDTATSKLVALVGSSSEWQAVARKSIDTCYSQVSSLGGQKDSLGCSVIAGSFMECMPSMMFTNCPSSAWTASAECDQLKAHLQKGCPLMTLFKGPHPH

>AaegOBP75

MKLNFALLTVIGLFAMACSQQPISQECFTRPNEGNPKDCCKAPNVIPPKDQFAECMQKYPKPSEPPTPGSMPPNHNCLAQCMFEQQGIMADGAVSKDAAISKTVAVMGGSSEWEATTKNVVEACFQKVSALGAQKDSQGCSVMAGSFMDCMPSMMFTNCPSSAWTASTECEQMKAHLQKGCPIFTLWKGPPPH

>AaegOBP76

MEIFHVGPMHQNGCPGPISQCASQNSNVAKQIDDYRKQCVELSDVSVDSAIKVHSGQVIENPDWSTKRYVQCFFQKMQFMDENGVMLKDAVVEFFSRIQDESRAKAMVENCDIQKENPLDTAYAVLVCYQGNKN

>AaegOBP77

MKHSGAIACCLLIAIVAVNAWPSYKRAEVRAHVRNCVKKTGIPGKNALKVLKGNFNDDSSEVKKFMKCMFQEVGFINEKDELLDNLLIAKIKENLEEDEADELIEKCSIVGDDINDTAFQIYKCYYENHDLPPDMLVR

>AaegOBP78

MKILEVVVFLTVVALCKADYSDKQKQKLDEFTSKCIEDLDLPKDSDLGKKFKYGQLKEKDDATKKFISCSMQKLSFMNETGSILEESIIEFLADKYDRTMAMNVITKCSKLKNESMEDKAAEFYDCFFMQKSFDI

>AaegOBP79

MKLSINLFVILMLTTLFVSTHQLGFKPFSAEKLRDIELICMKLLRQPIAFWYKYLNLEYPDDPITHCHLRCIGISTGLYGDEFGAHLDNIYEQFKENTLLNRTAWMEEKNNCLAKQFADGLPDDLCKRTFLTFKCFEVDYLLALSKSDCSKISI

>AaegOBP80

MKWSLKLLVLLTTLFIPSQQIIFAPHSAEEIRILEQGCVKLLLQPSVFWYKYLNLEYPDDPITHCHIRCLLIAAEFYDDELGAKLDNLFEQHQHDTPLDRTEWTEAKSICLARQFANGVPEDLCKRAYMTFKCFEIEFLISITRMDCNKISL

>AaegOBP81

MKAVRFLILLVIIGVFHTIPADAGQLLNKLITVCTQGQNPPADLVQRYRNGEFPNDRNTHCMMRCIALNLGVYDDLNGIHMHDTWQMFRRGRPASHEKAFAEQHRQCITQQTKDVPLDDYCGRVYAVYQCYKDEYEALLRNVRQGAAKARN

>AaegOBP82

MPPLLDENLLHGCKQLHGGEHLTRGLIHERGSCFIECAMNSTGTLVNGVLDQPKIVQLITTRTAGVSSDLTQVMVASCVKCFLTPLVMGNHSGHPLDSKHCRPAASIFVSCVNMEMFKMCLPEFWTNSDSCNNLRLHITNCPIPA

>AaegOBP83

MGNHWPSSHSILITVSIVFLFLLLEETWALKCRTEDGPSSDEIRKVIRVCMKRITSESENKSNNEYENYDSSYSDSNSDEDRESSTEGNTRRQTNGGNQSTNTRGRNGEDMSRGRDSSRSSDDRSRGDNRNRQDNGRRRDRERDYDYGPMGRRMDDGRNQQGRYKRQYYNDGAQGGYGYNYQQNDRYNRDRNQFMHPNGNTSSNGTNNTERDRACMMQCFFQEMKMTNNEGFPDKHKVLHVVTKDLRDYELRDFYTDSIQECFHMISMDNKLKDKCDYSMKFVTCLADRGQANCNDWENEAIMF

>AaegOBP84

MRRFKLASFILTLFATNVICSRHKIVQKSLAGTGVECQQYDPPWNCAVRCQTLLTRDWVDSTGMQSPYDRFFQPDPNDQCYMNRTQRCLLDKLSTVPRNKLCLRADSSVQCFLNQSGQVIMDQPKFVAPSRLLENQIFLECGTMLGFSRQRVWEVLYKGEFTLPEISCLVRCFLIRSGLYDDKSGLNLERFYVACGGYDDAFYHNVTKCIANVEAAGLCDKCTRAQRLALECVGSQYPIFVPVSQTDIDSTNNAGRDVNNYYTSNFNFNFGDVISQIGTMVPATGGG

>AaegOBP85

MSCLNLATLILAFFATSVICTRHKIVQKSLAGTDIECRQYDPPWNCAVRCQTLLMRDWVDSTGMQSPYDRFFQPDSNDRCNTNRTQRCLLDKLSTLPRNKLCLRADISVQCFLNQSGQVIMDQPKFVAPSRLLENQIFLECGTMLGFSRQRVWDVLYKGDFTHPEISCLVRCFLIRSGLYDDKSGLHLERFYVACGGYDDAFYHNVTKCIANVEAAGLCDKCTRAQRLALECVGSQYPIFVPIPLTGKLKLHG

>AaegOBP86

MTIFNALLAILACFSLPTDALQHNAVYKSINSAGPECRTILTRQSPLDCRLRCLSINTGDWDDCSGVPRTYDRFYVQDPTDVGYQQRTQQCIANVSVSILRGDICAFSARSTECYDANYYDIVLDQLVFVPSKSLQYQQTIRDCAGMLGFTEHVISDVLRDDCFALQETRCLLRCLLVREGLYGDQCGAQIDRLYVVTGGFDQLFRRDVKKCTGRLRAMGLDKCTEAYRVASECFPEDKAILPIFLKNKAILQEI

>AaegOBP87

MMSSGAFILSVVLSVSISVLQTSSLQHSATLKSFNEILSECSRYLPSNDEPCYDRCLGLVGRFWNDTIARPSVSVGRFYRPDPCDQNYVNRTQQCICDSVLPLPRKDVCLRASRGLQCYRNQYGRLIADEPLFVSVTPLQSSQIFQDCAQMLQIPRAKLEEIVQQGYSKSPEGSCLVRCYLVRAGLYSDSQGPDIARFAVQCEGYEDAYEASVARCYQKLKSEQLDKCTLAARTYDECIQANEYSNSNLEILGVLLGIITGLIPA

>AaegOBP88

MQVFAAIVKLVAITLGAIIASISCIEEHSASLKSILSSTAECNLYLPTEALRQECGTRCVSLVNRIWNDTNGRLSDTIGRFYVEGPQDPCARNRTLQCLEQVTASIPIRNSCKLADASVNCYRNNYGQLDVKSPRFVAFSDVQQVRILTECAAMLGVSDKLVQVVRNGLQSIPEGACLLRCLLIRQGLYSDQRGPDLKRVSVQCGGYEGYEQEWRANVTRCVAAVHAERICDKCLQAERIAVDCLQMHLHLYEVRSPKLRQHIPFGVEFYTRANAAAGSAAAAQARVITYITVEYYYWY

>AaegOBP89

MINQLLITLTTVNILTTSAVEDWRSPQLKSFSSAQQDCAVYLLLSNETVQQYVKSGYPDEFSSRKLINCILVQIHAFDELTGIKDHVLTNFFDQPGSCSEYVGRTQECLRTSVPKHCEGQPFEHAYRSFQCYYRNYGSLLMDTVRFIPYEQVDRIKHLTESFSIVNTSCKALRELSVGQGFIVENIADPMYTLAVRSGFYDREHGLYLDRLYTQFGKPALLSDATRQCLVRVSQQYQTEPLRLTQLVLQCVESEISTQSLFTETARQVLASNSSYCNVCEQLPSCVTMTPGVTSAAVTTTRAPLSTSTRPPYPSI

>AaegOBP90

MNAWDDETGIKDYVIRNYFKPADTDPSYKSRTQCCLRDKVANLDRCALFERAYHSFMCYYQNYGNIVPEAQFIPWYQVDREKHLREVFLIEGITRVQLEEFQRSDALKAKEYPILYYIDFVRTAFYDPSTGHNLERLYTQFGNPGLLADETRRCLDAVSLQYCDEPVRAYQGFDQCLRNYMTTEELFKTVVAQVLASNIVCR

>AaegOBP91

METRSIFIAVILSITSYVKAEDYEAPRLKTLATIEQECAGYLLLSNETLRSYIAASFPKDSTVQKLVHCFLVNMNAWDDETGIKDYVIRNYFKPSDTDSSYESRTQCCLRDKVSNLDRCAVFERAYHSFICYYQNYGNLVPEAQFIPWYQVDREKNLREVFLIEGITRAQLSKFQKSEERNPKEYPILYYMDVIRNAFYDPSTGHDLGRLYTQYGIQELLADETRQCLDTVSRNFFEEPTRAYQGYDQCLRKYLTCMWKNCCRLLLRRFWNQI

>AaegOBP92

MRCCLILLVSLVALHSSLALNHLEKLRWKTFREAELESAEYLFITHETLERYRSSGYPDEPSVRKLIGAIMVVLNAADEKLNLIKDYVLSQYFLPNTVDCQYKQHTKECLDRNVATLDPSDRLGRAYQTFQCYYKNFGGIKEDVGWVPYHYSEVVQMLEDCLYITNASNESLLQYCQGGYATSADYSNVAYCYAVRAGLLDKTTGFNVEKMYIQLGDDNLNDGDAKKCIAGVVNQYCKEPYRTMRIVVDCVLIYLPGVAGIVIAASNILGNPPECVIPPSPPPITQPCYNGRCL

>AaegOBP93

MSYQLLTPLVTFIMASSLAAEDWKSPELKSFSSAQQECAVYLLLSNQTVQRYVKNGYPDEFSCRNDSVSRTQECLRTTVPKHCEGQPFERAYRSFQCYYRNYGNLLKDTVRFIPYEQVDRVQHLKESFSIANTSCAALKDFCEGHGFNVAELAEALYVLGVRTGFYDPQHGPYIDRLYTQFGSPNLLSEATRQCVNRVSQQYSTEPVLITQLFLQCVEDDISTEALFTETAKEILASNQSFCNVCETLTSSVSSSTTEMVTTTTATISTSGAPLLTTTKGPYPYRSM

>AaegOBP94

MNHQLFITLAILSIITSLAAQDWNSPQLKSFSSAQQECAVYLLLSNETVQQCEVNGYPDDFNCRKLVNCILVQIYAYDERIGIRDNVITNFFEPPKSCSDYVSRTQECLQTTVPKQCGGQPFERAYLSFQCYYRNYGVLLKDAVRFLPYDPSSPKYSVKQIVESFSIANTSCKAIRNLSEGRGFTVENVADALYAFGIRNGFYDLQHGLYVDRLYTQVGVPNLLSEATRQCLACVSQQYNTEPLRITQLVLQCVEKDIATQLWFTQTAQAILASNNSYCNVCEPLRSCVDPTPSTQCGVSLVTTPKNPYPSI

>AaegOBP95

MRCCLIFMLPLVALQSSLALEHLETSRWKSFREAELESAEYLFITNETLERYRSNGYPDEPSVRKLIGAIMVVLNAAFEKLNLIKDYVISKYFIPNTVDCLYKQHTKECLDRNVATLDPSDRLGRAYQTFQCYYKNYGGIKVDVDWVPYHYSEVVQIVEDCLYITNASNESLHQYCRGEYATNAGYQNVVYCYFVRNGFYDKSTGFNVQRIYNQLGANNLIDDGTEKCITQVVNHHCKEPFRSMRVFLDCVVRYVPSSAAITEAASNILGNPPECVVPPSPPPKTQPCYNELCP

>AaegOBP96

MFRIGLLFVSFAVVSITAVDRHKIVYKSLQEAAVECGQYTIKGQCLGRCETLITGDWNDTTGMSPAYSRFFHPDPVDECNLNRTQRCLQTKVYTVPRPRTCQRASESIQCYLDQFGQVNLTAPQFVRFTPLQDDQIVLECAAIMGYTYEQVYAWIRESAFQRPETRCIYRCFLIRSGLYSDSEGLNMARFYVLCGGYEEDFYQRVEQCAARLRQEVPCNDKCTLAQRLAIECIGADYQAGNLATNANSKAVEGSRVQNINANPVNSVIDATNSETGNVITITRTNSDTYVYGDENTFENYFYESA

>AaegOBP97

MFRIGLFFASFAVVSITAVDRHKIVYKSLQEAAVECGQYTIKGQCLGRCETLITRDWNDTTGMSPAYSRFFHPDPVDECNLNRTQRCLQTKVYTVPRPRTCQRASESIQCYLDQFGQVNLTAPQFVRFTPLQDDQIVLECAAIMGYTYEQVYAWIRESAFQRPETRCIYRCFLIRSGLYSDSEGLNMARFYVLCGGYEEDFYQRVEQCAARLRQEVPCNDKCTLAQRLAIECIGADYQAGNLATNVNSKAVEGSRVQSFD

>AaegOBP98

MRNTIICIVFTLCAISPSNVLGLDHYIGYKSFDTYFRECGEYFEVPNCTLDEYAVNAYPDEPEVRNLIHCTLVGSRSWHDGSGVIESVMANFFNPGPEDTCYADRTRECILSSQVPCDSNITLAYKAFQCYYRQYGNLNESSQYMPCTDRELQVLINTSIIMVNVPKDELVNYSNGVQLNQPHFAELLYVIFIRGGFYYTDDKTLALNNLYTQFGNPELQTPETQQCVNSATAAWDGKRQRDLVYAYFVNCLQKIVPWLQLIQQVATSLSVRPFTPLAKPYSTTMRIITTGLIFTLCAISASLAQNNLQHYVVYKNFDTYFRECGEYFEVPNCTLDEYVVNAYPDEPEVRKLIHCALVSFGGWEGGIGVVEYVMSNFFNPGPEDTCYADRTRECIQNSQQPCDSNVTLAYKAFQCYYRQYGNLNQCLQFIPNSARELQVLIEASIAAVNVPNDELVNYSNGVELDQPHFAELIYVIFLRGGYYYPGQGLLLKNLYTQFGHPELMTPETQQCVDAATAAWDGKNQRDLVYAYLVNCLQKITPWLQLIQQVATSLVTVPPPPCPPPATPCTTTTTTTTTTPPPPNFSP

>AaegOBP99

MMLTGFSSCTPQNGRNGITRGCQRDSLRWSWKKKTKFMWVSSTLLVLSVLCVSAVELPPPHYVTRISFYTALQECAEYFQISENLLQQYISSSYPDDPSVHKLVRCSLMLLGCWDDITGMRRNVIENFFEIDPNDRDHVRRTNECIRKSTTEDVSSPAYVAFLCYHQQFGNFKLHSKRFVPFGSHELKQLVEMALNVAELPWFVPAQYATNDILYEPHFPPVLYFIFVRGGFYNAKIGFDLRNLFTQFGVEELLKADVEQCLANVVHTEKVNGHESIVIKGFQKCLAHFIPLLEVVQDVARSTSNDRSASVKACTGLNPSTQPPFYNRACED

>AaegOBP100

MGLKKWCIILPYLCSFAICVSALEHVATLKSFDEIRYECSQYLPSSEDEDCSLRCLGLVGRFWNDTIGTPSNSVGRFYRPDSCDQCYLNRTEQCLRRTVLNLPRSAVCQRASNGLLCYKDQYGQLINRAPQFVPVVKLRAMRIFRECAQMLEIPFDKVDRIFKEGRNNTSEGRCLTRCFLIRAGLYSDSRGPDIGRFAVQCEGYSVEYERTLVQCYEGLKAQQLDSCTLATRVLDECIQNNKYSYSNMDDVVTIQITDFTKLQVMVDLGTLVVFFPSL

>AaegOBP101

MEETDSNLITWHRVYLHREYVHGIGSETRNSLFHLHNARDLVLVTTCRHGKSWRDLQDARCDEDNREYDRTVALKAPTMQLRSKCLVLPLLGLCIFANRAVTLQHTATLKSFDELRIECSRYLPPVDALNNVEDCSDRCLGLVGRFWNDSISRTVYSVSRFYQPDSCDQDNLDRTEQCLCETVQSLPRNASCQRASCSMQCYQDQFGELINQKPQFVPVSKLRSAQIMSDCAQVLQISQDTVRQILHDGYNNTCEGRCLVRCYLIRAGLYSDRRGPNIARFSVQCEGYADEYERSVTDCYAGLKAQQLDKCTLAARFYDECILSNEYSNSNMDVIAALGGSLYGVILTTVGVTGYLVTSIVAGLSAAGIP

>AaegOBP102

MGLKKWCIILPYLCSFAICVSALEHVATLKSFDEIRYECSQYLPSSEDEDCSLRCLGLVGRFWNDTIGTPSNSVGRFYRPDSCDQCYLNRTEQCLRRTVLNLPGSAVCQRASNGLLCYKDQYGQLINRAPQFVPVVKLRAMRIFRECAQMLEIPFDKVDRIFKEGRNNTSEGRCLTRCFLIRAGLYSDSRGPDIGRFAVQCEGYSVEYERTLVQCYEGLKAQQLDSCTLATRVLDECIQNNEYSFSDLFDLLEPYLDGVISIGDFRRLELMIQLDNLVIYYPSLPSSSLGI

>AaegOBP103

MQVFAAIVKLVAITLGAIIASISCIEEHSASLKSILSSSAECNLYLPTEALRQECGTRCVSLVNRIWNDTNGRLSDTIGRFYVEGPQDPCARNRTLQCLEQVTASIPLRNSCQLADASVNCYRNNYGQLDVKSPRFVAFSDVQQVRILTECAAMLGVWDKLTQVVRNGLQSISEGACLLRCLLIRQGLYSDQRGPDLKRVSVQCGGYEGYEQEWRANVTRCVAAVRAERICDKCLQAERIAVDCLQMHLHLYEVRSPKLRQHIPFGVEFYTGANAAAGSAAAAQAQVTTYITVYYYYWY

>AaegOBP104

MQLFLTLLIFTLCTSAYAFLDHYVGHKRFDTIFRECGVYFQVPNCILDEYVANAFPDEPEVRNLIHCTLVGSKSWHDGSGVVEHVISNFFNPGPEDTCYADRTRDCIRNSRVPGGNNVTLAYKAFHCYYRQYGNLNHSEQFMPCSPQELQVLIKTSIAIVNVSQAELVNYSNGAVLDQPNFAELIYVIILRGGFYFTGQGLFLANLHTQFGNPELLTPETQQCVDAATAAWNGQRQKDLVHAYFVNCLRRITPWMQLIQDVATGLVRGSNAPCSTSSTTTSTTPSAVQPCYNVGN

>AaegOBP105

MWISAACLILALSFTSTSHVLGLDHYFSYKEFDSYFHECGEYFEVPNCTLDEYTANAFPDDPEVRRLIHCTMVIFKGWQDGLGVVESVMSDFFNPAPEDTCYADRTRDCIQNSQAPCDSNSTLAYKAFQCYFRQYGNLNQSRQFMPYTLREEQVLIETAIAIVNVPKDELVNYSNGILLDQPHFADVIYVVFVRGGFYDVVQGLSLDNLYTQCGKPELLTAETQQCVDAATSAWDGKSRKDLVYAYFVNCLQNVITFAQRIQEVATYLVAVPPSPCPPAPSTPCPTTTTTTTTTPPPPSTVPPCYNVRN

>AaegOBP106

MISIVSSEDAQPHLFLITQSIRSFPSALAECAQYYELSNCSLNRIVQESYPNEPDVRRLIRCALINVRSWNDTTGVQEQVMNSYFNPTPEDTCYLNRTRDCIERSRQLPGGDRDVQTRAYDAFICYYRQYGNLNETEQFLPFTDEESDQLMISVLSITEVSQEALVQFSEGNILDNKEFPAVLYTLYVRVGFYQDRIVPQHLYIQFGNPELLSPQTEQCIEAAVNSLPCEADDKDQVYRIFRNCLVGITRTLELTQSVSRQLLGLEPFCGNGDSGSSTNAPCAITASPASTSQAPYYNTVPR

>AaegOBP107

SIELSNRPSTIMNRIVLLVLISLCSASTVLADGLPHYIAENSFDISLRVCAEYFLVSNETIDGYYQQGFPEIEEVKQLLRCAMINLGAYDDTFGPLEYVLGNVFKPCPSDTEYAERTRSCVKKALDSICPSDVFSRAYASFMCYYRGYGNLITDEFFIPNSLLELTQMMLFVQSSLNLPDEVLVQYSQGNILNEPNFPNVLYVWAVRGGYFSVDEGIQLENLYIQYGIPGLLSQETRQCAADVAQANCNLDLVTLLYNMYVTCLRPLLPFESFVQTFAVEQLKCKTCGAVQPAKPSYTY

>AaegOBP108

MGLATTSIALLVLSLAAVGRCDLPQYSVYKSLFTALYECGEYLQVDNVTLDQYIYYGYPSIPEVKRLIHCAMVNVGAWNDNIGVRPNVFRYFFKPNELDTEYEERTQQCLAQICPNEYDQNYRAFETFSCYYRQYGRLVKEDVFNPLETLEFLQLLQFIKLVLNIPNEKVVQFAAGDYLNDPLFKQALYIGVVRIGALSRDKGFLPDVGYAQYGYPQLISPCVQKCIADVAAQYMNADKRELVYQVYVQCWYSFLDPFLRSQFQAALDGSLCDVQVKY

>AaegOBP109

MGLATTSIALLVLSLAAVGRCDLPQYSVYKSLFTALYECGEYLQVDNVTLDQYIYYGYPSIPEVKRLIHCAMVNVGAWNDNIGVRPNVFRYFFKPNELDTEYEERTQQCLAQICPNEYDQNYRAFETFSCYYRQYGRLVKEDVFNPLETLEFLQLLQFIKLVLNIPNEKVVQFAAGDYLNDPLFKQALYIGVVRIGALSRDKGFLPDVGYAQYGYPQLISPCVQKCIADVAAQYMNADKRELVYQVYVQCWYSFLDPFLRSQFQAALDGSLCDVQVKY

>AaegOBP110

MLSITFALVLFLSASSAVIVSPLDHAIETCGRDLQVCESKLASYRALSFPDDRETQCFIKCVLIELQAWSNPRRLLKHSTIQQYFIPDAADYSFEDRTRRCLDQTLPNCIPGDSCSRAYWTFLCYKDNYGNLIRQPNQFIPPTELDIAQHQLDCADILRIPREELLNAETLTNGSNCYARCILLRSEVYSDESGLDLDRLYVLLGYDTEKQAFIQYAQQFLATDSANCQTDRCLAAQVPYQLFHELLVKLFKRDGTRDISYVFDVNNV

>AaegOBP111

MSASAWIRVTFLLVVGLVAYCQAQDKYSQMYRGPVVDCREIFGYLNNLKEFFKDECGQLDEGCPWKLQKAIKWYGRVAKQIKQYVSATNRHSARFKSSRSTDSECYRYLNVLDGNCSVRCRGLVDRLWDDQSGLGLSITQFYKPDPEDKCYLNRTVRCLKGVSATESCSRVDKYVQCFNDQYGRKDTETARFIPFTTVQHTRILMECAAIQGIPVESLQRAAENGSGLPQEACLLRCFLIRQGLYSDAGGLDLERLEVQCGGYGSGWDPVAVRQCIAKVEDCDKCSKVQRIAKECLQAHFKVLPNPNSDTVESVPFLVEFYVGISVLGINFKGMISGCLLSIFCFFA

>AaegOBP112

MVQFVILLITHLAQLVTAADRHKIVYKSLQEANNECSLYNVPGGCLPRCVTQITRDWNDTVGMSPVYGRFFQPDPNDLCSNNRTERCLESKSSLISSKKTCLQASESVQCFMDHYGEINMTAPQFVRFTKLQDVQLIFECAAMLGYSSMEQLDALLRDSEFKRQETRCVFRCVMIRSGLYSDSEGLNMPRYYVLCGGYEDGFYQQAAECSARLRKEVPCDDKCTLAQRMANECIGVDYETSIMQSKGNTVNTIYAIQGSEVYNIDGQNANSNVALTSVQRDKTINIENTNSDLNKFGDTINVDNQP

>AaegOBP113

MFTVKYLLTLTSITIVSCSTSEHGFIFKRFHRGLLECAEILNIPKTTVQKSIEDQFRCNDQTKLLIHCVMVQLHTWSDGTGLRRSALVQFFEPTAYEALFEPRTDMCLTENLAYVDKCDFVTRAYVTFDCFYKQYGNLARNVHAVILNQKQLISALNVCFAIADIPQEAIQRLTVENVLEVPEAHCLLYIFSLRAGLYNEVGGVLMDSIYSQFGNRTLTQSGKITCVQHLLETSRFADRCSMLNAVYDRCLFDAIPINELIVEAAKHALANVGR

>AaegOBP114

MNFDLTLFSIVVLLAISTANATRSPSLKTIDQAVKECGTLWNVSPDYFEDFVRTGTGNSTQLKELVRCASIWCRWCNVSAHDVVYEVLQNYFNPSPDDPCFLNRTERCMKASLKDLPYTEVLERAFVSFLCYYQQYGNLNRSVQFIPYMLPQEQQVALDTLVIHSVPLETLRNFNDGVFKEGTFEFLLRTLLVRLNLYSDRAGPDVKRLYNQDGNESYLTPETAACIAEARKNCPSDRCKLVSNTLKNCLPQVYDDAVSLIKDAARMILQRMFCVQDLELNPILVERLVAKGAEEIFTNRHDCVI

>AgamOBP1

MKLVTFVFAALLCCSMTLGDTTPRRDAEYPPPELLEALKPLHDICLGKTGVTEEAIKKFSDEEIHEDEKLKCYMNCLFHEAKVVDDNGDVHLEKLHDSLPSSMHDIAMHMGKRCLYPEGETLCDKAFWLHKCWKQSDPKHYFLV

>AgamOBP2

MLAQASPLLLLLLLLVTQCLDGANCSTITTQRPAPRRDGQYPPPETLAFLRPLGKLCLEETGVSPEAIKRFSDADPFDDNRALKCYMDCMFRVTNVTDDRGELHMGKLLEHVPTEFEDIALRMGVRCTRPKGKDVCERAFWFHKCWKTSDPVHYYLV

>AgamOBP3

MGHDSCWSSRWRVLAALVIFQCAILMVRSDEPRRDANYPPPELLEKMKPMHDACVAETGASEDAIKRFSDQEIHEDDKLKCYMNCLFHQAGVVNDKGEFHYVKIQDFLPESMHLITLNWFKRCLYPEGENGCEKAFWLNKCWKTRDPVHYFLP

>AgamOBP4

MSVSVLVSSLVVLFCVQCLIEHIDGAMTMKQLTNSMDMMRQACAPKFKVEEAELHGLRKSIFPANPDKELKCYAMCIAQMAGTMTKKGEISFSKTMAQIEAMLPPEMKTMAKEALTHCKDTQTSYKDPCDKAYFSAKCAADFTPDTFMFP

>AgamOBP5

MAASRSCWWWRWWWDFILGLVAFFFIPFPSVECAMTRKQLINSMDMMRSACAPKFKVSTEMLDNLRGGIFAEDRELKCYTMCIAQMAGTMNKKGEINVPKTLAQMDAMLPPDMRDKAKEAIHSCRDVQGRYKDSCDKTFYSTKCLAEYDRDVFLFP

>AgamOBP6

MTSNAFYSSNTVTWVVAVIGVYCLVFRPALVHAQQSLTQADMDEIAKGMRKVCMSRHKISEEMANYPSQGIFPDDQEFKCYVACLMDLTQTSKKGKLNYDAAVKQIDILPENYRQPFRLGLDSCRTAADDATDRCEVAYILLKCFFKASPKFFFP

>AgamOBP7

MCEYSNTRNKMSNLVVVLVLLTMYIVLSAPFEIPDRYKKPAKMLHEICIAESGASEEQLRTCLDGTVPTAPAAKCYIHCLFDKIDVVDEATGRILLDRLLYIIPDDVKAAVDHLTRECSHIVTPDKCETAYETVKCYFNAHDEVIKFCHLLVLE

>AgamOBP8

MPSRKRLCRLLLLLLLPVDLELISQDADANVFPAYPVLRNSTPFSIFQTHGAYVVRTFADATAYRDECVQQYAGRGSSLIDYMRQVALHTDNADSRWCIVRCILQKADLLDGEGAPHEANVHAQMQHSNAIVEDPDDIRSETSRCLREPPAPDSGGGCLRAYTFFACIQSTEYDLF

>AgamOBP9

MLKFVVALLAFTAVVSAEFVVQTREDLLAYRAECVKSLGVSDELVEKYKSWNFPEDDTTQCYIKCIFNKMQLFDDTNGPIVDNLVVQLAHGRDANEVREEIVKCAGSNTDGNVCHWAFRGFQCFQKNNLSLIKASVKKD

>AgamOBP10

MVRVLIVFVALLTFAGQPFAVRGQQELSDLPEVKGYKLHCIESSGITESSAKKLAAGESIKEPDQPTKCFVQCFFQKLRLMDEKGVVLKDKLEVFLTKLMDADKAKDYVQQCDLRRTNPCDTAYAVYDCYLGKKAKLF

>AgamOBP11

MIKPFVCILIVAAGCANAFMYKHPYNHHQAAVLAHEPVVPVEFVKHTTSPAFRPASFLEVMEVVLDCFNTLRIPLQRFPSYLSGIFPEDPETKCFLRCVAIKLGVYCDEKGADLDRHCVQFGLGECCENFSNRHLVCLQQNSLPCPDRCTAAYKQELCFQEPIAKYLDYHFHDLVGLLHQAKCSHDLKMLHP

>AgamOBP12

MAPVRYHFVLWLLILIGVSSLVPPGECLDISKVTLDAAFYPLFGCARDLVVPEDLIELYKKRIFPDDQLTCCVFRCLGMRLGIYDDVKGFDVDKQYERVKDRLSVDEDTYKRGVKNCIRNVLRGRTLNNCEKAYLILNQCQGNTITNSLNQQLNEIRCN

>AgamOBP13

MKSFQIATLTVLLVLLAGTASAKKASTIFGMPLQQDPVPATSTFIVSDFLQFLQTAVTCFNKLRIPEERFPLYLAGVFPNCPETQCFVRCLSANLNLYCDETGSDIDRHYLQYGLGQDYNCFRQKAEQCLAANTSPCNDPCEAAYKQELCFLDEFRKYVDSNMNSLIAAVAVEKAEQNPVYYNMLAHN

>AgamOBP14

MKLSSAVLYFALLATAMVCRVQAGSAEELEQAKEMLRGLAAECKTKEGATDEDVEGFVNDKMPESRTQKCLAGCMQEQFGVSNGKAFQEDGFIEIAKMLMKGDETKIELAKEIAADCKAVANDDRCELAVDIMNCLKESAEKHGIELKH

>AgamOBP15/16

MLTIVVATSICLMATASANAPKSLSPELLQQMGQFRSECLRETGTTDEQIEQFNSPQSVQASHELQCYMYCMFRLHNVTRPNGELDLIDVYHAIPKQFNSIALKVLAKCNKSTGPIADACERAYSHHRCWKETEPELRLPVAVCLMF

>AgamOBP18/24

MKIELFTLSAPTVPRPGGPHTEGGRNADNFKLYSSLFVFPSPLQGARLEAEHVRRIHQNARECVKETGILPKNAFRVLSGDFSVDTMKAKCFVKCFLDKAGFIDDDGVIQQDVIREKLTVGIEAGKVNELIKKCSVEGTDACDTAYQMYKCFFSNHKVPKELFQMRKGIGRRNMQQ

>AgamOBP19

MAAYLISVVNYSNYGMYITQEQLEKTARTFRQVCQPKHKISDEVADAVNRGVFADTKDFKCYVSCLLDIMQVARKGKVNYEKSLKQIDTMLPDHMKPAFRAGLEACKSAAQGVKDHCEAATILLQCFYKNNPKFVFP

>AgamOBP20

MLFVFFTLLSCTKKKKIFPLRKSTVEQMMKSGEMIRSVCLGKTKVAEELVNGLRESKFADVKELKCYVNCVMEMMQTMKKGKLNYDASVKQIDTIMPDELAGPMRAALDICRTVADGIKNNCDAAYVLLQCLSKNNPKFIFP

>AgamOBP21

MQSLQIVFVVLLAAVSTMEQHEIAKSLAEQCRAELGGELPEDFATKMRLGDLTLDSETAKCTIQCMFAKVGFTLESGAANRDVLIAKLSKGNPTAKAEAFADVCENNEGETACDKAFSLYQCYHKNKSIFD

>AgamOBP22

MNSLLLIGGVLVVLNVQFVTAADNNESVIESCSNAVQGAANDELKVHYRANEFPDDPVTHCFVRCIGLELNLYDDKYGVDLQANWENLGNSDDADEEFVAKHRACLEAKNLETIEDLCERAYSAFQCLREDYEMYQNNNNATSE

>AgamOBP23

MKSFFCVASFFLLVASVHAFTLRQQKMVSIFALECMAETGIGAESLTKLRDGDLTANDRTAKCFMKCFFEKENFMDAEGKLQLEAIATALEKDYERAKIDEMLEKCGEQKEDACETAFNAYACYHDHYQNL

>AgamOBP25

MKFLVFAIVLSAICLDALVDGAAAPPPDLEDVSKIANGEAFALECLIESGLKLDSLAALSAKELDTNGSKIKCLVKCFFEKTGFMNKDGQLQEETITEQLSKFMPRERIESLVKNCNFQEADACETAYKVTECYFQNKAGLF

>AgamOBP26

MKTFVAIAVVALIAGTFALTIDQKKKAEGYAAECVKTTGVPPETAAKLKGGDFAGADDKTKCFAKCFLEKAGFMTDKGEIDEKTVIEKLSVDHDRAKVEGLVKKCNHKEANPCETAFKAYQCIYAAKGAVV

>AgamOBP27

MGRLDLVCLLAIVLLVHSCVSIAWSFSWACTMVKPFVFLLYRMDRTSWDHTSGVAMKPSCFGECFVKRAGFMNDNFTFNRDTIMRFTNRFVSKEISEKVYNICTDNVTPTYCVTAFDVYQCIYENVYKSWDSRK

>AgamOBP28

MKLLFATVLLAVCAAAQPLTDDQMKKAEGFALGCLEQHKGLNKEHLVLLRDGDFSKVDADTKCFLRCFLQQANFMDAAGKLQNDYVIERLSLNREKSKVEALVKKCSAGVEVEDSCETAFRAVECYHREKASLL

>AgamOBP29

MDENTPQKRCVSRAVTVGICGAIVLLLLVGTSPAPVEGLRCRTGEGPSADDVKRIVRTCMNKITNAGGGNFSSSSSSSTIERDRACLMQCFFEEMKATNADGFPEKHKVLHVITKDIREHELREFYVDSIQECFHMLGLDNRLKDKCDYSMRFVTCLSDRFETNCDDWESVTSAMF

>AgamOBP30

MVTQLSQPLPLRGQHTMATVNLYYLGLVCLLAVTATAASQCFRDAGQLKRVVQAQEECVRYLRIPCARLAVYNKFIYPNDAETQCMVRCMGLNLGWWNDTHGVQEASMRSFFHPDPNDCDYERRTYRCLHSQRLDRPAPHDEACERAYESFRCYYEHYGNLVVTPQFVRLNALQQLDVLLQCADMLQYPMPDRSFSCAKTHVAGAEGDFDCVLRCYMLRTGLYSEQYGPNLDRIYVQCNNYANETVFRETTDACYQRLRSDCQDECTLIARYVRECFPAGGIIFLNSLW

>AgamOBP31

MKQLVLLTICVLALMPLEVLSNDTKGLTIEKSFLQSVHDCAEYLQVPKHRLVQYLAYEFPPDEETKCLIFCVGTDLRWWNNTCGLQVPAIMNYFQPVLGDRQYEKRTSECLERNVHTAELPNNCCQAYETFQCYFREFGNLVTCPQYVPATKLQATQAALDCLTVLRVPTDLLQCYSKGDLPDVPETRCLYHCIDHRTGLYTTESGIHLSRFYVRDLEVNDLRYLSKETKACRDRVRMSGCDVCSEVYNTHRDCLSGIGVDGYTSGIIAEASRIALTNLATALSALPARSYAQRSPYPSFHRTCKAEHFGRSF

>AgamOBP32

MISIELKYITLACVLAATVTAGSHCHNDYYQLKSVSQAQEECARYQGIPCARLAVYNKYIYPNDTQTQCMVRCMGLNLGWWNDTHGVQEPAMRSFFHPDPDDCDYERRTYHCLNSQRLNHPSPHVDVCERAYESFRCYYEQYGNIVVTPQFVPLSDLQQVDVLLQCANMLPLTVGRSCAGGSKPSERDVDCLARCFLLRSGLYSEQHGPHLDRLYVQCNNYANETRFRETTGTCYRRLKSECQDECVLAGRFLRECFYEGGISIVNSLPASEASVESAGSLGSGQGSAELGESHQEKVLQTWKDLYDRENLQDLWDRQEL

>AgamOBP33

MATIKLKYITLACVLAATVTAGSHCHNDYYQLKSVSQAQEECARYQGIPCARLAVYNKYIYPNDTQTQCMVRCMGLNLGWWNDTHGVQEPAMRSFFHPDPDDCDYERRTYHCLNSQRLNHPSPHVDVCERAYESFRCYYEQYGNIVVTPQFVPLSDLQQVDVLLQCANMLPLTVGRSCAGGSKPSERDVDCLARCFLLRSGLYSEQHGPHLDRLYVQCNNYANETRFRETTGTCYRRLKSECQDECVLAGRFLRECFYEGGLLGSIPVLGGLGGLVGGLTGLTGLVPPVTLQLTSPGLAAVTLTLSAPSVMVGALPVPPVMVGTLGGAANVGIL

>AgamOBP34

MQFQLNCVQQATRATMNSFALSVFVLAVGAVSVSASLQHYVVEKSFNQAQAECAEYQGVHDDDLLRYVKEGYPDVEEVRCLLRCVAFNLRFWNHTTGLQKNMVAGHFVPYPDDFHNVERTEACLAENLYTCDDDLCTQVYKAFQCYYQYYGALSECPQFVVNSYLEDLQVAYDLFGMLAVSQSTLQSLAGGCFPSGEESLCFFYSFVTRSGLYSVEDGAKLERLYYQYKEEVFNPNNAQTVACLQNQKKLACKKSTCQQAYDTFQNCFGESRGLEYLLHTVFVDAAKAFLGQPVCYCNKVKTCPLHKCYGR

>AgamOBP35

MNFFTVSAIALVAIIGSIQAEHSPLPHYFVRKSFPEAQAECAVYLQVPDDRLQRYMREGYPDEPEVHCLVLCVLENLRAWENGTLHENVLANYFVPATEDCDNAKRTERCLVYLPQECNGEPCVQAYRAFQCYYQNYGTLTTCPEYVPSYYGEDLQLAYDLFDMLDVSEDTRRKLAGGCFPSGPESQCFFFAYVTRFGAWSKDAPLLHNLYTQSQEDAFKKDNAETNVCLTNLNKLACHKTRCEHATDVFSQCFGNTDLYKHFLAVFKDAAMTYTRQ

>AgamOBP36

MNFFTVSAIALVAIIGSIQAEHSPLPHYFVRKSFPEAQAECAVYLQVPDDRLQRYMREGYPDEPEVHCLVLCVLENLRAWENGTLHENVLANYFVPATEDCDNAKRTERCLVNLPQECNGEPCVQAYRAFQCYYQNYGTLTTCPEYVPSYYGEDLQLAYDLFDMLDVSEDTRRKLAGGCFPSGPESQCFFFAYVTRFGAWSKDAPLLHNLYTQSQEDAFKKDNAETNVCLTNLNKLACHKTRCEHATDVFSQCFGNTDLYKHFLAVFKDAAMTYTRQ

>AgamOBP37

MQFQLNCVQQATRATMNSFALSVFVLAVGAVSVSASLQHYVVEKSFNQAQAECAEYQGVHDDDLLRYVKEGYPDVEEVRCLLRCVAFNLRFWNHTTGLQKNMVAGHFVPYPDDFHNVERTEACLAENLYTCDDDLCTQVYKAFQCYYQYYGALSECPQFVVNSYLEDLQVAYDLFGMLAVSQSTLQSLAGGCFPSGEESLCFFYSFVTRSGLYSVEDGAKLERLYYQYKEEVFNPNNAQTVACLQNQKKLACKKSTCQQAYDTFQNCFGESRGLEYLLHTVFVDAAKAFLGQPVCYCNKVKTCPLHKCYGR

>AgamOBP38

MLTYRAWLLLALLGAQCALILGAPATGHGYDTKSFAQAYLECLRYLNISRQSLYAYDSAAVPLNCGSNCLLRCIGLNARWWHDETGLSERALVRFFRQAPADSLLQARACVAELPAPPADSCAGAYWSFRCYSDALGELIAHPAYVAPCGQEIRRAVSDCATMLQVEDGQLQTCVRTETFLRQGNGAALLRCVVLRLGLYADSTGVLCDRVRLLMDADTAEQWTVARAEEAKRCEEDLRALGADTCVVAAHAVELCYGWPAFGELWEVLKQEYGSSDDALAEESEQVVVRRSCTPWMRPLKMGRNRQKARPRRMRKSSCLKMLNRPNWTWQTGRSR

>AgamOBP39

MASSGQVVAAAAVLLLMQLQTVTSATFGARDPPPPALREAQAACVKYLGICENRLHQYNNSVYPTDQDTMCMVRCAGIMVGFWDDCQGLKLDGLANLFPALAANDRVRYQIMSCAEKRIATCPPQDTCARAYNGFRCFLDAQKGGFGAKDMQPQQSTPPQPFDAQEFIRSLSICAKLQRIPKDRRDLYVQGVFPNDDKTRSLIRCVGIRTGLYDDEQGPNIALLYSLFGAGQSESEFRRRANLCIDANQPLLEAQDKNAQAYVKLYRCFADQISALVRANANAMA

>AgamOBP40

MERDRSSSYVAAALLLVCISLASAPRGTEANIFGGKLYQKAQQDCILFMGINPLRLDQYKKFVYPPDRDTMCLIRCIGISLDFWDDILGFDVDLAEQEFSPLVDATFKKYLAGNITLKLELLDPLDNCARAYYAFRTFRAQIRQFIGTGTTTMAPSVNFQPLTAVQILDIIVDCAREVNLPPSFLTSLTKGIITDCPEVQCLIRCAAVRTGLYTDKDGALLANLHRQLDPPGEDLASFSLRQGMCLQRNQQPPTADCCTRAFKQFFTCLRPDFEQFFIRNRETVMQHFLYKTDQPAEDRQPPWCRTMCWIRSGAIWV

>AgamOBP41

MGYWALGTGLQLLLLILVLGGSELQVKAKGSLILRSFDEMVLECAELMSIVHSKLARIRSGVMLPDEDTKCLIRCVGISGRFWNDHTGLRKELLARYFVTDPADAYNVNRTETCLQELPALELNAEKCCGLAFESFLCYYYNYGNLRQDSVFVPLDHLQLQHVTSRCMDVHQITTEQLMSLSAEAMDANDKLHCLVRCIGLQTGVYSDREGVSIDRLNAQYGEGHCEKEFKTHAVECITKHRELAYGSPCKRAYHLLYKCFENVRNVISAYELPDSDGN

>AgamOBP42

MFTTRLLVGALVSLGLTACSFAFTEHGAIVQSIVQAQHECVTYLNLPKHRLYQYLMYNYSNDAKTKQMLRCVGLILQWWKSDGTLNEHVLAQYFMPDTSDSDYYNRTYRCIERKAPVDDDLCSRAFETFQCYLQQYGELLNCPKVVPLSDERLTETMHFCLDVLDIPFSDFEQWTSSSELFLHTEPARCLLRCFTIRAGLYSDQHGPFADRFKLQFGAPKPDVFDNELEGDYCVARLRREGHDACSLAARSLYECYYFADTLLPTFERILPLLRLVLHQPEVETAEME

>AgamOBP43

MCSNRSAFGLLLLAWLASVTILGVEAYATPPPTTANCTTVSTFDAALQECVVQLGIAPERLDQEYNLLLYPADRDTMCLVRCIGVLLRFWNDTTGLREATIRQYYEPAPEDQDYQNRTRSCLAALEPSVTDVCERAHRSFLCYHQHYGYLRKTDRYVPKTPLEMKQIQQDCVDVYGLDPARLNHYQDGQFPDDPETQCFVRCVGLRAGLYTDRDGPNIDRMYVQCESCADETLFRAKAGECIAAQRRHKLSKCTAAYRTLYHCFRDDQLDLYASLTTAAATAAAMTTTTTTKKSTPPNAIPALSVRKPSDRAKLSPDAWQLEIILEGLYNQKY

>AgamOBP44

MKQLVCIVVFALVTPNLIVAECDTKGLIVEKSFLQSVHDCTEYLQIPKERLGQYMANEFPPDDETKCLLFCVGVDLGWWNNTCGLQVPAIVSYFQPVQGDKQYEKRTKECLERRVGAIDSPNSCCQAYETFQCYFQEFGNLVTCPQYVRSTKLQATQAALDCLVMLRYPEKLLKVYASGKVEDSPETRCLYHCIDLRTGLYTQNGISLPRFFVRDAAYNDLRYLSKETKACRDRIRQSGCDKCSEVYNTHTECLSGLGEKGYTSGIITAAAKIALTNLCPAVALSYGGRKPSSTCSKASGTGQVYNLSYPGYKSRMSSCSRCGGRGH

>AgamOBP45

MQRRNASGGGVAVLLTAIMALLPTGCDASLDVPHLTLSKSFSRALQDCMEYLQVPGYRYAEYAANSFPDDPETKCLLRCVGLNLRWWNDTTGMQTAVIEGFFHPDPLDELYENRTAECLRKELSHADTTDCCCLAYDSFRCYLQHYGNLVPCARFYPEDETRFVRAAQDCIEFLQIPHKLLKSYSAGSFPDAPETRCLLRCFFLRTGVFHVDTGFDVERLYTRDYEQPDERYLAQETEARLHKLRGSTGDQCTEVYLAYRDVLGELGRAYYEYDVLQAAAAKMTVCEVAVEPPAMTTTTTTTTTTPTTATACPSTTEFNYKELNCQNCGRLFISNNGRVSCCRCMKSSTPFGKFFF

>AgamOBP46

MNPIVGKVFLVLCGSLLVTGAPNTCGKLDLKTDPFTCCTIPKLLDVTIVSSCFEKFPIDKDAADKGAASMPKTEVTDCMSECILNSTGIYNRRGDVDEKKLNSVFTDSLPANSPWLNVVRKAIKECTAKADKKDKEFQKDVADQKKATPKGTQVCNPEASFLVDCIHTTVFSDCPTNLRSTSTECDAIWNFLKNCPFSALRQ

>AgamOBP47

MKHLKAFDEAQNDIKAVQKRLSTSSTILSGIQKNMAHLNLLQIGVLSLIAVGSVFAGNPCLKGPPVPKNAAECCVTPFLVEPSAFMTCHSKWIGQTKRQMAMEGIPRGCCVAECVMNSTSLYSNGKIDREALTKLYLASTKSMAPEWNKITLDAIDGCFKMADTIKDEIEAGAKLTPAFEGEQICHPISGTILACMGMTLFAECPAKLFTVNDDCNKLKSYHSKCPFL

>AgamOBP48

MGQRQRVVVQLALCFLTFGALLQAGVLAGDNPCAAGPPVDTNPAECCPTPMLVDGTIMMDCYKKYGEQTKKQLQMDGIPRGCCIAECAMNATNMYADGMLKRDDLSKMFMDAVKDKPEWMSLVRDATNACFELAEKKMDEIEAGAKLEPSFEGEKICHPISGTILRCMGMMMFAQCPASVFNVNENCNKLREYGSICPMI

>AgamOBP49

MEWNWTFLFRSFLLLTLHLLPQSVADDCIDMDLHSMEVARCCRYEPISTEEVAEKCYQELAPNIPPNSSDFPVCFIDCSYRQMGYITNEANEIDQSKYGQFLAGFDTAYKIAVERAVAACATVQEDIRRDVANVPSKCNAFALLFHVCVTQITLKHCPDDRWTASEICGKVRMGVPPCA

>AgamOBP50

MHVALPFSVVGKLTCLSPFLQSIKVASCCQLEAFLTLPTYGNCLQTIAEKYPDALWQGTVCAFDCTYREMGILTGVDDINVEQISTNQAGYDQAYQEAIAKAVTACMAQKDKIREEADVVQSECSMFAVKFHACVSLETMRNCPAERWDSSVLCEKVRSGVTVCPL

>AgamOBP51

MCHRVLSLCGFLLLGLQCGWQTLAEDCMDIKIFVSETLQLFRLDGASPKFTAFSSFLQTTKVASCCQLEEFLTLKTYGNCLNTMAEKYPNSTLDYLVCGLDCTYREMGILTGVDDINVEQISTNQAVYGEAYQEAIGKAVDACLAQRDEFREQEKFTKSECGMFALKFQGCIMVESMRNCPAERWDSSVLCEKVRSGVAVCPP

>AgamOBP52

MLFKLFTIPFRCPLFFSKHPKQFPPSKKQSELPYCCQTEPLIPEHVSTKCKEREAANHNPGTELFEVCYQQCIYEELEAVDGLEIRVEKLYALAEGFPADYRHAVHLAIDECVKRLRKTRHMFEQMNAQCSLFGFAVDRCVRLLIYENCPTARWSASVACTKSRQGVPFC

>AgamOBP53

MSFRSISALVILLHLFVICTPMPECISQTQKFEVPHCCQMEELIPRPSRTKCQEKAAIDHNPGFQAYFVVNCLAQCQLEELEVIDGEELHLEKLYPLTAKFPADYRHAVRQAIDECDAWLQGKKKERRRPDGKAHCPLIGMEVENCLHRTTFSNCPNSRWKASITCNKVRQGLPFC

>AgamOBP54

MDLKKSVAVVFVSFGWMMLLATAADPDCENLKNRREEMEQCCQVNMIIPLDGAEDCSSSVDETSEPHDKMMCTLECKLKSLGLLNGDDLVEAKVQEYIDRLEGDWKGTAKTIATECITTITEMKKKIQERDHDMKCSPVGAFFMMCLMKHTQAKCPEDKWQNTSFCNKMRSGECFPKRGRQ

>AgamOBP55

MLPTGLERTVLWVTVIVLVKVMVKSDAQVCCMVEHTFPQEPYRVCHEQHATPQMDNGTVMCIHQCYYKAIGMFAADGKVNTDAYIKYRDELDPTLRDAFSYSMVVCAKIIAKRMNNNIAEVNRMRCSPLPYLFNRCLMEVGIGNCPPERWMNCKHG

>AgamOBP56

MLKLALFVGLVGCVVAYDFQDSFYNEVLMEDLLDNADEPIMFGRFRRSASEVQDDKCKRKYKCCNDANTENMEKIHEIKKQCFMEVRNKNKADGAYEPVDFFSCERLNKTKMEVICAMECVGRKKEVVNEDGTLIEPKLMEFVKSNFAADDWQQPLLAGHIETCVKEAKEKAAKMPREAGQCSSETSNFGYCMWRQMALACPKDKQVANKRCDRIREKLANNEPLHYYKAELEDM

>AgamOBP57

MGKVLILFVGALVVASVTAGRFERSVFAPRIKRDATMRCCNDGFEKSEVHAKFAEVRTACMEELGLGETTHEELIKNREHLNCITECIAKKEGIADENGALLHTDLAKVVLEHMSTIEWKVPLAEGFIQQCFDEVELTDGAFVPSDEAKCNPEGFDFVFCLWRQFTLACPEEFRDDSEKCVELRDKLTNKEDVSDLHDDIEAAE

>AgamOBP58

MSLHLFVRTSTTHGINMRSSSVWLIVVCAVTVASANSEELLRGKENCLRHDDFPSPNECCSKPQWINRYAVRRCRYIHAEVDGSRYERGSCEARCGLFKINMTMTDRIQRVRVYRPRLQTRGIDQGWINVVLKALSYCKPKVTQLQGRHVRTDEEMEQCEIAEDIFGDCVQAQMFMHCPRATWIESRSCQTMRELLATGCPYKTLGEVVVLNDEGYVRDDRILEEEYDRPYRGRGRTESPRYDYDDNDGYSRGGQYDQRGGNYPRGTERNRNGNGYGAGDDGGYVV

>AgamOBP59

MPRLLPEQVIETCRARPLPSVIPGVPDPLPENCIAECALNETGILFNGQFRVEQAVKALSTQVPNDTLTWQHVIEVASKKCYIITVGDSFYLRDVAKNLISPQCIPSSFRFLQCTFSIVYRDCPDIYWNYQNDRCGQFVVALNNCHYLFRHIWDI

>AgamOBP60

MLSFVFLASIIVGLVSSQPPAPDASCFQPTAVTAEDCCKIPKPIDNAIMEKCRAENPKPGQMPAPGVPRTEGCCIVQCAMMETGGFVNNALNTDAIKRSMASTLGADSNFGSLVNGAVDTCARQIQNDPAYSVAPISSSPDRAGCSFIPQGFVNCLYTALFKSCPAATWTESSDCQALKTKLDSGCPFFLLMGRGPRN

>AgamOBP61

MNRLVCAFGVIFVVATLELVLAHPGKDVLGCHNGTSITVDECCAIPMLANKTVIEKCKAAHPFKPPQNTDDKGPRGHPGECLAECIMKGMGALKNEKVDGPAFRKAIEPVVKANPAFAKLLDDTVKQCHESINVDSEFTRYVTKPVCKADAKAFINCVYGTLFEQCPTNVWTQKDGCTQLKDKIKKGCAYFALRKHGGRRMRPT

>AgamOBP62

MKQRCALAGCEKLLPAVLLLLFALQATVPEGTVAGCSMLNNDNAEQRGAAMLADPATVKQVPEVTMQDAIAQCNRSFIIQPEYLAELNQTGSFPEETDKIPLCFIRCYLKALGILTEDDKVNKEVALARNWATSGETVDECLEEMAGSACEQAYFFTRCVMTRALVDGKSKDNK

>AgamOBP63

MKTIACLVLASAFIACAVATISEEQREAARQLAGKCMQQTGASEDDVNRLRSGDTEGADRNTRCFVQCFFQGAGFVDQDGSVQTDELTQKLASEYGQEKADELVARCRNNDGPDACERSFRLLQCYMENRASLMF

>AgamOBP64

MGAFESGLGLLGWVAFGMVLLLAGRGCHAQDFKGAIDHCTKDFEMDMDIVVSLKYGDFTERDPLIECFTECLMKKSGFMYDDYTYNKTLIIGFAGRYLEPEGAQAVYDNCIDRFGQTVCVTGFEMYQCIHETAVSEWVSSNF

>AgamOBP65

MQLAICVWTAVCLQRNIIEGFLVELEAFPSSHQQPPKTSPPVRSCGETFNLTDPRTCCSIPYLLPADVVEPCLEIPLSPIDLAGESNVCVFWQCRAECALNRTEMLVDGHFQLETAMQQLTNATSEDSTLTKRIQYAIGACNELFLNCPPQYWTASDECNQLVRTLNNCPHFLVHTDTF

>AgamOBP66

MATTIARIGSANWAKVLVLLWLVQLATAGEPNPACKTMPTVDKDNEDKCCDVPEMFPNETLNACMEEYQKSSKPPLQKSCEITTCVLKKQSLIKSDNTVDKDKIKSYIKEMVKGSDEWKTLVEKAVLEECLPLMDKDPSNVLSKLKSSLGDCDPAPALTIACAAAKFYVNCPAKDRTKSPMCDEWRTFLSKCSNSLEDLNAIFMVLENQKTR

>AgamOBP67

MNPVVCAFGVIFVVVTLELVVAHPGKDVLGCHNGTSITVDECCAIPMLANKTVIEKCKAAHPFKPPQNTDDKGPRGHPGECIAECIMKGMGALKNEKVDGPAFRKAIEPVVKANPAFAKLLDDTVKQCHESINVDSEFTRYVTKPVCKADAKAFINCVYGTLFEQCPTNVWTQKDGCTQLKDKIKKGCAYFALRKHGGRRMRPT

>AgamOBP68

MATTIARIGSANWAKLLVLLWLVQLATAGEPNPACKTLPTVDKDNEDKCCDVPEMFPNETLNACMEEHQQSSKPPLQKSCEITTCVLKKQSLIKSDNTVDKDKIKSYIKEMVKGSDEWKTLVEKAVLEECLPLMDKDPSNVLSKLKSSLGDCDPAPALTIACAAAKFYVNCPAKDRTKSPMCDEWRTFLSKCSNSLEDLNAIFMVLENQKTR

>AgamOBP79

MDRLLLVLLSSASLLLTVYGIKHHIVTKSWSEAQSDCLQYLRVESPGRYLSHRYRDNQTSKQLIFCIILNLRIYDPTQNVLRLKAMGQFFNPDKTDTLYVNRTNACLLRVKVPPLVDSSEDSQLYSGVMGTLYEVFRCFYHCYGNINAIAPKLPPTVLELEKIQQECARMVGVSERLLDGGLQLSSHPRYSKLPRCIMLRSGGSVDYLTHRNNSSRRFKLKKNVENDTL

>AgamOBP80

MRRQYSMWASTVAVIACGSALMLLHPVGADAPKKRCLTKPNVSKKVDMVIHQCQEEIKSSLIEDALKIFTAEHGQWHDRRKRDEGGLDFSHPTIVSHEDKWIAGCLMQCVYRKNNAIDKNGWPTLDGLVSLYTDGVNEQGYFMATLRGVDRCLKGTSKKYQIKRNDAAENFEQCEVAFDVFDCISDMITDYCSGQMEDDH

>AgamOBP82

ICGAIVLLLLVGTSPAPVEGLRCRTGEGPSADDVKRIVRTCMNKITNAGTMGEWGQRDRNGEEQQMMRDYGRSHRRRKRQYYGGQTSGSSSSGSAGEHSYNGRASPQYGEAGQGGNGTRSGGNSSSSSSSTIERDRACLMQCFFEEMKATNADGFPEKHKVLHVITKDIREHELREFYVDSIQECFHMLGLDNRLKDKCDYSMRFVTCLSDRFETNCDDWESVTSAMF

>AgamOBP83

SITQEQLEKTARTFRQVCQPKHKISDEVADAVNRGVFADTKDFKCYVSCLLDIMQVARKGKVNYEKSLKQIDTMLPDHMKPAFRAGLEACKSAAQGVKDHCEAAAILLQCFYKNNPKFVFP

**Table S3 Amino acid sequences of 97 CSPs of *B. odoriphaga* and other Diptera insect used in phylogenetic analyses.**>BodoCSP1

MKYLVAFAFVAVVAMAAARPEDKYTTKYDGVDVDEILKSDRLFNNYFKCLMDKGKCTPDGSELKRVLPDALKTNCSKCSEKQKTGTEHVIKFLIDNKPAQWEALQNKYDPEHIYTTMFRDEAKNHGINV

>BodoCSP2

MNSKIRVLVCTLLVIISCGEHVVDGDDKNITRLLNNQVIVSRQIMCVLEKSPCDQLGRQLKAALPEVILRNCRNCSPTQAQNAQKLTNFLQTRYPDVWAMLIRKYRGV

>BodoCSP3

MTIKYCFLLLMISQILIVNKAQTKAYETKYDDIDLDELLKNDRLRHSYVKCLLGEGPCTPDGQELKNALPDAIQSKCSKCTEKQKAGAEKVTHYLIDNKPDEWQKLADKFDKDDDYKTKYLMEKEKDKSKSSEESKDSEED

>BodoCSP4

MNSITIIVFTLFVCATVAQEYTKKYDNVNIETILSNDRVITNYINCLLGKGACTKEGRELKKLLPDAIQTDCSKCTQEQKRNSRKVITFIRSRRPQDWAKLIAKYDPEGLFNKRSSFL

>BodoCSP5

MHRIFTVIGTILVYTKYAYCDQGIDLYSVRYNNLDIDTILNSDRLVTNYVECLLSRKPCSPEGKELKRILPEALRTKCGRCSNTQKEVALKVLKKLYVYYPKHYNDLRAKWDQTGEYHRRFEEYLREERFNSISGDTDRDQTQVVQKISPQNPLTTPASTTAHQISTLDTQRPARNPDDVQQNPTLNQFDLFNRFGGEDDENDQTAPIPSVQAPVRTFILNIRKRITSFLSLISP

>DmelCSP1

MKASLALVFCVCVGLAAAAPEKTYTNKYDSVNVDEVLGNNRVLGNYLKCLMDKGPCTAEGRELKRLLPDALHSDCSKCTEVQRKNSQKVINYLRANKAGEWKLLLNKYDPQGIYRAKHEGH

>DmelCSP2

MKMILALVVLGLVLVAAEDKYTTKYDNIDVDEILKSDRLFGNYFKCLVDNGKCTPEGRELKKSLPDALKTECSKCSEKQRQNTDKVIRYIIENKPEEWKQLQAKYDPDEIYIKRYRATAEASGIKV

>DmelCSP3

MGQPGFRRAIGHVSLVVALMCTTCFQVEGLPHPPATSPSPMMERMVEQAYDDKFDNVDLDEILNQERLLINYIKCLEGTGPCTPDAKMLKEILPDAIQTDCTKCTEKQRYGAEKVTRHLIDNRPTDWERLEKIYDPEGTYRIKYQEMKSKANEEP

>DmelCSP4

MLLLNKNRVISLVVNFIFLIILISSSVQADERNINKLLNNQVVVSRQIMCILGKSECDQLGLQLKAALPEVITRKCRNCSPQQAQKAQKLTTFLQTRYPDVWAMLLRKYDSA

>AgamCSP1

MKLFVVVALALVAAVAAQDKYTSKYDNINVDEILKSDRLFGNYYKCLLDQGRCTPDGNELKRILPDALQTNCEKCSEKQRDGAIKVINYLIQNRKDQWDVLQKKFDPENKYLEKYRGQAQKEGIKLD

>AgamCSP2

MKLFVAIAFALLALAAAQEQYTTKYDGIDLDEILKSDRLFNNYFKCLMDEGRCTPDGNELKKILPEALQTNCEKCSEKQRSGAIKVINYVIENRKEQWDALQKKYDPENLYVEKYREEAKKEGIKLE

>AgamCSP3

MKFFVVVALALVAAVAAQDKYTTKYDGVDLDEILKSDRLFNNYYKCLMDTGRCTPDGNELKRILPDALKTDCAKCSEKQKSGTEKVINYLIDNRKDQWENLQKKYDPENIYVNKYREDAKKKGINL

>AgamCSP4

MERFLLLLLFVAIVLGETANETYVTKYDNIDLEEIFSSKRLMDNYMNCLKNVGPCTPDGRELKDNLPDALMSDCVKCSEKQRIGSDKVIKFIVANRPDDFAILEQLYDPTGEYRRKYMQSDALAEHVKQEDRDLSSSGDGDADTETEAHATEHNSQDHDHREGQSDAE

>AgamCSP5

MRKVWLLASVVLAFLDFVKSQEVARTLYSTRYDNLDIDTILASNRLVTNYVDCLLSRKPCPPEGKDLKRILPEALRTKCARCSPIQKENALKIITRLYYDYPDQYRALRERWDPSGEYHRRFEEYLRGLQFNQIGGSNGGSGVGNTVLSNL

>AgamCSP6

MKHLTMVAIFAMVVVLASAQKYTDKFDNIDVDRVLSNDRILNNYLKCLLDKGPCTQEGRELKKTLPDALKTNCEKCSEKQRTSSRKVIAHLEERKPQEWKKLLDKYDPEGIYKSKFEKINKRS

>AgamCSP7

MLSAAVIVVMAALVIVGPQPAAANDSQNINRLLNNQVIVSRQIMCVLEKSPCDQLGRQLKAALPEVIQRNCRNCSPQQAQNAQKLTNFLQTRYPEVWAMLIRKYGAV

>AgamCSP8

MLHNLFLSLSLYVSVCGDPSGSTCAAEATTARTQVSDEALDKALSDKRYLMRQLKCALGEVACDPVGKRLKSLAPFVLRGACPQCTPAEMNQIKKTLAHLQRNFPSEWNKLVQTYAG

>AsinCSP1

MKLIVFIAFALVATVAAQQYTTKYDNIDVDEILKSDRLFNNYYKCLLDEGRCTPDGNELKRILPDALQTDCAKCSEKQRSGAIRVLNYLIQNRPTQWAVLQKKYDPENQYVEKYREQAKKEGIKLD

>AsinCSP2

MKLFVAIAFALLAIVAAQEQYTTKYDGIDLDEILKSDRLFNNYFKCLMDEGRCTPDGNELKKILPEALQTNCAKCSEKQRAGAIQVINYVIENRKEQWDALQKKYDPENLYIEKYREEAKKEGIKLE

>AsinCSP3

MKFFVVVALAMVAAVAAQEKYTTKYDGVDLDEILKSDRLFNNYYKCLLDQGRCTPDGNELKRILPDALQTDCAKCSEKQKSGTEKVINYLIDNRNDQWQNLQKKYDPENIYVNKYRDEANKKGIKL

>AsinCSP4

MLPLVLVVMAVMVLGSMADNYVTKYDNINLEEIFNSSRLMNNYMNCLKNVGPCTPDGKELKNNLPDALMSDCVKCSEKQRIGSDKVIKFIIANRPDDFATLEQLYDPTGEYRRKYLAPDGTLKPREDGEEDVPPVKETNDGDIEIDSVAHATEHKTAPSQDHDHGEGHTDESKN

>AsinCSP5

MRKVWLLASAVLAFLNFVKSQEVARTLYSARYDNLDIDTILGSNRLVSNYVDCLLSRKPCPPEGKDLKRILPEALRTKCARCSPIQKENALKIITRLYFDYPDQYRALRERWDPSGEYHRRFEDYLRGLQFNQIGGNGATNGGQAPSGGDNGNTVVENGGGNDRPVRNDLDRQPASQSSNVQAVVIDPTLSSGGRQRPNDPGPRHR

>AsinCSP6

MKNLSIVAVLAMLVVLVSAQKYTSKFDDIDVDRVLSNDRILNNYLKCLLDKGPCTQEGRELKKTLPDALKTNCEKCSEKQRTSSRKVIAHLEDRKPQEWKKLLDKYDPEGIYKSKFEKLNKRS

>AsinCSP7

MSSKALPNLFMLSAAVIALMAVLIAGPQPVVANDSQNINRLLNNQVIVSRQIMCVLEKSPCDQLGRQLKAALPEVIQRNCRNCSPQQAQNAQKLTNFLQTRYPEVWAMLIRKYGAV

>AsinCSP8

MSGKVSSRSSSSSICWFIGVGLCALVMVVQLAGLAEADATTTSRTQVSDEALDKALSDKRYLMRQLKCALGEVPCDPVGKRLKSLAPFVLRGACPQCTPAEMNQIKKTLAHLQRNFPSEWNKLVQTYAG

>CquiCSP1

MAVGVALAVLVLVLGGGQVAANDTQNLNRLLNNQVIVSRQIMCVLEKSPCDQLGRQLKAALPEVIQRNCRNCSPQQAQNAQKLTNFLQTRYPEVWAMLIRKYGAV

>CquiCSP2

MKSLVLSVLCLATLLVATTVAQQPRQYTDKFDNINVDQVLSNDRILSNYIRCLLDKGPCTQEGRELKKTLPDALRSNCEKCSEKQRNNSRKVISHLEAKKPADWKKLLDKYDPEGLYKSKFEKLNKRS

>CquiCSP3

MRNVWIVVASGLLAFANFVKSQETGRTLYSSRYDNLDIDTILSSNRLVNNYVDCLLSRKPCPPEGKDLKRILPEALRTKCGRCSSTQKENALKIITTLYYSYPDQYQALRERWDPSGEYHRRFEEYLRGIQFNQIGGNGNGNGGPSERPVRNDFDRDQSQILLQTLILSTTTAQPPVPEQRPTQLASAHSGGVSGDEAGGGSEPPTSIPATDSSPYIPLSGNPHKQ

>CquiCSP4

MFSNIVILGAVLLCCTLQAAVNGAEYDTKYDNVDLDEIFRSTRLLNNYMNCLKKVGPCTPEGKELKENLPDALANDCAKCSDKQKAGASKVIHFIVENRRDDFGALEKLYDPSGEFRRKYLDEQMHFRLHREEGGSAAVEEKSPASESEATTEEAAAAAQDHGQSAEGRR

>CquiCSP5

MKLYIVVALALIAAVAAQDKYTTKYDGIDLDEILKSDRLFNNYFKCLMDQGRCTPDGKELKRLLPDALNTNCSKCSEKQKQGTEKVVNYLIDNRPSQWKTLQEKYDPDNTYRTKYRVEAKKFGITV

>CquiCSP6

MKFFIVALALFALAVAQEDDGDKYTSKYDKIDLDDILGSDRLFKNYYNCLLDQGACTPEGNYLKRVLPEALETNCAKCTEKQDADSTKTIKYLSENRPAEWKVLKAKFDPENKYVEKYVDKAEKEGIKL

>CquiCSP7

MKFFIIALALFALAVAQEDDGDKYTSKYDKIDLDDILGSDRLFNNYYKCLLDQGPCTPEGNYLKRVLPEALETNCVKCTEKQDADSTKTIKYLSENRPAEWKVLKAKFDPENKYVEKYEDKAEKEGIKL

>CquiCSP8

MKFFIVALALVALVAAQEEEGDKYTTRYDNIDLDEILKSDRLFKNYYACLVEEGRCTAEGSYLKRILPEALETNCAKCSDKQRDDGVRAIKYMAENRAEEWKVLKARFDPENKYVEKYLADAEKEGIKL

>CquiCSP9

MKFFIVALALVALVAAQEEEGDKYTTKYDKIDLDDILKSDRLFKNYYACLLDDGPCTPEGSYLKRILPEALETNCAKCSDKQRDDGVRAIKYMAENRAEEWKVLKAKFDPENVYVEKYLADAEKEGIKL

>CquiCSP10

MKFFIVALALVALVAAQEEDGDKYTTRYDNIDLDEILKSDRLFKNYYNCLVDEGRCTAEGSYLKRILPDALETNCAKCSDKQRDDGVRAIKYMADNRAEEWKVLKARFDPENKYVEKYLADAEKEGIKL

>CquiCSP11

MKLFIVGLALFAVAFAQDAEVETLEEIGEKYTTKFDKIDLDDILKSDRLFKNYYNCLMEEGPCTPEGNYLKRVLPEALENSCNKCSEKQQKDSVKAIKYLTENRSEAWKVLKAKYDPENKYVEKYLTDADAEGIKL

>CquiCSP12

MKLIIVLALVALAAAQVPFTNKYDHINVEEILMSDRLFKNYFNCLIDEGACTPEASELKEKLPEALENNCELCTEKQKDTSVKVIRYLIDKRPVEWGVLKTKFDPNNKFVDRYREEAEAAGIKL

>CquiCSP13

MKLLIVFALVALVAAQDSTYTNKYDNIDVDEILKSDRLFKNYFNCLIDQGPCTPDATELKQSLPDALENNCSKCTPKQKEVGYKVVGWLINNRPEEWNVLRAKYDPENKFIEKYRDEAKAAGINL

>CquiCSP14

MKLLIVFALVALVAAQDSTYTNKYDHIDVDEILKSDRLFKNYYNCLIDQGPCTPDAAELKQSLPDALENNCSKCTPKQKETGYKVISSLIENRPAEWAVLQDKYDPERKFVEKYREEAAAAGIKL

>CquiCSP15

MKLLIAFALVALVAAQDSTYTNKYDHIDVDEILKSDRLFKNYYNCLIDQGPCTPDAAELKQSLPDALENNCSKCTPKQKETGYKVISSLIENRPAEWAVLQDKYDPERKFVEKYREEAAAAGIKL

>CquiCSP16

MKLLIAFALVALVAAQDSTYTNKYDHIDVDEILKSDRLFKNYYNCLIDQGPCTPDAAELKQSLPDALENNCSKCTPKQKETGYKVISSLIENRPAEWAVLQDKYDPERKFVEKYREEAAAAGIKL

>CquiCSP17

MKLLIVFALVALVAAQDSTYTNKYDHIDVEEILKSDRLFKNYYNCLIDQGPCTPDAAELKQSLPDALENNCSKCTPKQKETGYKVISSLIENRPAEWAVLQDKYDPERKFVEKYREEAAAAGIKL

>CquiCSP18

MKLFIVFALVALVAAQDNTYSSKYDNVDIDEILKTDRLFKNYYNCLIDQGPCTPDATELKQVLPDALENNCSKCTPKQKDAGYKVVGFLIDNRPEEWAVVRAKYDPENKFVEKYRGDAEAAGVKL

>CquiCSP19

MKTFIVFGLLALVAAQDSTYTNKYDHIDVEEILKSDRLFKNYYNCLIDQGPCTPDATELKQSLPDALENNCSKCTPKQKEVGNKVVAYLIESRPEEWAVLKAKFDPENKFVEKYREEAAAAGIKL

>CquiCSP20

MKFFVAFFALVALVAAQELYTNKFDTVDLDEILKSDRLFKNYYQCLLDEGRCTPDGNELKRVLPEALETNCAKCSEKQKTAGNKAFKYLAANRPTEWKALLAKFDPESKYTAKKLDIKIFIPFNY

>CquiCSP21

MKFFVAIFAALLAVAAAQELYTSKYDSVDVDEILKSDRLFKNYYQCLLEDGRCTPEGNELKRILPEALETNCAKCSEKQRSGAIKAFGYLSENRPEEWKALRARFDPENKYLEQYREEGEKNGIKF

>CquiCSP22

MKCVIVAVLALIALVSAQEAEQYTTKYDNIDLDEILKSDRLFNNYYKCLMDEGPCTPDGNELKRILPEALQTNCAKCSEAQRAGAIKVINHVIDNRPEQWKALQAKYDPENIYVEKYRTEAAEAGIAL

>CquiCSP23

MKYLIALALLIVAVAAQNKYTTKYDGIDLDEILKSDRLFNNYYKCLLEQPGGRCTPDANELKRILPEALQTNCAKCSKKQKDGAVKVINYLIDNRSAQWQVLQKKYDPENVYINQYRTEARAAGVKV

>CquiCSP24

MKCFIAFALLVVAVAAQNKYTTKYDGIDIDEILKSDRLFNNYYKCLLDQGRCTPDANELKRILPEALQTNCAKCTEKQKDGAVRVINYLIDNRSAQWQVLQKKFDPENVYINQYRNEARAAGIKV

>CquiCSP25

MYCKAVTTKMSSHQQQQQRRRHRTPVYCDQLASWLLLAVFAVSTVLLCCCVTSAQAQPQTPPTKSQVSDEALDKALSDKRYLMRQLKCALGEVPCDPVGKRLKSLAPFVLRGACPQCTATEMNQIKKTLAHLQRNFPQEWNKLVQTYAG

>CquiCSP26

MKFFIVALALFALAVAQEDDGDKYTSKYDKIDLDDILGSDRLFKNYYNCLLDQGACTPEGNYLKRVLPEALETNCAKCTEKQDADSTKTIKYLSENRPAEWKVLKAKFDPENKYVEKYVDKAEKEGIKL

>CquiCSP27

MIPSKYDKIDLDDILGSDRLFNNYYKCLLDQGPCTPEGNYLKRVLPEALETNCVKCTEKQDADSTKTIKFLSENRPAEWKVLKAKFDPENKYVEKYEDKAEKEGIKL

>AaegCSP1

MNTTKLVMLSATLIVALMVFNWPQPAAANDSQNLNRLLNNQVIVSRQIMCVLEKSPCDQLGRQLKGRSGVDF

>AaegCSP2

MKSVCLIVFGVVALVATVSAQQKYTDKFDNINVDQVLSNDRILSNYLKCLLEKGPCTQEGRELKKTLPDALRTNCEKCSEKQRTNSRKVISHLESKKPAEWKKLLDKYDPEGIYKSKFEKLNKRS

>AaegCSP3

MERKSTGCNPPQSSPPVLQTRLNRRAPSGEIPSDWDLEWQLFVPRIGIYPRTGKRGIVRCEKKIEREAKKPEENAVLKLNQCANKSGRYECELSSEQKKCFVRVASALLAFANFVKSQDSARNLYSSRYDNLDIDTILGSNRLVNNYVDCLLSRKPCPPEGKDLKRILPEALRTKCARCSVTQKENALKIITTLYYSYPDQYMALRERWDPSGEYHRRFEEYLQGIQFNQIGSNGNDRPVRNDFDRDQSQVLLQTLILSSTTVAPSSPPQPQPQSPNALPNGPEQRPQQLAPSAQLTQPAEATKKLE

>AaegCSP4

MSHKFCWIVVICAISIINVNCYDTKYDNVNLDEIFKSTRLLNNYINCLKNMGPCTPDAKELKELLPDALESECAHCTEKQKVGAERVINFVVDNRPDDFKILESMYDPAGEYRRKYLRDHPNFHDQGAPLTAADATENPPSSNGGDEAPTEESQQNQGQSEDGDDRRR

>AaegCSP5

MKLFAVVALALFAVAAAQEKYTTKYDGVDLDEILKSDRLFNNYYKCLMDQGRCTPDGNELKRVLPDALKTDCAKCSPKQRDGTQKVVNYLIDNRPSQWKNLQAKYDPQNIYVEKYRTEAKKAGIKL

>AaegCSP6

MKYFFVVFLALAATVIAQNEINQYTTKFDSIDVDEILKSDRLFNNYYKCLLDLGRCTPEGVELKRVLPEALETSCAKCSEKQRETSDRAIKYLTENRPEEWKVLKARYDPDNKYSKKNENDA

>AaegCSP7

MKLFIALALLAVAAAQEATYNNRYDNIDVEEILKSDRLFKNYFNCLMDAGPCTPEGTDLKKYLPDALETGCTKCTEKQRDTGNKVIAWLIENRPMEWVMLKSKYDPENKLTERYRELAAKAGIAL

>AaegCSP8

MKIIILCTLLAVVAAQEATYNNRYDNIDVEEILKSDRLFKNYFNCLMDAGPCTPEGTDLKKYLPDALETGCTKCTEKQRDTGNKVIAWLIENRPMEWTMLKNKYDPENKLTERYRELAAKAGIAL

>AaegCSP9

MKFLVAVLSLIAVAAAQDLYTTKFDNIDVDEILKSDRLFKSYYQCLLDEGRCTPEGNELKRSLPDALETGCSKCSEKQRSAGVRAVKYLSENRPTEFKALRNRFDPENKYVEQYVRDAEKEGITLNI

>AaegCSP10

MKIFIAVFTLMAVVAAQEFYTSKFDNIDVDEILKSDRLFKNYYQCLLDQGRCTPEGNELKRVLPDALETACSKCSEKQRSAGVRAVKYLSENRPAEFKALRARFDPENKYVDQYVRDAEKEGITLNIS

>AaegCSP11

MKFFVVALALIAAVAAQDEAMYTSKFDNINLDEILQSDRLFKNYYNCLTDAGPCTPEGNELKRVLPEALETNCAKCSPKQREAGTRAIKHVTENRPEEWKVLRARFDPENKYIERFSAEAEKEGIKL

>AaegCSP12

MDHSSISASKPSAHFKVLNRSFTHKFGATDTIMKIFVVALALIAAVAAQDEAMYTSKFDNINLDEILMSDRLFKNYYNCLTDAGPCTPEGNELKRVLPEALETNCAKCSPKQREAGTRAIKYVTENRAEEWKVLRARFDPEDKYVAQYLAEAEKEGIKL

>AaegCSP13

MKFFIVVLALFAVAAARPQEDKYTTKYDSIDIDEILKSDRLFKNYFNCLMDTGACTPEGNELKRVLPDSLENNCSKCSEKQQTSSTKIIKFLTENKPEEWTMLKAKYDPDNKYVQKYVADADKDGIKL

>AaegCSP14

MKFFIVALALFAAAAARPQEDKYTTKYDSIDIDEILKSDRLFKNYFNCLMDTGACTPEGNELKRVLPDSLENNCSKCSEKQQTSSTKIIKFLTENKPEEWTMLKAKYDPDNKYVQKYVADADKDGIKL

>AaegCSP15

MKFSIVVLALFAVAAAKPQDDKYTTKYDSIDIDEILKSDRLFKNYFNCLMDTGACTPEGNELKRVLPDSLENNCSKCSEKQQTSSTKIIKFLTENKPEEWTMLKAKYDPDNKYVQKYVADADKDGIKL

>AaegCSP16

MKFFIVVLALFAVAAARPQDDKYTTKYDSIDIDEILKSDRLFKNYFNCLMDTGACTPEGNELKRVLPDSLENNCSKCSEKQQTSSTKIIKFLTENKPEEWTMLKAKYDPDNKYVQKYVADADKDGIKL

>AaegCSP17

MKFFIVALVLIAVAAARPQDDKYTTKYDSIDIDEILKSDRLFKNYFNCLMDTGACTPEGNELKRVLPDSLENNCSKCSEKQQTSSTKIIKFLTENKPEEWTMLKAKYDPDNKYVQKYVADADKDGIKL

>AaegCSP18

MKFFIVALVLIAVAAARPQEDKYTTKYDSIDIDEILKSDRLFKNYFNCLMDTGACTPEGNELKRVLPDSLENNCSKCSEKQQTSSTKIIKFLTENKPEEWTMLKAKYDPDNKYVQKYVADADKDGIKL

>AaegCSP19

MKFFIVVLALFAVAAARPQEDKYTTKYDSIDIDEILKSDRLFKNYFNCLMDTGACTPEGNELKRVLPDSLENNCSKCSEKQQTSSTKIIKFLTENKPEEWTMLKAKYDPDNKYVQKYVADADKDGIKL

>AaegCSP20

MKFFIVALALIAVVAAQDDKYTTKYDSIDIDEILKSDRLFKNYFNCLMDTGACTPEGNELKRVLPDALENNCSKCSEKQQTSSTKIIKFLTENKPEAWTMLKAKYDPDNKYVAKYVADADKEGIKL

>AaegCSP21

MKFFIVALALIAVAAARPQDDKYTTKYDSIDIDEILKSDRLFKNYFNCLMDTGACTPEGNELKRVLPDALENNCSKCSEKQQTSSTKIIKFLTENKPEEWTMLKAKYDPDNKYVAKYVADADKKGIKL

>AaegCSP22

MKFFIVALALIAVAAAQDDKYTTKYDSIDIDEILKSDRLFKNYFNCLMDTGACTPEGNELKRVLPDALENNCSKCSEKQQTSSTKIIKFLTENKPEEWTMLKAKYDPDNKYVQKYVAEADKDGIKL

>AaegCSP23

MKFFIVALALFAVAAAQDDKYTTKYDSIDIDEILKSDRLFKNYFNCLMDTGACTPEGNELKRVLPDSLENNCSKCSEKQQTSSTKIIKFLTENKPEEWTMLKAKYDPDNKYVQKYVADADKDGIKL

>AaegCSP24

MKFFIVALALLAVVAAQDDKYTTKYDSVDIDEILKSERLFKNYYACLMDTGACTPDVNELKRVLPDALENNCAKCSEKQQNDSTKTIKYLTENKPEEWKALKAKYDPDNKYVEKYVADADKEGIKL

>AaegCSP25

MKIFIVALALIAVVAAQDDKYTTKYDSVDIDEILKSERLFKNYYACLMDTGACTPDVNELKRVLPDALENNCAKCSEKQQNDSTKTIKYLTENKPEEWKALKAKYDPDNKYVEKYVADADKEGIKL

>AaegCSP26

MKFFIFALALIALAAAKPQDDKYTTKYDSVDIDEILKSERLFKNYYACLMDTGACTPDVNELKRVLPDALENNCAKCSEKQQNDSTKTIKYLTENKPEEWKALKAKYDPDNKYVEKYVADADKEGIKL

>AaegCSP27

MKFFIVALALIALAAAKPQDDKYTTKYDSVDIDEILKSERLFKNYYACLMDTGACTPDVNELKRVLPDALENNCAKCSEKQQNDSTKTIKYLTENKPEEWKALKAKYDPDNKYVEKYVADADKEGIKL

>AaegCSP28

MKIFIVALALIALAAAKPQDDKYTTKYDSVDIDEILKSERLFKNYYACLMDTGACTPDVNELKRVLPEALENNCAKCSEKQQNDSTKTIKFLTENKPEEWKALKAKYDPDNKYVEKYVADADKEGIKL

>AaegCSP29

MKIFIVALALIALAAAKPQDDKYTTKYDSVDIDEILKSERLFKNYYACLMDTGACTPDVNELKRVLPEALENNCAKCSEKQQNDSTKTIKFLTENKPEEWKALKAKYDPDNKYVEKYVADADKEGIKL

>AaegCSP30

MKLFIVALALLAVVAAQDDKYTTKYDSVDIDEILKSERLFKNYYACLMDTGACTPDVNELKRVLPDALENNCAKCSEKQQNDSTKTIKYLTENKPEEWKALKAKYDPDNKYVEKYVADADKEGIKL

>AaegCSP31

MKIFIVALALIALAAAKPQDDKYTTKYDSVDIDEILKSERLFKNYYACLMDTGACTPDVNELKRVLPDALENNCAKCSEKQQNDSTKTIKYLTENKPEEWKALKAKYDPDNKYVEKYVADADKEGIKL

>AaegCSP32

MKIFILCAIMAVVAAQEATYNNRYDNIDVEEILKSDRLFKNYFNCLMDAGPCTPEGTDLKKYLPDALETGCTKCTEKQRDTGNKVIAWLIENRPMEWVMLKSKYDPENKLTERYRELAAKAGIAL

>AaegCSP33

MKLFIALALLAVAAAQEATYNSRYDNIDVEEILKSDRLFKNYFNCLMDAGPCTPEGTDLKKYLPDALETGCTKCTEKQRDTGNKVIAWLIENRPMEWVMLKSKYDPENKLTERYRELAAKAGIAL

>AaegCSP34

MKLFVAVFALIAVVAAQELYTSKFDNIDVDEILKSDRLFKNYYQCLMDEGRCTPEGNELKKILPEALETNCAKCSEKQRDGAIKAFGYLSENRPTEWKTLRDRFDPEGKYIEQYREEAEKNGIKF

>AaegCSP35

MKIMIVIAFALLAVASAQEEQYTTKYDNIDVEEILKSDRLFNNYFKCLMDEGPCTPDGNELKRILPEALQTNCAKCSESQRAGAIKVINYMIENRAEQWKALQEKYDPENIYLEQYRAEAEQSGITL

>AaegCSP36

MKSFIVIALALVVAVAAQNKYTSKYDGVDIDEILKSDRLFNNYYKCLLDQGRCTPDANELKRILPEALKTNCEKCSEKQREGATRVINYLIENRNQQWQTLQAKFDPENIYINQYRNEARAAGIKI

>AaegCSP37

MNSVNRYVLCIALIALFVASFTTAEENCEISANELGKIEQTLTHINQPIYTGDDESEVSDSDQCAQMLRGIHFQLRRLTQKYKLMNKGYVKAEEFAKMARDYEDQLSVLKNDLEQLKIGADSSAKQKMQELKKDIATLEQNVNTLHKDLEGITDELGKVRMDLCLTYMESNQLSNAQDKVKTLAPKYLMELVEQFLNKSEKNWLPVVDLSVAIPDLDDRGQVYKTVHEFLKTKNRDGGEDSILLEAEVLKMNATFHPGSKITEDRKKEIQDLLEKLSLTSTKIFDQWTQDLAKLENSAVYKNSIDRMFLTQMEKFGERVMAKDDYYSLRNFLKLLVVSTNYYKIAAYRKLIQEKIGHTLAVLMFDMMSMERTELQYDPHVPDEVVRMYDESITALPDSLKNIRSCLKLVKIYNHVTNQCILATNEVEDVNNSNPKFKSNVLGRRKLVKTASNDCTPFRLEPSADKASIRIITPKGDALTNINSIQPGLSWFNRVGAPYTNNHNMKLDYSADWILDANYANDSIKIESEFNAYQTMKSVDHLMVTNVGKVPHVVVAQYGLKGMEYAGAGMKDAEWKFKCDN

>AaegCSP38

MKLKVYICQVIFSFLAVSVFCEENCNIPESELSKIDHVLRHMEKPIYSEEQFASDNEECTNLLNGIHAQLRRLTQRYKLMNKGYVKVEEYQRMADDYEKQLKTLNDELVELQQHTSEKASATIAKLKEDIKKLDEEVGTLHEKLKGIKQDFEKVKRDLCVTYLNSNQMSKAKAKLKEMASTYLIEIVQQQLNKSNANIMPMLEFSAAIPDLDDMGEAYKEIYKFLEEQKRLEGEDSVLLEATVLKMNASLKEGSNITDERRTQIEGLLKDLATKSTIVFSTWTKELKKINDAVVIKNALDHMFVSQMKVFGALVGDTSDFGSIRNFVKLTVVCNNYYKVAAYKELIDRKIGNALGTIMFDLLTLEVNEMKFDPHVPDEIPKLFEATLSSLPNSLTELRTCLGKVQIYNKKTNKCVVATGNDFDVHKDKLGDFYRVVVADYGCTSFRLEASGDKASVRIVTPSGNPMSNVNLHLEGNSLHNYVATPKSNKPDRTPSSSDEWILDANYNNDTIKIESQFSDYKTKKTEVDHLLVRDINHLPHVLVARYGFMGLKNSDAKDTIEWNLKCGS

>AaegCSP39

MARGMGCWQPASQWGHKGFGFVTFQSEDVVDKVCEIHFHEINNKMVECKKAQPKEVMLPANLAKTRTAGRGTYDFMWSLGTLPDGFPAAAYAAYAAGRGFSGYPSFGLPYPTGNLNLAALHAHLAAAAATASAGGPHHHHHAHSSSSGNNTTPPPPTRSNPALVQLEATRIDCCLIPGGEHRTTYNSTNMHEMHFNPTSCRMRIVSLVGLVGSDRAYHPPPGTGAGESKLDKGIRHRSSIHSGAYTQLLCIRPNVCEGNYTRVDVAGEFVSTFPARFLDHYIVIVVVRRRSKALSSFDSTPPSTL

>AaegCSP40

MNRQLWIIIFAILCVAQAEEDNPTTEKMEELGIATINNFTREFYSYVEAVSQVLADLELTTTASITQIKHRIKHLLQEKCNLCSAKAEGPALDQGYVTTSNGSVIPVSYEQTRFGGGWIVLMQRYDGTVRFNRSWAEYRDGFGMVGHEFWLGLERIHQMTKDAEYELMIEMQDFEGNYKYAGYDAFAVGPEEERYPLAKVGKFNKTAYVDSFGKHRGYGFSTYDNDDNGCSNQYGRGGWWYYRKSCFGASLTGIWQNKQDWKSISWVWFSTEKKQVPLKFARMMMRLKTAE

>AaegCSP41

MILQFWVVTFSVLFAARADENHSILIKLNDLDHRFTQMFSQQFYRHTQQVTDRVSALKISIDTNLLELDQQIQQALDGIQSNESSSSASATKPPGLTTIPIGSEPRVPALYERERYGGDWLVVMHRYDGSVKFDRTWAEYRDGFGMVGQEFWYGLERLHQLTKEKSYELMVEMEDFNGSLKYAWYDKFVVGPEEQRYALVELGTFNGTTDGDSLKPHKGSGFSTYDNDDFGCSNKYAKGGWWYYSGKCYGSSLTGIWKNELAYSSIVWMKFSDVSNTPLKLVRMMIRPKN

>AaegCSP42

MVIQRVWRSSANSSTSGNTTATNSNSGALTASSLSATSSSELAPQNVPSGEIDGSQVVVPKMQRPKLSGEAMLEVMKMRYSGRSHSSQSAGDAALKSLELLRANIQYLFDKEIEVVVKKFSSLFFIPAIKNIKENLGESAISDDTLKTLYCSLLENSKSQYVGQIASPAESSLSRANTPGMELSDSDSSNDNVVPSGTTSLLQQALKRKLPEPNQHDGFKRQYFLQGSLYSQNHYSILQSLGNVQGSLPYQIRPSVLNPTVYTTTISPETLFIMDFKAGRALGVPDFRDRLANKHPEILRYCPDNQDRDWLLQQKQISPLNRNGRFFLLVLDEVRKLAERNSEYSNNPYMKLSDLQGFKLTEFIYAKVQKLIKDSADSSVKPTTATPSVTTATTAVPNSIQPRPRVSSLSSSHATLTALLSSPQQSQVNCSNSSGTIATIAGSSTTSGMDANTGGTGSGDTKT

>AaegCSP43

MFSKVKSLSTRGAASTCTVRLVLVVVFMLAISQVAAQSSSTTTPEATSSSGNNGSSPSTPNKSQVSDEALDKALNDKRYLMRQLKCALGEVPCDPVGKRLKSLAPFVLRGACPQCTPAELVQIKKTLAHLQRNFPAEWNKLVQTYAG

>DantCSP1

MKLLFAVVVAAFMATIVLADDKYTTKFDNIDVDEILKSDRLFNNYYKCLTDEGKCTPDGRELKKNLPDALQTECSKCSAKQKESSEKILNFIVENKPEEWKVLQAKYDPEGVYITKYREEAKKRGIKV

>DantCSP2

MFRLIWISLFISYLTFIQAVPHPPATTAAPLKQTYDNKFDNIDIDEILGQERLLKNYVKCLEGLGPCTPDGKMLKDIPILETLPDAMATNCAKCTERQKYGSDRVTHFLIDNRPEDWDRLEKIYNPEGSYKKAYLMEKQKLQPTNEDGDAKKD

**Table S4 Amino acid sequences of 318 OBPs and 138 CSPs of *B. odoriphaga* and other Diptera insect used in motif analyses.**

**Odorant binding proteins (OBPs)**

>BodoOBP1

MKSAVLLIVIGATLCLGQEIRRDKQYPPPEFLKALQPIHDVCVAKHGVTDEAIQQFSDGEIHEDEAMKCYMNCLFHEARVVDENGEVHLEMVHELLPESMKDIALNMGKKCLYPKGETQCDRAFWLHSCWKKADPKHYFLPGQHVWYLLE

>BodoOBP2

MKSSILLMIICATLCHSQEIRRDKQYPPPEFLKALQPIHDVCVAKHGVTDAAIQQFSDGEIHEDEAMKCYMNCLFHEAKVIDEAGELHYEKFVNLLPESMHDIALFMGKKCLYPKGETQCDRAFWLHSCWKKADPKHYFLPGQHIWYLLE

>BodoOBP3

MKTLTGLFIVCCGLVVIKASMTEEQQQAFQKLLKNCAEKEKASQADIDDFLASKPAVEPQAKCFRACLHETFGSMKNNKFSREGFLAVMKMKFEGDAEKIKIANEVADTCANEEDSDRCEAGAKICKCLGETSRAKGLIE

>BodoOBP4

MIFDCKLNLSIVLWLANTTLTAAFINDTWPPQVIIEAFDMVGFAEKCLKETNADIKKLEEFTFAHDLTNLPDDRSLKCYMACIFRQYQFLQPDNPRLQLQTLFNVFPAMTDDERSHFLKMASGCTKLRAKDPCDAVYIANVCMKRNANDYYYIPYDVEYWSNRNKNGK

>BodoOBP5

MKKYFHPFTTVTFLIISSNVAVIESAMTIQQLEYAGKAMRETCKPQFNVSDEIIDGLKIGQFPENDDLKCFVNCMMELMLLMRDGVSNIDAASKFFHLLPEDLREPYSNGMNACKNAADGVQGKCEVAYTLIKCLQKKNPVFAWP

>BodoOBP6

MAKSLKIIIVIAAIVTVCTAITDEQKKKASVHAEKCMEKTGVRRDDVTKVLMGDFSVTDKNIECFVKCFFEESGFMNAEGEFLDEVALEKLSMNADRNKAEALVNTCKLRMASTPCETAYKIYQCYIEHKAI

>BodoOBP7

MMKNTLSLISILFVFLVHKSEQADITEAQFMKTLSVFNSICQTRLSISDEQIAYVKQHPEELADDSKEKCFATCVLEMFNMVINGKVPGNLADESIGHFLPNKINEVAKSALDHCRNALDNTGNVHCVVGYNMLKCLAGKELKFFLL

>BodoOBP8

MKFFIAISLALVSTCFAADLRRDEKWPPKALLEYLEPIRISCMGKTGVTAEAIKEFSDGELHDDPKLKCYMNCVFNEAKVVDDKGDVHFEKIDTHIAQLDDEIRHIAENFLANCRSIKGDDPCERAFSVHKCWKLHDPKHYFLP

>BodoOBP9

MNRLVVVLTVVFITIVTSENSSSEEEKGENKFIELIECKNETGASDIDMGRVIIRANPITQAGKCLTACYLDKLGIMTQEKFVPENVMNYLDDVTLVGSNGKTLDKVVKEIADECKAISDTDKCESAAKILNCLNASAKKRGFCPNVF

>BodoOBP10

MTCRLVWLALLVYVNFGECGVTFEQVQKTGEMIRSVCQPRIKISTEMIDGIRKGQFPNDKLLKCYIHCAMEMSGIMKKNKVNYEAAIKTMEKLLSDDLLEENKNGLTKCKDSANGIKDNCEAGFVLVKCIYDNRNMNRDEFPFP

>BodoOBP11

MNHRQCLALLVCVNFFGVECGVTMEQMEKTSGMIRGVCQPRTKIATEMADGITQGQFPDDKALKCYMQCVLEMTGVMKKNKLNYEASLKQMDKLLPDDIVEENKNGITQCKDAANGVKDACEAAYILVQCMYKNNKKFTFA

>BodoOBP12

MTRRLVWLALLFYVNLGECGVTEQQAQKSREMIRSVCQPKVKIETEMADGIRQGNFPNDKALKCYTHCVMELSGIMKKNKVLYEAAMKQMEKLLSGDALEENRVSLTKCKDSAVGIKDNCEAGFVVTKCFYDNRSGKNEFKFP

>BodoOBP13

MRRMWFGLPISLALLLNIIGVSFCAMTRDQMIKSFNTIRNLCTPKFKLSSDQLNALRFGNFDNDDKEVKCYLLCTAQMTGVLTKKNEFHLEKVLKQIEAMYQSDNKQSAIDAAHHCKDVQKTYKDPCDKVFFTLKCVAEQMGKLFVWP

>BodoOBP14

MKYLIFSVLLATVVYADHDHWQIQTAENLQSYREVCVTEHGITPEQIAKYKSWNFPDDEKTHVYINCIFNKMGLFDDKTGFNIDHLVLQLGQNQNKDEVKAKIEKCADKNENKDSAAVWAFRGMKCFIAENLPLVQTSLKKPA

>BodoOBP15

MKLFYSIFLILLAALSLSADSAESNEIEALIVEKAKICQKETNASENDVDEMLDGAEPTTQTSKCLHSCLVKKLGVMNADNKLEPENLTKMVIDDTIKSSDGKTIKSLIKPISDACKSVTDPDECEAGVKIVDCLNAEAVKLGFKTGPF

>BodoOBP16

MSLSVKFLLLVCAIGVHAKPLSEEERQAMFTDLLTNCKAQEGASDSDIQEMINHQPPSTHAGQCLNACTMEAVGIMKDGRLSVEGSIKVGEMIGDAARIKNIEVVSRECESTVGSDRCDTALQIALCVNKSAAAHGIDPVKELM

>BodoOBP17

MKSVLVFALFAIVAVAHADDDGVLKEQIAQIIKECAEKTNFSDEGIKKLQAQDLSLTENVQCFQKCFFENGGIIKDKAFDNEKVSAIGRLYKQDEAKIKANLEKCIPLYKPDAFDCDAAWEIYKCFN

>BodoOBP18

MGKVLISLLLGLMWKVAVEAAIELPEHLKGPAKILRKTCQAETNVSEDLIEQSKGGYLPEDKALQCYIDCLFRTTGLYDAGGNIKFDDVYHIMPTEIKEKVDAVTAVCKTIHGSNQCERAWLTVKCYFDADPEHSLLP

>BodoOBP19

MYKFLFFVGFVSLTVVAYDFKDSEFNEYLMQDLLDYNQEVDDVFVSARIRRDTTTENKDEDKKCKSKKHGMWKCCNDNSNDKTFDKFKDMKKQCFQEIRGPKGEESLDMFSCEKVNKTKEEFVCVSQCVGQKLNIVDKDGNLIEAAVKEHVKNDISSSQWQKDVSDAVSDTCIAEAKEAVRANPPEAGKCSCAALKFSHCMWREMVKACPKDLQSDSNKCVKLREKLDKGEKIDFHQFHRYHHHRSDDSNDE

>BodoOBP20

MNFHCKIFILTNVLWLTAARRTDKWPPQIIVDTYGHALAQVRNCQEEYNIDARTIIEFEDSSDLNDLPNDRSLKCFLLCTYNAFKLVVPDSTKLQVVELLGAISKMEYDDRTSYLKMTSGCKLRSKDLCEAVYQLNVCMKKNNNDYYYLVDGSDYWIQRLQASSSHNSNDTLVTETASPSER

>BodoOBP21

MKYILCVICVSAVAILLVSVDAGKEEENMLKQVAEGCINETGATDADVTAMANKELPSTPTAKCFTFCVLKLFNCLTETGDFQQEEFMDLMRMGTDNNTVLTMCDEVSKKCVGTKSPDNDNCETAGLLVKCLDDGAKEAGYEKL

>BodoOBP22

MNLKLDLHSFGFVIFLTVIGDVFCAMTMKQLLKSMDMMRDTCSPKFSLSPETLAGLRKGVFLEDRELKCYTLCIAQMAGTITRKNEISLEKTIKQLESMLPPDIKQLAIETVTLCKDVQKQYKDPCDKTFYSAKCGYELQPDKFMFP

>BodoOBP23

MKYFAVVLLLVAAVSADEWTPKALPDLLAARKECVSKLSVPSETLEQYKTWNFPDDELTRSYVTCIFKEFGLFCDHEGFHTDRLITQFQTAHGVDIKPKIDECVTKTDADTTNEIWVFRAFKCFTAQHFSLVQSQLTKKTE

>BodoOBP24

MSFIVKIVLLACVLVVAAKPLTREEEEAMIAELVGKCKSSEGASDSDVQEALAHKPPTTRPGQCFNACVMENLGLMVGGKISVEGGVKLAERSGDPTKIKNSETVGKECQSVGGGDRCDSALAIMQCIAEASAKLGIDPSKDLH

>BodoOBP25

MMIPKLDLHSFRLFIFLTLIGDVFCAMTMKQLIKSMDMMRDTCAPKFSVTPETLAGLRNGIFLEDRELKCYTLCIAQMAGTITRKNEISLEKTIKQLESMLPPDVKQIAIDTVTLCKDVQKQYKDPCDKTFYSAKCGFELQPEKFMFP

>BodoOBP26

MILVRASIWFGFLLFIYAYKTDTFPPLTTVVKHFDLNYIVPNCYERSGVDKKELIEFEMTDDMEHLPNSTAIKTYLRCFGELSESLEVKSNKVLLGKLTAYFAELTKEQQEIYLGMVKGCVKRAKPITDLLEFPYNLTVCFKQNDNQHFYIFY

>BodoOBP27

MNSILVTVLFACLAGLVSTDEASDLKQKFQQVHLSCAARTLFNPVGIKNLIDLDFAAAQKIPNHDCFEKCVFQSIGTVTPTGLDNDKLLALAAIFQPQTVPQTQIQLVKCASLYDPNNFDCAAAWRLYLCLQNGESALSEPKAPTCLLVRAIWN

>BodoOBP28

MELKLLALVFCCVGFASCQAPRRDDKWPPPELIKFLKPIHDKCVSKIGVTEEAIKKFSDEEIHEDEKLKCYMNCVFHEGHVVDDNGELHLEKLAEGVEMLGPEVEAIALAMGKKCLKPEGETQCDRAFWYHKCWKTADPKHYFLI

>BodoOBP29

MKFIVVCVIVCAIALNFSLDFVYPAAVNTPQKYSASNFGTLLGQQHNERAKEITNAWEECNETYNVPLHEGIDLFQNPPWGEERYVDRMSKCLVDCTLRKTGVVTDCGLDEAEYMKQIKQLRFNYTSFNVNDESDNDKRKIWETIENRANSLPPLCYYVFSSDSFYTQIQHRIPTLNYQNRCYKLIQSYSKAARKCKDLKDPQGDDCETSWMIMRCIMDENRGQFYNSPFYPIKINALN

>BodoOBP30

MKFIFCVISLTAIVCLNFVQADYEQELFDNLRDDECFNKTQICTDKIKHVGIKVTEADRRGLCDANPLTTTGTKCLMDCQMEDMNWTNDKGFQKDKYLEFIKKTSLGQDKEYVQAAEKVAELCKKKKASFTDSCETAYIVLKCLQDEGNKLQRLNLGYSLFYVAPFFLEFE

>BodoOBP31

MKYFLLLLLVGLVYSDDDYVLKTRADVGESFRKCLSIQGVSRETFKNTDWTLSSTNPLVQTTIKCVVEDFGLFDETDGFNQDRVVKQFGGESGRTKVETCITDNPIGTPTVAQSVQNVMSCLEENGLKMYEDQPARVSTSE

>BodoOBP32

IIFVVALVSVFAISQADLSVSVSGVSWPNPGRTEPRPDEPTFSHYSLVEFLLVFQYEKRVAEIFDALKACNETWSVPRDEAIELFENPPPGLLRLTNKPAKCLIDCVIRKTGVMTDAGFQVDQYMLQFKQLRLNYTSYNLNSKPRAEKEEVLAAIKKFAEGNPKNYEMIEAYEWAANECKGRKDPDDDNCQTSWLIARCIINRAREKHFNAPFYPADLSYLK

>BodoOBP33

MIFVFILFGFLTHTISTLKSDTFPPMNKVRKHYDIDRIAKVCYEKSGVDKSELHKFEVTEDLQHIPNSHAIKVYLRCFAELSEAVAPDSNKVLLPKFMKYFDDLTPAEQLIYLNMGKGCLGRVSKITDLLEYSYKIAVCGKRNDNEHFHIFY

>BodoOBP34

MIAKLLHLSVLVTIISAKVDPICKLAAPTGVDPESCCKVPDILDHPTLEKCAIAVFNTNSSDVSSEVINVGSGKCLFECIFKEHGILKNGTVLPDVALKLLKEVIKSDEVWIPIVTNAIDTCQSMAASMPEKTMSPGTCDSLPAFYFGCFHTQLFKNCPNAKWTASEGCNQLKQNSEKCPVLTAVVSME

>BodoOBP35

MEKYIFVLSSVILVAFLSFSTVVAHPRAHPNFPDFEECKTAEKATDDDLKSLKDFNLPKTRGAKCVMTCLGEKTKKIADNKLNPEGVTNWFKVSPDYTEQMQQTVNEILEECRNITDQDRCEAGFKILECLKDGAMKRGLKVPFKRPSQ

>BodoOBP36

MKFIFVAIIVSALALSLCSCSNSTVNDSPFYKSLVTKDTFRDRIYFSTYFEHLLKEQFFQTQSVIVDAWKECSEHFYITDVDDAIDIFKNPPLEKNVKSHKKCLYNCIMNRTGVMTDDGFQEDQYIKTIKQLRLNYTSFNIDEKRNETKLKILKTVRRLAKGLNSFTMCSNRNSMSGRDDSEYLLESYARAAHKCKQVEYPEVLDRCEDSWNLMSCIIAQNQKRNFNSPFYPLWVKDLK

>BodoOBP37

MKLLFCVVFVSIAVASVTSTREELEKKTAECRKKISSMQDLSHQEMKCLGACYLEKLEVMDKANKLVPKKLDVLMDPTIKSNNGKFFKDVSKNIAEKCKTVTDKDKCEAALKIVTCIHDEAKKVGFNEDVMY

>BodoOBP38

MIFTRLIVTVIAATIWIGTANGEGCRIQKANPDVFSDAFGQPAAKTHDGKCIYDCMLRLSEVLKGGGLVDVDKVVKKLRAISSKDKLSSEEISKIEKAIKRCTNHRHISEDKCETAGIFVLCGSEEVAKEGLPPRTLVNALLAIYFDLPQRIIEESSPYGKGTFLFPVLWDKISEFVNSF

>BodoOBP39

MMKLICLILIVLTVERSYGLEDIEKILADCKTSEKVPDVEIMNFKNKVMSETKEAKCLTTCIMEKVELIKGNQINANGMMQLAKSMPNSNEKMLQSATEAVDECKPIKEADRCEYGFKVSKCLMDAAAKRQLSSIFA

>BodoOBP40

MMKLNKVVAVVAVFLIGFCGNALSDNAEMDKIVEICKAKEKATDADIEIWRSDPEPKPRMVKCLEACVGEQMGMMTTDNKISVDAVLKFYATETKDEKAVANMKEVLNECKAVTDKDRCESSAKILKCINDGTRKRGMKEMF

>BodoOBP41

MKYFILLLMVALVNLVHSEDGYVLKTRTDASETFRKCLSTLAVTRDVFKETEWSVDSDNPVVQATIKCVVKDFGLFDETIGFNRDRMVKQFGGEALRTRVEKCIDKYPIGVPTVDHSVQSVMNCLEEDNLKMYDNNTNVDV

>BodoOBP42

MKYLIGLILTTTMITANAVWQPPTKLQMIQAHDKCLRQSNLPPLVLTVSEFWNLSDDSSIRTIRDYVHCVLTTLHLFDDKSGFDVDRIMEQVTDIPYLKNTVIERSTIEKCADGNENNDLADVWAFRGFRCFMDIIKKRTNGIA

>BodoOBP43

MKHCLVVLCWIAAVLADEWTVKHSSDLKLARSKCATTLNVPDDAFSNYQQRIFSDSDPLAKAYIGCVFKELGVFGDNGFYVDRVVSQFVTEPADAARLEKCSEKIDTDTSDDIWAFRAFRCFTKVNEPEFYMQLDMTD

>BodoOBP44

MKNYISLILMFVLVANGDQEKWSVRTTRDGLYSYNNCVNKLNIPADDVLKIEKISDPLNRQILECVYNDLGFYTEGIGFRVDRIVQQFGGGPVMENVVTMCIPIGSSDPVADQIIVVEECFRMEKVGGYQE

>BodoOBP45

MKTLTGLFIVCCGLVVIKASMTEEQKQAFQKLLKNCAEKEKASQADIDDFLARTPAVEPQAKCFRACLHETLGSMKNNKFSREGFLAKVDITFEGDAEKIKIANEVADTCANEEDSDRCEAGAKICKCLIETSRAKGLIE

>BodoOBP46

MGKVLVALLLGLMWKVAVEAAIELPEHLKGPAKILRNTCKAETNVSEDLIEQSKGGYLPEDKALQCYIECLFRTVGLYDDAGNIVFDDVYHIMPTEIKEKVDAVTAVCKTIHGSNKCETAWLTVRCYFDADPEHSMLP

>BodoOBP47

MKSVLVFALFAMVAVAHANDDEALKETLGQVIKECAEKTNFSEEGIKKLHSNDFSLTENIQCFQKCFFEKSGIITDNAFDKNRLLAIRRVYKKDEATETLEKCASLYKPDAFDCDAAWEIFKCFN

>BodoOBP48

MQQFLCLTIVLLFAVVSNVQSDMSEEMEKLVADINICKNETGATDADIEELHNRNIPTTKVGKCFNKCIFNRIEILKDSKFSPEGLIKIMEMDKSNLRAKDGKTLLESMKDIIGECDKVTDTDECELTAKLRTCLNVAAKKREICPNVF

>BodoOBP49

MKSICFLLSLLLINSPKLVIGDGDDDAASILKKCQIEVDAPDEEVKLIVDKKMPTSRIGFCLLTCLYEHAGIIEDGQFVVENLNKLIGGSDGGSVGGRMAKQFIKEVTKDCKDVRNDDKCYLSRDIVLCAAESISKHASLLKEL

>DmelOBP8a

MAHCMYILLLLLLVVELTPPAIPVPMRSSPQSLALLRARDQCGRELTAAQRLQLDRMQFEDAAHVRHYLHCFWSRLQLWLDETGFQAQRIVQSFGGERRLNVEQALPAINGCNAKTSSRGSGAQTVVDWCFRAFVCVLATPVGEWYKRHMSDVINGNA

>DmelOBP18a

MKVVCSIAVLWICLITMWQSAGRVNAEGCLKHHNLTSAQVQAVAPSTPVADVPVAVKCYSRCLIQDYFGDDGKIDLQKVGKRGSQEDHVILSQCKQQFDGVTNLDTCDYPYLILQCYFKGKQSGTIAS

>DmelOBP19a

MKFHLLLVCVAISLGPIPQSEAGVTEEQMWSAGKLMRDVCLPKYPKVSVEVADNIRNGDIPNSKDTNCYINCILEMMQAIKKGKFQLESTLKQMDIMLPDSYKDEYRKGINLCKDSTVGLKNAPNCDPAHALLSCLKNNIKVFVFP

>DmelOBP19b

MTNLLLAVACAAVLMGSATADEEEGSMTVDEVVELIEPFGDACTPKPSRENIVEMVLNKEDAKHETKCFRHCMLEQFELMPEDQLQYNEDKTVDMINMMFPDREDDGRRIVKTCNEELKAEQDKCEAAHGIAMCMLREMRSSGFKIPEIKE

>DmelOBP19c

MKPSTPVAAIPLMTIVVAVLLQTHCVRGQTQAFDLAKLLPKTGTEPIWAVIDRNLPQVQELVTAARMECIQKLQLPRDQRPLGKVTNPSEKEKCLVECVLKKIKLMDADNKLNVGQVEKLTSLVTQDNKMAIAVSSSMAQACSRGISSKNPCEVAHLFNQCISRQLERNNVKLVW

>DmelOBP19d

MSHLVHLTVLLLVGILCLGATSAKPHEEINRDHLLELANECKAETGATDEDVEQLMSHDLPERHEAKCLRACVMKKLQIMDESGKLNKEHAIELVKVMSKHDAEKEDAPAEVVAKCEAIETPEDHCDAAFAYEECIYEQMREHGLELEEH

>DmelOBP28a

MQSTPIILVAIVLLGAALVRAFDEKEALAKLMESAESCMPEVGATDADLQEMVKKQPASTYAGKCLRACVMKNIGILDANGKLDTEAGHEKAKQYTGNDPAKLKIALDIGETCAAITVPDDHCEAAEAYGTCFRGEAKKHGLL

>DmelOBP44a

MKNAVAILLCALLGLASASDYKLRTAEDLQSARKECAASSKVTEALIAKYKTFDYPDDDITRNYIQCIFVKFDLFDEAKGFKVENLVAQLGQGKEDKAALKADIEKCADKNEQKSPANEWAFRGFKCFLGKNLPLVQAAVQKN

>DmelOBP46a

MCSQLFAFLLLLLTAFVTGRSTPPALDEDCELNSVDTMHDFCCDLHDESPQFSDCQMEWHEKIPYETDEEEQTYMFCTAECSFNSTNFLGRDRRSLNLNEVKEHLESDLVNDADIKLLYDTYVKCDKHALSLMPHKGVKQLSKRLSRLGCHPYPGLVLECVANEMILHCPTKRFRQTAQCEETRNHLKQCMQYLKYKS

>DmelOBP47a

MNRVLVLLLVLKMFALSESRFAKININLGLTVADESPKTITEEMIRLCGDQTDISLRELNKLQREDFSDPSESVQCFTHCLYEQMGLMHDGVFVERDLFGLLSDVSNTDYWPERQCHAIRGNNKCETAYRIHQCQQQLKQQQQNLLATKEVEVTTTPAGSDETKP

>DmelOBP47b

MSPSQLLVIFASLALNTRLVFGQATIDCQRPPQLVDPALCCKDGGRDQVAEQCAQRILGTANGQKAGGPPSLDTAACLAECILTSSKYIDEPQKLNLANIRSDLSAKFSNDTLYVETMTMAFSKCEPQSQRRLAMIMQQQQQVQQQKTQQQQPRCSPFSAIVLGCTYMEYFKNCPDHRWTPNAQCTLAKAYVTQCGLGA

>DmelOBP49a

MLSKSQLLLLVVGFCLNAAVSADVDCSKRPSFVNPKTCCPMPDFVTAELKQKCIKFDMTPPPPPDGEASGSFESKRRHHHPHPPPCFFSCIFNETGIYQNRKLDEAKLNAYLQEVFEDSSDLQTTATQAFTTCATKVADFEANLPPRPAPSPPPGFPMCPHDAGHLMGCVFRNMMKNCPDSIRNDSQQCTDMKEFFTKCKPPRGPPPSAEDM

>DmelOBP50a

MRTGRILVALIFLGLIIPFRAAKCRAAPKSVQNVHVCCSAPLPNWGVFNRECHKSAIQASVSINRISKSKVNLANFLIKCRLDCDFNASSVLQGNRLIQAKVRPMLERAFSNEPTIDAYESNFAKCSTVVRSKYQELSPLSRQSDACDRHALFYSLCAYARLIFTCPDKMWQRNNRMCQEAKAYAKKCPWPALKMFMRNT

>DmelOBP50b

MSSVLHLLGFLWLPLLVYSVSNDMGGLQKCTELLNTHKLVYCCGKSFLDKFPFVGSNCTPFWDDYGPCRYECLYRHWDLLDQDNKIKKPELYLMITSLYSPLNGYDKYGAAFKAAHETCEALGSRHADFLLLYSNQVADKMGMASSTCLPYAMLHAQCTMVYLTANCPRENWIDDPKCNSLQKLLSSCTKKLDEKTNALKGKDEELTDNGCGHIDSEGSNLLMACFLTLMIAKFISDH

>DmelOBP50c

MARHIALLICSLLAMAGCDPIDVDCTRRQDFNIVKDCCVYPTFRFDQFKSQCGKYMPVGAPRISPCLYECIFNKTNTVVDGAIHPDNARLMLEKLFGNQDFEEAYFNGLMGCSDSVQEMISNRRSRPQRKTEQCSPFSLFYGICAQRYVFNHCPSSSWSGTESCEMARLQNMNCSKPSRGSSHRL

>DmelOBP50d

MLHKLTWVLIFIPAFRAADPICSQRPDVTALRNCCKLPNLDFSSFNSKCSQYLVNGVHISPCSFECIFRAANALNGTHLVMENIEKMMKTILGSDEFVHVYLDGFRSCGNQEKVLIKAMKRRRVPITGKCGSMAIMYGLCAHRYVYRNCPESVWSKSATCNEAREYSIRCDDM

>DmelOBP50e

MHKYIICFGFLLIILECSLASFNCSAPPNFNNFDINTCCRTPELDMGDVPQKCHKYVSGLKSANSKYPSYAHLCYPDCIYRETGAMVNGKIKVNRVKQYLEEHVHRRDQEIVSHIVQSFESCLSNVKGHMKSLNIESYKVLPHGCSPFAGIIYSCVNAETFLNCPQQMWKNEKPCNLAKQFAEQCNPLPHVPLPSS

>DmelOBP51a

MKVFIGLVLLLAVTTLSSALFESEANECAKKLGITPDYFENFPHSSRVKCFYHCQMEKLEIIANGVVTPFDLKVLNISPESYDKYGVKVKPCLKLSHRDKCELGYLVFQCLKREFNL

>DmelOBP56a

MNSYFVIALSALFVTLAVGSSLNLSDEQKDLAKQHREQCAEEVKLTEEEKAKVNAKDFNNPTENIKCFANCFFEKVGTLKDGELQESVVLEKLGALIGEEKTKAALEKCRTIKGENKCDTASKLYDCFESFKPAPEAKA

>DmelOBP56b

MKLIYLLVVFLIFALSELVAGQSAAELAAYKQIQQACIKELNIAASDANLLTTDKEVANPSESVKCYHSCVYKKLGLLGDDGKPNTDKIVKLAQIRFSSLPVDKLKSLLTSCGTTKSAATCDFVYNYEKCVVKGISA

>DmelOBP56c

MYFRASLMALLCLTLSEFVSKAWVMFFIFYISFTRSLSVSLNMSMTRTLVPDPPNGTENKLSQEMLRACMRRTEISMSQLKLFHMSLMNSDYNNDNDIAPTPVQSIGDVNNLGDLDFNGNSQMPYLDLKHNEPLQCFVSCLYETLDLDRYNVLLEEAFKNQVQTIIQHEKAEIKECSDLQGKTRCEAAYKLHLCYNHLKTLEAEQRIREILERTEAENEGFGPEGSDFIDGIQHSGEAMTTAKSE

>DmelOBP56d

MKFLIVLSVILAISAAELQLSDEQKAVAHANGALCAQQEGITKDQAIALRNGNFDDSDPKVKCFANCFLEKIGFLINGEVQPDVVLAKLGPLAGEDAVKAVQAKCDATKGADKCDTAYQLFECYYKNRAHI

>DmelOBP56e

MKVFFVFAALAALSLASAVGLTDSQKAEAKQRAKACVKQEGITKEQAIALRSGNFADSDPKVKCFANCFLEQTGLVANGQIKPDVVLAKLGPIAGEANVKEVQAKCDSTKGADKCDTSYLLYKCYYENHAQF

>DmelOBP56f

MKVFLLFIFISAIWLQAFCMKSSEKIKACLKRQLGYTITENTKFDAKEDSLQSKCFYHCLLEVKGVIANDAISSEQPRKVLEKKYGITDTDELEKAEEKCHSIKASGKCELGYEILKCYQSITKH

>DmelOBP56g

MRATFALTLLLGCLSGILAQQANIDSSVSKELVTDCLKENGVTPQDLADLQSGKVKAEDAKDNVKCSSQCILVKSGFMDSTGKLLTDKIKSYYANSNFKDVIEKDLDRCSAVKGANACDTAFKILSCFQAAN

>DmelOBP56h

MKFTLFCIALAAFLSMGQCNPDFRQIMQQCMETNQVTEADLKEFMASGMQSSAKENLKCYTKCLMEKQGHLTNGQFNAQAMLDTLKNVPQIKDKMDEISSGVNACKDIKGTNDCDTAFKVTMCLKEHKAIPGHH

>DmelOBP56i

MHFFTCCALLLVVVTLPTCFVQAGPIKDQCMAAAGITAQDVANRHETDDPGHSVKCFFRCFLENIGIIADNQIIPGAFDRVLGHIVTAEAVERMEATCNMIKSETSHDESCEFAWQISECYEGVRLSDVKKGQRTRNHRG

>DmelOBP57a

MLKLWLICILTVSVVSIQSLSLLEETNYVSDCLASNNISQAEFQELIDRNSSEEDDLENTDRRYKCFIHCLAEKGNLLDTNGYLDVDKIDQIEPVSDELREILYDCKKIYDEEEDHCEYAFKMVTCLTESFEQSDEVTEAGKNTNKLNE

>DmelOBP57b

MFIYRLVFIAPLILLLFSLAKARHPFDIFHWNWQDFQECLQVNNITIGEYEKYARHETLDYLLNEKVDLRYKCNIKCQLERDSTKWLNAQGRMDLDLMNTTDKASKSITKCMEKAPEELCAYSFRLVMCAFKAGHPVIDSE

>DmelOBP57c

MFNTRLAIFLLLIVVSLSQAKESQPFDFFEGTYDDFIDCLRINNITIEEYEKFDDTDNLDNVLKENVELKHKCNIKCQLEREPTKWLNARGEVDLKSMKATSETAVSISKCMEKAPQETCAYVYKLVICAFKSGHSVIKFDSYEQIQEETAGLIAEQQADLFDYDTIDL

>DmelOBP57d

MLDQLTLCLLLNFLCANVLANTSVFNPCVSQNELSEYEAHQVMENWPVPPIDRAYKCFLTCVLLDLGLIDERGNVQIDKYMKSGVVDWQWVAIELVTCRIEFSDERDLCELSYGIFNCFKDVKLAAEKYVSISNAK

>DmelOBP57e

MSLRLVPHLACIIFILEIQFRIADSNDPCPHNQGIDEDIAESILGDWPANVDLTSVKRSHKCYVTCILQYYNIVTASGEIFLDKYYDTGVIDELAVAPKINRCRYEFRMETDYCSRIFAIFNCLRQEILTKS

>DmelOBP58a

MKQLIFLLICLSCGTCSIYALKCRSQEGLSEAELKRTVRNCMHRQDEDEDRGRGGQGRQGNGYEYGYGMDHDQEEQDRNPGNRGGYGNRRQRGLRQSDGRNHTSNDGGQCVAQCFFEEMNMVDGNGMPDRRKVSYLLTKDLRDRELRNFFTDTVQQCFRYLESNGRGRHHKCSAARELVKCMSEYAKAQCEDWEEHGNMLFN

>DmelOBP58b

MLRIGFVICVIISLRLNGLVAVRVHCRHMERIHEENIHHCCKHQDGHDDVTESCAKQTNFRLPSPNEEAIVDVTVDQAMVGTCWAKCVFDHYNLMENNTLDMDKVRSYYKRYHQTDPEYATEMLNAYEKCHTQSEEATEKFLSLPIVRAFSTAKFCKPTSSIIMSCVIYNFFHNCPASRWSNTTECVETLAFARKCKDVLTT

>DmelOBP58c

MKCTILLSFFSLIWFAGGIKIDCENTEAINEDHIHYCCKHPDGHNDLIEGCARETNFTLPNQNEEALVDITADRAIRGTCFGKCVFSKLNLMKDNNLDMDAVRSLFTERFPDDPEYAKEMINAFDHCHGKSEENTSMFLSKPLFKQMSKQFCDPKSSVVLACVIRQFFHNCPADRWSKTKECEDTLAFSKKCQDSLATL

>DmelOBP58d

MVNIVCYWTFLILVAVSKAQDNEETTAVAISSGDLTEDKCNTSRAGCCSELYIGEEEDLVKCFVIHSPKLPVDGDADIGKTLRFLSCFVECLYKQKKYIGKSDTINMKMVKLDAEKTFVDRPKEKDYHIAMFEFCRKDAVGVYNLLKASPGAKVLLKGACRPYLLMVFMCISDYHQKHECPYFRWEGTAKAGTKDMCENAKAECYQIDGITLPTKSPA

>DmelOBP69a

MVARHFSFFLALLILYDLIPSNQGVEINPTIIKQVRKLRMRCLNQTGASVDVIDKSVKNRILPTDPEIKCFLYCMFDMFGLIDSQNIMHLEALLEVLPEEIYKTINGLVSSCGTQKGKDGCDTAYETVKCYIAVNGKFIWEEIIVLLG

>DmelOBP76a

MKHWKRRSSAVFAIVLQVLVLLLPDPAVAMTMEQFLTSLDMIRSGCAPKFKLKTEDLDRLRVGDFNFPPSQDLMCYTKCVSLMAGTVNKKGEFNAPKALAQLPHLVPPEMMEMSRKSVEACRDTHKQFKESCERVYQTAKCFSENADGQFMWP

>DmelOBP83a

MALNGFGRRVSASVLLIALSLLSGALILPPAAAQRDENYPPPGILKMAKPFHDACVEKTGVTEAAIKEFSDGEIHEDEKLKCYMNCFFHEIEVVDDNGDVHLEKLFATVPLSMRDKLMEMSKGCVHPEGDTLCHKAWWFHQCWKKADPKHYFLP

>DmelOBP83b

MVKYPLILLLIGCAAAQEPRRDGEWPPPAILKLGKHFHDICAPKTGVTDEAIKEFSDGQIHEDEALKCYMNCLFHEFEVVDDNGDVHMEKVLNAIPGEKLRNIMMEASKGCIHPEGDTLCHKAWWFHQCWKKADPVHYFLV

>DmelOBP83c

MQMKSGILIALCLCLSLNEGLALLEHEGETINRCIQNYGGLTAENAERLERFKEWSDSYEEIPCFTRCYLSEMFDFYNNLTGFNKDGIVGVFGRPVYEACRKKLELPFESGESSCKHAYEGFHCITNKEF

>DmelOBP83d

MESHPFTVIDNMPNISPSAKDAMKDCLQDVHQDEWKSFDAFAYYPVNEPIPCFTRCFVDKLHIFEEKTRLWKLEAMKQNLGIPAKGARIRTCHRHRGRDRCATYYKQFTCYAMAV

>DmelOBP83e

MSSPRAVLVSLFLICSQALADLSGDAQTLEKCLRQLSSPESIAGDLRKLERYSSWTREEVPCLMRCLAREKGWFDVEENKWRLKQLTEDLGADVYNYCRFELRRMGSDGCSFAYRGLRCLKQAEMHAGTSLSTLLQCSRQLNATNVELLQYSKLKSKEPIPCLFQCFADAMGFYDPDGNWRLENWKQAFGPSGNEDQSSGADYSGCRLSGTQREVALSKCSWMYHEYKCWERVNGNKLVEDNEEQ

>DmelOBP83f

MQSQSLLLIVAAVATFLVAQVRAQWLPLLMETTAKFLLKDHADAEKAFEECREDYYVPDDIYEKYLNYEFPAHRRTSCFVKCFLEKLELFSEKKGFDERAMIAQFTSKSSKDLSTVQHGLEKCIDHNEAESDVCTWANRVFSCWLPINRHVVRKVFA

>DmelOBP84a

MYSALVRACAVIAFLILSPNCARALQDHAKDNGDIFIINYDSFDGDVDDISTTTSAPREADYVDFDEVNRNCNASFITSMTNVLQFNNTGDLPDDKDKVTSMCYFHCFFEKSGLMTDYKLNTDLVRKYVWPATGDSVEACEAEGKDETNACMRGYAIVKCVFTRALTDARNKPTV

>DmelOBP85a

MSPGSVVFSMFLTRPSLDKGNSECRKSLNLPAHRKFNFAELYTINMCIEECNFIGCGYIEIDPPFRLDLANIRTNLQTIAPQPQNESIPFLVDAYRKCELFRSSHGRRFTLHLPDIEFIEEPCNPFALQITICVRIHAMQKCPSEFYVDSDECRLAREYFTQCVGDIETNLA

>DmelOBP93a

MKTSNKIVFLLLQLNIWQLSSCCDVQKNDKAINSCRKSLLGNNSTNSNGEVRNLKSDKVALHACIAECSFRTNGFLLSNGTVNTQALQKSYQQRYKNDPNMSQLMLKSLNSCTDYARKRVQEFQWMPKKGDCDFYPATLLACVMEKVYINCPTSKWKNTSDCTAMWKYLVACDDVASNKKK

>DmelOBP99a

MKVFVAICVLIGLASADYVVKNRHDMLAYRDECVKELAVPVDLVEKYQKWEYPNDAKTQCYIKCVFTKWGLFDVQSGFNVENIHQQLVGNHADHNEAFHASLAACVDKNEQGSNACEWAYRGATCLLKENLAQIQKSLAPKA

>DmelOBP99b

MLKYLIVALALCAVAHADDWTPKTGEEIRKIRVDCLKENPLSNDQISQLKNLIFPNEPDVRQYLTCSAIKLGIFCDQQGYHADRLAKQFKMDLSEEEALQIAQSCVDDNAQKNPTDVWAFRGHQCMMASKIGDKVRAFVKAKAEEAKKKAA

>DmelOBP93c

MNHLRLEIICWSCLLIAMAVSTEAASVWKLPTAQMVYEDLEKCRQESQEEDAATLRCLVKKLGLWTDESGYNARRIAKIFAGHNQMEELMLVVEHCNRMEQDTSHLDDWAFLAYRCATSGQFGHWVKDFMSQKEVER

>DmelOBP93d

MKVLIVLLLGLAFVLADHHHHHHDYVVKTHEDLTNYRTQCVEKVHASEELVEKYKKWQYPDDAVTHCYLECIFQKFGFYDTEHGFDVHKIHIQLAGPGVEVHESDEVHQKIAHCAETHSKEGDSCSKAYHAGMCFMNSNLQLVQHSVKV

>AaegOBP1

MNGSVVFVLSALVSLSVGDVTPRRDAEYPPPEFLEAMKPLREICIKKTGVTEEAIIEFSDGKVHEDENLKCYMNCLFHEAKVVDDTGHVHLEKLHDALPDSMHDIALHMGKRCLYPEGENLCEKAFWLHKCWKESDPKHYFLI

>AaegOBP2

MMEQLMLAVLLAVFLGLVADVTMAAQIKDNLELPEYYKRPAKILHNICLAESGAMESKLKQCMDGVLHDDREVKCYIHCLFDKVDVIDEATGQILLDRLAPLAPDNDVKDVFNHLTKECGHIKLQDSCDTAYEVAKCYFAAHDQVVKFCHLLMADVTS

>AaegOBP3

MIRFIVFVSSCLVAVSIADVTPRRDAEYPPPELLQALKPLRDICQKKTGVSDEAILEFSDGKVHEDEKLKCYMNCLFHEAKVVDDTGHVHLEKLHDALPDSMRDIAMHMGKRCLYPEGENLCEKAFWLHKCWKESDPKHYFLI

>AaegOBP4

MGLHKVKLLFHVLLAVMLSLHTSESKSTMEQLAKASEMMRGVCVGKTKAPLDLIDGLGRGEFVENKDLKCYANCVLEMMQAMRKGKVNADSAIKQVDLLIPPEIGEPTKKAFDMCRNSADGIKNNCEAAWALVKCLHQKNPKYFFA

>AaegOBP5

MFQKFWILLAFVGPALTHSDDYYDSNRRSRGYNERCYQHEQFPEPSECCTRPLWINQYLVRPCRFSNVQRDGYRQEHEACSVSCGVYRINMEMLNNQVNSVRIFRPARLRAYGDEDWKRTVAAALKLCKKRITSMVGSRAREGREAELCEEANDVFADCLDGQLFLQCPARVFIRTEGCELAKSHLLDGCPYRSLTDTTERHRNDWYDRNQWNGASNGGYDQRNGDQYDDEYEDVNSGHQTRNGGGYNNQNQNGYDRHNNDRNRNNGQW

>AaegOBP6

MKRLASLLLLFLTVRAEVSTQHCAVAKLPCTLHSECLQYLNSNDDGPENCAYRCIALTARFWDDQKADVIRTISRFYLTDANDDDFRNRTEQCLQETQETFPVTESCQRASCAFSCYNDQFGEVIAVRPSFIPFTALEHRRIVRECVDILQIGPQSRQAILDEGLMEVPEGRCLLRCVLLREGLYNDWRGPRLGSLWVQTEGYEDRFFDTAQKCYPLLKMQTLEPCELAARFAAECLPSRVPFVETVFAALASNQ

>AaegOBP7

MILHLRILFAVIALVSFCSCNQHKIVQKSLGKANDECVSYGSSEQCLARCVTLVTRDWNETVGLSSVYDRFYQPDPEDLCNTNRTQRCLEALQSTVAPEDKCLRAAGSVQCYLDQYGQVDMATSRFVKSAPVQQQQIIWECGAMLGYSGDQILRSIDDKDYSMQETRCLYRCYLIRSGMYTDEGGLNMERFYVACGGYEDEFYRNVTECAARVRSSTRCDDRCTLAQRLASECIGTRYDQTLTPGPATIDARDGSSVTYAVFQNYAGRDMTNTFVLNQR

>AaegOBP8

MKCLVLISLLAVGSQAFFTPEQHEVAKRLTMACATEIGEGLPDNVGNRFREGDLTLTDDKSKCFMKCVFGKVGFIDDAGTVNKEVLVEKLSKGNTQAKAEMFAEKCNMFEGANGCEKAHGLFECYWKNKEIFA

>AaegOBP9

MLKLVVALLSVTIALNQIKAFTLQQRQQGDIYAIECIAETGVNPASVALLRVGDFSSNDKRSKCFIRCFFEKEGFMDSKGNLHTEKIADALAGDFNREKVETVLANCLTKEKTACETAFRMYECFYNHREGL

>AaegOBP10

MTSFRLANLTVFLVLLFCFMRGVHSADDLSKIPEIKGYELHCIEASGITESSAKKLRNGDDIASPDQSIKCYVQCFFSKLRLMNEKGVVQKDKVLSLLGKLMEEDKAKKLAEKCDLRRTNPCDTAYAMYDCYRQNKAKLL

>AaegOBP11

MRLLISIVSFALVGAALSVPQQANLEDIGKIRNGETYALECLLASGLDVSSLKSLQTGDFSNGDRVKCLVKCFFEKTGFMDAEGNLNEEAIVTQLSQFMPKDQVETLVKNCKIEGTDACDTAYQATECYFKNKAGLF

>AaegOBP12

MKSFVCVVLVAALIGVNALTEEQLKKADEFASECLEKSNGLSKETVGKLRSGDFANVDQDSKCFVKCFLERAGFMSTDGNLVADYAIERLSLDREKSKVEALVMKCSMQMDDPCETAFRAFECYYNGKASLL

>AaegOBP13

MKTFAAIVSFALIAGCMAVTEDQKEAARQLAGKCMQQTGTSEESVQRLRNGDTSGADDNTKCFVQCFFQGAGVVDGEGNMQEAFVTEKLASEYGQAKAEEVVQRCRNNSGANACERSFSLLQCYIANRASLM

>AaegOBP14

MKTIAAIASFALIVGCMAVTEEQKEAARQLAGKCMQQTGTSEESVQRLRNGDTSGADDNTKCFVQCFFQGAGVVDGEGNMQEAFVTEKLASEYGQAKAEEVVQRCRNNSGANACERSFSLLQCYIANRASLM*

>AaegOBP15

MKSLVASGVALLMLASSIVPGCAQDFKGAIDECTKEFEMDMDVVVSLKYGDFSERDPLIECFTECLMKRSGFMFDDFSYNKTLIIGFAGRYLEPEGAQYVYDNCVDKFGTTVCVTGFEMYQCIHETAVAEWVESNF

>AaegOBP16

MIVIVVLVALSSEVLGAGQHDAVFKSIESTGKECARYLNNDGTGDCNTHCVGVIDHVWNDTVAMFTRNYERFFVPAPEDLCYQNRTQRCLSQVDQVVPVSDKCARARQLGQCYADQYGQLNASQLQYRPMTNLQYNRVFQQCSSMLGLSNDVLKDIATKGVDSVPAFACLVRCTMIRMGLYSDNEGFDLTLATGQCGKYNPALDPVPCQAKVKAEECDRCKRTVRIANECLGLRLNVKQLEENQPPQLVFNIEVYDFNCIILCDFEINF

>AaegOBP17

MKSIASAVLLLIFSVLVHSQSIKDLVEECKQTVPISEELEKSFLKLEFPPEEKTTHCLLDCIGKSLKVMDEKSGINLAVVTKLLQEVEPEGVIGEEQVRCATEAATSKEDQCTMAFKLYQCFEKEFLALMKMKLDQGE

>AaegOBP18

MTTHRLFIAATLLVLLVSLAYASEVLTKRQQYDQHKLMCGKIVRSTKEDRELYSQSQYPETHDTACFLRCVSILSGSYDDETGVNLDVLYDVYGKGTTAEEYAEESKACLALRDEVECYCMKAYKPLMCLREQFKKRNTA

>AaegOBP19

MLKAMTSNRCWIAVAIVCLMGVAAQAGPDFRTKREQYDHSKQMCGKILRTPAADLEHYLRSDYPESHDTACFIRCVSILNGGYDDETGVNMGVLFETYGGSLTKEEYADEAKECLALRDEVECYCMKAYKPILCLKEQFKKRNVL

>AaegOBP20

MKPGVKLSLLLLIGLMALLDSTSGCSMTNNDGVEQREALLADPSTAPARSMKDYSVEDIYADCNKTFAISMDFLNELNDTGSFPDETDKTPMCFIRCFLQKGEIVTSDDKINKEQAVALGWVKNGETIDDCLQELTGNPCERAYFLMRCVSTRHLVEGRSKDSKKR

>AaegOBP21

MQIECIIVLTSLIAAASAGWRLQTVDDLLRNRNKCVKILNMQDDLQEEFGLFDFPDQDSAKCVFKCIMNRMGLFSDKRGPHVGRLVKQMKFASMSSTKAIRDEILNCAYQDMEMDPEDVCDRAYALYQCIQNSNLLQLKSPETVKT

>AaegOBP22

MKVFIAVFALIAVAAAEFTVSTTEDLQRYRTECVSSLNIPADYVEKFKKWEFPEDDTTMCYIKCVFNKMQLFDDTEGPLVDNLVHQLAHGRDAEEVRTEVLKCVDKNTDNNACHWAFRGFKCFQKNNLSLIKASIKKD

>AaegOBP23

MLKLVLCLSALGLVACYDFKDSFYNELVLEEILDSEDAPSLMDRFKRSNPEMMDDKCKRNHRHKCCNDANGENMDKFRETKKQCFNEVRSKDRSARGMMNPVDMFDCEKMNKTKQEYICAVECVGRKFDIIDKDGNLLTTDKLVKFTKDNFAADPWQETVVDGLVESCLKEVAEKNEKMKSSGEHTTCNPSSSNFGYCMWRQMTLACPKDKQDTSKKCERMREKFANNESFSMYHKHDFDDK

>AaegOBP24

MNKLILVAIVATVAIGTSQAFVPVFHRFRRSPSVRCCNDGFEDSINHEKVVAVRRTCAEELGLNEMSEEELLKNRENLVCLVECIAKKHELADETGDLLHEDLAKAVKEHFSVAEWKAPLLDDFIKQCFDHAEEEHEKHPTEGGKCNPEGFDFSYCLWRHFTLACPEELQDDSERCEAIRRKLKSDEDVGFWNNDFDETK

>AaegOBP25

MNKLTLVAIVATVAIGTSQAFVPVFHRFRRSPSVRCCNDGFEDSINHEKVIAIRRTCAEELGLNEMSEEELLKNRENLVCLVECIAKKHELADETGDLLHEDLAKAVKEHFSVAEWKAPLLDDFIKQCFDHAEEEHEKHPTEEGKCNPEGFDFSYCLWRHFTLACPEELQDDSERCEAIRGKLKSDEDVGFWNNDLDETK

>AaegOBP26

MADDMAYQHFKMCDKATKKSEPPVSSENQSHLSLPIRVDAKRLQRRDDDDADQLFANQLTGSAASQQVQVKLGTYKNDKQNSLIIHSGGNCRTSITPTTREIGESEKLCRMLKLVLCLSALGLVACYDFKDSFYNELVLEEILDSEDAPSLMDRFKRSNPEMMDDKCKRNHRHKCCNDANGENMDKFRETKKQCFNEVRSKDRSARGMMNPVDMFDCEKMNKTKQEYICAVECVGRKFDIIDKDGNLLTTDKLVKFTKDNFAADPWQETVVDGLVESCLKEVAEKNEKMKSSGEHTTCNPSSSNFGYCMWRQMTLACPKDKQDTSKKCERMREKFANNESFSMYHKHDFDDK

>AaegOBP27

MKTLSVIILGAWLVHLGGVMSSMTFEDMQETAKMMRGICQPKYGIPDDVAENASSGVFPDSREFKCYASCLMDLTHTAKRGKLNYEAAVKQITMLPDDFREPFRVGLDSCRNAADGIDDYCEVAYTLLKCFFKASPKFFFP

>AaegOBP28

MKVFAGLLIAAIAASASAVYYPPLAPSDVEESHFAYQLKSFRQELDECAEYLQVSPGSVENLVAYNYVTDDPSLKCLIRCAGINAGWWSVGGNNSGLQPPVIESYFAPGCDDTCYVKRTQDCVSANVVPCQDDCSQAYQAFLCYYHQYGNLKSSEEYIPLPQLDAVQAAIDCMLILRTPKELLEQYVQGVFPDVPETQCLYRCQYLAEGLYDGVTFNLTRNYIREYAVPSPQIKDPATQACVDSALSSSSCNECARFWAGRGCLKNYGVPNHSGSYFQVAAGLVLNQRTCLDEDLNPHIFTCCSDVHGSCKAACESNLSKKL

>AaegOBP29

QQDAVQAAIDCILTLRIPKELLEQYAKGVVPHLKSFRQELDECAEYLQVSPGSVENLVAYNYVTDDPSLKCLIRCAGINAGWWSVGGNNSGLQPPVIESYFAPGCDDTCYVKRTQDCVSANVVPCQDDCSQAYQAFLCYYHQYGNLKSSEEYIPLPQLDAVQAAIDCMLILRTPKELLEQYVQGVFPDVPETQCLYRCQYLAEGLYDGVTFNLTRNYIREYAVPSPQIKDPATQACVDSALSSSSCNECARFWAGRGCLKNYGVPNHSGSYFQVAAGLVLNQRTCL

>AaegOBP31

MGVLIGLFVTAIAATASAVYYPPMAPLDVEESHFAYQLKSFRQQLDECAEYLQISPGSVENLVAYNYVTDDPSLKCLIRCAGINAGWWSVGGNSSGLQPPVIESYFAPACDDTCYVKRTQDCVSANVAPCQDDCSQAYQAFLCYYHQYGNLKSSEEYIPLPQLDAVQAAIDCMLILRIPKELLEQYAQGIVIKGPETLCLYRCQYLAEGLYDGAAFNLTRVYIREYPVPAPQIKDPATQACVDAALAAPHCNECVRFEAGHACFDAYGVPNHTTPIFQVAAGLVLAQRTCLDEDLNPRYNAGGSAPQPTPAPTPAPTAAPTPAGCVYNCGA

>AaegOBP32

MSVLICLFITAIAATASAVYYPPLTPSNVEESNFAYQLKSFRQQLDECAEYLQISAGSVENLVAYNYVTDDPSLKCLIRCAGINGGWWSVGGNNSGLQAPVIESYFAPGCDDTCYVKRTQDCISANVVPCQDDCSKAYQTFLCYYHQYGNLKSSEEYIPLPPLDAVQAAVDCMLILRIPKELLEQYAQGVFPEVPETQCLYRCQYLAEGIYDGVTFNLTRDYIREYTVPSPQIKDPATQACVDNALASSSCNECARFWAGLACFRDYGVPNRSVGSFQVAAGLVLGQRTCLDEDLNPRYNAEGPAPPAPTSAPTSASTPAPTPAPTPAGCMYNCGS

>AaegOBP33

MSVLTCLFITAIAASASAVYYPPLAPSDVEESNFAYQLKSFRQQLDECAEYLQVSPGSVENLVAYNYVTDDPNLKCLIRCAGINGGWWSVGGNNSGLQPPVIESYFAPACDDTCYAKRTQDCLSANVAPCQDDCTQAYQSFLCYYHQYGNLKSSEEYIPLPQLDAVQAAIDCMLILRTPKELLEQYVQGVFPDVPETQCLFRCQYLAEGLYDGVTFNLTRDYIREYAVPSPQIKDPATQACVDSALSSSSCNECARFWAGVDCLKNYGVPNLSTSYFHVAAGLVLNQRTCLDEDLNPPPAPAPTPGCMYNCGS

>AaegOBP34

MIKIRIVTLLVAVLLLETLRPSDAAMTMKQIKESMETMRKACAPKFDVPETTLNDLKAGNFRPDASKDEKCYAKCIAQMAGTLTKKGEISFSKTTAQIEALLPTELKAPAKEALKACKEVHTDYKDSCDKVYYSVKCAADFNRDVFIFP

>AaegOBP35

MKFFVAIAVVALAAGAWALTIDQQKKAEAYAAECVKSTGVAPDTPVKLKKGEFAGADDKTKCFSKCVLEKAGFMNEKGEIQEKTVIDKLSVDHDKAKVEATLKKCNQKGANACDTAFKMTECFYNTKAGLV

>AaegOBP36

MVRPCLYYCCILIAIFCWVQSWLVGAAPQKAGEFSRSMGIEMTASQHGECVTETGVSEESIARFNGPEIFEDDDKLKCYMDCMFRKFGATKPDGEVDMIEVYHKIPKDFNSVALIVNNKCRDAIQGANQCERAFSHHKCWKQMAPEVNHVHS

>AaegOBP37

MYRKTLLAFFFFLFLSCGDAVQNLTALRGSDYPPMYLINLVKSALERCHQLIDIEDSVIVRFRDDGDYEGTEQLGCYLHCVFREKGYWIPEKSEVDIMKILDIVPKDFEQPALKMGLRCLKVKGDDDCSRSLWYHSCWKKNDPAKVES

>AaegOBP38

MVTTLLLLFLVGVINCQEPRRDANYPPPELLEKMKPMHDACVAETGASEDAIKRFSDQEIHEDDNLKCYMNCLFHKAGVVNDNGEFHYVKIQDFLPESMHLITLNWFKRCLYPQGDNLCEKAFWLNKCWKERDPVHYFLP

>AaegOBP39

MYVVNLVLVLLSLEILSTSDAAMTMKQLKNSLEMMRKACAPKFNVVEASLDELKAGRFANEADKELKCYTMCIAQMAGTLTKKGELSLSKTTAQIEAMLPQEIKAAAKEALNACKDIQSGFKDPCEKVYFSAKCAAEYNPDVFFFP

>AaegOBP40

MTRLVSSIICVWASLLLSISAQYLQNEALLQAQATCVEYLGIPEARLEQYNISVYPPDRDTMCMIRCAGIVLGFWEDEQGLLIDGAKQLFPDSGDVDLVAQKVLHCAERKLLSCDPADACARAYYSFRCAMRKFEPSNSALSTDQKLTPEKFLKAQIVCANILRIPHDHLKLYNQGVYPDDAETRCLLRCIGVRLELYSDAMGPNLDRLHSEFAIDQPLEEFKTRATLCCEANRPLIQDYCTAAYRNLYLCFREHFNAFTSQNRQTLLSHTSSPQTCIDLESDILLYGDDV

>AaegOBP41

MKHLVSFVLLALAIYPVHSARRYRIAAEECVQYLKICPTRLEQYLKFIFPEDRETMCFMRCVATKLNLWCDRKGLNWAVLEDRICPSVREKVEACVCRKLDLIDPYDHCPRAYYAFRCLRNYLQEIFLFKNLDRDFDDGYIVKVPSSKELIVPSCASCSNSFNPLTITEMTQKLLQCAKKCQLCSLNLCDRTTDPVVETPEFQCTVYCASICTGVYSEQKGILMDNLYAQLARCETRESFDYRLGLCFGRNALPEGSSPQAVVFQQYFKCLRGDYERFYSSNLEELLQIPGISKYCF

>AaegOBP42

MECCNTPMLLDKDIMMDCYQKYGDQTKKQMKLEGVPRGCCIAECGLNATGLYSNGMIKRDDMTKMFMDSVKDMPEWQMLVRDTLDECFKMAESKMDEIQAGAMLEPSFEGEKICHPISGTILRCMGMNLFVKCPAGVYNESDECNQLKEYSKMCPIM

>AaegOBP43

MKIVIATCFLVGLLLRFLVAGDDDVCKNGSPTNKSLWDCCNMPNLVNQDIRADCHQKYGEQTMKQMKLEGTPRGCCIAECQLNATGLYADGMIKRDEMTTMFMDSVKDTPEWQPMVRDLLDECFRQAEANKDIIAAGAMLEPSFEGEKICHPISGAIMRCMNKNLFLMCPKESFTEGPECTQLMDYFKMCNGI

>AaegOBP44

MESKTFHFLLPLLCTLASYTEALDHAAILKSPNELQLECSKYLPSIDVSRNVDCTDRCIGLVGRFWNDSIGRPAQTIARYYQPDTGSQDYITRTDQCLCEKVLTVPRNAYCQRASSGLQCYRDNYGQLLTGTPQFVPVTEIRAAQIFWDCAQMLQISRDRLTQIFKDGYNKTSEGRCLIRCFLVRAGLYSDCQGPNIGRFAVQCEGYSAEYEQAAVMTG

>AaegOBP45

MELKTCCIILPLLCTLTSYAEALDHAAILKSPNELQLECSKYLPSIDVSQNVDCYDRCIGLVGRFWNDSIGRPAQTIARYYQPDTGSQDHITRTDQCLCEKVLTVPRNAYCQRASSGLQCYLDNYGQLLTGTPQFVPVTEIRAAQIFWDCAQMLQISRDRLTQIFKDGYNKTSEGRCLIRCFLVRAGLYSDRQGPNIGRFAVQCEGYTAEYEQAVVSCYDRLKKESLDSCSLATRTMDECIQGNQFSSSDIDGLEKLEVQ

>AaegOBP46

MSGSISLIVLAVVALAGQVLGRHDATFKSFGSTSGECSRYLNNDGNGECNIHCVGVIGHAWNETLAKFTQNYAGYFVPDPQDDCYQNRTERCLLQVDNAIPVYDKCTRASKLGQCYADQYGQLNAIQPQYVPMTDLQYTRVFLQCAAILGLSNNDLNAMVQQGAYNTPAGACLLRCTLIRMGLYTDDAGIDVALATRQCGLYNATSDIAQCQAKVQAEECDKCKRTTRIAKECLNMHYNVRNVGDSYGLELYGVDTCYSSCSFFYCYYYACPYLSYYNTNYAGSSSYSGSSNSLTFAG

>AaegOBP47

MHLSALFFTVVSFLGSFCFAINPCIEGPPVNKSPSECCTTPALIDPPLMMKCFQKWGEQTKRQSKMDGIPRGCCVADCAMEGTKLISKGKFNREKARKVFMAVVKDQPQWQPIVNETLDACFKQADENMAEIEAGAKLKPSYKGEKICHPISGSILRCMNMKLFSKCPNDLFNSGPECDQLKLYHEKCPLN

>AaegOBP48

MKATVTSVLVLLAISHATLADPAAPDNVPASCLNKNFNVDPFECCKTPKLLDEGTVKECVHSFPPPQNAQDEIKPDCMSECVMNSTRIFDRRQNVNDAKAMETFLEKLNGKSVWAEIVQKAVKQCLDDADNRKEEFSRDMKALQQKFPKERICSPAAGFIMECVHVSVYKNCPASIFKDNLAGCPAIKKHLNVDNCPFYTIFPEKKAPKPVKRH

>AaegOBP49

MIYSQPPPDDKACFQGYKVDANGCCELPRFVAREINAKCDEEFKPLSPRLPPEVQAYEGSCVIECLFNVTGMFKDGKLQQDKIAQQLKKTIGADRNFAPLLGGVVTDCYRLVMDNPANSFKPIPVKPGRPGCSFIPQAYMNCVKSELFENCPKANWTAADGCDLLKQKLNVGCSYYSIMIGKKGLKS

>AaegOBP50

LLIRFNTNLKTQVVAQLPQEDISCYMGNVKIARECCLMPRFIDKTVDDACTAEHKNPGPRVPPWTAKTEGSCVVECVLTRIKSFSNNIIDKEATKLSFGKSIGIKTFFGAVTNRSVDLCHKRILNNTALQLANPVSHDSNRTACSFVPTVFLDCFKENVFMNCPKQKWINVPECNALRSKISSGCTFNAIKGYSNSTHF

>AaegOBP51

MLVHLLPTLIVTLLGIGTVVAQPRPDDPSCMEGNQRKAHDCCRMPMLVEQSVMNRCMTENPMTPPVPGVQRTEGCCIAHCVLTTLNAFRDNLIDAAAAKRALTQSMGANSSFVSLVSGVVDECVNLVHGNAAYKVAPVASTPGRPGCSFMPEGFVNCIKGRFFQQCPTAEWTRDAACDQLKQKLTAGCSFGSLMG

>AaegOBP52

MFKLGFLLILVSCLAISVQCVGFDPSCFQSSSSKKADDCCLLPKFYDSQMVSDCLTSISKSTNDVEKYQCLVECIAKKLNLFKGNTLDREATMQLYKARIGSVPHFAPIMDNIFQQCYDGMAVYAAQDRSDPTKCSALPMMLLNCIQTRLFQNCPAKLWQGGPECQELKEKLLEGCPYAAIASF

>AaegOBP53

MLNLWLMVLSFAVTTHNSTWDKSCFELKTSKRADDCCDIPGSFDEALLKRCYDEQKASKNEQEAIKCIAECVARELGAYKNHTLIRENSRLVFESTIGSDPNFRPVLGDVFEKCFNRITAIEAQETYKNATCHFAPAFMLNCVESGLFENCPVSIWNEGVGCDELKEKLEQGCPFFAISETL

>AaegOBP54

MFIDAYSILKLSCNDGPVDKSCFELRTTKRADDCCKIPDILVESDESMVRRCFAQQNKTLDEHETAKCAAECIARELGTFKNGALDKELAKKVLLGRLDKDKNFKPIVGGVLDKCLGRINAVIEKESKRNGTCNATANFLFDCAEQGLFENCPSSVWDSNDGCVELKNKLAQGCPYSAIAE

>AaegOBP55

MTKRMELVLFGLFAVVTLFQTGLGGVGVEGKATVEQMTKTGEMIRNVCIGKLKVAEDLVNMLGDKQFPDNKELKCYVNCIFEMMQVVKKGKLNYDAAMKQIDTIMPDELAEPMRIALNACRTASDGIKNNCDASYAIAQCVAKNNPKFVFP

>AaegOBP56

MEKTGKLFRQVCQPKHKLSDDILEAGKNGVFPDTKNFKCYISCLLDMMQVTKRGKISYEKSLKQIDQLLPDDMKPDFRKGLEACKDVASGIKDQCDSAFVLLNCFYENNPQFILP

>AaegOBP57

KFSSALSFCSLQLAWRFVTELQCANSDEEKKAQAKEMMRGMAEECKKKEGATDEDVEALLEDKTPETEVQKCFLSCFQHQFQISDGKRFNKDGFMQLSAMMFGEDQEKMATAEEIAEECSSVENADRCQLSVDIKECVEKAMDKRGIKMEK

>AaegOBP58

SSPFYHALQVCARDLSVPPDRFEQYRLLIFPDEPDTHCFVRCLLLGIRAWHDQTGVRHSALQQYFSPDDNPVDAYARVQTCLDYVSNSCAATESCTKAYWSLNCYKQQFGSYFFSREQFVPATDIQLAQAMFDCADKLDISRSIVAAYRNGNRSELISSRNPCYVRCVAISIDLYDDDAGLQWDHLYVQLGLNEHRENYLEQVHKVVSELQLSTMDRCAAAAQVIEPFLLTALQQSRTAYRTGIVVETTASITTTEVSVTKPMTTKTPSTTIQMTKASTSAPPTT

>AaegOBP59

MKPGVKLSLLLLIGLMALLDSTSGCSMTNNDGVEQREALLADPSTAPARSMKDYSVEDIYAECNKTFAISMDFLNELNDTGSFPDETDKTPMCFIRCFLQKGEIVTSDDKINKEQAVALGWVKSGETIDDCLQELTGNPCERAYFLMRCVSTRHLVEGRSKDSKKR

>AaegOBP60

MILLNMAVVLLEVMLTLAADKPIPRRDAEYPPPFVLEISKKPHKMCVASTGVSEAAIKRFSDEDIFEDDEKLKCYMQCLFEKLRYTDDKGELHLGKVMDSVPEEYEDIALKMGSKCLKPKGKTQCERAFWYHKCWKTSDPVVSICDYVFL

>AaegOBP61

MKTIAAIVSFALIVGCMAVTEEQKEAARQLAGKCMQQTGTSEESVQRLRNGDTSGADDNTKCFVQCFFQGAGVVDGEGNMQEAFVTEKLASEYGQAKAEEVVQRCRNNSGANACERSFSLLQCYIANRASLM

>AaegOBP62

MKIVIATCFLVGLLLRFLVAGEDDVCKNGSPTNKSLWDCCNMPNLVNQDIRADCHQKYGEQTMKQMKLEGTPRGCCIAECQLNATGLYADGMIKRDEMTTMFMDSVKDTPEWQPMVRDLLDECFREAEANKDIIAAGAMLEPSFEGEKICHPISGAIMRCMNKNLFLMCPKESFTEGPECTQLMDYFKMCNGI

>AaegOBP63

MECCNTPMLLDKDIMMDCYQKYGDQTKKQMKLEGVPRGCCIAECGLNATGLYSNGMIKRDDMTKMFMDSVKDMPEWQMLVRDTLDECFKMAESKMDEIQAGAMLEPSFEGEKICHPISGTILRCMGMNLFVKCPAGVYNESDECNQLKEYSKMCPIM

>AaegOBP65

MDTFNAIRNGDFSIRTPFIECFGDCLVKKAGFMNDDLSFNKDVIVKFASRFIKPEDAETVYSQCTADVAPVLCATAYDVYQCIYENALAKWGTRRNGK

>AaegOBP67

MNESLCRNNTVLARNCCRLPGIINQSIVDDCDDKFPHHAPVKRVEGSCVVDCMYKTIGAFQNGTLDLDITLQHISQTVGRYPNFEPLVNETVSWCYRNVTENPALQKSVGCSFIPQEMNDCVKKMLFMSCPPSNWTTKVECDDLKGKIAEGCSYSSLY

>AaegOBP68

MDIFIIGLMLASSVLGQPPAENKTCYQGNQKTAAECCPLPRMMEKSIADMCNSKYKALSPRVPPGVQKTEGSCVTQCIFTTIGGYNEKNNTLNIEAIRKAILTTTANAKAFLPLFNSSIDHCYPIISKDPQFLANPVSPIPEREGCSFLPPALMNCIKIDLFQGDVVSLDLSKAFDRAWRFPILKSFED

>AaegOBP69

MKMSYSHELLFVAMLSAVLHLSSAMDCKEVWERKHETADCCSAPAILNLDNLKSNIEGQEGNKHEKFFCGVHNLMKEQNLVDDEGNLDVDAMKQNTEGFDDEWKQIAQQAIDHCVQKTESMMADMEQRGGPKGQCQPTAGMFLMCLGKASIKNCPADKWNSSELCEKVKSGECDKRGHKH

>AaegOBP70

MIRVLLFLTFFVGATLSYDFKDPYFNDFLLEDLMVLQGRPALKKASDSEESQLQYTCCDYLNEENFSKLQQTQIVCYVENSLLSATKTKSGRAVSPVDMFSCDRLDKLKQQYICASDCVARKENITDDSGNLLGSEVLVPFVSQYYAPEVFQDEQIKEFVDTCLGESKTDETVANKCNPSSARFGYCMWRKTILSCPNERQDTSPACDNLRDKLLYQEAKYLSDETR

>AaegOBP71

MVRVLLFLTFVVGATLSYEFKDPYFNDLLLEDLMVLQGRPALTKASDSEESQLQYTCCDYLNEENFSKLQQTQIACYVENALLSATQMKSGRAVSPVDMFSCDRLDKLKQQYICASDCVARKENITDDSGNLLGSEVLIPFVSQYYAPEVFQDEQIKEFVQTCLDESKTDESVANKCNPSSARFGYCMWRKTILSCPTERQDTSPACDNLRDKLLYQEAKYLSDETR

>AaegOBP72

MNIIVFLAFLVLAVDTDKSPVDAECIDVEKNADEIRQCCDIPSPLEMENIQTCKEKYQEELGSDVPNLVACIFDCHARELGVLKDDLEIDEAKMMEYVSQTPDEDVKKLMVESAKECLKAKGEIMEKAKEHAMKCHPLAFMMTECIMHAVYSECDKLPNHWKDSEICSKVKNGAEPCE

>AaegOBP73

MQVLNFLCLVLLCLVLEKVAVAEECIKFEDHKDEILNCCKYQPPYPKDDVKECVQEAQGKSGGDKHEFFACLLECYLPKIGIINGDSIDEDKISEHLQSLDENARDILLAAYKECDESTTGTTRAQCSSYALDLETCVLQKLDQQCPDEFYNPSEICDKLKSGVEICH

>AaegOBP74

MKLNLALAALIGMVAMVHGQQQINQECFNRPNDKNPMECCRAPNIMPPREELITCMQKFPKPSGPPTPGSPPPGHNCMAECMLEQQGIMSGGALSKDTATSKLVALVGSSSEWQAVARKSIDTCYSQVSSLGGQKDSLGCSVIAGSFMECMPSMMFTNCPSSAWTASAECDQLKAHLQKGCPLMTLFKGPHPH

>AaegOBP75

MKLNFALLTVIGLFAMACSQQPISQECFTRPNEGNPKDCCKAPNVIPPKDQFAECMQKYPKPSEPPTPGSMPPNHNCLAQCMFEQQGIMADGAVSKDAAISKTVAVMGGSSEWEATTKNVVEACFQKVSALGAQKDSQGCSVMAGSFMDCMPSMMFTNCPSSAWTASTECEQMKAHLQKGCPIFTLWKGPPPH

>AaegOBP76

MEIFHVGPMHQNGCPGPISQCASQNSNVAKQIDDYRKQCVELSDVSVDSAIKVHSGQVIENPDWSTKRYVQCFFQKMQFMDENGVMLKDAVVEFFSRIQDESRAKAMVENCDIQKENPLDTAYAVLVCYQGNKN

>AaegOBP77

MKHSGAIACCLLIAIVAVNAWPSYKRAEVRAHVRNCVKKTGIPGKNALKVLKGNFNDDSSEVKKFMKCMFQEVGFINEKDELLDNLLIAKIKENLEEDEADELIEKCSIVGDDINDTAFQIYKCYYENHDLPPDMLVR

>AaegOBP78

MKILEVVVFLTVVALCKADYSDKQKQKLDEFTSKCIEDLDLPKDSDLGKKFKYGQLKEKDDATKKFISCSMQKLSFMNETGSILEESIIEFLADKYDRTMAMNVITKCSKLKNESMEDKAAEFYDCFFMQKSFDI

>AaegOBP79

MKLSINLFVILMLTTLFVSTHQLGFKPFSAEKLRDIELICMKLLRQPIAFWYKYLNLEYPDDPITHCHLRCIGISTGLYGDEFGAHLDNIYEQFKENTLLNRTAWMEEKNNCLAKQFADGLPDDLCKRTFLTFKCFEVDYLLALSKSDCSKISI

>AaegOBP80

MKWSLKLLVLLTTLFIPSQQIIFAPHSAEEIRILEQGCVKLLLQPSVFWYKYLNLEYPDDPITHCHIRCLLIAAEFYDDELGAKLDNLFEQHQHDTPLDRTEWTEAKSICLARQFANGVPEDLCKRAYMTFKCFEIEFLISITRMDCNKISL

>AaegOBP81

MKAVRFLILLVIIGVFHTIPADAGQLLNKLITVCTQGQNPPADLVQRYRNGEFPNDRNTHCMMRCIALNLGVYDDLNGIHMHDTWQMFRRGRPASHEKAFAEQHRQCITQQTKDVPLDDYCGRVYAVYQCYKDEYEALLRNVRQGAAKARN

>AaegOBP82

MPPLLDENLLHGCKQLHGGEHLTRGLIHERGSCFIECAMNSTGTLVNGVLDQPKIVQLITTRTAGVSSDLTQVMVASCVKCFLTPLVMGNHSGHPLDSKHCRPAASIFVSCVNMEMFKMCLPEFWTNSDSCNNLRLHITNCPIPA

>AaegOBP83

MGNHWPSSHSILITVSIVFLFLLLEETWALKCRTEDGPSSDEIRKVIRVCMKRITSESENKSNNEYENYDSSYSDSNSDEDRESSTEGNTRRQTNGGNQSTNTRGRNGEDMSRGRDSSRSSDDRSRGDNRNRQDNGRRRDRERDYDYGPMGRRMDDGRNQQGRYKRQYYNDGAQGGYGYNYQQNDRYNRDRNQFMHPNGNTSSNGTNNTERDRACMMQCFFQEMKMTNNEGFPDKHKVLHVVTKDLRDYELRDFYTDSIQECFHMISMDNKLKDKCDYSMKFVTCLADRGQANCNDWENEAIMF

>AaegOBP84

MRRFKLASFILTLFATNVICSRHKIVQKSLAGTGVECQQYDPPWNCAVRCQTLLTRDWVDSTGMQSPYDRFFQPDPNDQCYMNRTQRCLLDKLSTVPRNKLCLRADSSVQCFLNQSGQVIMDQPKFVAPSRLLENQIFLECGTMLGFSRQRVWEVLYKGEFTLPEISCLVRCFLIRSGLYDDKSGLNLERFYVACGGYDDAFYHNVTKCIANVEAAGLCDKCTRAQRLALECVGSQYPIFVPVSQTDIDSTNNAGRDVNNYYTSNFNFNFGDVISQIGTMVPATGGG

>AaegOBP85

MSCLNLATLILAFFATSVICTRHKIVQKSLAGTDIECRQYDPPWNCAVRCQTLLMRDWVDSTGMQSPYDRFFQPDSNDRCNTNRTQRCLLDKLSTLPRNKLCLRADISVQCFLNQSGQVIMDQPKFVAPSRLLENQIFLECGTMLGFSRQRVWDVLYKGDFTHPEISCLVRCFLIRSGLYDDKSGLHLERFYVACGGYDDAFYHNVTKCIANVEAAGLCDKCTRAQRLALECVGSQYPIFVPIPLTGKLKLHG

>AaegOBP86

MTIFNALLAILACFSLPTDALQHNAVYKSINSAGPECRTILTRQSPLDCRLRCLSINTGDWDDCSGVPRTYDRFYVQDPTDVGYQQRTQQCIANVSVSILRGDICAFSARSTECYDANYYDIVLDQLVFVPSKSLQYQQTIRDCAGMLGFTEHVISDVLRDDCFALQETRCLLRCLLVREGLYGDQCGAQIDRLYVVTGGFDQLFRRDVKKCTGRLRAMGLDKCTEAYRVASECFPEDKAILPIFLKNKAILQEI

>AaegOBP87

MMSSGAFILSVVLSVSISVLQTSSLQHSATLKSFNEILSECSRYLPSNDEPCYDRCLGLVGRFWNDTIARPSVSVGRFYRPDPCDQNYVNRTQQCICDSVLPLPRKDVCLRASRGLQCYRNQYGRLIADEPLFVSVTPLQSSQIFQDCAQMLQIPRAKLEEIVQQGYSKSPEGSCLVRCYLVRAGLYSDSQGPDIARFAVQCEGYEDAYEASVARCYQKLKSEQLDKCTLAARTYDECIQANEYSNSNLEILGVLLGIITGLIPA

>AaegOBP88

MQVFAAIVKLVAITLGAIIASISCIEEHSASLKSILSSTAECNLYLPTEALRQECGTRCVSLVNRIWNDTNGRLSDTIGRFYVEGPQDPCARNRTLQCLEQVTASIPIRNSCKLADASVNCYRNNYGQLDVKSPRFVAFSDVQQVRILTECAAMLGVSDKLVQVVRNGLQSIPEGACLLRCLLIRQGLYSDQRGPDLKRVSVQCGGYEGYEQEWRANVTRCVAAVHAERICDKCLQAERIAVDCLQMHLHLYEVRSPKLRQHIPFGVEFYTRANAAAGSAAAAQARVITYITVEYYYWY

>AaegOBP89

MINQLLITLTTVNILTTSAVEDWRSPQLKSFSSAQQDCAVYLLLSNETVQQYVKSGYPDEFSSRKLINCILVQIHAFDELTGIKDHVLTNFFDQPGSCSEYVGRTQECLRTSVPKHCEGQPFEHAYRSFQCYYRNYGSLLMDTVRFIPYEQVDRIKHLTESFSIVNTSCKALRELSVGQGFIVENIADPMYTLAVRSGFYDREHGLYLDRLYTQFGKPALLSDATRQCLVRVSQQYQTEPLRLTQLVLQCVESEISTQSLFTETARQVLASNSSYCNVCEQLPSCVTMTPGVTSAAVTTTRAPLSTSTRPPYPSI

>AaegOBP90

MNAWDDETGIKDYVIRNYFKPADTDPSYKSRTQCCLRDKVANLDRCALFERAYHSFMCYYQNYGNIVPEAQFIPWYQVDREKHLREVFLIEGITRVQLEEFQRSDALKAKEYPILYYIDFVRTAFYDPSTGHNLERLYTQFGNPGLLADETRRCLDAVSLQYCDEPVRAYQGFDQCLRNYMTTEELFKTVVAQVLASNIVCR

>AaegOBP91

METRSIFIAVILSITSYVKAEDYEAPRLKTLATIEQECAGYLLLSNETLRSYIAASFPKDSTVQKLVHCFLVNMNAWDDETGIKDYVIRNYFKPSDTDSSYESRTQCCLRDKVSNLDRCAVFERAYHSFICYYQNYGNLVPEAQFIPWYQVDREKNLREVFLIEGITRAQLSKFQKSEERNPKEYPILYYMDVIRNAFYDPSTGHDLGRLYTQYGIQELLADETRQCLDTVSRNFFEEPTRAYQGYDQCLRKYLTCMWKNCCRLLLRRFWNQI

>AaegOBP92

MRCCLILLVSLVALHSSLALNHLEKLRWKTFREAELESAEYLFITHETLERYRSSGYPDEPSVRKLIGAIMVVLNAADEKLNLIKDYVLSQYFLPNTVDCQYKQHTKECLDRNVATLDPSDRLGRAYQTFQCYYKNFGGIKEDVGWVPYHYSEVVQMLEDCLYITNASNESLLQYCQGGYATSADYSNVAYCYAVRAGLLDKTTGFNVEKMYIQLGDDNLNDGDAKKCIAGVVNQYCKEPYRTMRIVVDCVLIYLPGVAGIVIAASNILGNPPECVIPPSPPPITQPCYNGRCL

>AaegOBP93

MSYQLLTPLVTFIMASSLAAEDWKSPELKSFSSAQQECAVYLLLSNQTVQRYVKNGYPDEFSCRNDSVSRTQECLRTTVPKHCEGQPFERAYRSFQCYYRNYGNLLKDTVRFIPYEQVDRVQHLKESFSIANTSCAALKDFCEGHGFNVAELAEALYVLGVRTGFYDPQHGPYIDRLYTQFGSPNLLSEATRQCVNRVSQQYSTEPVLITQLFLQCVEDDISTEALFTETAKEILASNQSFCNVCETLTSSVSSSTTEMVTTTTATISTSGAPLLTTTKGPYPYRSM

>AaegOBP94

MNHQLFITLAILSIITSLAAQDWNSPQLKSFSSAQQECAVYLLLSNETVQQCEVNGYPDDFNCRKLVNCILVQIYAYDERIGIRDNVITNFFEPPKSCSDYVSRTQECLQTTVPKQCGGQPFERAYLSFQCYYRNYGVLLKDAVRFLPYDPSSPKYSVKQIVESFSIANTSCKAIRNLSEGRGFTVENVADALYAFGIRNGFYDLQHGLYVDRLYTQVGVPNLLSEATRQCLACVSQQYNTEPLRITQLVLQCVEKDIATQLWFTQTAQAILASNNSYCNVCEPLRSCVDPTPSTQCGVSLVTTPKNPYPSI

>AaegOBP95

MRCCLIFMLPLVALQSSLALEHLETSRWKSFREAELESAEYLFITNETLERYRSNGYPDEPSVRKLIGAIMVVLNAAFEKLNLIKDYVISKYFIPNTVDCLYKQHTKECLDRNVATLDPSDRLGRAYQTFQCYYKNYGGIKVDVDWVPYHYSEVVQIVEDCLYITNASNESLHQYCRGEYATNAGYQNVVYCYFVRNGFYDKSTGFNVQRIYNQLGANNLIDDGTEKCITQVVNHHCKEPFRSMRVFLDCVVRYVPSSAAITEAASNILGNPPECVVPPSPPPKTQPCYNELCP

>AaegOBP96

MFRIGLLFVSFAVVSITAVDRHKIVYKSLQEAAVECGQYTIKGQCLGRCETLITGDWNDTTGMSPAYSRFFHPDPVDECNLNRTQRCLQTKVYTVPRPRTCQRASESIQCYLDQFGQVNLTAPQFVRFTPLQDDQIVLECAAIMGYTYEQVYAWIRESAFQRPETRCIYRCFLIRSGLYSDSEGLNMARFYVLCGGYEEDFYQRVEQCAARLRQEVPCNDKCTLAQRLAIECIGADYQAGNLATNANSKAVEGSRVQNINANPVNSVIDATNSETGNVITITRTNSDTYVYGDENTFENYFYESA

>AaegOBP97

MFRIGLFFASFAVVSITAVDRHKIVYKSLQEAAVECGQYTIKGQCLGRCETLITRDWNDTTGMSPAYSRFFHPDPVDECNLNRTQRCLQTKVYTVPRPRTCQRASESIQCYLDQFGQVNLTAPQFVRFTPLQDDQIVLECAAIMGYTYEQVYAWIRESAFQRPETRCIYRCFLIRSGLYSDSEGLNMARFYVLCGGYEEDFYQRVEQCAARLRQEVPCNDKCTLAQRLAIECIGADYQAGNLATNVNSKAVEGSRVQSFD

>AaegOBP99

MMLTGFSSCTPQNGRNGITRGCQRDSLRWSWKKKTKFMWVSSTLLVLSVLCVSAVELPPPHYVTRISFYTALQECAEYFQISENLLQQYISSSYPDDPSVHKLVRCSLMLLGCWDDITGMRRNVIENFFEIDPNDRDHVRRTNECIRKSTTEDVSSPAYVAFLCYHQQFGNFKLHSKRFVPFGSHELKQLVEMALNVAELPWFVPAQYATNDILYEPHFPPVLYFIFVRGGFYNAKIGFDLRNLFTQFGVEELLKADVEQCLANVVHTEKVNGHESIVIKGFQKCLAHFIPLLEVVQDVARSTSNDRSASVKACTGLNPSTQPPFYNRACED

>AaegOBP100

MGLKKWCIILPYLCSFAICVSALEHVATLKSFDEIRYECSQYLPSSEDEDCSLRCLGLVGRFWNDTIGTPSNSVGRFYRPDSCDQCYLNRTEQCLRRTVLNLPRSAVCQRASNGLLCYKDQYGQLINRAPQFVPVVKLRAMRIFRECAQMLEIPFDKVDRIFKEGRNNTSEGRCLTRCFLIRAGLYSDSRGPDIGRFAVQCEGYSVEYERTLVQCYEGLKAQQLDSCTLATRVLDECIQNNKYSYSNMDDVVTIQITDFTKLQVMVDLGTLVVFFPSL

>AaegOBP101

MEETDSNLITWHRVYLHREYVHGIGSETRNSLFHLHNARDLVLVTTCRHGKSWRDLQDARCDEDNREYDRTVALKAPTMQLRSKCLVLPLLGLCIFANRAVTLQHTATLKSFDELRIECSRYLPPVDALNNVEDCSDRCLGLVGRFWNDSISRTVYSVSRFYQPDSCDQDNLDRTEQCLCETVQSLPRNASCQRASCSMQCYQDQFGELINQKPQFVPVSKLRSAQIMSDCAQVLQISQDTVRQILHDGYNNTCEGRCLVRCYLIRAGLYSDRRGPNIARFSVQCEGYADEYERSVTDCYAGLKAQQLDKCTLAARFYDECILSNEYSNSNMDVIAALGGSLYGVILTTVGVTGYLVTSIVAGLSAAGIP

>AaegOBP102

MGLKKWCIILPYLCSFAICVSALEHVATLKSFDEIRYECSQYLPSSEDEDCSLRCLGLVGRFWNDTIGTPSNSVGRFYRPDSCDQCYLNRTEQCLRRTVLNLPGSAVCQRASNGLLCYKDQYGQLINRAPQFVPVVKLRAMRIFRECAQMLEIPFDKVDRIFKEGRNNTSEGRCLTRCFLIRAGLYSDSRGPDIGRFAVQCEGYSVEYERTLVQCYEGLKAQQLDSCTLATRVLDECIQNNEYSFSDLFDLLEPYLDGVISIGDFRRLELMIQLDNLVIYYPSLPSSSLGI

>AaegOBP103

MQVFAAIVKLVAITLGAIIASISCIEEHSASLKSILSSSAECNLYLPTEALRQECGTRCVSLVNRIWNDTNGRLSDTIGRFYVEGPQDPCARNRTLQCLEQVTASIPLRNSCQLADASVNCYRNNYGQLDVKSPRFVAFSDVQQVRILTECAAMLGVWDKLTQVVRNGLQSISEGACLLRCLLIRQGLYSDQRGPDLKRVSVQCGGYEGYEQEWRANVTRCVAAVRAERICDKCLQAERIAVDCLQMHLHLYEVRSPKLRQHIPFGVEFYTGANAAAGSAAAAQAQVTTYITVYYYYWY

>AaegOBP104

MQLFLTLLIFTLCTSAYAFLDHYVGHKRFDTIFRECGVYFQVPNCILDEYVANAFPDEPEVRNLIHCTLVGSKSWHDGSGVVEHVISNFFNPGPEDTCYADRTRDCIRNSRVPGGNNVTLAYKAFHCYYRQYGNLNHSEQFMPCSPQELQVLIKTSIAIVNVSQAELVNYSNGAVLDQPNFAELIYVIILRGGFYFTGQGLFLANLHTQFGNPELLTPETQQCVDAATAAWNGQRQKDLVHAYFVNCLRRITPWMQLIQDVATGLVRGSNAPCSTSSTTTSTTPSAVQPCYNVGN

>AaegOBP105

MWISAACLILALSFTSTSHVLGLDHYFSYKEFDSYFHECGEYFEVPNCTLDEYTANAFPDDPEVRRLIHCTMVIFKGWQDGLGVVESVMSDFFNPAPEDTCYADRTRDCIQNSQAPCDSNSTLAYKAFQCYFRQYGNLNQSRQFMPYTLREEQVLIETAIAIVNVPKDELVNYSNGILLDQPHFADVIYVVFVRGGFYDVVQGLSLDNLYTQCGKPELLTAETQQCVDAATSAWDGKSRKDLVYAYFVNCLQNVITFAQRIQEVATYLVAVPPSPCPPAPSTPCPTTTTTTTTTPPPPSTVPPCYNVRN

>AaegOBP106

MISIVSSEDAQPHLFLITQSIRSFPSALAECAQYYELSNCSLNRIVQESYPNEPDVRRLIRCALINVRSWNDTTGVQEQVMNSYFNPTPEDTCYLNRTRDCIERSRQLPGGDRDVQTRAYDAFICYYRQYGNLNETEQFLPFTDEESDQLMISVLSITEVSQEALVQFSEGNILDNKEFPAVLYTLYVRVGFYQDRIVPQHLYIQFGNPELLSPQTEQCIEAAVNSLPCEADDKDQVYRIFRNCLVGITRTLELTQSVSRQLLGLEPFCGNGDSGSSTNAPCAITASPASTSQAPYYNTVPR

>AaegOBP107

SIELSNRPSTIMNRIVLLVLISLCSASTVLADGLPHYIAENSFDISLRVCAEYFLVSNETIDGYYQQGFPEIEEVKQLLRCAMINLGAYDDTFGPLEYVLGNVFKPCPSDTEYAERTRSCVKKALDSICPSDVFSRAYASFMCYYRGYGNLITDEFFIPNSLLELTQMMLFVQSSLNLPDEVLVQYSQGNILNEPNFPNVLYVWAVRGGYFSVDEGIQLENLYIQYGIPGLLSQETRQCAADVAQANCNLDLVTLLYNMYVTCLRPLLPFESFVQTFAVEQLKCKTCGAVQPAKPSYTY

>AaegOBP108

MGLATTSIALLVLSLAAVGRCDLPQYSVYKSLFTALYECGEYLQVDNVTLDQYIYYGYPSIPEVKRLIHCAMVNVGAWNDNIGVRPNVFRYFFKPNELDTEYEERTQQCLAQICPNEYDQNYRAFETFSCYYRQYGRLVKEDVFNPLETLEFLQLLQFIKLVLNIPNEKVVQFAAGDYLNDPLFKQALYIGVVRIGALSRDKGFLPDVGYAQYGYPQLISPCVQKCIADVAAQYMNADKRELVYQVYVQCWYSFLDPFLRSQFQAALDGSLCDVQVKY

>AaegOBP109

MGLATTSIALLVLSLAAVGRCDLPQYSVYKSLFTALYECGEYLQVDNVTLDQYIYYGYPSIPEVKRLIHCAMVNVGAWNDNIGVRPNVFRYFFKPNELDTEYEERTQQCLAQICPNEYDQNYRAFETFSCYYRQYGRLVKEDVFNPLETLEFLQLLQFIKLVLNIPNEKVVQFAAGDYLNDPLFKQALYIGVVRIGALSRDKGFLPDVGYAQYGYPQLISPCVQKCIADVAAQYMNADKRELVYQVYVQCWYSFLDPFLRSQFQAALDGSLCDVQVKY

>AaegOBP110

MLSITFALVLFLSASSAVIVSPLDHAIETCGRDLQVCESKLASYRALSFPDDRETQCFIKCVLIELQAWSNPRRLLKHSTIQQYFIPDAADYSFEDRTRRCLDQTLPNCIPGDSCSRAYWTFLCYKDNYGNLIRQPNQFIPPTELDIAQHQLDCADILRIPREELLNAETLTNGSNCYARCILLRSEVYSDESGLDLDRLYVLLGYDTEKQAFIQYAQQFLATDSANCQTDRCLAAQVPYQLFHELLVKLFKRDGTRDISYVFDVNNV

>AaegOBP111

MSASAWIRVTFLLVVGLVAYCQAQDKYSQMYRGPVVDCREIFGYLNNLKEFFKDECGQLDEGCPWKLQKAIKWYGRVAKQIKQYVSATNRHSARFKSSRSTDSECYRYLNVLDGNCSVRCRGLVDRLWDDQSGLGLSITQFYKPDPEDKCYLNRTVRCLKGVSATESCSRVDKYVQCFNDQYGRKDTETARFIPFTTVQHTRILMECAAIQGIPVESLQRAAENGSGLPQEACLLRCFLIRQGLYSDAGGLDLERLEVQCGGYGSGWDPVAVRQCIAKVEDCDKCSKVQRIAKECLQAHFKVLPNPNSDTVESVPFLVEFYVGISVLGINFKGMISGCLLSIFCFFA

>AaegOBP112

MVQFVILLITHLAQLVTAADRHKIVYKSLQEANNECSLYNVPGGCLPRCVTQITRDWNDTVGMSPVYGRFFQPDPNDLCSNNRTERCLESKSSLISSKKTCLQASESVQCFMDHYGEINMTAPQFVRFTKLQDVQLIFECAAMLGYSSMEQLDALLRDSEFKRQETRCVFRCVMIRSGLYSDSEGLNMPRYYVLCGGYEDGFYQQAAECSARLRKEVPCDDKCTLAQRMANECIGVDYETSIMQSKGNTVNTIYAIQGSEVYNIDGQNANSNVALTSVQRDKTINIENTNSDLNKFGDTINVDNQP

>AaegOBP113

MFTVKYLLTLTSITIVSCSTSEHGFIFKRFHRGLLECAEILNIPKTTVQKSIEDQFRCNDQTKLLIHCVMVQLHTWSDGTGLRRSALVQFFEPTAYEALFEPRTDMCLTENLAYVDKCDFVTRAYVTFDCFYKQYGNLARNVHAVILNQKQLISALNVCFAIADIPQEAIQRLTVENVLEVPEAHCLLYIFSLRAGLYNEVGGVLMDSIYSQFGNRTLTQSGKITCVQHLLETSRFADRCSMLNAVYDRCLFDAIPINELIVEAAKHALANVGR

>AaegOBP114

MNFDLTLFSIVVLLAISTANATRSPSLKTIDQAVKECGTLWNVSPDYFEDFVRTGTGNSTQLKELVRCASIWCRWCNVSAHDVVYEVLQNYFNPSPDDPCFLNRTERCMKASLKDLPYTEVLERAFVSFLCYYQQYGNLNRSVQFIPYMLPQEQQVALDTLVIHSVPLETLRNFNDGVFKEGTFEFLLRTLLVRLNLYSDRAGPDVKRLYNQDGNESYLTPETAACIAEARKNCPSDRCKLVSNTLKNCLPQVYDDAVSLIKDAARMILQRMFCVQDLELNPILVERLVAKGAEEIFTNRHDCVI

>AgamOBP1

MKLVTFVFAALLCCSMTLGDTTPRRDAEYPPPELLEALKPLHDICLGKTGVTEEAIKKFSDEEIHEDEKLKCYMNCLFHEAKVVDDNGDVHLEKLHDSLPSSMHDIAMHMGKRCLYPEGETLCDKAFWLHKCWKQSDPKHYFLV

>AgamOBP2

MLAQASPLLLLLLLLVTQCLDGANCSTITTQRPAPRRDGQYPPPETLAFLRPLGKLCLEETGVSPEAIKRFSDADPFDDNRALKCYMDCMFRVTNVTDDRGELHMGKLLEHVPTEFEDIALRMGVRCTRPKGKDVCERAFWFHKCWKTSDPVHYYLV

>AgamOBP3

MGHDSCWSSRWRVLAALVIFQCAILMVRSDEPRRDANYPPPELLEKMKPMHDACVAETGASEDAIKRFSDQEIHEDDKLKCYMNCLFHQAGVVNDKGEFHYVKIQDFLPESMHLITLNWFKRCLYPEGENGCEKAFWLNKCWKTRDPVHYFLP

>AgamOBP4

MSVSVLVSSLVVLFCVQCLIEHIDGAMTMKQLTNSMDMMRQACAPKFKVEEAELHGLRKSIFPANPDKELKCYAMCIAQMAGTMTKKGEISFSKTMAQIEAMLPPEMKTMAKEALTHCKDTQTSYKDPCDKAYFSAKCAADFTPDTFMFP

>AgamOBP5

MAASRSCWWWRWWWDFILGLVAFFFIPFPSVECAMTRKQLINSMDMMRSACAPKFKVSTEMLDNLRGGIFAEDRELKCYTMCIAQMAGTMNKKGEINVPKTLAQMDAMLPPDMRDKAKEAIHSCRDVQGRYKDSCDKTFYSTKCLAEYDRDVFLFP

>AgamOBP6

MTSNAFYSSNTVTWVVAVIGVYCLVFRPALVHAQQSLTQADMDEIAKGMRKVCMSRHKISEEMANYPSQGIFPDDQEFKCYVACLMDLTQTSKKGKLNYDAAVKQIDILPENYRQPFRLGLDSCRTAADDATDRCEVAYILLKCFFKASPKFFFP

>AgamOBP7

MCEYSNTRNKMSNLVVVLVLLTMYIVLSAPFEIPDRYKKPAKMLHEICIAESGASEEQLRTCLDGTVPTAPAAKCYIHCLFDKIDVVDEATGRILLDRLLYIIPDDVKAAVDHLTRECSHIVTPDKCETAYETVKCYFNAHDEVIKFCHLLVLE

>AgamOBP8

MPSRKRLCRLLLLLLLPVDLELISQDADANVFPAYPVLRNSTPFSIFQTHGAYVVRTFADATAYRDECVQQYAGRGSSLIDYMRQVALHTDNADSRWCIVRCILQKADLLDGEGAPHEANVHAQMQHSNAIVEDPDDIRSETSRCLREPPAPDSGGGCLRAYTFFACIQSTEYDLF

>AgamOBP9

MLKFVVALLAFTAVVSAEFVVQTREDLLAYRAECVKSLGVSDELVEKYKSWNFPEDDTTQCYIKCIFNKMQLFDDTNGPIVDNLVVQLAHGRDANEVREEIVKCAGSNTDGNVCHWAFRGFQCFQKNNLSLIKASVKKD

>AgamOBP10

MVRVLIVFVALLTFAGQPFAVRGQQELSDLPEVKGYKLHCIESSGITESSAKKLAAGESIKEPDQPTKCFVQCFFQKLRLMDEKGVVLKDKLEVFLTKLMDADKAKDYVQQCDLRRTNPCDTAYAVYDCYLGKKAKLF

>AgamOBP11

MIKPFVCILIVAAGCANAFMYKHPYNHHQAAVLAHEPVVPVEFVKHTTSPAFRPASFLEVMEVVLDCFNTLRIPLQRFPSYLSGIFPEDPETKCFLRCVAIKLGVYCDEKGADLDRHCVQFGLGECCENFSNRHLVCLQQNSLPCPDRCTAAYKQELCFQEPIAKYLDYHFHDLVGLLHQAKCSHDLKMLHP

>AgamOBP12

MAPVRYHFVLWLLILIGVSSLVPPGECLDISKVTLDAAFYPLFGCARDLVVPEDLIELYKKRIFPDDQLTCCVFRCLGMRLGIYDDVKGFDVDKQYERVKDRLSVDEDTYKRGVKNCIRNVLRGRTLNNCEKAYLILNQCQGNTITNSLNQQLNEIRCN

>AgamOBP13

MKSFQIATLTVLLVLLAGTASAKKASTIFGMPLQQDPVPATSTFIVSDFLQFLQTAVTCFNKLRIPEERFPLYLAGVFPNCPETQCFVRCLSANLNLYCDETGSDIDRHYLQYGLGQDYNCFRQKAEQCLAANTSPCNDPCEAAYKQELCFLDEFRKYVDSNMNSLIAAVAVEKAEQNPVYYNMLAHN

>AgamOBP14

MKLSSAVLYFALLATAMVCRVQAGSAEELEQAKEMLRGLAAECKTKEGATDEDVEGFVNDKMPESRTQKCLAGCMQEQFGVSNGKAFQEDGFIEIAKMLMKGDETKIELAKEIAADCKAVANDDRCELAVDIMNCLKESAEKHGIELKH

>AgamOBP15/16

MLTIVVATSICLMATASANAPKSLSPELLQQMGQFRSECLRETGTTDEQIEQFNSPQSVQASHELQCYMYCMFRLHNVTRPNGELDLIDVYHAIPKQFNSIALKVLAKCNKSTGPIADACERAYSHHRCWKETEPELRLPVAVCLMF

>AgamOBP18/24

MKIELFTLSAPTVPRPGGPHTEGGRNADNFKLYSSLFVFPSPLQGARLEAEHVRRIHQNARECVKETGILPKNAFRVLSGDFSVDTMKAKCFVKCFLDKAGFIDDDGVIQQDVIREKLTVGIEAGKVNELIKKCSVEGTDACDTAYQMYKCFFSNHKVPKELFQMRKGIGRRNMQQ

>AgamOBP19

MAAYLISVVNYSNYGMYITQEQLEKTARTFRQVCQPKHKISDEVADAVNRGVFADTKDFKCYVSCLLDIMQVARKGKVNYEKSLKQIDTMLPDHMKPAFRAGLEACKSAAQGVKDHCEAATILLQCFYKNNPKFVFP

>AgamOBP20

MLFVFFTLLSCTKKKKIFPLRKSTVEQMMKSGEMIRSVCLGKTKVAEELVNGLRESKFADVKELKCYVNCVMEMMQTMKKGKLNYDASVKQIDTIMPDELAGPMRAALDICRTVADGIKNNCDAAYVLLQCLSKNNPKFIFP

>AgamOBP21

MQSLQIVFVVLLAAVSTMEQHEIAKSLAEQCRAELGGELPEDFATKMRLGDLTLDSETAKCTIQCMFAKVGFTLESGAANRDVLIAKLSKGNPTAKAEAFADVCENNEGETACDKAFSLYQCYHKNKSIFD

>AgamOBP22

MNSLLLIGGVLVVLNVQFVTAADNNESVIESCSNAVQGAANDELKVHYRANEFPDDPVTHCFVRCIGLELNLYDDKYGVDLQANWENLGNSDDADEEFVAKHRACLEAKNLETIEDLCERAYSAFQCLREDYEMYQNNNNATSE

>AgamOBP23

MKSFFCVASFFLLVASVHAFTLRQQKMVSIFALECMAETGIGAESLTKLRDGDLTANDRTAKCFMKCFFEKENFMDAEGKLQLEAIATALEKDYERAKIDEMLEKCGEQKEDACETAFNAYACYHDHYQNL

>AgamOBP25

MKFLVFAIVLSAICLDALVDGAAAPPPDLEDVSKIANGEAFALECLIESGLKLDSLAALSAKELDTNGSKIKCLVKCFFEKTGFMNKDGQLQEETITEQLSKFMPRERIESLVKNCNFQEADACETAYKVTECYFQNKAGLF

>AgamOBP26

MKTFVAIAVVALIAGTFALTIDQKKKAEGYAAECVKTTGVPPETAAKLKGGDFAGADDKTKCFAKCFLEKAGFMTDKGEIDEKTVIEKLSVDHDRAKVEGLVKKCNHKEANPCETAFKAYQCIYAAKGAVV

>AgamOBP27

MGRLDLVCLLAIVLLVHSCVSIAWSFSWACTMVKPFVFLLYRMDRTSWDHTSGVAMKPSCFGECFVKRAGFMNDNFTFNRDTIMRFTNRFVSKEISEKVYNICTDNVTPTYCVTAFDVYQCIYENVYKSWDSRK

>AgamOBP28

MKLLFATVLLAVCAAAQPLTDDQMKKAEGFALGCLEQHKGLNKEHLVLLRDGDFSKVDADTKCFLRCFLQQANFMDAAGKLQNDYVIERLSLNREKSKVEALVKKCSAGVEVEDSCETAFRAVECYHREKASLL

>AgamOBP29

MDENTPQKRCVSRAVTVGICGAIVLLLLVGTSPAPVEGLRCRTGEGPSADDVKRIVRTCMNKITNAGGGNFSSSSSSSTIERDRACLMQCFFEEMKATNADGFPEKHKVLHVITKDIREHELREFYVDSIQECFHMLGLDNRLKDKCDYSMRFVTCLSDRFETNCDDWESVTSAMF

>AgamOBP30

MVTQLSQPLPLRGQHTMATVNLYYLGLVCLLAVTATAASQCFRDAGQLKRVVQAQEECVRYLRIPCARLAVYNKFIYPNDAETQCMVRCMGLNLGWWNDTHGVQEASMRSFFHPDPNDCDYERRTYRCLHSQRLDRPAPHDEACERAYESFRCYYEHYGNLVVTPQFVRLNALQQLDVLLQCADMLQYPMPDRSFSCAKTHVAGAEGDFDCVLRCYMLRTGLYSEQYGPNLDRIYVQCNNYANETVFRETTDACYQRLRSDCQDECTLIARYVRECFPAGGIIFLNSLW

>AgamOBP31

MKQLVLLTICVLALMPLEVLSNDTKGLTIEKSFLQSVHDCAEYLQVPKHRLVQYLAYEFPPDEETKCLIFCVGTDLRWWNNTCGLQVPAIMNYFQPVLGDRQYEKRTSECLERNVHTAELPNNCCQAYETFQCYFREFGNLVTCPQYVPATKLQATQAALDCLTVLRVPTDLLQCYSKGDLPDVPETRCLYHCIDHRTGLYTTESGIHLSRFYVRDLEVNDLRYLSKETKACRDRVRMSGCDVCSEVYNTHRDCLSGIGVDGYTSGIIAEASRIALTNLATALSALPARSYAQRSPYPSFHRTCKAEHFGRSF

>AgamOBP32

MISIELKYITLACVLAATVTAGSHCHNDYYQLKSVSQAQEECARYQGIPCARLAVYNKYIYPNDTQTQCMVRCMGLNLGWWNDTHGVQEPAMRSFFHPDPDDCDYERRTYHCLNSQRLNHPSPHVDVCERAYESFRCYYEQYGNIVVTPQFVPLSDLQQVDVLLQCANMLPLTVGRSCAGGSKPSERDVDCLARCFLLRSGLYSEQHGPHLDRLYVQCNNYANETRFRETTGTCYRRLKSECQDECVLAGRFLRECFYEGGISIVNSLPASEASVESAGSLGSGQGSAELGESHQEKVLQTWKDLYDRENLQDLWDRQEL

>AgamOBP33

MATIKLKYITLACVLAATVTAGSHCHNDYYQLKSVSQAQEECARYQGIPCARLAVYNKYIYPNDTQTQCMVRCMGLNLGWWNDTHGVQEPAMRSFFHPDPDDCDYERRTYHCLNSQRLNHPSPHVDVCERAYESFRCYYEQYGNIVVTPQFVPLSDLQQVDVLLQCANMLPLTVGRSCAGGSKPSERDVDCLARCFLLRSGLYSEQHGPHLDRLYVQCNNYANETRFRETTGTCYRRLKSECQDECVLAGRFLRECFYEGGLLGSIPVLGGLGGLVGGLTGLTGLVPPVTLQLTSPGLAAVTLTLSAPSVMVGALPVPPVMVGTLGGAANVGIL

>AgamOBP34

MQFQLNCVQQATRATMNSFALSVFVLAVGAVSVSASLQHYVVEKSFNQAQAECAEYQGVHDDDLLRYVKEGYPDVEEVRCLLRCVAFNLRFWNHTTGLQKNMVAGHFVPYPDDFHNVERTEACLAENLYTCDDDLCTQVYKAFQCYYQYYGALSECPQFVVNSYLEDLQVAYDLFGMLAVSQSTLQSLAGGCFPSGEESLCFFYSFVTRSGLYSVEDGAKLERLYYQYKEEVFNPNNAQTVACLQNQKKLACKKSTCQQAYDTFQNCFGESRGLEYLLHTVFVDAAKAFLGQPVCYCNKVKTCPLHKCYGR

>AgamOBP35

MNFFTVSAIALVAIIGSIQAEHSPLPHYFVRKSFPEAQAECAVYLQVPDDRLQRYMREGYPDEPEVHCLVLCVLENLRAWENGTLHENVLANYFVPATEDCDNAKRTERCLVYLPQECNGEPCVQAYRAFQCYYQNYGTLTTCPEYVPSYYGEDLQLAYDLFDMLDVSEDTRRKLAGGCFPSGPESQCFFFAYVTRFGAWSKDAPLLHNLYTQSQEDAFKKDNAETNVCLTNLNKLACHKTRCEHATDVFSQCFGNTDLYKHFLAVFKDAAMTYTRQ

>AgamOBP36

MNFFTVSAIALVAIIGSIQAEHSPLPHYFVRKSFPEAQAECAVYLQVPDDRLQRYMREGYPDEPEVHCLVLCVLENLRAWENGTLHENVLANYFVPATEDCDNAKRTERCLVNLPQECNGEPCVQAYRAFQCYYQNYGTLTTCPEYVPSYYGEDLQLAYDLFDMLDVSEDTRRKLAGGCFPSGPESQCFFFAYVTRFGAWSKDAPLLHNLYTQSQEDAFKKDNAETNVCLTNLNKLACHKTRCEHATDVFSQCFGNTDLYKHFLAVFKDAAMTYTRQ

>AgamOBP37

MQFQLNCVQQATRATMNSFALSVFVLAVGAVSVSASLQHYVVEKSFNQAQAECAEYQGVHDDDLLRYVKEGYPDVEEVRCLLRCVAFNLRFWNHTTGLQKNMVAGHFVPYPDDFHNVERTEACLAENLYTCDDDLCTQVYKAFQCYYQYYGALSECPQFVVNSYLEDLQVAYDLFGMLAVSQSTLQSLAGGCFPSGEESLCFFYSFVTRSGLYSVEDGAKLERLYYQYKEEVFNPNNAQTVACLQNQKKLACKKSTCQQAYDTFQNCFGESRGLEYLLHTVFVDAAKAFLGQPVCYCNKVKTCPLHKCYGR

>AgamOBP38

MLTYRAWLLLALLGAQCALILGAPATGHGYDTKSFAQAYLECLRYLNISRQSLYAYDSAAVPLNCGSNCLLRCIGLNARWWHDETGLSERALVRFFRQAPADSLLQARACVAELPAPPADSCAGAYWSFRCYSDALGELIAHPAYVAPCGQEIRRAVSDCATMLQVEDGQLQTCVRTETFLRQGNGAALLRCVVLRLGLYADSTGVLCDRVRLLMDADTAEQWTVARAEEAKRCEEDLRALGADTCVVAAHAVELCYGWPAFGELWEVLKQEYGSSDDALAEESEQVVVRRSCTPWMRPLKMGRNRQKARPRRMRKSSCLKMLNRPNWTWQTGRSR

>AgamOBP39

MASSGQVVAAAAVLLLMQLQTVTSATFGARDPPPPALREAQAACVKYLGICENRLHQYNNSVYPTDQDTMCMVRCAGIMVGFWDDCQGLKLDGLANLFPALAANDRVRYQIMSCAEKRIATCPPQDTCARAYNGFRCFLDAQKGGFGAKDMQPQQSTPPQPFDAQEFIRSLSICAKLQRIPKDRRDLYVQGVFPNDDKTRSLIRCVGIRTGLYDDEQGPNIALLYSLFGAGQSESEFRRRANLCIDANQPLLEAQDKNAQAYVKLYRCFADQISALVRANANAMA

>AgamOBP40

MERDRSSSYVAAALLLVCISLASAPRGTEANIFGGKLYQKAQQDCILFMGINPLRLDQYKKFVYPPDRDTMCLIRCIGISLDFWDDILGFDVDLAEQEFSPLVDATFKKYLAGNITLKLELLDPLDNCARAYYAFRTFRAQIRQFIGTGTTTMAPSVNFQPLTAVQILDIIVDCAREVNLPPSFLTSLTKGIITDCPEVQCLIRCAAVRTGLYTDKDGALLANLHRQLDPPGEDLASFSLRQGMCLQRNQQPPTADCCTRAFKQFFTCLRPDFEQFFIRNRETVMQHFLYKTDQPAEDRQPPWCRTMCWIRSGAIWV

>AgamOBP41

MGYWALGTGLQLLLLILVLGGSELQVKAKGSLILRSFDEMVLECAELMSIVHSKLARIRSGVMLPDEDTKCLIRCVGISGRFWNDHTGLRKELLARYFVTDPADAYNVNRTETCLQELPALELNAEKCCGLAFESFLCYYYNYGNLRQDSVFVPLDHLQLQHVTSRCMDVHQITTEQLMSLSAEAMDANDKLHCLVRCIGLQTGVYSDREGVSIDRLNAQYGEGHCEKEFKTHAVECITKHRELAYGSPCKRAYHLLYKCFENVRNVISAYELPDSDGN

>AgamOBP42

MFTTRLLVGALVSLGLTACSFAFTEHGAIVQSIVQAQHECVTYLNLPKHRLYQYLMYNYSNDAKTKQMLRCVGLILQWWKSDGTLNEHVLAQYFMPDTSDSDYYNRTYRCIERKAPVDDDLCSRAFETFQCYLQQYGELLNCPKVVPLSDERLTETMHFCLDVLDIPFSDFEQWTSSSELFLHTEPARCLLRCFTIRAGLYSDQHGPFADRFKLQFGAPKPDVFDNELEGDYCVARLRREGHDACSLAARSLYECYYFADTLLPTFERILPLLRLVLHQPEVETAEME

>AgamOBP43

MCSNRSAFGLLLLAWLASVTILGVEAYATPPPTTANCTTVSTFDAALQECVVQLGIAPERLDQEYNLLLYPADRDTMCLVRCIGVLLRFWNDTTGLREATIRQYYEPAPEDQDYQNRTRSCLAALEPSVTDVCERAHRSFLCYHQHYGYLRKTDRYVPKTPLEMKQIQQDCVDVYGLDPARLNHYQDGQFPDDPETQCFVRCVGLRAGLYTDRDGPNIDRMYVQCESCADETLFRAKAGECIAAQRRHKLSKCTAAYRTLYHCFRDDQLDLYASLTTAAATAAAMTTTTTTKKSTPPNAIPALSVRKPSDRAKLSPDAWQLEIILEGLYNQKY

>AgamOBP44

MKQLVCIVVFALVTPNLIVAECDTKGLIVEKSFLQSVHDCTEYLQIPKERLGQYMANEFPPDDETKCLLFCVGVDLGWWNNTCGLQVPAIVSYFQPVQGDKQYEKRTKECLERRVGAIDSPNSCCQAYETFQCYFQEFGNLVTCPQYVRSTKLQATQAALDCLVMLRYPEKLLKVYASGKVEDSPETRCLYHCIDLRTGLYTQNGISLPRFFVRDAAYNDLRYLSKETKACRDRIRQSGCDKCSEVYNTHTECLSGLGEKGYTSGIITAAAKIALTNLCPAVALSYGGRKPSSTCSKASGTGQVYNLSYPGYKSRMSSCSRCGGRGH

>AgamOBP45

MQRRNASGGGVAVLLTAIMALLPTGCDASLDVPHLTLSKSFSRALQDCMEYLQVPGYRYAEYAANSFPDDPETKCLLRCVGLNLRWWNDTTGMQTAVIEGFFHPDPLDELYENRTAECLRKELSHADTTDCCCLAYDSFRCYLQHYGNLVPCARFYPEDETRFVRAAQDCIEFLQIPHKLLKSYSAGSFPDAPETRCLLRCFFLRTGVFHVDTGFDVERLYTRDYEQPDERYLAQETEARLHKLRGSTGDQCTEVYLAYRDVLGELGRAYYEYDVLQAAAAKMTVCEVAVEPPAMTTTTTTTTTTPTTATACPSTTEFNYKELNCQNCGRLFISNNGRVSCCRCMKSSTPFGKFFF

>AgamOBP46

MNPIVGKVFLVLCGSLLVTGAPNTCGKLDLKTDPFTCCTIPKLLDVTIVSSCFEKFPIDKDAADKGAASMPKTEVTDCMSECILNSTGIYNRRGDVDEKKLNSVFTDSLPANSPWLNVVRKAIKECTAKADKKDKEFQKDVADQKKATPKGTQVCNPEASFLVDCIHTTVFSDCPTNLRSTSTECDAIWNFLKNCPFSALRQ

>AgamOBP47

MKHLKAFDEAQNDIKAVQKRLSTSSTILSGIQKNMAHLNLLQIGVLSLIAVGSVFAGNPCLKGPPVPKNAAECCVTPFLVEPSAFMTCHSKWIGQTKRQMAMEGIPRGCCVAECVMNSTSLYSNGKIDREALTKLYLASTKSMAPEWNKITLDAIDGCFKMADTIKDEIEAGAKLTPAFEGEQICHPISGTILACMGMTLFAECPAKLFTVNDDCNKLKSYHSKCPFL

>AgamOBP48

MGQRQRVVVQLALCFLTFGALLQAGVLAGDNPCAAGPPVDTNPAECCPTPMLVDGTIMMDCYKKYGEQTKKQLQMDGIPRGCCIAECAMNATNMYADGMLKRDDLSKMFMDAVKDKPEWMSLVRDATNACFELAEKKMDEIEAGAKLEPSFEGEKICHPISGTILRCMGMMMFAQCPASVFNVNENCNKLREYGSICPMI

>AgamOBP49

MEWNWTFLFRSFLLLTLHLLPQSVADDCIDMDLHSMEVARCCRYEPISTEEVAEKCYQELAPNIPPNSSDFPVCFIDCSYRQMGYITNEANEIDQSKYGQFLAGFDTAYKIAVERAVAACATVQEDIRRDVANVPSKCNAFALLFHVCVTQITLKHCPDDRWTASEICGKVRMGVPPCA

>AgamOBP50

MHVALPFSVVGKLTCLSPFLQSIKVASCCQLEAFLTLPTYGNCLQTIAEKYPDALWQGTVCAFDCTYREMGILTGVDDINVEQISTNQAGYDQAYQEAIAKAVTACMAQKDKIREEADVVQSECSMFAVKFHACVSLETMRNCPAERWDSSVLCEKVRSGVTVCPL

>AgamOBP51

MCHRVLSLCGFLLLGLQCGWQTLAEDCMDIKIFVSETLQLFRLDGASPKFTAFSSFLQTTKVASCCQLEEFLTLKTYGNCLNTMAEKYPNSTLDYLVCGLDCTYREMGILTGVDDINVEQISTNQAVYGEAYQEAIGKAVDACLAQRDEFREQEKFTKSECGMFALKFQGCIMVESMRNCPAERWDSSVLCEKVRSGVAVCPP

>AgamOBP52

MLFKLFTIPFRCPLFFSKHPKQFPPSKKQSELPYCCQTEPLIPEHVSTKCKEREAANHNPGTELFEVCYQQCIYEELEAVDGLEIRVEKLYALAEGFPADYRHAVHLAIDECVKRLRKTRHMFEQMNAQCSLFGFAVDRCVRLLIYENCPTARWSASVACTKSRQGVPFC

>AgamOBP53

MSFRSISALVILLHLFVICTPMPECISQTQKFEVPHCCQMEELIPRPSRTKCQEKAAIDHNPGFQAYFVVNCLAQCQLEELEVIDGEELHLEKLYPLTAKFPADYRHAVRQAIDECDAWLQGKKKERRRPDGKAHCPLIGMEVENCLHRTTFSNCPNSRWKASITCNKVRQGLPFC

>AgamOBP54

MDLKKSVAVVFVSFGWMMLLATAADPDCENLKNRREEMEQCCQVNMIIPLDGAEDCSSSVDETSEPHDKMMCTLECKLKSLGLLNGDDLVEAKVQEYIDRLEGDWKGTAKTIATECITTITEMKKKIQERDHDMKCSPVGAFFMMCLMKHTQAKCPEDKWQNTSFCNKMRSGECFPKRGRQ

>AgamOBP55

MLPTGLERTVLWVTVIVLVKVMVKSDAQVCCMVEHTFPQEPYRVCHEQHATPQMDNGTVMCIHQCYYKAIGMFAADGKVNTDAYIKYRDELDPTLRDAFSYSMVVCAKIIAKRMNNNIAEVNRMRCSPLPYLFNRCLMEVGIGNCPPERWMNCKHG

>AgamOBP56

MLKLALFVGLVGCVVAYDFQDSFYNEVLMEDLLDNADEPIMFGRFRRSASEVQDDKCKRKYKCCNDANTENMEKIHEIKKQCFMEVRNKNKADGAYEPVDFFSCERLNKTKMEVICAMECVGRKKEVVNEDGTLIEPKLMEFVKSNFAADDWQQPLLAGHIETCVKEAKEKAAKMPREAGQCSSETSNFGYCMWRQMALACPKDKQVANKRCDRIREKLANNEPLHYYKAELEDM

>AgamOBP57

MGKVLILFVGALVVASVTAGRFERSVFAPRIKRDATMRCCNDGFEKSEVHAKFAEVRTACMEELGLGETTHEELIKNREHLNCITECIAKKEGIADENGALLHTDLAKVVLEHMSTIEWKVPLAEGFIQQCFDEVELTDGAFVPSDEAKCNPEGFDFVFCLWRQFTLACPEEFRDDSEKCVELRDKLTNKEDVSDLHDDIEAAE

>AgamOBP58

MSLHLFVRTSTTHGINMRSSSVWLIVVCAVTVASANSEELLRGKENCLRHDDFPSPNECCSKPQWINRYAVRRCRYIHAEVDGSRYERGSCEARCGLFKINMTMTDRIQRVRVYRPRLQTRGIDQGWINVVLKALSYCKPKVTQLQGRHVRTDEEMEQCEIAEDIFGDCVQAQMFMHCPRATWIESRSCQTMRELLATGCPYKTLGEVVVLNDEGYVRDDRILEEEYDRPYRGRGRTESPRYDYDDNDGYSRGGQYDQRGGNYPRGTERNRNGNGYGAGDDGGYVV

>AgamOBP59

MPRLLPEQVIETCRARPLPSVIPGVPDPLPENCIAECALNETGILFNGQFRVEQAVKALSTQVPNDTLTWQHVIEVASKKCYIITVGDSFYLRDVAKNLISPQCIPSSFRFLQCTFSIVYRDCPDIYWNYQNDRCGQFVVALNNCHYLFRHIWDI

>AgamOBP60

MLSFVFLASIIVGLVSSQPPAPDASCFQPTAVTAEDCCKIPKPIDNAIMEKCRAENPKPGQMPAPGVPRTEGCCIVQCAMMETGGFVNNALNTDAIKRSMASTLGADSNFGSLVNGAVDTCARQIQNDPAYSVAPISSSPDRAGCSFIPQGFVNCLYTALFKSCPAATWTESSDCQALKTKLDSGCPFFLLMGRGPRN

>AgamOBP61

MNRLVCAFGVIFVVATLELVLAHPGKDVLGCHNGTSITVDECCAIPMLANKTVIEKCKAAHPFKPPQNTDDKGPRGHPGECLAECIMKGMGALKNEKVDGPAFRKAIEPVVKANPAFAKLLDDTVKQCHESINVDSEFTRYVTKPVCKADAKAFINCVYGTLFEQCPTNVWTQKDGCTQLKDKIKKGCAYFALRKHGGRRMRPT

>AgamOBP62

MKQRCALAGCEKLLPAVLLLLFALQATVPEGTVAGCSMLNNDNAEQRGAAMLADPATVKQVPEVTMQDAIAQCNRSFIIQPEYLAELNQTGSFPEETDKIPLCFIRCYLKALGILTEDDKVNKEVALARNWATSGETVDECLEEMAGSACEQAYFFTRCVMTRALVDGKSKDNK

>AgamOBP63

MKTIACLVLASAFIACAVATISEEQREAARQLAGKCMQQTGASEDDVNRLRSGDTEGADRNTRCFVQCFFQGAGFVDQDGSVQTDELTQKLASEYGQEKADELVARCRNNDGPDACERSFRLLQCYMENRASLMF

>AgamOBP64

MGAFESGLGLLGWVAFGMVLLLAGRGCHAQDFKGAIDHCTKDFEMDMDIVVSLKYGDFTERDPLIECFTECLMKKSGFMYDDYTYNKTLIIGFAGRYLEPEGAQAVYDNCIDRFGQTVCVTGFEMYQCIHETAVSEWVSSNF

>AgamOBP65

MQLAICVWTAVCLQRNIIEGFLVELEAFPSSHQQPPKTSPPVRSCGETFNLTDPRTCCSIPYLLPADVVEPCLEIPLSPIDLAGESNVCVFWQCRAECALNRTEMLVDGHFQLETAMQQLTNATSEDSTLTKRIQYAIGACNELFLNCPPQYWTASDECNQLVRTLNNCPHFLVHTDTF

>AgamOBP66

MATTIARIGSANWAKVLVLLWLVQLATAGEPNPACKTMPTVDKDNEDKCCDVPEMFPNETLNACMEEYQKSSKPPLQKSCEITTCVLKKQSLIKSDNTVDKDKIKSYIKEMVKGSDEWKTLVEKAVLEECLPLMDKDPSNVLSKLKSSLGDCDPAPALTIACAAAKFYVNCPAKDRTKSPMCDEWRTFLSKCSNSLEDLNAIFMVLENQKTR

>AgamOBP67

MNPVVCAFGVIFVVVTLELVVAHPGKDVLGCHNGTSITVDECCAIPMLANKTVIEKCKAAHPFKPPQNTDDKGPRGHPGECIAECIMKGMGALKNEKVDGPAFRKAIEPVVKANPAFAKLLDDTVKQCHESINVDSEFTRYVTKPVCKADAKAFINCVYGTLFEQCPTNVWTQKDGCTQLKDKIKKGCAYFALRKHGGRRMRPT

>AgamOBP68

MATTIARIGSANWAKLLVLLWLVQLATAGEPNPACKTLPTVDKDNEDKCCDVPEMFPNETLNACMEEHQQSSKPPLQKSCEITTCVLKKQSLIKSDNTVDKDKIKSYIKEMVKGSDEWKTLVEKAVLEECLPLMDKDPSNVLSKLKSSLGDCDPAPALTIACAAAKFYVNCPAKDRTKSPMCDEWRTFLSKCSNSLEDLNAIFMVLENQKTR

>AgamOBP79

MDRLLLVLLSSASLLLTVYGIKHHIVTKSWSEAQSDCLQYLRVESPGRYLSHRYRDNQTSKQLIFCIILNLRIYDPTQNVLRLKAMGQFFNPDKTDTLYVNRTNACLLRVKVPPLVDSSEDSQLYSGVMGTLYEVFRCFYHCYGNINAIAPKLPPTVLELEKIQQECARMVGVSERLLDGGLQLSSHPRYSKLPRCIMLRSGGSVDYLTHRNNSSRRFKLKKNVENDTL

>AgamOBP80

MRRQYSMWASTVAVIACGSALMLLHPVGADAPKKRCLTKPNVSKKVDMVIHQCQEEIKSSLIEDALKIFTAEHGQWHDRRKRDEGGLDFSHPTIVSHEDKWIAGCLMQCVYRKNNAIDKNGWPTLDGLVSLYTDGVNEQGYFMATLRGVDRCLKGTSKKYQIKRNDAAENFEQCEVAFDVFDCISDMITDYCSGQMEDDH

>AgamOBP82

ICGAIVLLLLVGTSPAPVEGLRCRTGEGPSADDVKRIVRTCMNKITNAGTMGEWGQRDRNGEEQQMMRDYGRSHRRRKRQYYGGQTSGSSSSGSAGEHSYNGRASPQYGEAGQGGNGTRSGGNSSSSSSSTIERDRACLMQCFFEEMKATNADGFPEKHKVLHVITKDIREHELREFYVDSIQECFHMLGLDNRLKDKCDYSMRFVTCLSDRFETNCDDWESVTSAMF

>AgamOBP83

SITQEQLEKTARTFRQVCQPKHKISDEVADAVNRGVFADTKDFKCYVSCLLDIMQVARKGKVNYEKSLKQIDTMLPDHMKPAFRAGLEACKSAAQGVKDHCEAAAILLQCFYKNNPKFVFP

>DplaOBP1

MKFLVVFAFVAMAVCSIRAELTKEEAIAIVHECKEAEGASDADVEAMMKHEPADSKEGKCMRACALKKFGVLSEDGKLIKDASIELTKRFIKDEDKKELATEVIEACEALEVNEDHCEAASEYGACIKKEFELKELDSSAEI

>DplaOBP2

MSTITLVYGILLTATIFIVHTAWSQKVEPRRDDTYPPPDLLAKLKPVHDTCVGKTGVTEDAIKKFSDEEIHEDDLLKCYMYCIFDEMNVLHDDGEVHLEKVLDMLPETMHDIAINMGKKCLYPKGDTNCERAFWLHSCWKKADPVHYFLV

>DplaOBP3

MTLKAIYRTQLLYTILIVLSLMNRWTHAQEPRRDAEYPPPAILKMAKPFHDICVGKTGVTEEAIKEFSDGQVHEDEALKCYMNCLFHEFDVVDANGDVHLETLFASIPGSLRDLLVNASQNCVHPEGDTLCHKAWWFHQCWKKADPVHYFLI

>DplaOBP4

MKFLYICVVAVIMGVAQVQCDMKEDMHKLSQACVTESHATDEEITNYFKNGMKDEDAKDNVKCHMKCMMEKQGHLKNGAVDEEAVKKTLQSIPALKDHQDEINKAIADCKSKKGANDCDTAYQITKCMAAHKSAM

>DplaOBP5

MSSKLNLVILIATLIGIVKITDVFGGATEEQMWAAGALMRDVCFPKFPKVSKELADGIKAGNLPDEKDAKCYVNCILEMMQTMKKGKFLYESTLKQVDLLMPDDYKDEYKAGAATCKDVANGIKNNCDSAYAIFTCLKKEISRFVFP

>DplaOBP7

MSSILIKLKFIIIGLTCIREISAITMEQFEQSLDMMRNGCAPKFKVNLEQLDALRNGYFDETIGSEIRCYAKCVAQLAGTLTKKGDFSIPKATAQVPIILPPEIQETAKAALNSCKDIQKNYKESCDRVFYVSKCVRDFAPEVFKFP

>DplaOBP8

MLKMKPWLFNTICLIMTIFLITNNALEIPEHLRKHARRIHKRCQNQSDTPEAVIKESLKGGLPNNKNFECYIQCLFDIMGIMDESNTIHIDNLLQILPEEMHPMVTMLSQACGTKDGDGKCNIAFNTMQCYVENNPTIIKDQFEFLFGN

>DplaOBP9

MNFVLKLIIFLSVAAIVYSDDEKFKLDDLVEAMMGFTDECEEPKPTKENAKEVIKFVKDAQKPSKCLRYCLMSQFNLITEGETRLKKDETVKMMSMMYSDADKDLEEIVEMCNDRNEKEMDKCENAHLHGICIYEELLARDYKMPEFEE

>DplaOBP10

MKKGFLIDAAVPVVVVVILMSIFTCAQKPRRDENYPPPDFLKRFSIIHDVCVEKTGVTEDAIKEFSDGEIHEDPALKCYMNCLFHEVNVVDDNGELHYEQLKRLLPDNLKEFVQHIIDACESHVPQGGTQCDRAWSWHVCFKQTDPVHYFLP

>DplaOBP11

MKFIVKFLVFAAIAVALQKVVAAEDDAAFKEFVEKSQQCKDKLGISDEEAEEAHKAHQNGEEIDGKFKCFAHCIAEEMDVLDGTGKFDVAKMEAKHAMSGDELEKINECKGEHDMENDDCEYSYKMMGCLMSK

>DplaOBP12

MKFFVVLAVLAISGYAVNAQDPIKLTEEQKLIVLQHAAECAKQTSASKEALQDLRAGKFSNVDNNTKCFTNCFLEKSGFLVDGQVQPAVVSKKLGSIVGADKINTIMAQCNGSKGANNCDTAFELFKCYYLKNAAIL

>DplaOBP13

MKAFITLAVVCLVAGASALDVKLSDEQTAKATVYYNECIKEENVSEEDAMKLKNKDLASPSKNMKCFSACFFEKAGTFKDNELQADVVMEKFGAIIGEEKTKAILEKCKSVKSDDRCDTAFALYECFETEKSKLVAA

>DplaOBP14

MKIFVIICLIVCIDNTLQQSLDGLMEIVKASVENCYEDEANTKKIEVTEAGFEDIANGSRDAVRNAKCIRYCIMKKHNLFSPDNSLNESEIIPFFAYLFNNSIDNNHLKSIIAECNDVIVKENDRCERSYMANSCILQKLNESGMKDI

>DplaOBP15

MKATIAILLALFALVSADYKLRNQEDLMKARKECMEAKKVTAELIEKYKKFDFPDDEITRCYIECIFEKFQLFDPKDGFKNENLIAQLGAGKENKDEVKADVEKCADKNEQKSDSCAWAFRGFKCFISKNLPLVMESLKKN

>DplaOBP16

MLKFLPIFIVAFVAVKAYDFTDFDFNQYLFDEFVSMNDDDSVSRQRRDTEVMPAGGEKKECGGRRGDWKKDFACCAGDKFDPEHFKMIKETKKQCAAKLRANNSDVENFDPFDCEKMGRIKQLIICEGECFSKAMNILDEAGKLNREVIVKHLSEHWVNDSEWKKAAYEGYVDKCIAKAEAIEQHADQKCNSVPMEFHYCIWGQFINGCPADLRVDSSKCNKMRERHSQGTGSFLNKHVLHDFLHHGHGKTLGSEEKDM

>DplaOBP17

MSPKLNLMQMVITAFILSFVILCNGKNCHDDPVVLNEELFACCKGHPKYTSEPCIDALLANDTFSPECLVDCMYSQYKIYNGEDIDLAAVKIFLDSRITDEAFNVVYLHAYKKCSKLNKALIESKFSFIEIKNSHGCDVYPTFMEFCVWYHTIVDCPPKYATNDETCSHKRQWINECLLES

>DplaOBP18

MVNELRIIKILTIIMQIYVVAGIDCKTPVKVDPFSCCPVQEMISKEIVDKCTGNSKKNSTMATSVDSKASTTSTPTDPKPNGALMHSCINTCVLNETGVTTGMDFKLNETRLTEYLKLALNGSTDLIPVVENSFKTCSSELQKKMQDKMKNSPSTNMSQDGMMHNTNCSPFASHLLECVFLKSLKNCPKSVQTNTNICNEKIDYMKECIPDTFDNKENSS

>DplaOBP19

MKVFITLAVVCLVAGAFAVDVKLTDEQKAKSKVHFGECVKQENVSDEDVQKLKSKDFANPSKNMKCFGACFFEKAGVLKDNEVQEAVVMEKLGSIIGEANAKAVLDKCKNVKGEDRCETGFKIFACAEAEKAKLAAA

>DplaOBP20

MKAAALIIILNLGVLITAQSKTSDEFPATSEKCFQREHVPQSYKTNFHNFQYPNEEIVQKYIHCISSELEIWDNTNGFNLEKISQQYRSRANEEVVLPVISKCNRDNQNSNKPLWCYRAFLCILNTQVGNGLKRTYAANAKKVIYQMAIIKVNQ

>DplaOBP21

MKLQLAFLLFSLALVSAKFEIRDRADALRAHEECREDNNVPDDAYDKFLNYDFVDHKRTKCYVKCWVEKMGIFRKKGFNEADIVYQFTHNHPYYLQPVRHGLEKCIDHNESESDVCTFAHRVFSCWLKNISKLYVKFLVNKLCKI

>MdesOBP1

MMKPVRKTCQEKTGVTDSAIMEFSDGQVHEDAALKCYMNCLFHEFKVVDDNGEVHFEKIQTHVDRMDDEIRHIADTLLKKCQNPTGADQCERAFSIHKCWKTNDPKVISN

>MdesOBP2

MNTKFFAVFGICTILCCICCSGATETTVQPPDQKTSQMSNDQGDQLDQETIMMMCNETFRTPTEYLIELNTTGAFPDETDKTPMCYIRCYLENVGVMKDDKLDREKAIQMSWSTSEDALDECNKEVADRMNPCEKAYFLTRCVMMRNIVDTRTNNEEK

>MdesOBP4

MKLLVWWSVIGAVIGLSKQSLTPEQRKAFEEKLVSDCSKKEGATSDDLAAIAKREMPTTPTGKCMHACFIETLGFAKDNKVSIDGIVEIAKLSFDGDTGKVQTAREIGEECAGVNDSDRCEFAMKMIGCAETATKNRNINFSELFQ

>MdesOBP5

MWKLSAMLILCLCSKIEGQDYDCDQSPPISVEPLTCCKISKPFDRKNFPECFLDSVITTTIAIPSTTLPSIPVTELNDDYDYPINNFGYNGNRERPSYRDRKNEKRRNGGNNRYNNNEDNYRGDSSDNNERRTSNSKNNDYNENNENEDDYPWKHHHDHHHWGWGHHRFGGYHGSRHRRQAIFQPSAQRNQCAIECIFNQTSILSNGRFDRDIALEVYADYYPEVPIDLLTRTIDKCSKQLRNYRLGNRRRNRNPNYNKLPNRQCRNGSGLFATCFNREIYRNCPVELDNDSEDCLSLRTFIDNCSPIRGTREIGGIANVDVDEYGNIILDDPLN

>MdesOBP6

MNLLVFCTLLAVIATISAHGPMNDEQTAKMMEFKQQCADELKISPEDMNKMKSGEILANSNPNSKCFIKCFYDKAGVMVDGKINEAVAMEKIGQYVGPDQVANVFNQCKEAGGADPCEIASKFYECIHKNKH

>MdesOBP7

MVKLLVSVTLLAIVATISANNPINDEQKSKMMDIKQQCADELKLSQEDINKMKSSETANLNPSQKCFAKCIFEKTGVMVDGKINELVAVEKISLFAGPEQAANILNQCKEIGGADPYRKCVKKEYFFSVILLV

>MdesOBP8

MWSNISYKMDGNSVLGYQFLAAHCLVNTKTLRIKPNFFLFSWKTKSKTIFKMKFFVVAVVFTIVCGAFAVTDEQKQIAEKHVKECAVETSFTPEDLTKLRTNPKAAVGDAKAQKFSKCFLHKLGFINDAGDFQEHVVIEKLSKGGDRAKIEEIVKQCKSVIGANKDENPIKLYSCYLEKKALAA

>MdesOBP9

MKYILAVVLIAAAVYAADEWKIQTQENLVSYRPECVSSLSIGEDLVNEYKKFNFPNDEKTQCYINCIFKKMGLFDEKEGFNVERLVKQLGQGKNETVIRQEVIKCADKNPNKTGACQWAYRGFLCFKTAHLDLVQASVKKN

>MdesOBP10

MLLNVWSEKTLLIAAFLLAMTIQRSDGALSMKQFQQSLDMMRKTCLPKSGASESAINGLKTGNFPDDDKPLKCYAYCVLEMSGILTKKKEFNEAKVKTQAELMLPAELKEGTLRAWEACKAVSKNYKDHCDPLSLINGFKRGRMLTRAQTVIVRMFEAICNCTTQSAATLPTKVSSTRSLSTSK

>MdesOBP11

MHDTCVGKTGVSEDTIKQFSDGDTIFEDEKLKCYMDCLLHEMGMILPDGKIDLVNLHESFNDDKEIHFTFMHMIRKCLYPNGDGCERAFNLNKCFKQADPKVNYYN

>MdesOBP12

MCVCEHQINIQKKKIFINHCISFCVCIFKKKKIKKRTIYPILSKNNDKLIILNFARFQLRFFQAIKKFSDGEIHEDDKLKCYMNCLFHEASRDDVGSHDRNLHHGGSQYLQVDKKEKEKVN

>MdesOBP13

MTLKQFDQSMAMFKKQCLQKTGADPALIDGIHRGDFPDHIQELKCFSNCIAVAAGTVNKKGDVVEAKVMKQINTVLPEQLREYATASLQACKDISLKYKDPCDKSYYSTKCMYEFNPEKFIYP

>MdesOBP14

MFTIFKFICAIAFLCNSIMAGVTLEQMRQSGDVVRNVCQPRSKASDDLVLGIREGKFPEDKALKVYVHCVFEMMSVMKKNKVTYEPALKQFDALLPDDYVEPYKNGLTICKDSTGGVKSAPEVAYNLLKCFYANNPKFTFA

>MdesOBP15

MQSVTLKEFQHTLKIVHDTCQTVTKVEEELIQQVRDGTFVDVPVIKCYVHCFMEMLHLMEGTAGQFHVANKHTDYMIPAELFDTTIKAFGHCEGATNGMTDNCEAAYSMLKCFKADNNEFWFT

>MdesOBP16

MKYLIELNTTGAFPDETDKTPMCYIRCYLENVGVMKDDKLDREKAIQMSWSTSEDALDECNKEVADRMNPCEKAYFLTRCVMMRNIVDTRTNNEEK

>MdesOBP17

MPSKIDKNKYLCRSDDGPTDGELRKIARTCMHRIGGNETDTESEENSNENKSNDEYNDDHNGHSNDRFNSNRQYNNGNNYGFNFQSEYQNDPYANIGNIQTYQGRNGYMYNNWNNQNSPRRPNEMSYGNDHYNSQNTNRNRFDPRYGNVDTSNTGNYNSFGSKSNDRKFSNQNHNFKNNNNDNRTQQDRACLMHCFFHELKMTNNEDLPDQRKMLQVLTMDMRNHEVKQFYINSIQECFHLVDNLDNYNRRKDKCDMAYKIVSVLISRTNKLLFLLDSVFRSGIHLLKIMMACKNIRFINCRLPFPFSDAERSCGIWPDCNILVSRSTSCNDVAFLIASRIL

>MdesOBP18

MVKALDIPGHLQAPLRILHKACVSETGVAEEHIQNSKNGNLPNVAELKCYILCLLEHSGMIESDGTIHWEQVMHLLPESNQETARMVTDTCKTKHGDTRCETAYETIKCYFEAAPEVRY

>MdesOBP19

MDGPKRLLFPLLFLCLIYGIRSELTAAELMNMMEGFRASCQPKFNVTDDLLDGINLGRFPENNQLKCYVKCILEAMNMMKKGKINHELAIKQIKLFMPAGIREEYLNGINTCKDRGTVEADPLIS

>MdesOBP20

MKYLIELNTTGAFPDETDKTPMCYIRCYLENVGVMKDDKLDREKAIQMSWSTSEDALDECNKEVADRMNPCEKAYFLTRCVMMRNIVDTRTNNEEK

**Chemosensory proteins (CSPs)**

>BodoCSP1

MKYLVAFAFVAVVAMAAARPEDKYTTKYDGVDVDEILKSDRLFNNYFKCLMDKGKCTPDGSELKRVLPDALKTNCSKCSEKQKTGTEHVIKFLIDNKPAQWEALQNKYDPEHIYTTMFRDEAKNHGINV

>BodoCSP2

MNSKIRVLVCTLLVIISCGEHVVDGDDKNITRLLNNQVIVSRQIMCVLEKSPCDQLGRQLKAALPEVILRNCRNCSPTQAQNAQKLTNFLQTRYPDVWAMLIRKYRGV

>BodoCSP3

MTIKYCFLLLMISQILIVNKAQTKAYETKYDDIDLDELLKNDRLRHSYVKCLLGEGPCTPDGQELKNALPDAIQSKCSKCTEKQKAGAEKVTHYLIDNKPDEWQKLADKFDKDDDYKTKYLMEKEKDKSKSSEESKDSEED

>BodoCSP4

MNSITIIVFTLFVCATVAQEYTKKYDNVNIETILSNDRVITNYINCLLGKGACTKEGRELKKLLPDAIQTDCSKCTQEQKRNSRKVITFIRSRRPQDWAKLIAKYDPEGLFNKRSSFL

>BodoCSP5

MHRIFTVIGTILVYTKYAYCDQGIDLYSVRYNNLDIDTILNSDRLVTNYVECLLSRKPCSPEGKELKRILPEALRTKCGRCSNTQKEVALKVLKKLYVYYPKHYNDLRAKWDQTGEYHRRFEEYLREERFNSISGDTDRDQTQVVQKISPQNPLTTPASTTAHQISTLDTQRPARNPDDVQQNPTLNQFDLFNRFGGEDDENDQTAPIPSVQAPVRTFILNIRKRITSFLSLISP

>DmelCSP1

MKASLALVFCVCVGLAAAAPEKTYTNKYDSVNVDEVLGNNRVLGNYLKCLMDKGPCTAEGRELKRLLPDALHSDCSKCTEVQRKNSQKVINYLRANKAGEWKLLLNKYDPQGIYRAKHEGH

>DmelCSP2

MKMILALVVLGLVLVAAEDKYTTKYDNIDVDEILKSDRLFGNYFKCLVDNGKCTPEGRELKKSLPDALKTECSKCSEKQRQNTDKVIRYIIENKPEEWKQLQAKYDPDEIYIKRYRATAEASGIKV

>DmelCSP3

MGQPGFRRAIGHVSLVVALMCTTCFQVEGLPHPPATSPSPMMERMVEQAYDDKFDNVDLDEILNQERLLINYIKCLEGTGPCTPDAKMLKEILPDAIQTDCTKCTEKQRYGAEKVTRHLIDNRPTDWERLEKIYDPEGTYRIKYQEMKSKANEEP

>DmelCSP4

MLLLNKNRVISLVVNFIFLIILISSSVQADERNINKLLNNQVVVSRQIMCILGKSECDQLGLQLKAALPEVITRKCRNCSPQQAQKAQKLTTFLQTRYPDVWAMLLRKYDSA

>DsimCSP1

MKASLALVFCVIVGLAAAAPEKTYTNKYDSVNVDEVLTNNRVLGNYLKCLMDNGPCTPEGRELKRLLPDALHSDCSKCTEVQRKNSQKVINYLRANKAGEWKLLLKKYDPQGIYRAKHEGH

>DsimCSP2

MKMILALVVLGLVLVAAEDKYTTKYDNIDVDEILKSNRLFGNYFKCLVDNGKCTPEGRELKKSLPDALKTECSKCSEKQRQNTDKVIRYIIENKPEEWKQLQAKYDPEEIYIKRYRATAEASGIKV

>DsimCSP3

MGQPGFRRAMGHFSLVVALMCTTCFQVEGLPHPPATSPSPMMERMVEQAYDDKFDNVDLDEILNQERLLINYIKCLEGTGPCTPDAKMLKEILPDAIQTDCTKCTEKQRYGAEKVTRHLIDNRPTDWERLEKIYDPEGTYRIKYQEMKSKANEEP

>DsimCSP4

MLLLNKNRVLSLVVNFIFLIILISSSVQADERNINKLLNNQVVVSRQIMCILGKSECDQLGLQLKAALPEVITRKCRNCSPQQAQKAQKLTTFLQTRYPDVWAMLLRKYDSA

>DsecCSP1

MKASLALVFCVIVGLAAAAPEKTYTNKYDSVNVDEVLTNNRVLGNYLKCLMDKGACTPEGRELKRLLPDALHSDCSKCTEVQRKNSQKVINYLRANKAAEWKLLLNKYDPQGIYRAKHEGH

>DsecCSP2

MKMILALVVLGLVLVAAEDKYTTKYDNIDVDEILKSTRLFENYFKCLVDNGKCTPEGRELKKSLPDALKTECSKCSEKQRQNTDKVIRYIIENKPEEWKQLQAKYDPEEIYIKRYRATAEASGIKV

>DsecCSP3

MGQPGFRRAMGHFSLVVALMCTTCFQVEGLPHPPATSPSPMMERMVEQAYDDKFDNVDLEEILNQERLLINYIKCLEGTGPCTPDAKMLKEILPDAIQTDCTKCTEKQRYGAEKVTRHLIDNRPTDWERLEKIYDPEGTYRIKYQEMKSKANEEP

>DsecCSP4

MLLLNKNRVLSLVVNFIFLIILISSSVQADERNINKLLNNQVVVSRQIMCILGKSECDQLGLQLKAALPEVITRKCRNCSPQQAQKAQKLTTFLQTRYPDVWAMLLRKYDSA

>DyakCSP1

MKASLALVFCVFAGLAAAASQQQYTSKFDNVNVDEVLNNNRVLNHYLKCLMEKGPCTPEGRELKRLLPDALQSDCSKCTDVQRKNSEKVINYLRTHRAGEWKLLLDKYDPNGIYRAKHGAH

>DyakCSP2

MKMIIALFVLGLVLVAAEDKYTTKYDNIDVDEILKSDRLFGNYFKCLVDTGKCTPEGRELKKSLPDALKTECSKCSERQRQNTDKVIRYIIDNKPEEWKQLQAKFDPEEIYIKRYRATAESSGIKV

>DyakCSP3

MGQPNFRRAMGHISLVVALICTTCFQVEGLPHPPATSPAPMMERMVEQAYDDKFDNVDLDEILNQERLLINYIRCLEGTGPCTPDAKMLKEILPDAIQTDCTKCTEKQRYGAEKVTRHLIDNRPTDWERLEKIYDPEGTYRIKYQEMKANANAEP

>DyakCSP4

MLFLNKNRVLSLVVNFIFLIILISSSVQADERNINKLLNNQVVVSRQIMCILGKSECDQLGLQLKAALPEVITRKCRNCSPQQAQKAQKLTTFLQTRYPDVWAMLLRKYDSA

>DereCSP1

MKASLALVFCVVAGLAAAASQQQYTNKFDNVNVDEVLSNNRVLNNYLKCLMEKGPCTAEGRELKRLLPDALQSDCSKCTDVQRKNSEKVITFLRVNKPGEWKLLLNKYDPKGIYRARHEGH

>DereCSP2

MKMIIALFVLGLVLVAADDKYTTKYDNIDVDEILKSNRLFGNYFKCLVDTGKCTPEGRELKKSLPDALKTECSKCSEKQRQNTDKVIRYIIDNKPEEWKQLQAKYDPEEIYIKRYRATAEASGIKV

>DereCSP3

MGQPGFRRAIGHISLVVALMCTTCFQVEGLPHPPTTTPAPMMERMVEQAYDDKFDNVDLDEILNQERLLINYIKCLEGTGPCTPDAKMLKEILPDAIQTDCTKCTEKQRYGAEKVTRHLIDNRPTDWERLEKIYDPEGTYRIKYQEMKAKANAEP

>DereCSP4

MLFLNKNRVLSLVVNFIFLIILISSSVQADERNINKLLNNQVVVSRQIMCILGKSECDQLGIQLKAALPEVITRKCRNCSPQQAQKAQKLTTFLQTRYPDVWAMLLRKYDSA

>DanaCSP1

MKASLAIAVCLVAVSLVAAAPQKQYTNKFDNVNVDEVLGNDRVLNNYLKCLMEKGPCTPEGRELKRLLPDALQSECSKCTEVQRRNSEKVINFLRSHRAGEWKLLLDKYDSKGIYRAKHDAALKKQH

>DanaCSP2

MKMIFALLVLGVALVAAEEKYTTKYDNIDVDEILKSDRLFTNYFKCLIDAGKCTPEGRELKKSLPDALKTECSKCSEKQRENTDKVIRFIIDNKPEEWKQLQAKFDPEDIYVKRYRSQAAERGIKV

>DanaCSP4

MLFLNKNRALSLVLNFIFLIILISSNAQADERNINKLLNNQVVVSRQIMCILGKSECDQLGLQLKAALPEVITRKCRNCSPQQAQKAQKLTTFLQTRYPDVWAMLLRKYDSA

>DpseCSP1

MKASLAIVLCVLAGLAAAAPDQYTSKFDNVNVDDVLGNQRVLNNYLKCLMDKGPCTAEGRELKRLLPDALQSDCSKCTAAQRRNSEKVINILRSKYPGEWKQLLDKYDSKGIYRSKYEAAAKKQH

>DpseCSP2

MKMLLTLLLLGVVLVAAEEKYTTKYDNLDVDEILKSDRLFTNYFKCLIETGKCTPEGRELKKSLPDALKTECSKCSERQRSNTDKVIRFIIDNKPEEWKQLQTKFDPEDIYIKRYRAQATNAGIKI

>DpseCSP3

MSRTGIILFRTAVVVTLMCVFCIQINGLPHPPLTTLAPRLDGVYNEKFDNVDLDEILIQERLLNNYIKCLESAGPCTPDAKMLKDILPDAVLTDCTKCTEKQKIGAEKVTRHLIDNRPNDWERLEKIYDPEGTYRFKYLKSKANGNKSL

>DpseCSP4

MLSLNKNQAQFLVLYFIFFIIFISSKAQAEERNINKLLNNQVVVSRQIMCILEKSECDQLGQQLKAALPEVITRKCRNCSPQQAQKAQKLTTFLQTRYPDVWAMLIRKYQSV

>DperCSP1

MKASLAIVLCVLAGLAAAAPDQYTSKFDNVNVDDVLGNQRVLNNYLKCLMDKGPCTAEGRELKRLLPDALQSDCSKCTAAQRRNSEKVINILRSKYPGEWKQLLDKYDSKGIYRSKYEAAAKKQH

>DperCSP2

MKMLLTLLLLGVVLVAAEEKYTTKYDNLDVDEILKSDRLFTNYFKCLIETGKCTPEGRELKKSLPDALKTECSKCSERQRSNTDKVIRFIIDNKPEEWKQLQTKFDPEDIYIKRYRAQATNAGIKI

>DperCSP3

MSRTGIILFRTAVVVTLMCVFCIQINGLPHPPLTTLAPRLDGVYNEKFDNVDLDEILIQERLLNNYIKCLESAGPCTPDAKMLKDILPDAVLTDCTKCTEKQKIGAEKVTRHLIDNRPNDWERLEKIYDPEGTYRFKYLKSKANGNKSL

>DperCSP4

MLSLNKNQAQFLVLYFIFFIIFISSKAQAEERNINKLLNNQVVVSRQIMCILEKSECDQLGQQLKAALPEVITRKCRNCSPQQAQKAQKLTTFLQTRYPDVWAMLIRKYQSV

>DmojCSP1

MKASLVILLLGVFAALALARPEKKYTNKFDNVNVDEVLSNNRILNAYIKCLMESGPCTAEGRELKKLLPDALESECSKCTDVQRRNSNKVINYLRTNKPREWTMLLDKYDSKGIYRAKYEKHTKNN

>DmojCSP2

MKSFILFFALAIACVAADEKYTTKYDNIDVDEILRSDRLFTNYFKCLVDTGKCTPDARELKKSLPDALLTECSKCSEKQKQNTDKVIRYVIDNKPEEWKVLQAKYDPDEVYIKRYRAQAQKAGIPL

>DmojCSP3

MFRFTRTLLMQLQLLLLAVLLLLEVVPGRAHPPATTAMPAALMDASGSAYDSKFDNVDIDEILSQERLLNNYIKCLESQGPCTPDAKMLKDILPDAMMTDCAKCTEKQKYGADKVTRHLIDNRPEDWLRLEKIYDPQATYRIKYEASKTANGENPNMQVDATNQAEGDS

>DmojCSP4

MFFRKISEKSILVLIFLFMMLIPAQVDADEKNINRLLNNQAVVSRQIMCILEKSPCDQLGKQLKAALPEVITRKCRNCSPQQAQSAQKLTSFLQARYPDVWAMLIKKYQTA

>DwilCSP1

MKTSVAILVVLAFAGLALAAPEKQYTNKFDNVNVDDVLGNGRILNNYLKCLMEKGPCTPEGRELKRLLPDALESECSKCTEVQRRNSNKVINFLRSNKPGEWKLLLDKYDSKGIYRSKYESQAKKGH

>DwilCSP2

MKAAFVLLTLSIALVIADDKYTTKYDSVDIDEILKSERLFSNYFKCLIETGKCTPEGRELRKSLPDALKTECSKCSDKQKENTDKVIRFILDNKPDQWKQLQDKYDPEDLYIKRYRAKAAERGIKIK

>DwilCSP3

MSRSFDNRLAGRGTIQLLLMMLMALNVADAVPHPPPTAAAAPANNPPTAAYDNKFDNVDLDEILQQERLLNNYIKCLESTGPCTPDAKMLKEILPDAILTDCMKCTEKQKYGSEKVTRHLIDNRAKDWERLEKIYDPEGSYRIKYQESKNKDNDKDEKVVVNAMEKAQGDA

>DwilCSP4

MFLLNKNRTYLLVLHIIFLIILLTSKVNADEKNINKLLNNQVIVSRQIMCILEKSPCDQLGRQLKAALPEVITRKCRNCSPQQAQKAQKLTTFLQTRYPDVWAMLIRKYENA

>DvirCSP1

MKASLAILLVCVFAGLTAARPDQKYTNKFDNVNVDEVLGNNRILNNYIKCLMDKGPCTPEGRELKKLLPDALESECSKCTEVQRRNSNKVINFLRVNKPGEWKLLLDKYDSKGIYRSKYEKHVSH

>DvirCSP2

MKAYFLLLALAIAYVAAEEKYTTKYDNIDVDEILKSDRLFNNYFKCLVDTGKCTPEGRELKKSLPDALKTECSKCSEKQRQNTDKVIRYIIDNKPEEWKVLQEKYDPEEIYIKRYRGQASKAGINI

>DvirCSP3

MFRFDLSQLLLLLLPLLLAVLCRAHPPATTAMPAALIEAPGDVYDSKFDNVDIDEILSQERLLNNYIKCLESVGPCTPDAKMLKEILPDAMMTDCVKCTDKQKYGAEKVTRHLIDNRPEDWQRLEKIYDPQATYRMKYEETKTANGKSSNIELEALDQAQGDS

>DvirCSP4

MLCLKETQKYILILMFFLIIILPSKIDADEKNINRLLNNQAVVSRQIMCILEKSPCDQLGRQLKAALPEVITRKCRNCSPQQAQSAQKLTSFLQARYPDVWAMLLKKYQNV

>DgriCSP1

MKASLAILLCIVCGLAAARPDQKYTNKFDSVNVDDVLGNNRILNNYIKCLMEKGPCTPEGRELKRLLPDALESECSKCTEVQRRNSNKVINYLRTNKPGEWKLLLDKYDSKGIYRAKYEKHL

>DgriCSP2

MKATFLLVTLAIIVCVNGDEKYTTKYDNIDVDEILRSDRLFNNYFKCLIDTGKCTPEAKELKKSLPDALKTECSKCSEKQRQNSDKVLRHIIDHKPDEWKVLQAKYDPEEVYSKRYRAQAKEAGIHI

>DgriCSP3

MPARLINASDEGAAYDSKFDNVDLDEILGQARLLNNYIKCLENQGPCTPDAKMLKDILPDAMMTDCVKCTEKQKYGAEKVTRHLIDNRPNDWERLEKIYDPEGSYRIKYEATKTANDESQTVVFQPLAQGDS

>DgriCSP4

MFLIKKRQKEILIFIFFFIIIIPSKVDAEERNINKLLNNQAVVSRQIMCILEKSPCDQLGRQLKAALPEVITRKCRNCSPQQAQSAQKLTAFLQARYPDVWAMLIKKYQNV

>AgamCSP1

MKLFVVVALALVAAVAAQDKYTSKYDNINVDEILKSDRLFGNYYKCLLDQGRCTPDGNELKRILPDALQTNCEKCSEKQRDGAIKVINYLIQNRKDQWDVLQKKFDPENKYLEKYRGQAQKEGIKLD

>AgamCSP2

MKLFVAIAFALLALAAAQEQYTTKYDGIDLDEILKSDRLFNNYFKCLMDEGRCTPDGNELKKILPEALQTNCEKCSEKQRSGAIKVINYVIENRKEQWDALQKKYDPENLYVEKYREEAKKEGIKLE

>AgamCSP3

MKFFVVVALALVAAVAAQDKYTTKYDGVDLDEILKSDRLFNNYYKCLMDTGRCTPDGNELKRILPDALKTDCAKCSEKQKSGTEKVINYLIDNRKDQWENLQKKYDPENIYVNKYREDAKKKGINL

>AgamCSP4

MERFLLLLLFVAIVLGETANETYVTKYDNIDLEEIFSSKRLMDNYMNCLKNVGPCTPDGRELKDNLPDALMSDCVKCSEKQRIGSDKVIKFIVANRPDDFAILEQLYDPTGEYRRKYMQSDALAEHVKQEDRDLSSSGDGDADTETEAHATEHNSQDHDHREGQSDAE

>AgamCSP5

MRKVWLLASVVLAFLDFVKSQEVARTLYSTRYDNLDIDTILASNRLVTNYVDCLLSRKPCPPEGKDLKRILPEALRTKCARCSPIQKENALKIITRLYYDYPDQYRALRERWDPSGEYHRRFEEYLRGLQFNQIGGSNGGSGVGNTVLSNL

>AgamCSP6

MKHLTMVAIFAMVVVLASAQKYTDKFDNIDVDRVLSNDRILNNYLKCLLDKGPCTQEGRELKKTLPDALKTNCEKCSEKQRTSSRKVIAHLEERKPQEWKKLLDKYDPEGIYKSKFEKINKRS

>AgamCSP7

MLSAAVIVVMAALVIVGPQPAAANDSQNINRLLNNQVIVSRQIMCVLEKSPCDQLGRQLKAALPEVIQRNCRNCSPQQAQNAQKLTNFLQTRYPEVWAMLIRKYGAV

>AgamCSP8

MLHNLFLSLSLYVSVCGDPSGSTCAAEATTARTQVSDEALDKALSDKRYLMRQLKCALGEVACDPVGKRLKSLAPFVLRGACPQCTPAEMNQIKKTLAHLQRNFPSEWNKLVQTYAG

>AsinCSP1

MKLIVFIAFALVATVAAQQYTTKYDNIDVDEILKSDRLFNNYYKCLLDEGRCTPDGNELKRILPDALQTDCAKCSEKQRSGAIRVLNYLIQNRPTQWAVLQKKYDPENQYVEKYREQAKKEGIKLD

>AsinCSP2

MKLFVAIAFALLAIVAAQEQYTTKYDGIDLDEILKSDRLFNNYFKCLMDEGRCTPDGNELKKILPEALQTNCAKCSEKQRAGAIQVINYVIENRKEQWDALQKKYDPENLYIEKYREEAKKEGIKLE

>AsinCSP3

MKFFVVVALAMVAAVAAQEKYTTKYDGVDLDEILKSDRLFNNYYKCLLDQGRCTPDGNELKRILPDALQTDCAKCSEKQKSGTEKVINYLIDNRNDQWQNLQKKYDPENIYVNKYRDEANKKGIKL

>AsinCSP4

MLPLVLVVMAVMVLGSMADNYVTKYDNINLEEIFNSSRLMNNYMNCLKNVGPCTPDGKELKNNLPDALMSDCVKCSEKQRIGSDKVIKFIIANRPDDFATLEQLYDPTGEYRRKYLAPDGTLKPREDGEEDVPPVKETNDGDIEIDSVAHATEHKTAPSQDHDHGEGHTDESKN

>AsinCSP5

MRKVWLLASAVLAFLNFVKSQEVARTLYSARYDNLDIDTILGSNRLVSNYVDCLLSRKPCPPEGKDLKRILPEALRTKCARCSPIQKENALKIITRLYFDYPDQYRALRERWDPSGEYHRRFEDYLRGLQFNQIGGNGATNGGQAPSGGDNGNTVVENGGGNDRPVRNDLDRQPASQSSNVQAVVIDPTLSSGGRQRPNDPGPRHR

>AsinCSP6

MKNLSIVAVLAMLVVLVSAQKYTSKFDDIDVDRVLSNDRILNNYLKCLLDKGPCTQEGRELKKTLPDALKTNCEKCSEKQRTSSRKVIAHLEDRKPQEWKKLLDKYDPEGIYKSKFEKLNKRS

>AsinCSP7

MSSKALPNLFMLSAAVIALMAVLIAGPQPVVANDSQNINRLLNNQVIVSRQIMCVLEKSPCDQLGRQLKAALPEVIQRNCRNCSPQQAQNAQKLTNFLQTRYPEVWAMLIRKYGAV

>AsinCSP8

MSGKVSSRSSSSSICWFIGVGLCALVMVVQLAGLAEADATTTSRTQVSDEALDKALSDKRYLMRQLKCALGEVPCDPVGKRLKSLAPFVLRGACPQCTPAEMNQIKKTLAHLQRNFPSEWNKLVQTYAG

>CquiCSP1

MAVGVALAVLVLVLGGGQVAANDTQNLNRLLNNQVIVSRQIMCVLEKSPCDQLGRQLKAALPEVIQRNCRNCSPQQAQNAQKLTNFLQTRYPEVWAMLIRKYGAV

>CquiCSP2

MKSLVLSVLCLATLLVATTVAQQPRQYTDKFDNINVDQVLSNDRILSNYIRCLLDKGPCTQEGRELKKTLPDALRSNCEKCSEKQRNNSRKVISHLEAKKPADWKKLLDKYDPEGLYKSKFEKLNKRS

>CquiCSP3

MRNVWIVVASGLLAFANFVKSQETGRTLYSSRYDNLDIDTILSSNRLVNNYVDCLLSRKPCPPEGKDLKRILPEALRTKCGRCSSTQKENALKIITTLYYSYPDQYQALRERWDPSGEYHRRFEEYLRGIQFNQIGGNGNGNGGPSERPVRNDFDRDQSQILLQTLILSTTTAQPPVPEQRPTQLASAHSGGVSGDEAGGGSEPPTSIPATDSSPYIPLSGNPHKQ

>CquiCSP4

MFSNIVILGAVLLCCTLQAAVNGAEYDTKYDNVDLDEIFRSTRLLNNYMNCLKKVGPCTPEGKELKENLPDALANDCAKCSDKQKAGASKVIHFIVENRRDDFGALEKLYDPSGEFRRKYLDEQMHFRLHREEGGSAAVEEKSPASESEATTEEAAAAAQDHGQSAEGRR

>CquiCSP5

MKLYIVVALALIAAVAAQDKYTTKYDGIDLDEILKSDRLFNNYFKCLMDQGRCTPDGKELKRLLPDALNTNCSKCSEKQKQGTEKVVNYLIDNRPSQWKTLQEKYDPDNTYRTKYRVEAKKFGITV

>CquiCSP6

MKFFIVALALFALAVAQEDDGDKYTSKYDKIDLDDILGSDRLFKNYYNCLLDQGACTPEGNYLKRVLPEALETNCAKCTEKQDADSTKTIKYLSENRPAEWKVLKAKFDPENKYVEKYVDKAEKEGIKL

>CquiCSP7

MKFFIIALALFALAVAQEDDGDKYTSKYDKIDLDDILGSDRLFNNYYKCLLDQGPCTPEGNYLKRVLPEALETNCVKCTEKQDADSTKTIKYLSENRPAEWKVLKAKFDPENKYVEKYEDKAEKEGIKL

>CquiCSP8

MKFFIVALALVALVAAQEEEGDKYTTRYDNIDLDEILKSDRLFKNYYACLVEEGRCTAEGSYLKRILPEALETNCAKCSDKQRDDGVRAIKYMAENRAEEWKVLKARFDPENKYVEKYLADAEKEGIKL

>CquiCSP9

MKFFIVALALVALVAAQEEEGDKYTTKYDKIDLDDILKSDRLFKNYYACLLDDGPCTPEGSYLKRILPEALETNCAKCSDKQRDDGVRAIKYMAENRAEEWKVLKAKFDPENVYVEKYLADAEKEGIKL

>CquiCSP10

MKFFIVALALVALVAAQEEDGDKYTTRYDNIDLDEILKSDRLFKNYYNCLVDEGRCTAEGSYLKRILPDALETNCAKCSDKQRDDGVRAIKYMADNRAEEWKVLKARFDPENKYVEKYLADAEKEGIKL

>CquiCSP11

MKLFIVGLALFAVAFAQDAEVETLEEIGEKYTTKFDKIDLDDILKSDRLFKNYYNCLMEEGPCTPEGNYLKRVLPEALENSCNKCSEKQQKDSVKAIKYLTENRSEAWKVLKAKYDPENKYVEKYLTDADAEGIKL

>CquiCSP12

MKLIIVLALVALAAAQVPFTNKYDHINVEEILMSDRLFKNYFNCLIDEGACTPEASELKEKLPEALENNCELCTEKQKDTSVKVIRYLIDKRPVEWGVLKTKFDPNNKFVDRYREEAEAAGIKL

>CquiCSP13

MKLLIVFALVALVAAQDSTYTNKYDNIDVDEILKSDRLFKNYFNCLIDQGPCTPDATELKQSLPDALENNCSKCTPKQKEVGYKVVGWLINNRPEEWNVLRAKYDPENKFIEKYRDEAKAAGINL

>CquiCSP14

MKLLIVFALVALVAAQDSTYTNKYDHIDVDEILKSDRLFKNYYNCLIDQGPCTPDAAELKQSLPDALENNCSKCTPKQKETGYKVISSLIENRPAEWAVLQDKYDPERKFVEKYREEAAAAGIKL

>CquiCSP15

MKLLIAFALVALVAAQDSTYTNKYDHIDVDEILKSDRLFKNYYNCLIDQGPCTPDAAELKQSLPDALENNCSKCTPKQKETGYKVISSLIENRPAEWAVLQDKYDPERKFVEKYREEAAAAGIKL

>CquiCSP16

MKLLIAFALVALVAAQDSTYTNKYDHIDVDEILKSDRLFKNYYNCLIDQGPCTPDAAELKQSLPDALENNCSKCTPKQKETGYKVISSLIENRPAEWAVLQDKYDPERKFVEKYREEAAAAGIKL

>CquiCSP17

MKLLIVFALVALVAAQDSTYTNKYDHIDVEEILKSDRLFKNYYNCLIDQGPCTPDAAELKQSLPDALENNCSKCTPKQKETGYKVISSLIENRPAEWAVLQDKYDPERKFVEKYREEAAAAGIKL

>CquiCSP18

MKLFIVFALVALVAAQDNTYSSKYDNVDIDEILKTDRLFKNYYNCLIDQGPCTPDATELKQVLPDALENNCSKCTPKQKDAGYKVVGFLIDNRPEEWAVVRAKYDPENKFVEKYRGDAEAAGVKL

>CquiCSP19

MKTFIVFGLLALVAAQDSTYTNKYDHIDVEEILKSDRLFKNYYNCLIDQGPCTPDATELKQSLPDALENNCSKCTPKQKEVGNKVVAYLIESRPEEWAVLKAKFDPENKFVEKYREEAAAAGIKL

>CquiCSP20

MKFFVAFFALVALVAAQELYTNKFDTVDLDEILKSDRLFKNYYQCLLDEGRCTPDGNELKRVLPEALETNCAKCSEKQKTAGNKAFKYLAANRPTEWKALLAKFDPESKYTAKKLDIKIFIPFNY

>CquiCSP21

MKFFVAIFAALLAVAAAQELYTSKYDSVDVDEILKSDRLFKNYYQCLLEDGRCTPEGNELKRILPEALETNCAKCSEKQRSGAIKAFGYLSENRPEEWKALRARFDPENKYLEQYREEGEKNGIKF

>CquiCSP22

MKCVIVAVLALIALVSAQEAEQYTTKYDNIDLDEILKSDRLFNNYYKCLMDEGPCTPDGNELKRILPEALQTNCAKCSEAQRAGAIKVINHVIDNRPEQWKALQAKYDPENIYVEKYRTEAAEAGIAL

>CquiCSP24

MKCFIAFALLVVAVAAQNKYTTKYDGIDIDEILKSDRLFNNYYKCLLDQGRCTPDANELKRILPEALQTNCAKCTEKQKDGAVRVINYLIDNRSAQWQVLQKKFDPENVYINQYRNEARAAGIKV

>CquiCSP25

MYCKAVTTKMSSHQQQQQRRRHRTPVYCDQLASWLLLAVFAVSTVLLCCCVTSAQAQPQTPPTKSQVSDEALDKALSDKRYLMRQLKCALGEVPCDPVGKRLKSLAPFVLRGACPQCTATEMNQIKKTLAHLQRNFPQEWNKLVQTYAG

>CquiCSP26

MKFFIVALALFALAVAQEDDGDKYTSKYDKIDLDDILGSDRLFKNYYNCLLDQGACTPEGNYLKRVLPEALETNCAKCTEKQDADSTKTIKYLSENRPAEWKVLKAKFDPENKYVEKYVDKAEKEGIKL

>CquiCSP27

MIPSKYDKIDLDDILGSDRLFNNYYKCLLDQGPCTPEGNYLKRVLPEALETNCVKCTEKQDADSTKTIKFLSENRPAEWKVLKAKFDPENKYVEKYEDKAEKEGIKL

>AaegCSP2

MKSVCLIVFGVVALVATVSAQQKYTDKFDNINVDQVLSNDRILSNYLKCLLEKGPCTQEGRELKKTLPDALRTNCEKCSEKQRTNSRKVISHLESKKPAEWKKLLDKYDPEGIYKSKFEKLNKRS

>AaegCSP3

MERKSTGCNPPQSSPPVLQTRLNRRAPSGEIPSDWDLEWQLFVPRIGIYPRTGKRGIVRCEKKIEREAKKPEENAVLKLNQCANKSGRYECELSSEQKKCFVRVASALLAFANFVKSQDSARNLYSSRYDNLDIDTILGSNRLVNNYVDCLLSRKPCPPEGKDLKRILPEALRTKCARCSVTQKENALKIITTLYYSYPDQYMALRERWDPSGEYHRRFEEYLQGIQFNQIGSNGNDRPVRNDFDRDQSQVLLQTLILSSTTVAPSSPPQPQPQSPNALPNGPEQRPQQLAPSAQLTQPAEATKKLE

>AaegCSP4

MSHKFCWIVVICAISIINVNCYDTKYDNVNLDEIFKSTRLLNNYINCLKNMGPCTPDAKELKELLPDALESECAHCTEKQKVGAERVINFVVDNRPDDFKILESMYDPAGEYRRKYLRDHPNFHDQGAPLTAADATENPPSSNGGDEAPTEESQQNQGQSEDGDDRRR

>AaegCSP5

MKLFAVVALALFAVAAAQEKYTTKYDGVDLDEILKSDRLFNNYYKCLMDQGRCTPDGNELKRVLPDALKTDCAKCSPKQRDGTQKVVNYLIDNRPSQWKNLQAKYDPQNIYVEKYRTEAKKAGIKL

>AaegCSP6

MKYFFVVFLALAATVIAQNEINQYTTKFDSIDVDEILKSDRLFNNYYKCLLDLGRCTPEGVELKRVLPEALETSCAKCSEKQRETSDRAIKYLTENRPEEWKVLKARYDPDNKYSKKNENDA

>AaegCSP7

MKLFIALALLAVAAAQEATYNNRYDNIDVEEILKSDRLFKNYFNCLMDAGPCTPEGTDLKKYLPDALETGCTKCTEKQRDTGNKVIAWLIENRPMEWVMLKSKYDPENKLTERYRELAAKAGIAL

>AaegCSP8

MKIIILCTLLAVVAAQEATYNNRYDNIDVEEILKSDRLFKNYFNCLMDAGPCTPEGTDLKKYLPDALETGCTKCTEKQRDTGNKVIAWLIENRPMEWTMLKNKYDPENKLTERYRELAAKAGIAL

>AaegCSP9

MKFLVAVLSLIAVAAAQDLYTTKFDNIDVDEILKSDRLFKSYYQCLLDEGRCTPEGNELKRSLPDALETGCSKCSEKQRSAGVRAVKYLSENRPTEFKALRNRFDPENKYVEQYVRDAEKEGITLNI

>AaegCSP10

MKIFIAVFTLMAVVAAQEFYTSKFDNIDVDEILKSDRLFKNYYQCLLDQGRCTPEGNELKRVLPDALETACSKCSEKQRSAGVRAVKYLSENRPAEFKALRARFDPENKYVDQYVRDAEKEGITLNIS

>AaegCSP11

MKFFVVALALIAAVAAQDEAMYTSKFDNINLDEILQSDRLFKNYYNCLTDAGPCTPEGNELKRVLPEALETNCAKCSPKQREAGTRAIKHVTENRPEEWKVLRARFDPENKYIERFSAEAEKEGIKL

>AaegCSP12

MDHSSISASKPSAHFKVLNRSFTHKFGATDTIMKIFVVALALIAAVAAQDEAMYTSKFDNINLDEILMSDRLFKNYYNCLTDAGPCTPEGNELKRVLPEALETNCAKCSPKQREAGTRAIKYVTENRAEEWKVLRARFDPEDKYVAQYLAEAEKEGIKL

>AaegCSP13

MKFFIVVLALFAVAAARPQEDKYTTKYDSIDIDEILKSDRLFKNYFNCLMDTGACTPEGNELKRVLPDSLENNCSKCSEKQQTSSTKIIKFLTENKPEEWTMLKAKYDPDNKYVQKYVADADKDGIKL

>AaegCSP14

MKFFIVALALFAAAAARPQEDKYTTKYDSIDIDEILKSDRLFKNYFNCLMDTGACTPEGNELKRVLPDSLENNCSKCSEKQQTSSTKIIKFLTENKPEEWTMLKAKYDPDNKYVQKYVADADKDGIKL

>AaegCSP15

MKFSIVVLALFAVAAAKPQDDKYTTKYDSIDIDEILKSDRLFKNYFNCLMDTGACTPEGNELKRVLPDSLENNCSKCSEKQQTSSTKIIKFLTENKPEEWTMLKAKYDPDNKYVQKYVADADKDGIKL

>AaegCSP16

MKFFIVVLALFAVAAARPQDDKYTTKYDSIDIDEILKSDRLFKNYFNCLMDTGACTPEGNELKRVLPDSLENNCSKCSEKQQTSSTKIIKFLTENKPEEWTMLKAKYDPDNKYVQKYVADADKDGIKL

>AaegCSP17

MKFFIVALVLIAVAAARPQDDKYTTKYDSIDIDEILKSDRLFKNYFNCLMDTGACTPEGNELKRVLPDSLENNCSKCSEKQQTSSTKIIKFLTENKPEEWTMLKAKYDPDNKYVQKYVADADKDGIKL

>AaegCSP18

MKFFIVALVLIAVAAARPQEDKYTTKYDSIDIDEILKSDRLFKNYFNCLMDTGACTPEGNELKRVLPDSLENNCSKCSEKQQTSSTKIIKFLTENKPEEWTMLKAKYDPDNKYVQKYVADADKDGIKL

>AaegCSP19

MKFFIVVLALFAVAAARPQEDKYTTKYDSIDIDEILKSDRLFKNYFNCLMDTGACTPEGNELKRVLPDSLENNCSKCSEKQQTSSTKIIKFLTENKPEEWTMLKAKYDPDNKYVQKYVADADKDGIKL

>AaegCSP20

MKFFIVALALIAVVAAQDDKYTTKYDSIDIDEILKSDRLFKNYFNCLMDTGACTPEGNELKRVLPDALENNCSKCSEKQQTSSTKIIKFLTENKPEAWTMLKAKYDPDNKYVAKYVADADKEGIKL

>AaegCSP21

MKFFIVALALIAVAAARPQDDKYTTKYDSIDIDEILKSDRLFKNYFNCLMDTGACTPEGNELKRVLPDALENNCSKCSEKQQTSSTKIIKFLTENKPEEWTMLKAKYDPDNKYVAKYVADADKKGIKL

>AaegCSP22

MKFFIVALALIAVAAAQDDKYTTKYDSIDIDEILKSDRLFKNYFNCLMDTGACTPEGNELKRVLPDALENNCSKCSEKQQTSSTKIIKFLTENKPEEWTMLKAKYDPDNKYVQKYVAEADKDGIKL

>AaegCSP23

MKFFIVALALFAVAAAQDDKYTTKYDSIDIDEILKSDRLFKNYFNCLMDTGACTPEGNELKRVLPDSLENNCSKCSEKQQTSSTKIIKFLTENKPEEWTMLKAKYDPDNKYVQKYVADADKDGIKL

>AaegCSP24

MKFFIVALALLAVVAAQDDKYTTKYDSVDIDEILKSERLFKNYYACLMDTGACTPDVNELKRVLPDALENNCAKCSEKQQNDSTKTIKYLTENKPEEWKALKAKYDPDNKYVEKYVADADKEGIKL

>AaegCSP25

MKIFIVALALIAVVAAQDDKYTTKYDSVDIDEILKSERLFKNYYACLMDTGACTPDVNELKRVLPDALENNCAKCSEKQQNDSTKTIKYLTENKPEEWKALKAKYDPDNKYVEKYVADADKEGIKL

>AaegCSP26

MKFFIFALALIALAAAKPQDDKYTTKYDSVDIDEILKSERLFKNYYACLMDTGACTPDVNELKRVLPDALENNCAKCSEKQQNDSTKTIKYLTENKPEEWKALKAKYDPDNKYVEKYVADADKEGIKL

>AaegCSP27

MKFFIVALALIALAAAKPQDDKYTTKYDSVDIDEILKSERLFKNYYACLMDTGACTPDVNELKRVLPDALENNCAKCSEKQQNDSTKTIKYLTENKPEEWKALKAKYDPDNKYVEKYVADADKEGIKL

>AaegCSP28

MKIFIVALALIALAAAKPQDDKYTTKYDSVDIDEILKSERLFKNYYACLMDTGACTPDVNELKRVLPEALENNCAKCSEKQQNDSTKTIKFLTENKPEEWKALKAKYDPDNKYVEKYVADADKEGIKL

>AaegCSP29

MKIFIVALALIALAAAKPQDDKYTTKYDSVDIDEILKSERLFKNYYACLMDTGACTPDVNELKRVLPEALENNCAKCSEKQQNDSTKTIKFLTENKPEEWKALKAKYDPDNKYVEKYVADADKEGIKL

>AaegCSP30

MKLFIVALALLAVVAAQDDKYTTKYDSVDIDEILKSERLFKNYYACLMDTGACTPDVNELKRVLPDALENNCAKCSEKQQNDSTKTIKYLTENKPEEWKALKAKYDPDNKYVEKYVADADKEGIKL

>AaegCSP31

MKIFIVALALIALAAAKPQDDKYTTKYDSVDIDEILKSERLFKNYYACLMDTGACTPDVNELKRVLPDALENNCAKCSEKQQNDSTKTIKYLTENKPEEWKALKAKYDPDNKYVEKYVADADKEGIKL

>AaegCSP32

MKIFILCAIMAVVAAQEATYNNRYDNIDVEEILKSDRLFKNYFNCLMDAGPCTPEGTDLKKYLPDALETGCTKCTEKQRDTGNKVIAWLIENRPMEWVMLKSKYDPENKLTERYRELAAKAGIAL

>AaegCSP33

MKLFIALALLAVAAAQEATYNSRYDNIDVEEILKSDRLFKNYFNCLMDAGPCTPEGTDLKKYLPDALETGCTKCTEKQRDTGNKVIAWLIENRPMEWVMLKSKYDPENKLTERYRELAAKAGIAL

>AaegCSP34

MKLFVAVFALIAVVAAQELYTSKFDNIDVDEILKSDRLFKNYYQCLMDEGRCTPEGNELKKILPEALETNCAKCSEKQRDGAIKAFGYLSENRPTEWKTLRDRFDPEGKYIEQYREEAEKNGIKF

>AaegCSP35

MKIMIVIAFALLAVASAQEEQYTTKYDNIDVEEILKSDRLFNNYFKCLMDEGPCTPDGNELKRILPEALQTNCAKCSESQRAGAIKVINYMIENRAEQWKALQEKYDPENIYLEQYRAEAEQSGITL

>AaegCSP36

MKSFIVIALALVVAVAAQNKYTSKYDGVDIDEILKSDRLFNNYYKCLLDQGRCTPDANELKRILPEALKTNCEKCSEKQREGATRVINYLIENRNQQWQTLQAKFDPENIYINQYRNEARAAGIKI

>AaegCSP37

MNSVNRYVLCIALIALFVASFTTAEENCEISANELGKIEQTLTHINQPIYTGDDESEVSDSDQCAQMLRGIHFQLRRLTQKYKLMNKGYVKAEEFAKMARDYEDQLSVLKNDLEQLKIGADSSAKQKMQELKKDIATLEQNVNTLHKDLEGITDELGKVRMDLCLTYMESNQLSNAQDKVKTLAPKYLMELVEQFLNKSEKNWLPVVDLSVAIPDLDDRGQVYKTVHEFLKTKNRDGGEDSILLEAEVLKMNATFHPGSKITEDRKKEIQDLLEKLSLTSTKIFDQWTQDLAKLENSAVYKNSIDRMFLTQMEKFGERVMAKDDYYSLRNFLKLLVVSTNYYKIAAYRKLIQEKIGHTLAVLMFDMMSMERTELQYDPHVPDEVVRMYDESITALPDSLKNIRSCLKLVKIYNHVTNQCILATNEVEDVNNSNPKFKSNVLGRRKLVKTASNDCTPFRLEPSADKASIRIITPKGDALTNINSIQPGLSWFNRVGAPYTNNHNMKLDYSADWILDANYANDSIKIESEFNAYQTMKSVDHLMVTNVGKVPHVVVAQYGLKGMEYAGAGMKDAEWKFKCDN

>AaegCSP38

MKLKVYICQVIFSFLAVSVFCEENCNIPESELSKIDHVLRHMEKPIYSEEQFASDNEECTNLLNGIHAQLRRLTQRYKLMNKGYVKVEEYQRMADDYEKQLKTLNDELVELQQHTSEKASATIAKLKEDIKKLDEEVGTLHEKLKGIKQDFEKVKRDLCVTYLNSNQMSKAKAKLKEMASTYLIEIVQQQLNKSNANIMPMLEFSAAIPDLDDMGEAYKEIYKFLEEQKRLEGEDSVLLEATVLKMNASLKEGSNITDERRTQIEGLLKDLATKSTIVFSTWTKELKKINDAVVIKNALDHMFVSQMKVFGALVGDTSDFGSIRNFVKLTVVCNNYYKVAAYKELIDRKIGNALGTIMFDLLTLEVNEMKFDPHVPDEIPKLFEATLSSLPNSLTELRTCLGKVQIYNKKTNKCVVATGNDFDVHKDKLGDFYRVVVADYGCTSFRLEASGDKASVRIVTPSGNPMSNVNLHLEGNSLHNYVATPKSNKPDRTPSSSDEWILDANYNNDTIKIESQFSDYKTKKTEVDHLLVRDINHLPHVLVARYGFMGLKNSDAKDTIEWNLKCGS

>AaegCSP39

MARGMGCWQPASQWGHKGFGFVTFQSEDVVDKVCEIHFHEINNKMVECKKAQPKEVMLPANLAKTRTAGRGTYDFMWSLGTLPDGFPAAAYAAYAAGRGFSGYPSFGLPYPTGNLNLAALHAHLAAAAATASAGGPHHHHHAHSSSSGNNTTPPPPTRSNPALVQLEATRIDCCLIPGGEHRTTYNSTNMHEMHFNPTSCRMRIVSLVGLVGSDRAYHPPPGTGAGESKLDKGIRHRSSIHSGAYTQLLCIRPNVCEGNYTRVDVAGEFVSTFPARFLDHYIVIVVVRRRSKALSSFDSTPPSTL

>AaegCSP40

MNRQLWIIIFAILCVAQAEEDNPTTEKMEELGIATINNFTREFYSYVEAVSQVLADLELTTTASITQIKHRIKHLLQEKCNLCSAKAEGPALDQGYVTTSNGSVIPVSYEQTRFGGGWIVLMQRYDGTVRFNRSWAEYRDGFGMVGHEFWLGLERIHQMTKDAEYELMIEMQDFEGNYKYAGYDAFAVGPEEERYPLAKVGKFNKTAYVDSFGKHRGYGFSTYDNDDNGCSNQYGRGGWWYYRKSCFGASLTGIWQNKQDWKSISWVWFSTEKKQVPLKFARMMMRLKTAE

>AaegCSP41

MILQFWVVTFSVLFAARADENHSILIKLNDLDHRFTQMFSQQFYRHTQQVTDRVSALKISIDTNLLELDQQIQQALDGIQSNESSSSASATKPPGLTTIPIGSEPRVPALYERERYGGDWLVVMHRYDGSVKFDRTWAEYRDGFGMVGQEFWYGLERLHQLTKEKSYELMVEMEDFNGSLKYAWYDKFVVGPEEQRYALVELGTFNGTTDGDSLKPHKGSGFSTYDNDDFGCSNKYAKGGWWYYSGKCYGSSLTGIWKNELAYSSIVWMKFSDVSNTPLKLVRMMIRPKN

>AaegCSP42

MVIQRVWRSSANSSTSGNTTATNSNSGALTASSLSATSSSELAPQNVPSGEIDGSQVVVPKMQRPKLSGEAMLEVMKMRYSGRSHSSQSAGDAALKSLELLRANIQYLFDKEIEVVVKKFSSLFFIPAIKNIKENLGESAISDDTLKTLYCSLLENSKSQYVGQIASPAESSLSRANTPGMELSDSDSSNDNVVPSGTTSLLQQALKRKLPEPNQHDGFKRQYFLQGSLYSQNHYSILQSLGNVQGSLPYQIRPSVLNPTVYTTTISPETLFIMDFKAGRALGVPDFRDRLANKHPEILRYCPDNQDRDWLLQQKQISPLNRNGRFFLLVLDEVRKLAERNSEYSNNPYMKLSDLQGFKLTEFIYAKVQKLIKDSADSSVKPTTATPSVTTATTAVPNSIQPRPRVSSLSSSHATLTALLSSPQQSQVNCSNSSGTIATIAGSSTTSGMDANTGGTGSGDTKT

>AaegCSP43

MFSKVKSLSTRGAASTCTVRLVLVVVFMLAISQVAAQSSSTTTPEATSSSGNNGSSPSTPNKSQVSDEALDKALNDKRYLMRQLKCALGEVPCDPVGKRLKSLAPFVLRGACPQCTPAELVQIKKTLAHLQRNFPAEWNKLVQTYAG

>DantCSP1

MKLLFAVVVAAFMATIVLADDKYTTKFDNIDVDEILKSDRLFNNYYKCLTDEGKCTPDGRELKKNLPDALQTECSKCSAKQKESSEKILNFIVENKPEEWKVLQAKYDPEGVYITKYREEAKKRGIKV

>DantCSP2

MFRLIWISLFISYLTFIQAVPHPPATTAAPLKQTYDNKFDNIDIDEILGQERLLKNYVKCLEGLGPCTPDGKMLKDIPILETLPDAMATNCAKCTERQKYGSDRVTHFLIDNRPEDWDRLEKIYNPEGSYKKAYLMEKQKLQPTNEDGDAKKD

**Table S5 Primers used for qRT-PCR analysis of olfactory genes in *B. odoriphaga*.**

| Genes | Forward primer (5’ → 3’) | Reverse primer (5’ → 3’) |
| --- | --- | --- |
| **Odorant binding proteins (OBPs)** | | |
| *BodoOBP1* | GATGAAAGATATTGCCCTGAA | GCCTTCTTCCAACAACTG |
| *BodoOBP2* | CACGAAGATGAGGCAATG | ACCCATAAACAAGGCAATG |
| *BodoOBP3* | CAAGCAAAGTGTTTCCGA | GTATCAGCAACTTCATTAGCA |
| *BodoOBP4* | ATGTATTTCCCGCAATGAC | GTATGTAGTAGTAATCGTTAGCAT |
| *BodoOBP5* | GTAAACCACAATTTAATGTCAGT | GAGCATCAATTCCATCATACA |
| *BodoOBP6* | CTCCGTCACAGACAAGAA | TTATTCCTATCAGCATTCATCG |
| *BodoOBP7* | TTGTCGGTATTCAATTCCATT | TTAGAATCGTCGGCAAGT |
| *BodoOBP8* | CGAAAGTTGTTGATGATAAAGG | AAGAAGTTCTCAGCAATGTG |
| *BodoOBP9* | GTGCCAGAGAATGTAATGAAT | TCGGTATCGGATATTGCTT |
| *BodoOBP10* | TAACTACGAGGCAGCAAT | ATTCCATTCGCACTGTCT |
| *BodoOBP11* | AACTATGAGGCATCTCTGAA | TTACTCCATTAGCAGCATCT |
| *BodoOBP12* | AGATGGCAGATGGAATAAGA | TTCGTAGAGCACCTTGTT |
| *BodoOBP13* | CAATGACGATAAGGAAGTGAA | TGCTTATTGTCCGATTGATAC |
| *BodoOBP14* | ACAGAACATGGCATTACAC | CGGTCTTATCGTCAAACAG |
| *BodoOBP15* | CACTACTCAGACCTCCAAA | TTGCCATCACTTGACTTG |
| *BodoOBP16* | TCAACCATCAACCACCAT | TAGATCCTTCAACCGATAGTC |
| *BodoOBP17* | CGGCATCATCAAAGACAA | CTGGCTTGTATAGAGGGATA |
| *BodoOBP18* | AGATAAGGCTCTTCAATGTTAC | TCAGTTGGCATAATGTGATAC |
| *BodoOBP19* | GTATGTGGCGTGAAATGG | CGATGGAATTGATGGAAGTC |
| *BodoOBP20* | TGACATCAGGTTGTAAGTTAAG | AATAATCCGAGCCATCCA |
| *BodoOBP21* | CTGACTGAGACTGGTGATT | AGGTGACTTAGTTCCAACA |
| *BodoOBP22* | AGATACTTGCTCACCGAAA | GGTTATTGTTCCAGCCATC |
| *BodoOBP23* | CACTGGAACAATACAAGACAT | AGTTTGGAATTGCGTTATCA |
| *BodoOBP24* | GCACATAAACCTCCAACAA | TTACACCTCCTTCAACTGATA |
| *BodoOBP25* | CTGTGCATTGCTCAGATG | AAAGGGTTACGGTGTCAA |
| *BodoOBP26* | GATGAAAGATATTGCCCTGAA | GCCTTCTTCCAACAACTG |
| *BodoOBP27* | CACGAAGATGAGGCAATG | ACCCATAAACAAGGCAATG |
| *BodoOBP28* | AGGTGTGGAAATGTTAGGA | ATGAGGAAATAATGCTTTGGAT |
| *BodoOBP29* | CAACACAATGAAAGAGCAAAG | TTCTGTCCACATATCGTTCT |
| *BodoOBP30* | AGGCATCAAAGTGACAGA | TCCAGTTCATATCTTCCATCT |
| *BodoOBP31* | AGAACACCGATTGGACTT | TCACCACCGAATTGCTTA |
| *BodoOBP32* | AACTGGTGTGATGACTGAT | CTTCTTCTTTCTCTGCTCTTG |
| *BodoOBP33* | TGTGGCTCCAGATTCTAAT | AATACTCAAGTAGGTCCGTTA |
| *BodoOBP34* | CGACGAAGTATGGATACCT | TATGAAAGCAGCCAAAGTAAA |
| *BodoOBP35* | AGGAGCAAAGTGTGTGAT | CATCTGTTCCGTGTAGTCA |
| *BodoOBP36* | CAATGTGTTCTAATCGTAATTCG | AGTCCTCGCATCTATCAAG |
| *BodoOBP37* | ATTGGATGTGCTCATGGA | GTCTTTGTCTGTAACCGTTT |
| *BodoOBP38* | AATGTGAGACTGCTGGAA | ATTATCCGCTGTGGTAAGT |
| *BodoOBP39* | TTAAGAACAAGGTTATGTCAGAA | TCATTCCATTTGCGTTAATCT |
| *BodoOBP40* | CACAGACGCCGATATAGA | GTAGTCATCATTCCCATTTGT |
| *BodoOBP41* | AGCATTAAGGACAAGAGTAGA | CACATCCACATTCGTATTATTG |
| *BodoOBP42* | TTATGGAACAGGTCACAGATA | CACACATCAGCAAGATCATTA |
| *BodoOBP43* | GCATTCTCTAATTATCAACAACG | ACCACACGATCCACATAA |
| *BodoOBP44* | TTTACACCGAGGGCATAG | AGCACTCTTCAACCACAA |
| *BodoOBP45* | AAAGGTGGACATAACATTCG | GCTCCTGCTTCACATCTA |
| *BodoOBP46* | TCGGAAGATTTGATTGAACAA | ATATTACCTGCGTCGTCATA |
| *BodoOBP47* | ACATCCAATGCTTCCAGA | TTGCCTCATCCTTCTTGT |
| *BodoOBP48* | GCGATAAAGTGACCGATAC | AACACATTTGGGCAGATTT |
| *BodoOBP49* | TCGGTTGGTGGTAGAATG | TCAGTAGACTTGCGTGTT |
| **Chemosensory proteins (CSPs)** | | |
| *BodoCSP1* | AGACAAATACACCACCAAAT | TTCCCTTATCCATCAAACATT |
| *BodoCSP2* | ATGTTGTGGATGGTGATG | CTTAGAATAACTTCAGGTAATGC |
| *BodoCSP3* | CCATTCAGTCTAAGTGTT | TTGTCTTATAGTCATCATCT |
| *BodoCSP4* | ACGAAGAAATACGATAACG | TATGGCATCAGGTAGAAG |
| *BodoCSP5* | TGCTGAAGAAGTTGTATG | CACCAGATATTGAGTTGAA |
| **Reference genes** | | |
| *RPS15* | ATCGTGGCGTCGATTTGGAT | CTCATTTGGTGGGGCTTCCT |
| *RPL18* | TTCCCTTATCCATCAAACATT | CTTAGAATAACTTCAGGTAATGC |

**Table S6 Quality metrics of Unigenes in *B. odoriphaga* transcriptomes.**

| Sample | Total Number | Total Length | Mean Length | N50 | N70 | N90 | GC (%) |
| --- | --- | --- | --- | --- | --- | --- | --- |
| FA-1 | 26818 | 26793946 | 999 | 1595 | 980 | 412 | 38.15 |
| FA-2 | 27373 | 26674663 | 974 | 1529 | 947 | 403 | 38.03 |
| FA-3 | 30514 | 34346738 | 1125 | 1891 | 1145 | 448 | 38.59 |
| FB-1 | 22165 | 30082994 | 1357 | 2226 | 1459 | 575 | 40.07 |
| FB-2 | 21777 | 28448430 | 1306 | 2139 | 1395 | 548 | 40.21 |
| FB-3 | 20839 | 28256513 | 1355 | 2196 | 1445 | 587 | 40.26 |
| MA-1 | 28436 | 29313149 | 1030 | 1644 | 1016 | 428 | 38.49 |
| MA-2 | 27882 | 32092351 | 1151 | 1927 | 1166 | 468 | 38.54 |
| MA-3 | 27104 | 28301675 | 1044 | 1681 | 1037 | 432 | 38.36 |
| MB-1 | 33625 | 35984840 | 1070 | 1804 | 1050 | 418 | 38.37 |
| MB-2 | 34690 | 45446910 | 1310 | 2229 | 1371 | 535 | 38.53 |
| MB-3 | 34252 | 41908291 | 1223 | 2188 | 1268 | 470 | 38.56 |
| All-Unigene | 55867 | 86711434 | 1552 | 2806 | 1701 | 655 | 38.08 |

N50: a weighted median statistic that 50% of the Total Length is contained in Unigenes great than or equal to this value. GC (%): the percentage of G and C bases in all Unigenes.

FA: female antennae; FB: female body; MA: male antennae; MB: male body.

**Table S7 Summary of functional annotation of *B. odoriphaga* transcriptomes.**

| Values | Total | Nr-Annotated | Nt-Annotated | Swissprot-Annotated | KEGG-Annotated | COG-Annotated | Interpro-Annotated | GO-Annotated | Overall |
| --- | --- | --- | --- | --- | --- | --- | --- | --- | --- |
| Number | 55,867 | 32,492 | 17,867 | 26,930 | 26,289 | 15,633 | 26,541 | 11,578 | 35,013 |
| Percentage | 100% | 58.16% | 31.98% | 48.20% | 47.06% | 27.98% | 47.51% | 20.72% | 62.67% |

Overall: the number of Unigenes which be annotated with at least one functional database.

**Table S8 Conserved domains of odorant binding proteins in *B. odoriphaga*.**

| Proteins | Domains | Domain definition | Position (AA) | E-value | Interpro family | GO function |
| --- | --- | --- | --- | --- | --- | --- |
| BodoOBP1 | 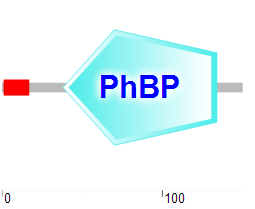 | Insect pheromone/odorant binding protein domains | 38 to 134 | 3.42e-21 | IPR006170  PBP_GOBP | odorant binding (GO:0005549) |
| BodoOBP2 | 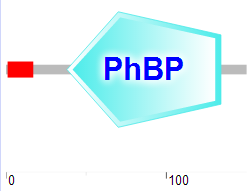 | Insect pheromone/odorant binding protein domains | 38 to 134 | 5.78e-19 | IPR006170  PBP_GOBP | odorant binding (GO:0005549) |
| BodoOBP3 | 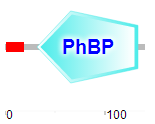 | Insect pheromone/odorant binding protein domains | 32 to 133 | 8.72e-5 | IPR006170  PBP_GOBP | odorant binding (GO:0005549) |
| BodoOBP4 | 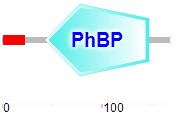 | Insect pheromone/odorant binding protein domains | 43 to 147 | 1.01e-9 | IPR006170  PBP_GOBP | odorant binding (GO:0005549) |
| BodoOBP5 | 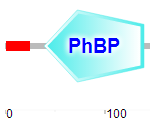 | Insect pheromone/odorant binding protein domains | 39 to 139 | 1.13e-12 | IPR006170  PBP_GOBP | odorant binding (GO:0005549) |
| BodoOBP6 | 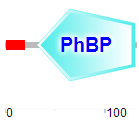 | Insect pheromone/odorant binding protein domains | 32 to 130 | 1.16e-18 | IPR006170  PBP_GOBP | odorant binding (GO:0005549) |
| BodoOBP7 | 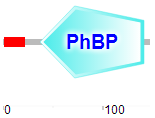 | Insect pheromone/odorant binding protein domains | 37 to 141 | 4.78e-2 | IPR006170  PBP_GOBP | odorant binding (GO:0005549) |
| BodoOBP8 | 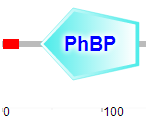 | Insect pheromone/odorant binding protein domains | 38 to 137 | 1.56e-15 | IPR006170  PBP_GOBP | odorant binding (GO:0005549) |
| BodoOBP9 | 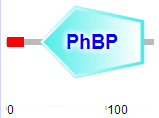 | Insect pheromone/odorant binding protein domains | 34 to 138 | 4.29e-4 | IPR006170  PBP_GOBP | odorant binding (GO:0005549) |
| BodoOBP10 | 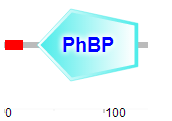 | Insect pheromone/odorant binding protein domains | 33 to 134 | 1.49e-12 | IPR006170  PBP_GOBP | odorant binding (GO:0005549) |
| BodoOBP11 | 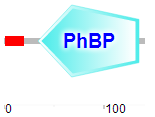 | Insect pheromone/odorant binding protein domains | 34 to 135 | 7.46e-13 | IPR006170  PBP_GOBP | odorant binding (GO:0005549) |
| BodoOBP12 | 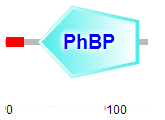 | Insect pheromone/odorant binding protein domains | 33 to 134 | 2.98e-12 | IPR006170  PBP_GOBP | odorant binding (GO:0005549) |
| BodoOBP13 | 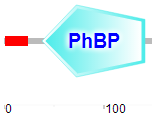 | Insect pheromone/odorant binding protein domains | 38 to 141 | 6.10e-18 | IPR006170  PBP_GOBP | odorant binding (GO:0005549) |
| BodoOBP14 | 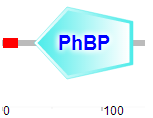 | Insect pheromone/odorant binding protein domains | 32 to 131 | 1.79e-24 | IPR006170  PBP_GOBP | odorant binding (GO:0005549) |
| BodoOBP15 | 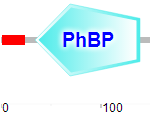 | Insect pheromone/odorant binding protein domains | 34 to 139 | 3.08e-5 | IPR006170  PBP_GOBP | odorant binding (GO:0005549) |
| BodoOBP16 | 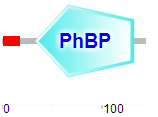 | Insect pheromone/odorant binding protein domains | 33 to 132 | 2.19e-10 | IPR006170  PBP_GOBP | odorant binding (GO:0005549) |
| BodoOBP17 | 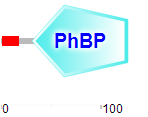 | Insect pheromone/odorant binding protein domains | 31 to 127 | 2.88e-5 | IPR006170  PBP_GOBP | odorant binding (GO:0005549) |
| BodoOBP18 | 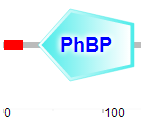 | Insect pheromone/odorant binding protein domains | 35 to 131 | 6.90e-17 | IPR006170  PBP_GOBP | odorant binding (GO:0005549) |
| BodoOBP19 | 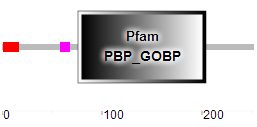 | PBP/GOBP family domain | 76 to 204 | 9.90e-8 | IPR006170  PBP_GOBP | odorant binding (GO:0005549) |
| BodoOBP20 | 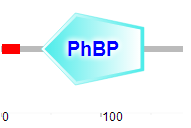 | Insect pheromone/odorant binding protein domains | 40 to 143 | 5.28e-4 | IPR006170  PBP_GOBP | odorant binding (GO:0005549) |
| BodoOBP21 | 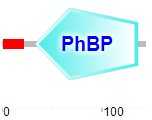 | Insect pheromone/odorant binding protein domains | 33 to 136 | 8.51e-8 | IPR006170  PBP_GOBP | odorant binding (GO:0005549) |
| BodoOBP22 | 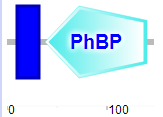 | Insect pheromone/odorant binding protein domains | 38 to 140 | 5.20e-12 | IPR006170  PBP_GOBP | odorant binding (GO:0005549) |
| BodoOBP23 | 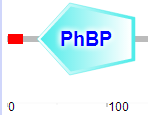 | Insect pheromone/odorant binding protein domains | 30 to 128 | 4.48e-16 | IPR006170  PBP_GOBP | odorant binding (GO:0005549) |
| BodoOBP24 | 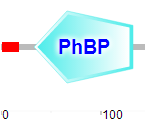 | Insect pheromone/odorant binding protein domains | 33 to 132 | 2.77e-7 | IPR006170  PBP_GOBP | odorant binding (GO:0005549) |
| BodoOBP25 | 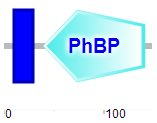 | Insect pheromone/odorant binding protein domains | 39 to 141 | 3.73e-13 | IPR006170  PBP_GOBP | odorant binding (GO:0005549) |
| BodoOBP26 | 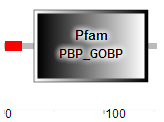 | PBP/GOBP family domain | 30 to 144 | 2.70e-6 | IPR006170  PBP_GOBP | odorant binding (GO:0005549) |
| BodoOBP27 | 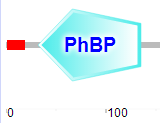 | Insect pheromone/odorant binding protein domains | 32 to 135 | 0.908 | IPR006170  PBP_GOBP | odorant binding (GO:0005549) |
| BodoOBP28 | 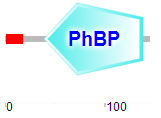 | Insect pheromone/odorant binding protein domains | 39 to 138 | 1.68e-20 | IPR006170  PBP_GOBP | odorant binding (GO:0005549) |
| BodoOBP29 | 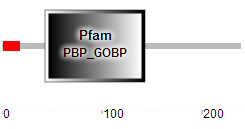 | PBP/GOBP family domain | 43 to 143 | 3.70e-5 | IPR006170  PBP_GOBP | odorant binding (GO:0005549) |
| BodoOBP30 | 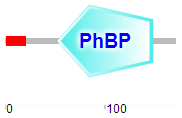 | Insect pheromone/odorant binding protein domains | 52 to 148 | 2.94e-2 | IPR006170  PBP_GOBP | odorant binding (GO:0005549) |
| BodoOBP31 | 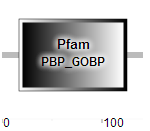 | PBP/GOBP family domain | 17 to 125 | 5.90e-9 | IPR006170  PBP_GOBP | odorant binding (GO:0005549) |
| BodoOBP32 | 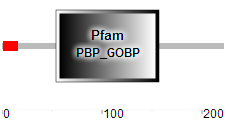 | PBP/GOBP family domain | 54 to 157 | 2.30e-5 | IPR006170  PBP_GOBP | odorant binding (GO:0005549) |
| BodoOBP33 | 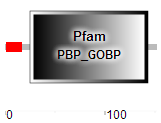 | PBP/GOBP family domain | 25 to 143 | 1.60e-8 | IPR006170  PBP_GOBP | odorant binding (GO:0005549) |
| BodoOBP34 | 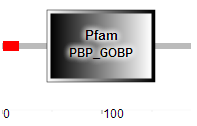 | PBP/GOBP family domain | 45 to 153 | 1.30e-4 | IPR006170  PBP_GOBP | odorant binding (GO:0005549) |
| BodoOBP35 | 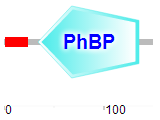 | Insect pheromone/odorant binding protein domains | 34 to 135 | 2.27e-3 | IPR006170  PBP_GOBP | odorant binding (GO:0005549) |
| BodoOBP36 | 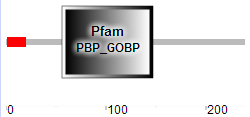 | PBP/GOBP family domain | 56 to 147 | 1.10e-5 | IPR006170  PBP_GOBP | odorant binding (GO:0005549) |
| BodoOBP37 | 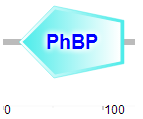 | Insect pheromone/odorant binding protein domains | 16 to 121 | 2.08e-2 | IPR006170  PBP_GOBP | odorant binding (GO:0005549) |
| BodoOBP38 | 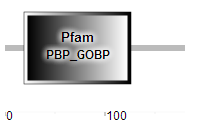 | PBP/GOBP family domain | 19 to 126 | 2.50e-9 | IPR006170  PBP_GOBP | odorant binding (GO:0005549) |
| BodoOBP39 | 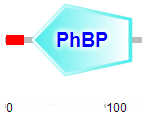 | Insect pheromone/odorant binding protein domains | 29 to 127 | 5.04e-10 | IPR006170  PBP_GOBP | odorant binding (GO:0005549) |
| BodoOBP40 | 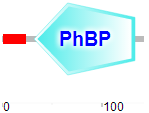 | Insect pheromone/odorant binding protein domains | 32 to 133 | 1.56e-6 | IPR006170  PBP_GOBP | odorant binding (GO:0005549) |
| BodoOBP41 | 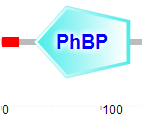 | Insect pheromone/odorant binding protein domains | 33 to 129 | 6.87e-2 | IPR006170  PBP_GOBP | odorant binding (GO:0005549) |
| BodoOBP42 | 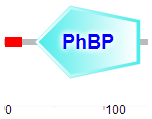 | Insect pheromone/odorant binding protein domains | 31 to 136 | 5.79e-10 | IPR006170  PBP_GOBP | odorant binding (GO:0005549) |
| BodoOBP43 | 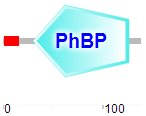 | Insect pheromone/odorant binding protein domains | 30 to 126 | 3.88e-11 | IPR006170  PBP_GOBP | odorant binding (GO:0005549) |
| BodoOBP44 | 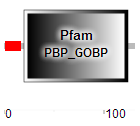 | PBP/GOBP family domain | 20 to 124 | 1.80e-7 | IPR006170  PBP_GOBP | odorant binding (GO:0005549) |
| BodoOBP45 | 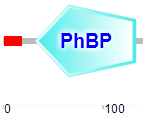 | Insect pheromone/odorant binding protein domains | 32 to 133 | 7.08e-5 | IPR006170  PBP_GOBP | odorant binding (GO:0005549) |
| BodoOBP46 | 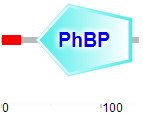 | Insect pheromone/odorant binding protein domains | 35 to 131 | 2.04e-19 | IPR006170  PBP_GOBP | odorant binding (GO:0005549) |
| BodoOBP47 | 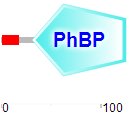 | Insect pheromone/odorant binding protein domains | 31 to 125 | 1.11e-6 | IPR006170  PBP_GOBP | odorant binding (GO:0005549) |
| BodoOBP48 | 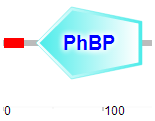 | Insect pheromone/odorant binding protein domains | 33 to 139 | 1.49e-3 | IPR006170  PBP_GOBP | odorant binding (GO:0005549) |
| BodoOBP49 | 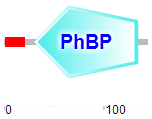 | Insect pheromone/odorant binding protein domains | 30 to 134 | 0.51 | IPR006170  PBP_GOBP | odorant binding (GO:0005549) |

**Table S9 Conserved domains of chemosensory proteins in *B. odoriphaga*.**

| Proteins | Domains | Domain definition | Position (AA) | E-value | Interpro family |
| --- | --- | --- | --- | --- | --- |
| BodoCSP1 | 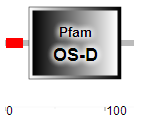 | Insect pheromone-binding family, A10/OS-D | 24 to 116 | 3.60e-39 | IPR005055  *OS_D_A10/PebIII* |
| BodoCSP2 | 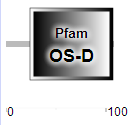 | Insect pheromone-binding family, A10/OS-D | 24 to 107 | 1.20e-17 | IPR005055  *OS_D_A10/PebIII* |
| BodoCSP3 | 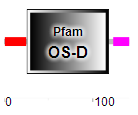 | Insect pheromone-binding family, A10/OS-D | 26 to 118 | 3.30e-36 | IPR005055  *OS_D_A10/PebIII* |
| BodoCSP4 | 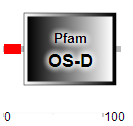 | Insect pheromone-binding family, A10/OS-D | 21 to 113 | 4.80e-36 | IPR005055  *OS_D_A10/PebIII* |
| BodoCSP5 | 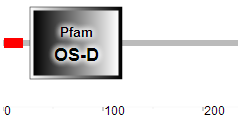 | Insect pheromone-binding family, A10/OS-D | 27 to 119 | 1.50e-29 | IPR005055  *OS_D_A10/PebIII* |
